# Drug Repurposing in Rare Diseases: An Integrative Study of Drug Screening and Transcriptomic Analysis in Nephropathic Cystinosis

**DOI:** 10.3390/ijms222312829

**Published:** 2021-11-27

**Authors:** Francesco Bellomo, Ester De Leo, Anna Taranta, Laura Giaquinto, Gianna Di Giovamberardino, Sandro Montefusco, Laura Rita Rega, Anna Pastore, Diego Luis Medina, Diego Di Bernardo, Maria Antonietta De Matteis, Francesco Emma

**Affiliations:** 1 Renal Diseases Research Unit, Bambino Gesù Children’s Hospital, IRCCS, 00165 Rome, Italy; ester.deleo@opbg.net (E.D.L.); anna.taranta@opbg.net (A.T.); laurarita.rega@opbg.net (L.R.R.); 2 Telethon InstituFte of Genetics and Medicine, 80078 Naples, Italy; giaquinto@tigem.it (L.G.); s.montefusco@tigem.it (S.M.); medina@tigem.it (D.L.M.); dibernardo@tigem.it (D.D.B.); dematteis@tigem.it (M.A.D.M.); 3 Research Biobank, Bambino Gesù Children’s Hospital, IRCCS, 00165 Rome, Italy; gianna.digiovamberardino@opbg.net; 4 Management Diagnostic Innovations Research Unit, Bambino Gesù Children’s Hospital, IRCCS, 00165 Rome, Italy; anna.pastore@opbg.net; 5 Department of Chemical, Materials and Industrial Production Engineering, University of Naples Federico II, 80138 Naples, Italy; 6 Department of Medical Biotechnologies and Molecular Medicine, University of Naples Federico II, 80138 Naples, Italy; 7 Division of Nephrology, Department of Pediatric Subspecialties, Bambino Gesù Children’s Hospital, IRCCS, 00165 Rome, Italy

**Keywords:** cystinosis, drug repositioning, high throughput screening, transcriptome, high content screening

## Abstract

Diagnosis and cure for rare diseases represent a great challenge for the scientific community who often comes up against the complexity and heterogeneity of clinical picture associated to a high cost and time-consuming drug development processes. Here we show a drug repurposing strategy applied to nephropathic cystinosis, a rare inherited disorder belonging to the lysosomal storage diseases. This approach consists in combining mechanism-based and cell-based screenings, coupled with an affordable computational analysis, which could result very useful to predict therapeutic responses at both molecular and system levels. Then, we identified potential drugs and metabolic pathways relevant for the pathophysiology of nephropathic cystinosis by comparing gene-expression signature of drugs that share common mechanisms of action or that involve similar pathways with the disease gene-expression signature achieved with RNA-seq.

## 1. Introduction

Rare diseases have increasingly become a public health priority [1]. Epidemiological data show that taken together, rare diseases affect between 3.5% and 5.9% of the global population [1]. The high number and the heterogeneity of rare diseases limit the interest of pharmaceutical industries in the development of new treatments. To this end, drug-repositioning strategies using cell-based phenotypic assays in combination with bioinformatics analysis tools, may represent successful strategies to identify new therapies [2].

Nephropathic cystinosis [OMIM #219800] is a rare genetic disorder that develops during the first months of life, causing end-stage kidney disease and other extra-renal complications that are secondary to lysosomal cystine accumulation [3,4]. Cysteamine treatment facilitates clearance of lysosomal cystine and is the current standard of care for cystinosis [5]. However, cysteamine does not treat the proximal tubular dysfunction of cystinosis (i.e., renal Fanconi syndrome) and delays but cannot prevent progression of kidney failure [6]. It also reduces the incidence of other complications, but it is unclear that these can be prevented in the long-term [6,7].

**Figure 1:**
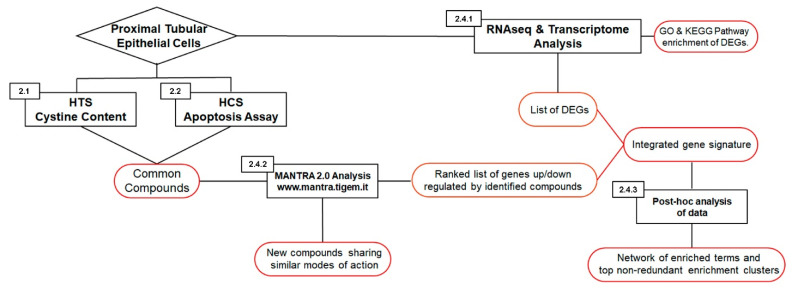
Flow diagram of the study.

In this work, we used a drug repositioning approach to find new treatments for cystinosis by combining a conventional “activity-based” strategy with in silico analysis. First, we performed a high-throughput screening to identify molecules that reduce cystine accumulation and a secondary high-content screening to identify molecules that also prevent apoptosis. These analyses were performed using conditionally immortalized proximal tubular epithelial cells obtained from patients bearing the classical homozygous 57-kb deletion that removes the first 10 exons of the *CTNS* gene (*CTNS*^−/−^ ciPTECs). These screenings were achieved using the Prestwick chemical library, which contained at least 1200 small molecules, 98% of them were off-patent drugs already approved by the FDA, EMA, JAN, and other agencies. We then performed a transcriptome analysis to identify differentially expression genes (DEGs) by comparing wild type and cystinotic cells. Finally, these data were combined in silico with online gene expression datasets obtained from cells and tissues exposed to the compounds that were selected in the drug-screening. This approach allowed identifying new drugs able to reduce cystine content and prevent apoptosis, and new metabolic pathways that could be involved in the pathophysiology of nephropathic cystinosis (Figure 1).

## 2. Results

### 2.1. High Throughput Screening Based on Cell Cystine Content

**Figure 2:**
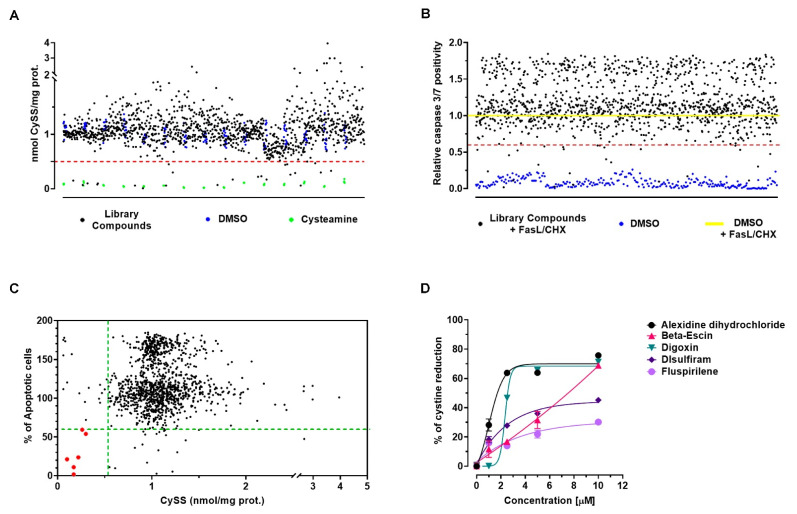
Drug screening for cystine content and apoptosis. (**A**) High throughput screening for cystine level normalized for protein content in *CTNS*^−/−^ ciPTECs treated with 1200 small molecules of Prestwick chemical library and incubated for 24 h. Black dots: individual library compound at a final concentration of 10 µM; blue dots: DMSO (vehicle as negative control); green dots: cells treated with 100 µM of cysteamine (positive control). (**B**) High-content screening of small molecules that reduce apoptosis in *CTNS*^−/−^ ciPTECs. Relative caspase 3/7 positivity of each well was shown; percent values of each well were normalized with the average percent of apoptosis in untreated cells exposed to apoptosis stimuli (yellow line) of each plate. Each black dot represents the mean value obtained with each compound. Blue dots show the same results for non-induced cells that were exposed to vehicle. After plotting the results, an arbitrary threshold was selected, which made it possible to identify 27 compounds that reduced by at least of 40% the apoptosis rate (red dash line). All experiments were performed in triplicates in different plates. (**C**) HTS and HCS data sets for cystine content and apoptosis rate were combined in a single plot, allowing to identify 6 drugs that potentially corrected both phenotypes (red dots). (**D**) Dose-response curve of cystine-depleting lead compounds was generated using four-parameters logistic regression model to interpolate data.

High lysosomal cystine content is the hallmark of cystinosis. We have therefore chosen to perform a high-throughput screening to identify drugs capable of reducing intracellular cystine levels. Preliminary experiments were performed to find the minimum number of cells that allowed accurate cystine measurement using an HPLC-based assay, and to optimize protein quantification using a limited number of sample manipulations (see Methods). These tests showed that cystine could be accurately measured in 1 × 10^4^ *CTNS*^−/−^ ciPTECs (Supplementary Figure S1A). Comparison of cystine levels in cells treated with vehicle or with cysteamine, which in vitro clears nearly all lysosomal cystine content, showed a Z-factor for this assay of 0.83 (Supplementary Figure S1B). Screening of the Prestwick chemical library at a final concentration of 10 µM for 24 h, identified 24 drugs that reduced cystine levels by ≥50% (Figure 2A). Confirmatory assay was then performed with 1 × 10^5^ cells, showing the efficacy of selected molecules in reducing cell cystine content (Supplementary Figure S1C). Notably, these drugs belong to different therapeutic groups (listed in Supplementary Table S1).

### 2.2. High-Content Drug Screening Based on Apoptosis Assay

Cystinotic cells are known to be more sensitive to pro-apoptotic stimuli than *CTNS*^+/+^ cells [8,9,10,11]. We performed as secondary screening an imaging-based high content screening using the Prestwick chemical library to identify drugs that, in addition to reducing cystine content, also protect cystinotic cells from apoptosis. In agreement with previous studies, our preliminary data confirmed higher sensitivity of *CTNS*^−/−^ ciPTEC cells to undergo apoptosis (data not shown).

Therefore, *CTNS*^−/−^ ciPTECs were pretreated for 1 h with compounds of the chemical library at a final concentration of 10 µM. Apoptosis was then induced, and caspase-3/7 activation was analyzed using specific dyes after 5 h. We found 27 compounds that reduced apoptosis by ≥40% compared to untreated cells (Figure 2B and Supplementary Table S2).

### 2.3. Selection of Candidate Drugs by Crossing Data Resulting from the Two Screenings

In the next step, we crossed data from the two screenings and identified 6 drugs that reduced cystine content and also prevented apoptosis (Figure 2C). Alexidine dihydrochloride is an antibacterial agent that it has recently been demonstrated to have also an anticancer activity by targeting the mitochondrial tyrosine phosphatase PTPMT1 [12]. Auranofin, an anti-rheumatic agent, strongly reduces activation of the NLRP3 inflammasome and inhibits activity of cystine-glutamate antiporter, system Xc [13]. Beta-escin, a natural mixture of triterpene saponins is mainly known for its anti-edematous, anti-inflammatory and venotonic properties; it has a glucocorticoid-like activity and may modulate lysosomal stability by acting on cholesterol homeostasis [14]. Digoxin is one of the oldest cardiovascular drugs that originates from the Digitalis plant. Digoxin may inhibit Nrf2 signaling pathway [15,16] and recent preclinical proof of concept studies showed that, likewise to alexidine dihydrochloride, it engages TFEB activation mechanisms via three distinct Ca^2+^ sources and Ca^2+^-sensing pathways [17]. Disulfiram, used to treat alcohol dependence, is primarily an aldehyde dehydrogenase inhibitor, and it recently emerged as a promising candidate in cancer therapy through regulation of the AKT-FOXO axis [18]. Finally, Fluspirilene, a neuroleptic drug with antagonist action of dopamine D2 receptor and Ca^2+^ channel-blocking activity [19].

Dose–response of cystine-depleting lead compounds was performed to confirm the results of primary screening, compare the fits of each treatment, and find the lowest concentration with the best response. The compounds were tested in *CTNS*^−/−^ ciPTECs for 24 h at final concentration of 10–5–2.5 and 1 µM. Auranofin showed high toxicity and was discarded, the other compounds induced significant reduction of cystine content even at lowest concentration (Figure 2D).

### 2.4. Identification of Metabolic Pathways Altered in Cystinosis and Potentially Modulated by Selected Compounds

Although many pathophysiological aspects of cystinosis have been clarified, many unknowns still remain. Therefore, we performed a transcriptome analysis comparing *CTNS*^+/+^ and *CTNS*^−/−^ ciPTECs in the intent of identifying biological processes that are altered in cystinosis. Then the transcriptional profile, obtained by transcriptome, was compared with the gene-expression signature of lead compounds, suggested by the free online software MANTRA 2.0: https://mantra.tigem.it (accessed on 18 October 2021). This post-hoc analysis allowed to show metabolic pathways that possibly could be corrected by selected compounds.

#### 2.4.1. RNA-Seq Analysis and Study of Differentially Expressed Genes

**Figure 3:**
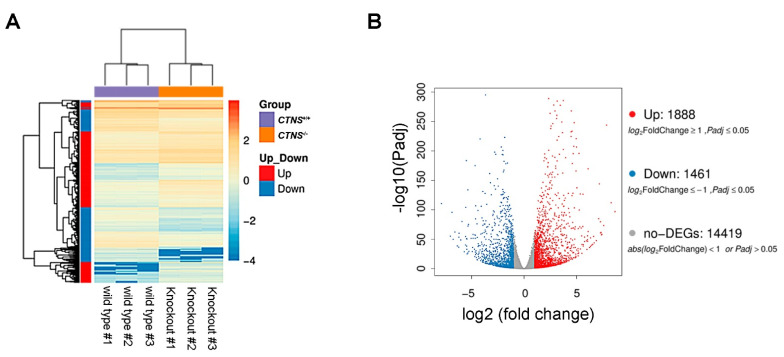
Transcriptome analysis. (**A**) Triplicate of *CTNS*^+/+^ (wild-type) and *CTNS*^−/−^ (knockout) samples are represented on the *x* axis of the heatmap. DEGs are reported on the *y* axis and color represents the log10 transformed gene expression level from weak (low expression) to strong (high expression). (**B**) Volcano plot showing DEGs obtained from the RNA-seq dataset with log2 transformed fold change in abscissa and −log10 transformed significance in ordinate (all data in triplicates). Upregulated genes are represented as red points, downregulated genes represented as blue points and no-DEGs represented as gray points.

We performed triplicate RNA-seq analyses comparing *CTNS*^+/+^ and *CTNS*^−/−^ ciPTECs. Based on gene expression levels, a number of DEGs were identified by using DEseq2 algorithms [20] (Figure 3A). Specifically, after applying an FDR < 0.05, 3349 genes showed significant changes between *CTNS*^+/+^ and *CTNS*^−/−^ ciPTECs. Among them, 1888 were upregulated (log2 Fold Change ≥ 1) and 1461 were downregulated (log2 fold change ≤1) (Figure 3B and Supplementary Table S3).

**Figure 4:**
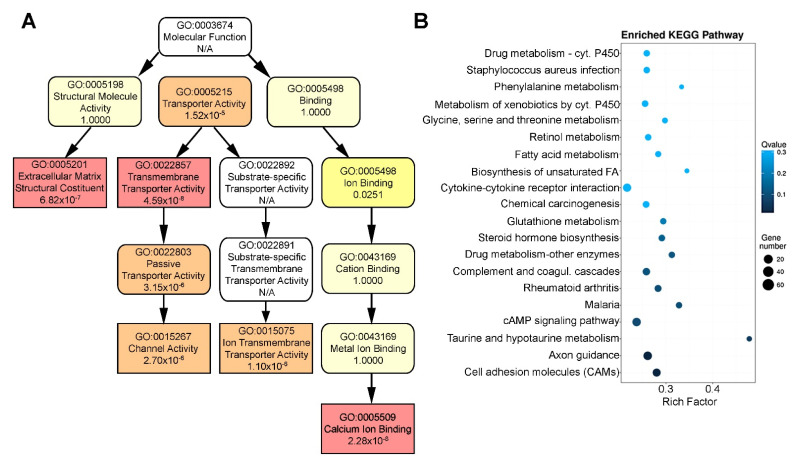
Gene Ontology and pathway analysis of DEGs. (**A**) GO functional enrichment of DEGs represented in the directed acyclic graph where each node shows the name of the GO term and the *p*-value. The darker (red) color corresponds to the lower *p*-value which indicates the more significant enrichment. (**B**) The top 20 functionally enriched KEGG pathways found in the analysis of DEGs in *CTNS^−/−^* vs. *CTNS^+/+^* ciPTECs, in order of significance from bottom to top.

To gain an insight into the biological functions of the most significantly up- or downregulated genes in cystinotic cells, Gene Ontology enrichment analysis was performed. The enriched GO output containing down- and upregulated genes was analyzed and presented with a directed acyclic graph (DAG). The analysis identified significant modulation of genes associated with extracellular matrix structural components (GO:0005201), with transmembrane transporter activity (GO:0022857) and calcium binding and signaling (GO:0005509) (Figure 4A). In particular, as reported in Supplementary Table S3, we found altered expression of numerous genes of COL gene family, showing a potential role of collagen in cystine crystallization, already observed in stromal cornea [21], also in tubular cells. Interestingly, the analysis highlighted a perturbation of 79 solute carrier (SLC) groups belonging to 30 different families; 13 ATP-binding cassette transporters (ABC transporters) belonging to 4 of 7 families classified by the Human Genome Organization (HUGO); and 26 potassium voltage-gated channels (KCN), which could be the cause of indirect pathological effects [22,23]. Modulated genes, included in calcium binding and signaling GO enriched output, were primarily represented by protocadherins (PCDH), a large family of calcium-dependent cell–cell adhesion molecules.

All the DEGs were also mapped to the reference pathway in the (Kyoto Encyclopedia of Genes and Genomes) KEGG database to further investigate changes in cystinosis. Functional enrichment of pathways was shown in Figure 4B, and specifically, a significant change emerged in the expression of genes involved in cell adhesion (claudins, CLDN; junctional adhesion molecule 3, JAM3; platelet endothelial cell adhesion molecule 1, PECAM1); taurine and hypotaurine metabolism (gamma-glutamyltransferases, GGT1, GGT2, GGTLC2); and cAMP signaling (hydroxycarboxylic acid receptors, HCARs; phosphodiesterase, PDE4).

#### 2.4.2. MANTRA Analysis

**Figure 5:**
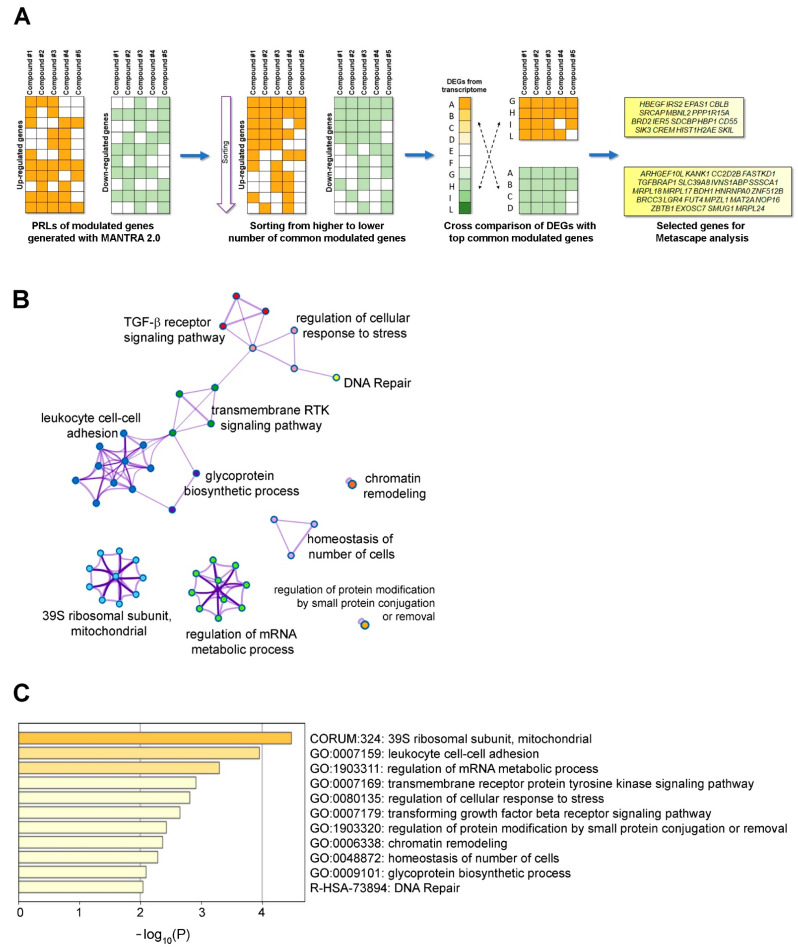
Post hoc analysis of DEGs of *CTNS*^−/−^ ciPTECs. (**A**) The first step of the pipeline is a schematic representation of the MANTRA output, with the prototype ranked list (PRL) of genes potentially up- or downregulated by each compound; then, lists of genes are organized in order to have at the top those modulated from the highest number of compounds; finally, it was selected upregulated genes, and simultaneously modulated from at least four compounds, which have the downregulated counterpart in the list of DEGs obtained from transcriptome, and vice versa. Identified genes are analyzed with web application Metascape. (**B**) Network of enriched terms where each node represents an enriched term that is colored by its cluster ID and the thicker of edge link represents their similarity. (**C**) The bar graph lists the top 11 clusters with their representative enriched terms; color scale is proportional to the statistical significance of the analysis.

In parallel, we performed an additional analysis with the MANTRA 2.0 software (https://mantra.tigem.it/, accessed on 18 October 2021). As described by Iorio et al. 2010, MANTRA 2.0 is a collaborative on-line resource that integrates, by an aggregation algorithm, gene expression profiles of multiple human cell lines following treatment with bioactive small molecules available in public data (https://clue.io/cmap, accessed on 18 October 2021). Therefore, MANTRA generated for each analyzed compound a Prototype Ranked List (PRL) [24,25]. We downloaded PRLs of the five lead compounds and selected those genes that were commonly up- or downregulated for the next analyses (Supplementary Table S4 and Figure 5A).

Furthermore, MANTRA can show the similarity relationship across the small molecules by representing it as a network. We thus identified 38 additional compounds that may have similar activity to the five selected candidate drugs (Supplementary Figure S3).

#### 2.4.3. Post Hoc Analysis of Data Generated by RNA-Seq and MANTRA Analysis

To identify common metabolic pathways and/or cell processes modulated by selected compounds, we compared DEGs identified by transcriptome analysis (listed in Supplementary Table S3) with the ranked list of modulated genes generated by MANTRA analysis (listed in Supplementary Table S4) as shown in the pipeline of Figure 5A.

In this way we found 16 downregulated and 23 upregulated genes in *CTNS*^−/−^ ciPTECs that were theoretically up- and downregulated, respectively, by at least four of selected compounds (Figure 5A). Therefore, these genes were analyzed by Metascape (http://metascape.org, accessed on 18 October 2021) to conduct pathway and process enrichment analysis by using multiple ontology sources (see Figure 5B,C) [26]. The analysis showed an involvement of the 39S mitochondrial ribosomal subunit (CORUM:324), leucocyte cell–cell adhesion (GO:0007159), and regulation of mRNA metabolic process (GO:1903311). In addition, the gene set analyzed also showed a regulation of transmembrane receptor protein tyrosine kinase signaling pathway (GO:0007169), of TGF-β receptor signaling pathway (GO:0007179) and the involvement of cell response to stress (GO:0080135). Interestingly, this analysis, which showed an involvement of a mitochondrial ribosomal subunit, supported recent finding about the critical role of mitochondrial translation apparatus to cellular health [27,28,29]. In addition, the analysis highlighted that the selected drugs probably induced a signaling cascade that, through the modulation of specific genes related to metabolic processes, resulted also in antioxidant action.

## 3. Discussion

Low availability and high costs of drugs remain a significant challenge in the treatment of patients with rare diseases. Despite growing incentives, the annual investment per capita is still limited [30]. High-throughput drug screenings usually yield encouraging data, but further developments are often not pursued as they are deemed too expensive considering the rarity of the diseases [31]. Drug repurposing/repositioning is increasingly becoming an alternative approach because the pharmacokinetic, pharmacodynamics and toxicity profiles of candidate drugs have already been characterized in humans. This approach considerably reduces the development costs [32,33]. In addition, recent advances in omics science have generated large databases and online computational pharmacology tools, allowing “in silico drug repurposing” [34,35].

The combined drug repositioning strategies adopted in this work are an example of this integrative approach. Gene-expression signatures of compounds identified by drug screening were combined with the transcriptional profile of the disease of interest. Our disease model was nephropathic cystinosis, a rare genetic disorder characterized by cystine accumulation [36], tendency of cells to undergo apoptosis [37,38], mitochondrial impairment [39,40], and defective autophagy [41,42], among others. The current therapy for cystinosis is limited to cysteamine. This drug reacts with cystine to form a lysine-like mixed disulfide that exits lysosomes through the PQLC2 cationic amino acid transporter [43]. Despite its effectiveness in lowering cystine, cysteamine does not prevent renal tubular damage and can only delay progression to end-stage kidney disease, without preventing it [44]. Different cell pathways are likely to play an important role in the pathophysiology of the disease [5]. In addition, cysteamine has side effects that limit compliance of patients, particularly during adolescence [45].

For these reasons, the cystinosis community needs new therapeutic alternatives. In this work, we used drug repurposing approaches and transcriptional analyses to identify new potential therapies. Candidate drugs were directly found through HCS/HTS, and indirectly through the characterization of cell pathways that are modified by candidate drugs and by the disease itself.

Sirota et al. proposed in 2011 that drugs reversing changes in the transcriptional profile of a given disease, may correct the disease phenotype, irrespectively of the biological targets that they act on [46]. We therefore performed two drug screenings using a small molecule library characterized by high chemical and pharmacological diversity to find compounds that firstly reduced cell cystine content and that also protected cystinotic cells from apoptosis. Since these molecules are already used in human medicine, they can rapidly be tested in preclinical studies, and if their efficacy is confirmed, they can be proposed for clinical studies.

We identified six compounds by crossing data of two screenings and confirmed five, namely alexidine dihydrochloride (phosphatidylglycerophosphatase inhibitor), beta-escin (nitric oxide synthase stimulant), digoxin (ATPase inhibitor), disulfiram (aldehyde dehydrogenase inhibitor), and fluspirilene (dopamine receptor antagonist).

Alexidine dihydrochloride was shown to have the most potent lysosomal cholesterol-reducing activity in a high-content screening for modifiers of Niemann-Pick type C disease [47] and it was also a hit of the previous cell-based phenotypic drug screening for compounds that reduced the autophagy-related protein p62/SQSTM1 levels in cystinotic cells [42]. Beta-escin has an antioxidant potential [48] and induces perturbation in cholesterol homeostasis, which causes a cascade of cellular responses like decreased NFκB activation [14]. Similarly, disulfiram, an old anti-alcoholism drug, has anti-inflammatory properties and inhibits NFκB [49,50] and has a direct effect on autophagy [51]. These effects could be very useful in management of cystinosis, which shows inflammasome activation and high ROS production [52,53]. Digoxin is one of the oldest cardiovascular medications used today to manage heart failure, it reversibly inhibits the Na-K ATPase, which has been shown to play a critical role in cell adhesion [54], and it may block autosis [55].

Furthermore, as demonstrated by Wang et al. 2017, alexidine dihydrochloride and digoxin showed therapeutic potential in other metabolic disorders and in ageing by activating TFEB [17]. This aspect could explain the best efficacy in cystine depletion showed in dose–response curve by these two drugs in our model. Finally, fluspirilene, a potent antipsychotic drug used for the treatment of schizophrenia, was demonstrated to induce autophagy, and promote long-lived protein degradation [56], processes compatible with the rescue of cystinotic phenotype [57]

Similarly, abnormal tight junction signaling in primary tubular epithelial cells obtained from *CTNS*^−/−^ mice and the development of swan-neck deformities in proximal tubules of these same animals, support the concept that cell adhesion processes may represent a valuable target to improve the Fanconi syndrome in cystinosis [41,58].

These first findings were “amplified” in silico with the additional identification of drugs with similar transcriptional activities (MANTRA 2.0 software) [24,25], illustrating the power of this type of approach. In this way, niclosamide, which was initially discarded because it decreased cystine content but had little effects on apoptosis, was brought back into consideration. When combined with ethanolamine (NEN—niclosamide ethanolamine), the solubility, absorption, and systemic bioavailability of niclosamide in vivo was considerably improved [59]. Niclosamide can ameliorate renal damage by regulating mitochondrial redox balance [60] and autophagy processes, which decreases the level of inflammatory cytokines [61]. It also mitigates renal fibrosis by inhibiting *HIPK2* expression in the tubulointerstitial compartment [62].

As previously reported, current therapy based on cysteamine treatment, although significantly reduces cystine content in tissues, does not stop the evolution of the disease. Therefore, we accompanied computational/theoretical approaches to the experimental approach in order to find additional biological targets for drugs that could ameliorate the phenotype and the disease progress [34]. First, we performed RNA-seq analysis on ciPTECs, albeit a limitation of this study is that the identification of differentially expressed genes of ciPTECs does not distinguish uniquely between causal and correlative relationships because these cell lines were not isogenic. However, the analysis showed results coherent with previous studies (i.e., cAMP signaling and cell-adhesion pathways [39,63] or identified new potential targets, such as taurine/hypotaurine metabolism, which are involved in cystine metabolism [64] or netrin-1, a laminin-like protein with an important role in cell adhesion and tissue morphogenesis [65,66].

Post hoc analysis, accomplished with the help of free online tools, showed several altered biological processes that could be corrected by the selected compounds. Involvement of mitoribosomes, essential for synthesizing mitochondrial membrane proteins, is a new aspect and further investigations will be necessary to find the functional significance of this association. Immune cell adhesion is a critical step in the inflammatory response and this fundamental biological process, confirmed by our analysis, is already being studied with the aim of finding a treatment for inflammation associated with renal disease in cystinosis [67,68,69]. Receptors of tyrosine kinases (RTK) and downstream signal transduction pathways could be also good candidates since they were shown to have a potential therapeutic action in renal diseases [70]. Signaling pathway of TGF-β and/or its receptors may not be an ideal target due to their variety and complexity action in renal fibrosis and inflammation with effects sometimes opposing [71]. Finally, regulation of cellular response to stress represented a key process with a central role in the pathogenesis and in therapeutic action.

## 4. Conclusions

These combined approaches firstly have allowed identifying five candidate drugs that are currently being studied in our laboratory using mouse and rat models of cystinosis, a *conditio sine qua non* to define their therapeutic use in humans. Secondly, we delineated by computational analysis the biological processes in cystinotic proximal tubular cells that could be considered for in vitro pre-clinical studies.

## 5. Materials and Methods

### 5.1. Cell Culture

Conditionally immortalized proximal tubular epithelial cells (ciPTECs), from healthy donors and patient with cystinosis bearing the classical homozygous 57-kb deletion (*CTNS*^−/−^ ciPTECs), were obtained from Radboud University Medical Center, Nijmegen, The Netherlands, and cultured as described in Wilmer et al. [72] in a humidified atmosphere with 5% CO_2_ at 37 °C.

### 5.2. Screening of the Prestwick Chemical Library in CTNS^−/−^ ciPETCs

The Prestwick Chemical Library was purchased from Prestwick Chemical (Illkirch-Graffenstaden, France) and consisted of a collection of 1200 off-patent small molecules, most of them approved for human use by US Food and Drug Administration (FDA), European Medicines Agency (EMA) and other regulatory agencies.

For cystine determination, *CTNS*^−/−^ ciPETCs were seeded in 48-well plates at 5 × 10^4^ cells/well and, after 48 h, 10 µM of each library compound was added to individual wells for 24 h using the epMotion 5075 automated pipetting system. We included *CTNS*^−/−^ ciPETCs treated with cysteamine 100 µM as a positive control and cells treated with DMSO 0.1% *v*/*v*, the vehicle in which compounds are dissolved, as a negative control. Cells were washed twice with PBS, treated with 75 µL of 10 mM N-ethylmaleimide, and lysed with four cycles of freezing and thawing. Proteins were precipitated by adding 75 µL of 10% sulfosalicylic acid to the cell lysates and incubating overnight at 4 °C. Plates were then centrifuged at 3000× *g* for 15 min at 4 °C and 25 µL of supernatant from each well were transferred in derivatization tubes. Samples were then processed and analyzed for cystine content by reverse-phase high-performance liquid chromatography as described by Pastore et al. [73]. Protein precipitated in each well was dissolved in 150 µL of 0.1 M NaOH and BCA protein assay was performed in accordance with the manufacturer’s protocol (Bio Rad Laboratories, Hercules, CA, USA).

For apoptosis determination, we developed a quantitative apoptosis assay based on caspases 3/7 positivity of cells exposed to pro-apoptosis stimuli, namely a combination of Fas-ligand and cycloheximide (Supplementary Figure S2A). *CTNS*^−/−^ ciPETCs were seeded at 5 × 10^3^ cells/well in 384-well poly-D-lysine coated plates (Perkin Elmer, Waltham, MA, USA). After 48 h, cells were pre-incubated with 10 µM of library compounds for one hour and treated with Fas ligand (0.5 µg/mL) and cycloheximide (10 µg/mL) for 5 h to induce apoptosis. After treatment, ciPTECs were incubated with 4 µM of CellEvent Caspase-3/7 Green Detection Reagent (Invitrogen life technologies, Carlsbad, CA, USA) for 30 min. Cells were then fixed with 4% paraformaldehyde and nuclei were marked with Hoechst 33258. Cells were imaged with the automated Opera Phenix™ High Content Screening System (Perkin Elmer, Beaconsfield, UK) and apoptotic cells were quantified as CellEvent positive nuclei/total cells. Manipulations were automated by using the Hamilton Liquid handler (Hamilton Company, Bonaduz, Switzerland). The performance of the screening was evaluated with Z-factor calculated on 32 positive and 32 negative controls for each 384 well plate. Z-factor of the assay was 0.55 (Supplementary Figure S2B), a value that is considered optimal for HCS assay [74]. Quality control was performed by calculating the Z-factor of each plate (Supplementary Figure S2C).

### 5.3. RNA Extraction, Library Construction, and Sequencing

Total RNA extraction from wild-type and *CTNS*^−/−^ ciPTECs, library construction, sequencing service, and transcriptome analyses were performed at BGI Genomics.

Integrity of total RNA extracted (RNA concentration, RIN value, 28S/18S and the fragment length distribution) was assessed by Agilent 4200 Bioanalyzer system.

Library was generated using MGIEasy RNA Library Prep Set. Workflow of library construction and sequencing involved purifying the poly-A containing mRNA molecules, using poly-T oligo-attached magnetic beads. Following purification, the mRNA was fragmented into small pieces using divalent cations under elevated temperature. The cleaved RNA fragments were copied into first strand cDNA using reverse transcriptase and random primers. Then it was performed the second strand cDNA synthesis using DNA Polymerase I and RNase H. These cDNA fragments then had the addition of a single ‘A’ base and subsequent ligation of the adapter. The products were then purified and enriched with PCR amplification. PCR product was quantified by Qubit and pooled samples together to make a single strand DNA circle (ssDNA circle), which gave the final library. DNA nanoballs (DNBs) were generated with the ssDNA circle by rolling circle replication (RCR) to enlarge the fluorescent signals at the sequencing process. The DNBs were loaded into the patterned nanoarrays, and pair-end reads of 100 bp were read through on the DNBSEQ-G400 for the following data analysis study. For this step, the DNBseq platform combined the DNA nanoball-based nanoarrays and stepwise sequencing using the combinational probe-anchor synthesis sequencing method.

SOAPnuke 1.5.2 software (https://github.com/BGI-flexlab/SOAPnuke, accessed on 18 October 2021) was used to filter reads by removing reads with adaptors, reads in which unknown bases (N) are more than 10%, and low-quality reads.

Clean reads were then mapped by using HISAT2 2.0.4 (Hierarchical Indexing for Spliced Alignment of Transcripts) using the following parameters: --phred33 --sensitive --no-discordant --no-mixed -I 1-X 1000.

StringTie [75] was used to reconstruct transcripts (parameters: -f 0.3 -j 3 -c 5 -g 100 -s 10000 -p 8), and Cufflinks (parameters: -p 12) tools [76] were used to compare reconstructed transcripts to reference annotation.

For gene expression analysis, clean reads were mapped to the human GRCh38/hg38 build reference genome using Bowtie2 v2.2.5 (http://bowtie-bio.sourceforge.net/Bowtie2/index.shtml, accessed on 18 October 2021) using the following parameters: -q --phred33 --sensitive --dpad 0 --gbar 99999999 --mp 1,1 --np 1 --score-min L,0,-0.1 -I 1 -X1000 --no-mixed --no-discordant -p 1 -k 200 [77], and then we calculated gene expression level with RSEM v1.2.12 (http://deweylab.biostat.wisc.edu/RSEM, accessed on 18 October 2021) using default settings [78].

Based on the gene expression level, DEGs between experimental groups were identified by using DESeq2 algorithms, a software based on the negative binomial distribution, performed as described in Michael I et al. [20] with following parameters: fold change ≥ 2.00 and adjusted *p*-value ≤ 0.05.

Biological function analysis of the DEGs was enriched by Gene Ontology (GO) and Kyoto Encyclopedia of Genes and Genomes (KEGG) pathway.

RNA-Seq data accompanying this paper are available through Gene Expression Omnibus (GEO) repository, under accession number GSE184805.

### 5.4. Statistical Analyses

To monitor the performance of the screenings, we used the Z-factor statistical parameter [11]. Z-factor for HTS was evaluated by using the formula Z-factor = $1-\frac{3\left(\sigma_{p}+\sigma_{n}\right)}{\left|\mu_{p}-\mu_{n}\right|}$ where σ_p_ and σ_n_ are the SDs of the positive or negative sample, and µ_p_ and µ_n_ represent the averages. Z-factor for HCS was evaluated by using the same formula of above divided by square root of replicate number. When only two groups were compared, unpaired two-tailed *t*-tests were used for numerical data. Differences between groups were compared by one-way ANOVA and, if significant, pairwise comparisons were evaluated by the Bonferroni multiple comparisons test. All differences were considered statistically significant with *p*-value < 0.05. GraphPad Prism 8 software was used for all statistical analyses.

## Data Availability

RNA-Seq data accompanying this paper are available through Gene Expression Omnibus (GEO) repository, under accession number GSE184805.

## Supplementary Materials

Supplementary materials can be found at https://www.mdpi.com/article/10.3390/ijms222312829/s1.
