# Supplementary material for: Drug Repurposing in Rare Diseases: An Integrative Study of Drug Screening and Transcriptomic Analysis in Nephropathic Cystinosis
Source: Int J Mol Sci. 2021 Nov 27;22(23):12829. doi: 10.3390/ijms222312829 (PMC8657658; doi:10.3390/ijms222312829)
Supplement: Supplementary file 1 [file ijms-22-12829-s001.zip › Supplementary Table S1.pdf]

No.	Chemical name	Structure	CAS number	Therapeutic group	Mechanism of action
**#1**	Alexidine dihydrochloride	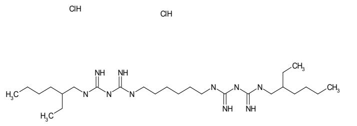	22573-93-9	Antibacterial	Detergent
**#2**	Amphotericin B	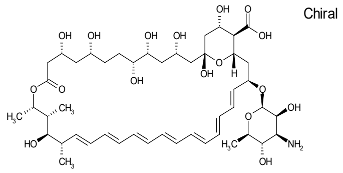	1397-89-3	Antibacterial	Ergosterol ligand
**#3**	Apramycin	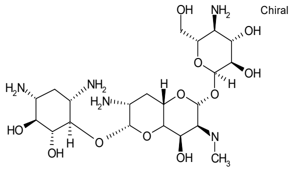	37321-09-8	Antibacterial	
**#4**	Astemizole	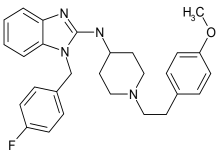	68844-77-9	Antihistaminic	H1 antagonist
**#5**	Auranofin	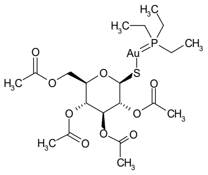	34031-32-8	Antirheumatic	
**#6**	Beta-Escin	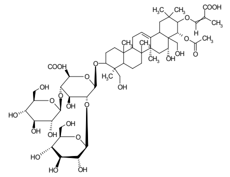	11072-93-8	Peripheral vascular disorders	
**#7**	Chrysene-1,4-quinone	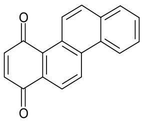	100900-16-1	-	-
**#8**	Cycloheximide	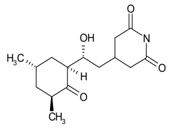	66-81-9	Antibacterial	-
**#9**	Digoxin	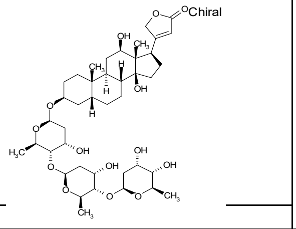	20830-75-5	Cardiotonic	Na^+ K^+ ATPase inhibitor
**#10**	Disulfiram	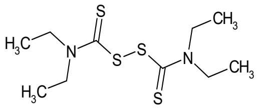	97-77-8	Alcohol deterrent	Dopamine beta-hydroxylase inhibitor

**#11**	Dolasetron mesilate	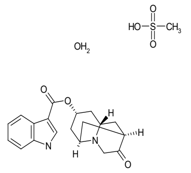	115956-13-3	Nausea in chemotherapy	5-HT3 antagonist
**#12**	Felbinac	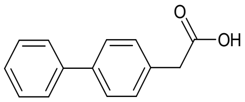	5728-52-9	Anti-inflammatory	Cyclooxygenase Inhibitor
**#13**	Fluspirilen	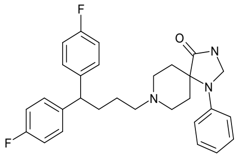	1841-19-6	Antipsychotic	
**#14**	Niclosamide	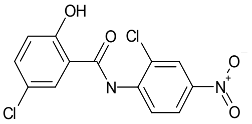	50-65-7	Anthelmintic	Helmintic DNA damage
**#15**	Perhexiline maleate	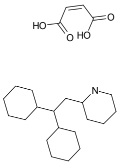	6724-53-4	Vasodilatator	Ca^{2+} blocking agent
**#16**	Prazosin hydrochloride	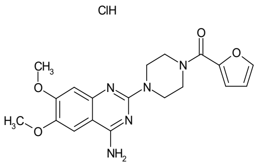	19237-84-4	Anti-hypertensive	Adrenergic receptor antagonist
**#17**	Quinacrine dihydrochloride dihydrate	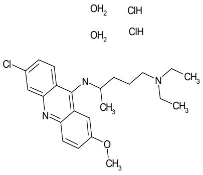	6151-30-0	Antimalarial	Monoamine oxydase inhibitor
**#18**	Suloctidil	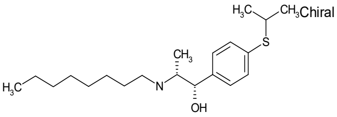	54063-56-8	Vasodilatator	-
**#19**	Terfenadine	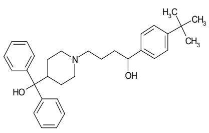	50679-08-8	Antihistaminic	H1 antagonist
**#20**	Thiethylperazine dimalate	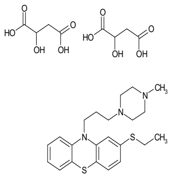	52239-63-1	Antiemetic	Adrenergic antagonist
**#21**	Thimerosal	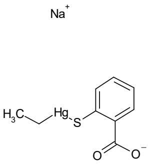	54-64-8	Antiseptic	

**#22**	Thioridazine hydrochloride	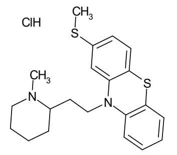	130-61-0	Neuroleptic	Ca^{2+} channel antagonist
**#23**	Thonzonium bromide	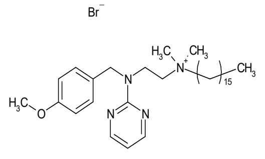	553-08-2	Antiseptic	Detergent
**#24**	Tocainide hydrochloride	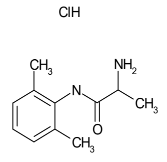	71395-14-7	Anti-arrhythmic	Na^{+} channel inhibitor
