# Supplementary material for: Drug Repurposing in Rare Diseases: An Integrative Study of Drug Screening and Transcriptomic Analysis in Nephropathic Cystinosis
Source: Int J Mol Sci. 2021 Nov 27;22(23):12829. doi: 10.3390/ijms222312829 (PMC8657658; doi:10.3390/ijms222312829)
Supplement: Supplementary file 1 [file ijms-22-12829-s001.zip › Supplementary Table S2.pdf]

No.	Chemical name	Structure	CAS number	Therapeutic group	Mechanism of action
#1	(+,-)-Synephrine	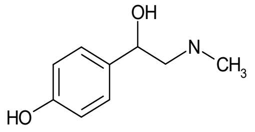	94-07-5	Vasoconstrictor	Adrenergic receptor agonist
#2	Alexidine dihydrochloride	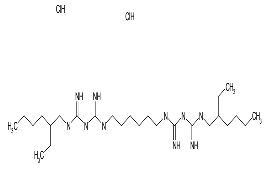	22573-93-9	Antibacterial	Detergent
#3	Auranofin	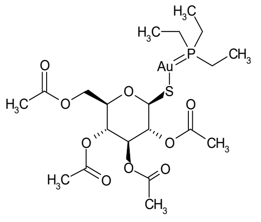	34031-32-8	Antirheumatic	
#4	Benfotiamine	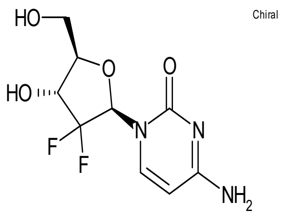	22457-89-2	Vitamin	-
#5	Beta-Escin	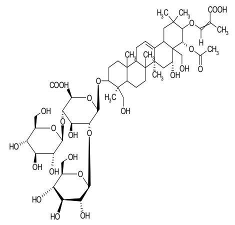	11072-93-8	Peripheral vascular disorders	
#6	Bromopride	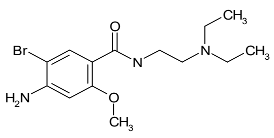	4093-35-0	Antiemetic	Dopamine antagonist
#7	Ciclopirox ethanolamine	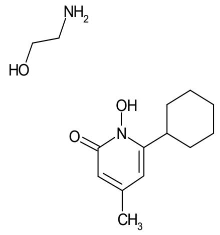	41621-49-2	Antifungal	Cell membrane proteins synthesis inhibitor

**#8**	Clodronate	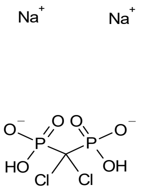	22560-50-5	Bone resorption inhibitor	Bisphosphonate
**#9**	Daunorubicin hydrochloride	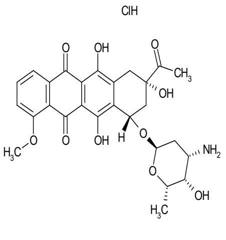	23541-50-6	Antineoplastic	DNA intercaling
**#10**	Digitoxigenin	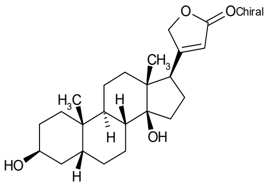	143-62-4	Cardiotonic	Na+ K+ ATPase inhibitor
**11**	Digoxin	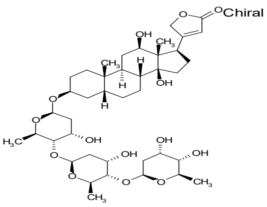	20830-75-5	Cardiotonic	Na+ K+ ATPase inhibitor
**#12**	Disulfiram	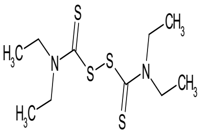	97-77-8	Alcohol deterrent	Dopamine beta-hydroxylase inhibitor
**#13**	Doxorubicin hydrochloride	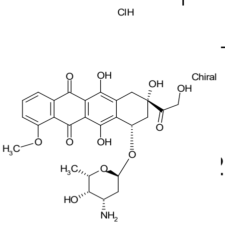	[illegible]5316-40-9	Antibacterial	DNA intercalant
**#14**	Fluspirilen	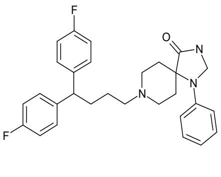	1841-19-6	Antipsychotic	
**#15**	Formoterol fumarate	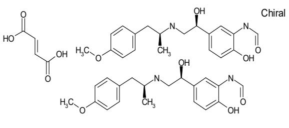	43229-80-7	Antiasthmatic	beta2-agonist

**#16**	Gemcitabine		95058-81-4	Antineoplastic	
**#17**	Glimepiride	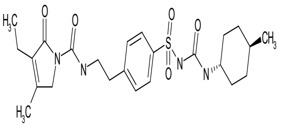	93479-97-1	Antidiabetic	
**#18**	Hexachlorophene	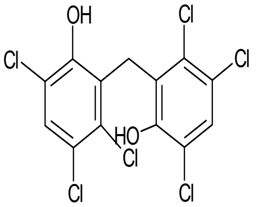	70-30-4	Antiseptic	
**#19**	Lanatoside C	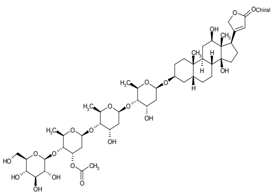	17575-22-3	Cardiotonic	Na+ K+ ATPase inhibitor
**#20**	Meclofenoxate hydrochloride	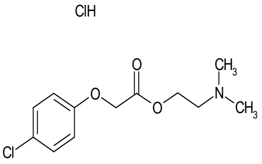	3685-84-5	Cerebral stimulant	Acetylcholine precursor
**#21**	Mitoxantrone dihydrochloride	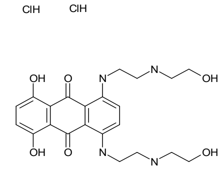	70476-82-3	Antineoplastic	DNA topoisomerase II inhibitor
**#22**	Nalidixic acid sodium salt	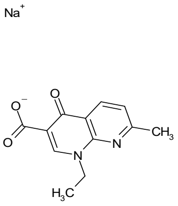	3374-05-8	Antibiotic	Subunit of DNA gyrase inhibitor
**#23**	Parthenolide	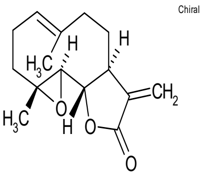	20554-84-1	Antiinflammatory	MAP kinase inhibitor

**#24**	Sulfapyridine	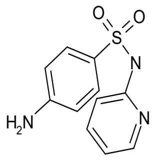	144-83-2	Antibacterial	
**#25**	Thimerosal	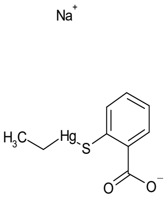	54-64-8	Antiseptic	
**#26**	Torsemide	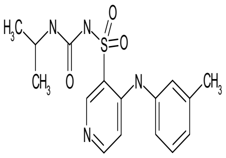	56211-40-6	Antihypertensive	Na-K-Cl symporter inhibitor
**#27**	Verteporfin	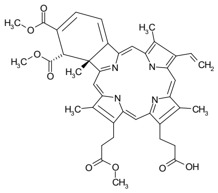	129497-78-5	Treatment of age-related macular degeneration	
