# Supplementary material for: Drug Repurposing in Rare Diseases: An Integrative Study of Drug Screening and Transcriptomic Analysis in Nephropathic Cystinosis
Source: Int J Mol Sci. 2021 Nov 27;22(23):12829. doi: 10.3390/ijms222312829 (PMC8657658; doi:10.3390/ijms222312829)
Supplement: Supplementary file 1 [file ijms-22-12829-s001.zip › Supplementary Table S3.pdf]

GeneID	Length	Symbol	log2FoldChange (*CTNS*$^{-/-}$/*CTNS*$^{+/+}$)	P_{value}	P_{adj}
8653	4476	DDX3Y	8.66	1.69E-99	1.33E-97
8287	9284	USP9Y	8.30	1.04E-114	1.05E-112
2028	3400	ENPEP	7.86	4.78E-247	1.89E-244
590	2449	BCHE	7.59	9.06E-76	4.52E-74
7404	6186	UTY	7.42	1.10E-60	3.92E-59
9086	1411	EIF1AY	7.37	1.16E-59	4.05E-58
7544	5312	ZFY	7.33	6.46E-64	2.50E-62
55655	3590	NLRP2	7.29	5.33E-63	2.02E-61
8209	1623	C21orf33	7.28	3.81E-82	2.14E-80
4237	1124	MFAP2	7.17	6.02E-147	9.64E-145
5947	913	RBP1	7.11	2.40E-87	1.51E-85
151258	2158	SLC38A11	6.88	9.51E-50	2.55E-48
22829	4262	NLGN4Y	6.68	5.92E-46	1.42E-44
1.01E+08	702	KRBOX1	6.63	4.33E-57	1.42E-55
846	5270	CASR	6.54	2.22E-43	4.95E-42
26762	2044	HAVCR1	6.53	0.00E+00	0.00E+00
9476	1605	NAPSA	6.44	6.17E-42	1.31E-40
5672	2113	PSG4	6.34	1.70E-54	5.23E-53
3547	2846	IGSF1	6.33	5.89E-40	1.17E-38
1E+08	5427	C4B_2	6.24	7.35E-38	1.37E-36
10568	4165	SLC34A2	6.23	0.00E+00	0.00E+00
6678	3603	SPARC	6.15	0.00E+00	0.00E+00
84557	1047	MAP1LC3A	6.10	1.82E-65	7.28E-64
204962	4825	SLC44A5	6.10	1.39E-172	2.77E-170
9971	1877	NR1H4	6.06	1.87E-48	4.76E-47
9365	4633	KL	6.05	5.19E-58	1.73E-56
154664	16575	ABCA13	6.04	2.91E-51	8.04E-50
9547	1989	CXCL14	6.03	0.00E+00	0.00E+00
54627	5455	MAP10	5.99	1.24E-34	2.06E-33
2242	2714	FES	5.95	2.23E-49	5.88E-48
84898	11392	PLXDC2	5.77	3.53E-48	8.85E-47
165	4095	AEBP1	5.76	7.78E-56	2.46E-54
55258	2226	THNSL2	5.64	6.10E-43	1.34E-41
6568	2009	SLC17A1	5.64	2.27E-40	4.58E-39
85453	4478	TSPYL5	5.62	2.69E-129	3.46E-127
7373	6792	COL14A1	5.60	5.78E-74	2.77E-72
1577	1617	CYP3A5	5.52	5.90E-43	1.30E-41
26539	1127	OR10H1	5.51	6.98E-28	9.06E-27
138065	2971	RNF183	5.49	6.80E-58	2.26E-56
81031	4232	SLC2A10	5.45	3.14E-71	1.42E-69
26287	1520	ANKRD2	5.36	2.07E-35	3.53E-34
83690	4286	CRISPLD1	5.36	1.01E-129	1.33E-127

56477	2175	CCL28	5.31	1.95E-73	9.18E-72
79781	3185	IQCA1	5.26	2.86E-102	2.37E-100
5174	2100	PDZK1	5.26	0.00E+00	0.00E+00
6913	3463	TBX15	5.23	5.02E-199	1.39E-196
10417	1825	SPON2	5.21	1.99E-28	2.64E-27
2348	1140	FOLR1	5.19	0.00E+00	0.00E+00
8808	2712	IL1RL2	5.17	6.90E-28	8.96E-27
10537	1006	UBD	5.16	1.87E-70	8.26E-69
9087	1702	TMSB4Y	5.16	1.30E-23	1.40E-22
56142	5430	PCDHA6	5.16	6.05E-32	9.19E-31
137970	8985	UNC5D	5.14	5.98E-36	1.04E-34
114805	5683	GALNT13	5.13	2.46E-27	3.11E-26
51365	1801	PLA1A	5.03	3.47E-140	5.31E-138
10786	1649	SLC17A3	5.01	1.41E-33	2.27E-32
143686	9443	SESN3	5.00	1.02E-209	3.06E-207
216	2378	ALDH1A1	4.97	5.61E-224	1.85E-221
124976	4349	SPNS2	4.95	0.00E+00	0.00E+00
1285	7277	COL4A3	4.95	6.84E-169	1.34E-166
6750	665	SST	4.94	3.08E-21	2.95E-20
222611	5135	ADGRF2	4.94	6.76E-23	6.99E-22
140886	3447	PABPC5	4.93	1.51E-26	1.85E-25
1396	480	CRIP1	4.89	3.93E-38	7.43E-37
54328	4269	GPR173	4.89	1.69E-73	7.99E-72
202374	4059	STK32A	4.88	1.47E-20	1.37E-19
5625	2008	PRODH	4.86	3.24E-20	2.96E-19
4094	6143	MAF	4.84	1.57E-69	6.79E-68
6328	8756	SCN3A	4.83	2.03E-52	5.86E-51
57556	6643	SEMA6A	4.79	5.40E-106	4.80E-104
57586	5184	SYT13	4.78	4.12E-251	1.70E-248
284217	8782	LAMA1	4.78	0.00E+00	0.00E+00
56521	1111	DNAJC12	4.75	8.47E-43	1.86E-41
26471	887	NUPR1	4.74	5.32E-211	1.66E-208
6539	3234	SLC6A12	4.74	1.22E-37	2.26E-36
1.03E+08	2544	CBSL	4.73	0.00E+00	0.00E+00
6519	2350	SLC3A1	4.72	2.66E-29	3.67E-28
3115	4055	HLA-DPB1	4.72	1.75E-71	7.94E-70
1E+08	2671	ZNF730	4.71	2.43E-20	2.23E-19
2263	3977	FGFR2	4.69	1.61E-198	4.41E-196
728689	2090	EIF3CL	4.66	5.89E-131	7.99E-129
389840	4419	MAP3K15	4.65	7.24E-29	9.80E-28
10690	12894	FUT9	4.63	5.03E-81	2.77E-79
341640	16179	FREM2	4.62	1.19E-50	3.25E-49
56144	5365	PCDHA4	4.61	9.61E-18	7.79E-17
4057	2648	LTF	4.60	3.48E-71	1.57E-69
3936	3708	LCP1	4.60	1.27E-181	2.75E-179
710	1844	SERPING1	4.58	8.56E-237	3.17E-234
81615	1906	TMEM163	4.57	8.86E-127	1.05E-124
1580	1982	CYP4B1	4.56	3.70E-42	7.98E-41

4881	4196	NPR1	4.56	1.40E-18	1.18E-17
167410	3979	LIX1	4.55	7.21E-32	1.09E-30
153218	509	SPINK13	4.54	9.02E-42	1.91E-40
151651	1984	EFHB	4.52	6.16E-39	1.19E-37
91156	11711	IGFN1	4.50	3.04E-271	1.46E-268
8706	3356	B3GALNT1	4.50	1.13E-70	4.99E-69
123	2026	PLIN2	4.49	1.52E-235	5.50E-233
388630	5146	TRABD2B	4.48	8.06E-228	2.81E-225
340578	2612	DCAF12L2	4.48	9.98E-21	9.36E-20
8631	1378	SKAP1	4.46	1.40E-36	2.50E-35
56098	4623	PCDHGC4	4.46	8.34E-53	2.43E-51
4246	521	SCGB2A1	4.46	7.43E-18	6.07E-17
1.03E+08	450	LINC01835	4.45	7.57E-18	6.17E-17
6540	1758	SLC6A13	4.42	1.01E-32	1.56E-31
51477	2192	ISYNA1	4.39	0.00E+00	0.00E+00
26167	2926	PCDHB5	4.39	3.62E-36	6.34E-35
139221	4143	MUM1L1	4.38	9.75E-24	1.05E-22
203859	6658	ANO5	4.38	5.65E-25	6.43E-24
1.01E+08	2281	TBC1D3D	4.37	1.04E-90	6.84E-89
7620	2100	ZNF69	4.34	1.89E-46	4.59E-45
55466	3011	DNAJA4	4.33	3.40E-47	8.42E-46
6696	1635	SPP1	4.31	0.00E+00	0.00E+00
1.02E+08	2247	LOC101929839	4.31	8.15E-98	6.16E-96
5502	1689	PPP1R1A	4.31	0.00E+00	0.00E+00
122402	4245	TDRD9	4.30	5.43E-19	4.67E-18
1.08E+08	2247	LOC107987205	4.30	1.15E-74	5.59E-73
729085	3449	FAM198A	4.28	3.32E-16	2.46E-15
729877	2048	TBC1D3H	4.27	8.23E-15	5.64E-14
26157	1523	GIMAP2	4.26	7.22E-21	6.81E-20
2615	4130	LRRC32	4.24	5.18E-105	4.54E-103
25999	3391	CLIP3	4.22	8.47E-131	1.14E-128
1644	2058	DDC	4.22	2.65E-28	3.49E-27
151354	6366	FAM84A	4.20	5.63E-39	1.09E-37
222643	3128	UNC5CL	4.19	1.56E-79	8.43E-78
8756	3397	ADAM7	4.18	1.93E-23	2.05E-22
9022	815	CLIC3	4.16	8.22E-47	2.02E-45
91543	3607	RSAD2	4.16	9.93E-29	1.33E-27
5063	6571	PAK3	4.16	2.00E-37	3.68E-36
680	1413	BRS3	4.14	4.95E-15	3.43E-14
153020	2918	RASGEF1B	4.12	1.02E-43	2.31E-42
79836	7765	LONRF3	4.12	1.63E-38	3.11E-37
115701	5720	ALPK2	4.08	0.00E+00	0.00E+00
5669	2050	PSG1	4.07	6.40E-37	1.15E-35
827	3604	CAPN6	4.06	1.45E-55	4.56E-54
7367	2099	UGT2B17	4.06	1.55E-17	1.25E-16
342527	2019	SMTNL2	4.06	7.36E-59	2.50E-57
80059	3464	LRRTM4	4.05	2.82E-13	1.73E-12
4939	3192	OAS2	4.05	3.40E-78	1.76E-76

9938	2140	ARHGAP25	4.04	2.58E-18	2.15E-17
85409	2026	NKD2	4.03	3.72E-16	2.75E-15
79656	1849	BEND5	4.02	2.99E-19	2.62E-18
1.08E+08	2525	LOC107986351	4.02	2.59E-23	2.73E-22
9215	3308	LARGE1	4.01	1.39E-93	9.77E-92
3702	4065	ITK	4.00	8.95E-15	6.11E-14
353345	3685	GPR141	3.99	7.46E-13	4.44E-12
57633	5976	LRRN1	3.97	1.82E-15	1.30E-14
2331	3271	FMOD	3.97	1.85E-48	4.71E-47
7412	3197	VCAM1	3.95	1.39E-191	3.34E-189
10516	2637	FBLN5	3.94	4.19E-54	1.27E-52
83988	3581	NCALD	3.93	3.53E-18	2.92E-17
5314	13813	PKHD1	3.93	1.08E-122	1.22E-120
10912	1049	GADD45G	3.91	3.65E-24	3.99E-23
11262	1359	SP140	3.91	9.54E-32	1.44E-30
63910	2605	SLC17A9	3.91	5.46E-71	2.44E-69
84968	2278	PNMA6A	3.88	4.70E-12	2.65E-11
3764	2396	KCNJ8	3.88	2.35E-33	3.74E-32
26223	1794	FBXL21	3.86	1.56E-14	1.05E-13
5973	1473	RENBP	3.86	3.59E-19	3.12E-18
79170	1491	PRR15L	3.86	9.21E-13	5.46E-12
9245	2240	GCNT3	3.86	5.57E-73	2.60E-71
9201	6637	DCLK1	3.85	1.15E-97	8.64E-96
133688	2789	UGT3A1	3.85	7.84E-12	4.36E-11
2678	2506	GGT1	3.84	0.00E+00	0.00E+00
79589	2851	RNF128	3.84	1.09E-118	1.17E-116
1837	4997	DTNA	3.84	3.73E-60	1.32E-58
140733	4139	MACROD2	3.83	4.67E-98	3.55E-96
91937	2965	TIMD4	3.83	2.42E-24	2.67E-23
27255	3511	CNTN6	3.81	6.49E-84	3.78E-82
3772	3689	KCNJ15	3.80	0.00E+00	0.00E+00
57631	4876	LRCH2	3.79	7.27E-109	6.80E-107
728957	3465	ZNF705D	3.79	4.38E-13	2.64E-12
64344	5374	HIF3A	3.78	2.07E-19	1.83E-18
5919	765	RARRES2	3.78	2.20E-127	2.67E-125
3248	2834	HPGD	3.77	1.83E-108	1.69E-106
6444	6042	SGCD	3.77	1.90E-15	1.35E-14
3748	5075	KCNC3	3.77	9.44E-94	6.68E-92
80704	3927	SLC19A3	3.76	1.89E-15	1.35E-14
3026	3069	HABP2	3.75	1.55E-71	7.04E-70
5455	4525	POU3F3	3.74	8.06E-289	4.34E-286
9075	2932	CLDN2	3.74	1.12E-205	3.31E-203
55228	3679	PNMA8A	3.74	1.77E-58	5.98E-57
9369	7215	NRXN3	3.73	7.00E-92	4.77E-90
570	3377	BAAT	3.71	7.29E-41	1.49E-39
10246	2205	SLC17A2	3.71	6.52E-11	3.43E-10
8284	6697	KDM5D	3.69	2.12E-165	4.01E-163
163351	4853	GBP6	3.69	2.76E-12	1.58E-11

57101	3593	ANO2	3.68	6.63E-20	5.99E-19
440077	3438	ZNF705A	3.67	2.19E-11	1.19E-10
2239	3714	GPC4	3.66	3.24E-234	1.15E-231
3627	1227	CXCL10	3.65	2.88E-11	1.55E-10
249	2487	ALPL	3.64	5.07E-41	1.05E-39
131616	997	TMEM42	3.64	1.35E-22	1.37E-21
84680	2039	ACCS	3.64	2.48E-58	8.37E-57
23302	5766	WSCD1	3.64	1.61E-112	1.60E-110
346689	1514	KLRG2	3.63	5.62E-61	2.03E-59
4316	1153	MMP7	3.61	0.00E+00	0.00E+00
26289	2489	AK5	3.60	3.25E-85	1.96E-83
115908	1261	CTHRC1	3.60	2.31E-252	9.75E-250
1437	800	CSF2	3.60	5.33E-17	4.16E-16
6819	2778	SULT1C2	3.60	6.10E-94	4.33E-92
1268	6589	CNR1	3.60	6.02E-42	1.28E-40
162514	6133	TRPV3	3.59	8.28E-13	4.92E-12
1593	2407	CYP27A1	3.59	2.32E-98	1.77E-96
5909	3309	RAP1GAP	3.59	3.43E-77	1.75E-75
55026	3338	TMEM255A	3.59	3.24E-48	8.17E-47
440044	2371	SLC22A20P	3.58	3.54E-20	3.23E-19
80333	3509	KCNIP4	3.58	1.85E-11	1.00E-10
1.08E+08	5946	LOC107986797	3.57	1.40E-24	1.56E-23
79132	2572	DHX58	3.55	8.10E-52	2.29E-50
162967	6217	ZNF320	3.54	1.97E-46	4.78E-45
1.02E+08	4683	MEI4	3.54	7.07E-10	3.40E-09
1.08E+08	4067	LOC107987345	3.53	7.77E-10	3.73E-09
51314	2327	NME8	3.53	7.54E-10	3.62E-09
196740	6018	VSTM4	3.52	1.60E-60	5.70E-59
220388	2434	CCDC89	3.52	2.61E-12	1.50E-11
93082	1553	NEURL3	3.51	4.79E-16	3.52E-15
23600	3330	AMACR	3.51	0.00E+00	0.00E+00
163404	3046	PLPPR5	3.51	1.01E-13	6.43E-13
23072	6701	HECW1	3.50	4.05E-15	2.82E-14
56141	2370	PCDHA7	3.50	1.15E-09	5.43E-09
200150	3531	PLD5	3.50	5.15E-70	2.25E-68
6192	910	RPS4Y1	3.49	5.99E-09	2.68E-08
79190	2513	IRX6	3.49	4.18E-12	2.37E-11
50507	2499	NOX4	3.49	9.35E-29	1.26E-27
146227	2896	BEAN1	3.49	8.77E-66	3.54E-64
1.08E+08	4669	LOC107984449	3.49	3.63E-10	1.79E-09
283208	2299	P4HA3	3.48	8.83E-75	4.33E-73
170679	913	PSORS1C1	3.47	1.52E-27	1.94E-26
3429	633	IFI27	3.47	3.77E-22	3.77E-21
353189	5086	SLCO4C1	3.45	7.53E-281	3.72E-278
2015	2630	ADGRE1	3.44	6.94E-10	3.34E-09
79887	1951	PLBD1	3.43	1.06E-27	1.37E-26
5521	2098	PPP2R2B	3.43	1.05E-11	5.79E-11
1.05E+08	1098	LOC105372824	3.42	1.54E-15	1.11E-14

128218	1979	TMEM125	3.42	1.22E-11	6.72E-11
10117	5674	ENAM	3.41	3.34E-19	2.91E-18
6414	2164	SELENOP	3.41	1.11E-16	8.51E-16
5121	566	PCP4	3.40	3.98E-09	1.81E-08
113802	1768	HENMT1	3.40	3.30E-10	1.63E-09
920	3049	CD4	3.40	3.74E-17	2.94E-16
91227	1004	GGTLC2	3.40	3.87E-10	1.90E-09
59353	2086	TMEM35A	3.40	4.37E-09	1.98E-08
139065	6161	SLITRK4	3.39	1.69E-74	8.17E-73
122970	1703	ACOT4	3.39	3.75E-26	4.50E-25
388125	1576	C2CD4B	3.39	5.10E-09	2.29E-08
1286	9907	COL4A4	3.37	2.02E-197	5.35E-195
22986	4515	SORCS3	3.36	4.90E-13	2.95E-12
260293	2074	CYP4X1	3.36	2.19E-09	1.01E-08
3694	4558	ITGB6	3.36	6.16E-122	6.80E-120
56911	1795	MAP3K7CL	3.35	1.11E-92	7.70E-91
286676	2827	ILDR1	3.35	8.00E-09	3.54E-08
28	1580	ABO	3.34	6.09E-26	7.23E-25
3131	5341	HLF	3.33	1.12E-89	7.23E-88
5158	2723	PDE6B	3.33	3.48E-29	4.77E-28
200407	2384	CREG2	3.32	1.39E-18	1.18E-17
29114	1220	TAGLN3	3.32	4.41E-37	7.98E-36
1.08E+08	3507	LOC107986352	3.32	3.88E-27	4.87E-26
54843	2609	SYTL2	3.31	0.00E+00	0.00E+00
255743	4443	NPNT	3.30	9.51E-228	3.25E-225
1080	6143	CFTR	3.30	2.42E-51	6.70E-50
29881	4913	NPC1L1	3.29	4.25E-10	2.08E-09
10875	4268	FGL2	3.29	2.06E-12	1.19E-11
53942	6198	CNTN5	3.29	1.61E-08	6.93E-08
55504	3915	TNFRSF19	3.29	6.55E-90	4.23E-88
7216	4298	TRO	3.28	3.76E-84	2.22E-82
84251	8467	SGIP1	3.28	9.72E-73	4.51E-71
27012	6996	KCNV1	3.28	1.92E-13	1.20E-12
2705	1623	GJB1	3.26	7.86E-09	3.48E-08
10964	3515	IFI44L	3.26	4.09E-106	3.65E-104
220416	3136	LRRC63	3.25	5.27E-15	3.65E-14
4688	2186	NCF2	3.25	1.21E-49	3.22E-48
23024	3738	PDZRN3	3.25	7.83E-10	3.75E-09
55195	2533	CCDC198	3.24	1.82E-131	2.49E-129
341359	3286	SYT10	3.24	7.77E-11	4.05E-10
1.08E+08	3332	LOC107987433	3.24	4.61E-17	3.61E-16
27233	2612	SULT1C4	3.24	3.40E-12	1.94E-11
256764	5497	WDR72	3.24	3.71E-55	1.15E-53
79799	2972	UGT2A3	3.24	3.17E-09	1.45E-08
147463	3678	ANKRD29	3.22	2.19E-70	9.59E-69
4645	9520	MYO5B	3.21	4.88E-163	8.76E-161
158866	2277	ZDHHC15	3.20	6.22E-10	3.00E-09
460	7304	ASTN1	3.18	2.17E-08	9.28E-08

23779	1603	ARHGAP8	3.18	3.94E-42	8.46E-41
55084	6036	SOBP	3.18	1.92E-20	1.77E-19
8424	1880	BBOX1	3.16	1.00E-08	4.42E-08
1410	967	CRYAB	3.15	5.39E-288	2.82E-285
83401	1479	ELOVL3	3.15	3.54E-15	2.48E-14
132946	2211	ARL9	3.15	1.12E-09	5.31E-09
54463	3226	RETREG1	3.15	4.74E-186	1.08E-183
29887	2482	SNX10	3.15	1.60E-221	5.07E-219
55086	3829	CXorf57	3.14	1.20E-32	1.87E-31
2196	14621	FAT2	3.14	1.55E-56	5.00E-55
84752	2722	B3GNT9	3.13	4.55E-76	2.29E-74
2596	1747	GAP43	3.13	1.63E-08	7.02E-08
1428	1559	CRYM	3.13	1.96E-12	1.14E-11
486	584	FXYD2	3.12	8.65E-264	3.84E-261
55553	8910	SOX6	3.12	1.40E-41	2.94E-40
1.03E+08	2184	LOC102724788	3.11	4.22E-71	1.89E-69
4920	3264	ROR2	3.10	8.59E-80	4.65E-78
3773	4008	KCNJ16	3.10	2.99E-258	1.30E-255
6649	1546	SOD3	3.10	5.45E-127	6.50E-125
4685	4374	NCAM2	3.10	1.43E-07	5.64E-07
23500	6228	DAAM2	3.10	2.92E-46	7.06E-45
84179	2077	SLC49A3	3.08	1.84E-33	2.94E-32
25924	4851	MYRIP	3.07	1.06E-08	4.66E-08
2938	1377	GSTA1	3.06	2.04E-07	7.92E-07
9098	7897	USP6	3.06	1.33E-91	8.91E-90
27010	2410	TPK1	3.06	1.10E-37	2.03E-36
1608	5701	DGKG	3.05	2.16E-07	8.38E-07
64108	1027	RTP4	3.05	1.22E-41	2.57E-40
25891	3151	PAMR1	3.05	2.91E-18	2.42E-17
91977	3493	MYOZ3	3.04	2.70E-11	1.46E-10
56999	7295	ADAMTS9	3.03	8.95E-111	8.69E-109
140766	4190	ADAMTS14	3.02	2.10E-173	4.24E-171
2702	3198	GJA5	3.02	3.31E-07	1.26E-06
10178	10703	TENM1	3.02	1.65E-07	6.48E-07
5678	2048	PSG9	3.01	2.38E-16	1.78E-15
25797	1719	QPCT	3.01	2.64E-177	5.53E-175
94234	2338	FOXQ1	3.01	7.12E-51	1.95E-49
5345	2297	SERPINF2	3.01	3.16E-69	1.36E-67
30835	4051	CD209	3.01	1.03E-08	4.53E-08
340267	3339	COL28A1	3.00	1.40E-43	3.15E-42
285195	3435	SLC9A9	3.00	5.66E-15	3.91E-14
6425	1883	SFRP5	3.00	4.30E-09	1.95E-08
90665	2144	TBL1Y	2.99	4.08E-07	1.54E-06
9473	2724	THEMIS2	2.99	5.56E-18	4.55E-17
93100	1753	NAPRT	2.98	4.06E-56	1.29E-54
56121	2891	PCDHB15	2.98	1.70E-14	1.14E-13
3113	1788	HLA-DPA1	2.98	4.34E-35	7.36E-34
54033	2452	RBM11	2.97	8.32E-10	3.98E-09

115749	1674	C12orf56	2.97	1.74E-08	7.49E-08
6690	623	SPINK1	2.97	5.14E-07	1.92E-06
5284	4279	PIGR	2.97	2.07E-29	2.87E-28
30820	2031	KCNIP1	2.96	4.09E-129	5.23E-127
79628	26588	SH3TC2	2.96	5.29E-21	5.01E-20
55586	2179	MIOX	2.95	3.97E-10	1.94E-09
56147	3452	PCDHA1	2.95	7.15E-08	2.91E-07
5265	2184	SERPINA1	2.95	3.69E-271	1.73E-268
114	4100	ADCY8	2.94	6.80E-07	2.50E-06
11279	7442	KLF8	2.94	1.01E-46	2.47E-45
1907	1253	EDN2	2.94	3.87E-192	9.54E-190
1307	4382	COL16A1	2.93	1.57E-158	2.73E-156
80183	3575	RUBCNL	2.93	1.76E-07	6.89E-07
56113	3709	PCDHGA2	2.92	7.76E-15	5.32E-14
4345	3377	CD200	2.92	4.11E-97	3.08E-95
56101	4316	PCDHGB5	2.92	8.57E-95	6.16E-93
56111	4496	PCDHGA4	2.92	5.26E-18	4.31E-17
57639	3333	CCDC146	2.91	1.68E-92	1.16E-90
440533	1936	PSG8	2.91	2.37E-07	9.15E-07
126364	2430	LRRC25	2.91	9.21E-08	3.70E-07
117289	3516	TAGAP	2.91	5.04E-07	1.88E-06
23743	2651	BHMT2	2.89	1.45E-30	2.10E-29
56108	4117	PCDHGA7	2.89	6.97E-35	1.17E-33
342125	3303	TMC3	2.88	1.27E-06	4.55E-06
5016	2258	OVGP1	2.88	1.35E-28	1.80E-27
57007	2078	ACKR3	2.88	9.89E-11	5.11E-10
22871	6841	NLGN1	2.87	4.72E-86	2.91E-84
200010	3187	SLC5A9	2.87	2.26E-07	8.75E-07
6402	2442	SELL	2.87	3.34E-07	1.27E-06
1.05E+08	3261	LOC105373310	2.87	1.89E-10	9.54E-10
183	2587	AGT	2.87	3.09E-28	4.06E-27
5168	3111	ENPP2	2.86	1.94E-46	4.71E-45
84460	3314	ZMAT1	2.86	3.20E-32	4.90E-31
2620	2448	GAS2	2.86	5.05E-15	3.50E-14
7038	8340	TG	2.86	4.83E-35	8.19E-34
56145	4455	PCDHA3	2.86	5.12E-07	1.91E-06
158584	1914	FAAH2	2.85	6.05E-16	4.43E-15
201305	1715	SPNS3	2.84	4.64E-15	3.22E-14
343450	5200	KCNT2	2.84	2.70E-54	8.25E-53
728441	2663	GGT2	2.83	1.13E-14	7.63E-14
1004	7291	CDH6	2.83	2.33E-281	1.18E-278
1.03E+08	2220	LOC102724823	2.83	2.14E-06	7.50E-06
84419	850	C15orf48	2.82	1.51E-50	4.09E-49
79605	3373	PGBD5	2.82	3.22E-36	5.66E-35
7103	1088	TSPAN8	2.82	1.26E-06	4.52E-06
125206	2021	SLC5A10	2.81	3.01E-08	1.27E-07
140462	1798	ASB9	2.81	4.37E-25	5.00E-24
26033	4713	ATRNL1	2.81	3.05E-66	1.24E-64

56104	4590	PCDHGB1	2.80	4.31E-07	1.62E-06
56133	2779	PCDHB2	2.80	6.50E-19	5.56E-18
259	1450	AMBP	2.80	3.07E-09	1.41E-08
1795	7650	DOCK3	2.80	9.78E-52	2.75E-50
1E+08	1431	MKRN2OS	2.79	2.11E-10	1.06E-09
170680	1185	PSORS1C2	2.79	9.30E-10	4.43E-09
114800	3371	CCDC85A	2.79	4.05E-07	1.53E-06
5196	878	PF4	2.79	2.97E-06	1.02E-05
126969	2169	SLC44A3	2.78	2.48E-61	9.10E-60
54988	1813	ACSM5	2.77	5.71E-11	3.01E-10
3172	3852	HNF4A	2.77	4.37E-08	1.81E-07
3382	2083	ICA1	2.76	3.23E-53	9.54E-52
130733	1601	TMEM178A	2.76	1.64E-87	1.04E-85
3037	3275	HAS2	2.75	2.51E-06	8.70E-06
159989	1738	DEUP1	2.74	2.76E-06	9.53E-06
51473	4709	DCDC2	2.73	1.57E-249	6.35E-247
1.08E+08	955	NA	2.73	1.10E-09	5.24E-09
5592	6219	PRKG1	2.73	7.45E-08	3.02E-07
22996	2219	TTC39A	2.73	2.25E-118	2.40E-116
9508	5842	ADAMTS3	2.72	6.66E-167	1.27E-164
8989	5582	TRPA1	2.72	1.68E-06	5.94E-06
79849	2500	PDZD3	2.72	8.79E-07	3.19E-06
8796	3160	SCEL	2.72	2.93E-17	2.32E-16
90527	2127	DUOXA1	2.72	9.45E-07	3.42E-06
81491	5973	GPR63	2.72	6.56E-16	4.79E-15
10218	2307	ANGPTL7	2.72	1.79E-06	6.32E-06
3730	5733	ANOS1	2.72	6.33E-36	1.10E-34
4257	945	MGST1	2.71	1.97E-194	4.93E-192
3359	2260	HTR3A	2.71	1.00E-06	3.63E-06
1.01E+08	2622	APELA	2.71	1.04E-06	3.76E-06
114769	793	CARD16	2.71	5.79E-13	3.46E-12
4312	2081	MMP1	2.70	3.37E-11	1.81E-10
93986	6537	FOXP2	2.70	1.24E-44	2.88E-43
54578	2251	UGT1A6	2.70	3.52E-39	6.87E-38
373863	1681	DND1	2.70	9.58E-24	1.03E-22
6549	5464	SLC9A2	2.70	4.14E-06	1.41E-05
2243	2381	FGA	2.70	7.26E-53	2.12E-51
83716	4617	CRISPLD2	2.69	0.00E+00	0.00E+00
1230	2690	CCR1	2.69	6.05E-07	2.24E-06
3208	1440	HPCA	2.68	1.32E-06	4.71E-06
57209	4148	ZNF248	2.67	2.68E-158	4.58E-156
1573	1927	CYP2J2	2.67	9.84E-17	7.56E-16
166824	6020	RASSF6	2.67	2.88E-57	9.52E-56
719	1985	C3AR1	2.67	2.38E-20	2.18E-19
83543	3453	AIF1L	2.67	0.00E+00	0.00E+00
7903	4535	ST8SIA4	2.66	3.68E-41	7.64E-40
196410	1531	METTL7B	2.66	5.25E-47	1.29E-45
112817	2234	HOGA1	2.66	5.48E-145	8.70E-143

387695	788	C10orf99	2.65	3.01E-19	2.63E-18
285349	5937	ZNF660	2.65	7.84E-08	3.17E-07
10396	8125	ATP8A1	2.64	4.05E-46	9.75E-45
158326	9615	FREM1	2.64	7.12E-06	2.36E-05
33	2664	ACADL	2.63	4.62E-18	3.80E-17
6928	2746	HNF1B	2.63	7.00E-163	1.24E-160
2259	2646	FGF14	2.63	9.17E-15	6.26E-14
5801	2650	PTPRR	2.62	2.26E-07	8.75E-07
51296	2137	SLC15A3	2.61	1.92E-82	1.09E-80
84941	2730	HSH2D	2.61	4.05E-19	3.51E-18
3119	1655	HLA-DQB1	2.61	1.83E-15	1.30E-14
1.02E+08	2859	LOC101928193	2.60	1.41E-05	4.50E-05
57126	2238	CD177	2.60	2.80E-09	1.29E-08
3787	6173	KCNS1	2.60	1.85E-13	1.15E-12
347733	2019	TUBB2B	2.59	1.67E-139	2.49E-137
1.02E+08	2402	LOC101929599	2.59	6.82E-06	2.26E-05
8635	1245	RNASET2	2.59	0.00E+00	0.00E+00
3769	3373	KCNJ13	2.59	4.25E-06	1.44E-05
8644	1250	AKR1C3	2.58	9.14E-105	7.92E-103
10333	6683	TLR6	2.58	2.04E-22	2.07E-21
3111	3485	HLA-DOA	2.58	5.40E-35	9.13E-34
202333	12760	CMYA5	2.58	1.83E-10	9.25E-10
2731	3820	GLDC	2.58	9.35E-75	4.56E-73
2562	5581	GABRB3	2.58	2.31E-13	1.43E-12
57600	7043	FNIP2	2.58	1.54E-245	5.96E-243
10050	3217	SLC17A4	2.58	5.47E-08	2.25E-07
130013	1768	ACMSD	2.57	4.84E-09	2.18E-08
64284	2180	RAB17	2.56	7.82E-37	1.40E-35
1188	2188	CLCNKB	2.56	3.41E-17	2.69E-16
23136	3719	EPB41L3	2.56	2.14E-05	6.66E-05
374	1290	AREG	2.56	2.71E-152	4.54E-150
862	6954	RUNX1T1	2.56	5.24E-06	1.76E-05
91703	1495	ACY3	2.55	1.66E-05	5.25E-05
221416	5238	C6orf223	2.54	2.60E-12	1.49E-11
163115	3142	ZNF781	2.54	5.90E-06	1.97E-05
3934	863	LCN2	2.54	3.38E-241	1.28E-238
440836	970	ODF3B	2.53	2.18E-06	7.62E-06
7594	6787	ZNF43	2.53	3.48E-13	2.12E-12
4147	3771	MATN2	2.53	2.24E-165	4.18E-163
79822	5442	ARHGAP28	2.53	1.19E-18	1.01E-17
11227	5721	GALNT5	2.52	8.73E-15	5.97E-14
1520	4107	CTSS	2.52	3.98E-105	3.50E-103
685	2779	BTC	2.52	1.24E-29	1.72E-28
114088	4888	TRIM9	2.52	1.03E-50	2.81E-49
1551	2099	CYP3A7	2.50	3.17E-05	9.64E-05
27285	1521	TEKT2	2.50	8.66E-06	2.83E-05
55888	3071	ZKSCAN7	2.50	1.66E-11	9.06E-11
25805	1732	BAMBI	2.50	3.16E-23	3.31E-22

6564	3142	SLC15A1	2.50	2.70E-15	1.91E-14
64798	3689	DEPTOR	2.50	1.18E-49	3.14E-48
80763	2331	SPX	2.50	6.34E-39	1.22E-37
5730	837	PTGDS	2.49	3.14E-10	1.55E-09
1.08E+08	6094	LOC107986554	2.49	1.10E-08	4.83E-08
9963	2340	SLC23A1	2.49	1.41E-16	1.07E-15
56977	10294	STOX2	2.49	1.26E-83	7.25E-82
4121	4307	MAN1A1	2.49	2.40E-122	2.68E-120
53630	2446	BCO1	2.48	1.03E-15	7.45E-15
4599	2837	MX1	2.48	3.73E-71	1.68E-69
23498	1598	HAAO	2.48	1.12E-09	5.29E-09
5641	2003	LGMN	2.48	4.26E-267	1.94E-264
2918	3300	GRM8	2.47	3.77E-18	3.12E-17
8842	3970	PROM1	2.47	1.45E-148	2.34E-146
127534	1986	GJB4	2.47	4.37E-07	1.64E-06
1E+08	2917	ERVMER34-1	2.47	4.55E-59	1.56E-57
84034	4009	EMILIN2	2.46	1.48E-18	1.25E-17
3790	2373	KCNS3	2.46	1.98E-74	9.55E-73
256076	7339	COL6A5	2.46	1.35E-05	4.32E-05
81693	1574	AMN	2.46	7.40E-07	2.71E-06
4741	2835	NEFM	2.46	1.31E-29	1.82E-28
4045	9401	LSAMP	2.46	4.90E-14	3.21E-13
57722	6327	IGDCC4	2.46	1.01E-111	9.89E-110
55351	3035	STK32B	2.45	1.27E-149	2.11E-147
7104	1624	TM4SF4	2.45	6.31E-06	2.10E-05
9037	11677	SEMA5A	2.45	1.46E-60	5.21E-59
1.05E+08	1344	LOC105373102	2.45	6.05E-24	6.56E-23
10125	4683	RASGRP1	2.45	4.37E-18	3.60E-17
27237	2918	ARHGEF16	2.45	2.22E-50	5.98E-49
221393	3180	ADGRF4	2.45	6.85E-19	5.87E-18
347404	5910	LANCL3	2.44	9.39E-06	3.06E-05
84944	1845	MAEL	2.44	3.24E-12	1.85E-11
125113	1735	KRT222	2.44	4.50E-18	3.70E-17
84676	1771	TRIM63	2.44	1.16E-06	4.17E-06
29108	780	PYCARD	2.43	2.86E-48	7.23E-47
134285	1255	TMEM171	2.43	1.11E-41	2.33E-40
54769	4386	DIRAS2	2.42	2.44E-18	2.03E-17
4889	2489	NPY5R	2.42	1.69E-16	1.28E-15
1264	1629	CNN1	2.42	3.47E-79	1.85E-77
152816	1884	ODAPH	2.42	5.88E-20	5.33E-19
26507	5988	CNNM1	2.42	4.71E-19	4.06E-18
6809	3490	STX3	2.42	3.92E-163	7.11E-161
256435	2925	ST6GALNAC3	2.41	3.79E-07	1.44E-06
55061	2633	SUSD4	2.41	5.54E-54	1.67E-52
1582	3950	CYP8B1	2.41	7.29E-06	2.41E-05
85477	2920	SCIN	2.41	1.71E-102	1.43E-100
130576	1420	LYPD6B	2.40	5.25E-05	0.000155117
5671	1922	PSG3	2.40	5.38E-08	2.21E-07

5570	1385	PKIB	2.40	1.49E-18	1.26E-17
113220	2192	KIF12	2.40	1.38E-137	2.01E-135
50617	3152	ATP6V0A4	2.40	7.30E-10	3.51E-09
7364	1593	UGT2B7	2.39	5.06E-140	7.68E-138
7113	3135	TMPRSS2	2.39	5.70E-31	8.39E-30
64078	4047	SLC28A3	2.38	4.02E-07	1.52E-06
83953	2659	FCAMR	2.38	4.29E-08	1.78E-07
796	1256	CALCA	2.38	7.91E-05	0.000228142
64926	3277	RASAL3	2.38	1.24E-05	3.98E-05
340273	2396	ABCB5	2.38	1.82E-05	5.72E-05
259307	1809	IL4I1	2.38	5.32E-53	1.56E-51
51127	3011	TRIM17	2.37	3.95E-11	2.11E-10
1139	3763	CHRNA7	2.37	7.22E-20	6.51E-19
653602	2058	NA	2.37	1.12E-05	3.60E-05
22866	5658	CNKSR2	2.37	3.29E-09	1.50E-08
50940	7979	PDE11A	2.37	1.72E-30	2.48E-29
728888	3497	NPIPB11	2.36	4.34E-37	7.85E-36
23426	5369	GRIP1	2.36	2.43E-21	2.34E-20
2330	3598	FMO5	2.36	9.85E-27	1.21E-25
55753	3611	OGDHL	2.36	2.54E-73	1.19E-71
56935	983	SMCO4	2.36	6.62E-48	1.66E-46
284293	816	HMSD	2.36	4.38E-07	1.65E-06
285533	1531	RNF175	2.36	5.96E-28	7.77E-27
6641	4901	SNTB1	2.35	4.59E-114	4.61E-112
1462	6505	VCAN	2.35	2.71E-204	7.91E-202
9720	5327	CCDC144A	2.35	1.60E-21	1.55E-20
79817	6528	MOB3B	2.35	6.51E-17	5.05E-16
2045	5853	EPHA7	2.35	2.61E-16	1.95E-15
23286	4744	WWC1	2.34	3.02E-292	1.68E-289
128414	1547	NKAIN4	2.34	9.62E-21	9.04E-20
6424	2974	SFRP4	2.34	3.12E-06	1.07E-05
366	2744	AQP9	2.34	2.79E-17	2.21E-16
54457	2186	TAF7L	2.34	2.86E-05	8.75E-05
653857	2747	ACTR3C	2.33	8.59E-69	3.68E-67
5920	779	RARRES3	2.33	2.15E-158	3.71E-156
84868	2448	HAVCR2	2.33	8.45E-10	4.04E-09
1824	4849	DSC2	2.33	1.14E-86	7.13E-85
54360	1019	CYTL1	2.33	4.51E-36	7.89E-35
643707	4563	GOLGA6L4	2.32	3.84E-52	1.10E-50
22885	4038	ABLIM3	2.32	7.70E-129	9.70E-127
56139	5420	PCDHA10	2.32	0.000119721	0.000335946
8382	1237	NME5	2.32	8.96E-11	4.64E-10
147798	2172	TMC4	2.31	1.56E-12	9.12E-12
9053	4028	MAP7	2.31	3.68E-75	1.81E-73
10317	2934	B3GALT5	2.31	1.63E-11	8.89E-11
3899	8673	AFF3	2.31	3.34E-09	1.52E-08
92291	3270	CAPN13	2.31	8.77E-08	3.53E-07
81849	5317	ST6GALNAC5	2.30	3.81E-06	1.30E-05

55281	2072	TMEM140	2.30	1.51E-19	1.34E-18
389114	5584	ZNF662	2.30	3.58E-24	3.90E-23
163732	1312	CITED4	2.30	8.02E-35	1.34E-33
330	6295	BIRC3	2.29	4.39E-183	9.75E-181
1645	1515	AKR1C1	2.29	1.33E-20	1.24E-19
2901	3940	GRIK5	2.29	4.03E-18	3.33E-17
728780	2466	ANKDD1B	2.29	9.11E-07	3.31E-06
134288	1758	TMEM174	2.29	0.00015305	0.000423649
65267	11110	WNK3	2.28	1.23E-12	7.25E-12
51703	3374	ACSL5	2.28	1.06E-35	1.83E-34
8710	1951	SERPINB7	2.28	1.94E-05	6.07E-05
288	7351	ANK3	2.28	3.87E-67	1.61E-65
57576	3823	KIF17	2.28	2.23E-06	7.76E-06
10068	2023	IL18BP	2.27	1.17E-49	3.13E-48
648791	1745	PPP1R3G	2.27	4.65E-08	1.92E-07
341208	5951	HEPHL1	2.27	1.95E-12	1.13E-11
85301	6056	COL27A1	2.26	2.22E-161	3.91E-159
79919	2517	C2orf54	2.26	0.000151784	0.000420714
2353	2158	FOS	2.26	1.43E-66	5.88E-65
1288	6333	COL4A6	2.26	1.75E-15	1.25E-14
79623	2362	GALNT14	2.26	1.18E-175	2.42E-173
3371	6703	TNC	2.26	2.23E-176	4.60E-174
83478	3634	ARHGAP24	2.26	1.71E-85	1.04E-83
92292	2192	GLYATL1	2.26	5.85E-10	2.83E-09
54502	4625	RBM47	2.26	1.03E-137	1.51E-135
203111	1403	ERICH5	2.25	5.59E-28	7.30E-27
1187	2553	CLCNKA	2.25	0.000164625	0.000453639
1.03E+08	2028	LOC102724197	2.25	2.52E-08	1.07E-07
8637	728	EIF4EBP3	2.25	7.85E-05	0.000226577
56146	3070	PCDHA2	2.25	0.000167725	0.000461322
2880	1334	GPX5	2.25	0.000194806	0.000530714
56102	4727	PCDHGB3	2.25	2.34E-08	9.95E-08
27143	4413	PALD1	2.25	2.75E-12	1.57E-11
9413	2171	FAM189A2	2.25	3.39E-21	3.24E-20
3965	1725	LGALS9	2.25	1.19E-15	8.60E-15
240	2566	ALOX5	2.25	2.80E-25	3.22E-24
5648	3451	MASP1	2.25	6.76E-06	2.24E-05
259217	5558	HSPA12A	2.24	3.28E-116	3.43E-114
84302	4293	TMEM246	2.24	3.27E-58	1.10E-56
3081	1757	HGD	2.24	4.67E-63	1.78E-61
6356	925	CCL11	2.24	0.000185022	0.000505531
340527	13034	NHSL2	2.23	1.30E-07	5.15E-07
201501	4435	ZBTB7C	2.23	2.14E-10	1.07E-09
727684	847	C1orf195	2.23	0.000227579	0.000613228
219285	6528	SAMD9L	2.22	1.27E-78	6.65E-77
5627	3599	PROS1	2.22	5.50E-130	7.29E-128
1277	5596	COL1A1	2.22	2.80E-96	2.08E-94
58473	2030	PLEKHB1	2.22	3.03E-39	5.93E-38

127707	5070	KLHDC7A	2.21	1.81E-66	7.41E-65
1238	2978	ACKR2	2.21	3.33E-06	1.14E-05
1400	3074	CRMP1	2.21	1.13E-48	2.90E-47
10265	2107	IRX5	2.21	3.13E-34	5.12E-33
2312	12747	FLG	2.21	1.49E-19	1.32E-18
54869	2395	EPS8L1	2.21	3.25E-07	1.24E-06
287	8312	ANK2	2.21	4.41E-69	1.89E-67
148268	5544	ZNF570	2.20	2.71E-16	2.02E-15
79679	2441	VTCN1	2.20	2.62E-05	8.05E-05
149466	1506	C1orf210	2.20	9.87E-05	0.00028088
8448	2232	DOC2A	2.20	6.01E-07	2.23E-06
1.02E+08	1759	LOC101929773	2.19	3.11E-06	1.07E-05
143689	3214	PIWIL4	2.19	2.38E-13	1.47E-12
56704	4256	JPH1	2.19	1.18E-37	2.19E-36
148753	2903	FAM163A	2.19	5.50E-05	0.000162046
51129	1890	ANGPTL4	2.18	5.89E-36	1.03E-34
7033	1054	TFF3	2.18	0.000301882	0.000799379
23643	647	LY96	2.17	2.84E-05	8.69E-05
148523	1255	CIART	2.17	1.14E-14	7.70E-14
6280	586	S100A9	2.17	5.82E-05	0.000170596
2747	2348	GLUD2	2.17	3.90E-05	0.000117235
2939	1320	GSTA2	2.17	0.000325062	0.000857439
2946	1232	GSTM2	2.17	3.06E-25	3.51E-24
26996	1990	GPR160	2.17	1.65E-48	4.20E-47
115361	6160	GBP4	2.16	7.10E-49	1.83E-47
7096	3786	TLR1	2.16	7.38E-31	1.08E-29
2357	1337	FPR1	2.16	1.46E-23	1.56E-22
1E+08	3441	ZNF705E	2.16	2.74E-07	1.05E-06
375616	3767	KCP	2.15	1.98E-40	4.00E-39
26045	5628	LRRTM2	2.15	1.18E-12	6.96E-12
164045	3490	HFM1	2.15	1.95E-06	6.85E-06
153328	1850	SLC25A48	2.15	0.000373677	0.000976683
56143	5218	PCDHA5	2.14	0.000380859	0.000994869
6288	580	SAA1	2.14	1.09E-189	2.59E-187
730755	880	KRTAP2-3	2.14	2.57E-16	1.93E-15
2264	3063	FGFR4	2.14	1.96E-68	8.36E-67
346389	9167	MACC1	2.14	4.04E-49	1.06E-47
3434	4409	IFIT1	2.14	5.96E-41	1.23E-39
56110	2442	PCDHGA5	2.13	1.20E-06	4.31E-06
57717	4549	PCDHB16	2.13	0.000113687	0.00032053
5332	5600	PLCB4	2.13	1.74E-124	2.00E-122
26253	1264	CLEC4E	2.12	1.39E-53	4.13E-52
26249	6664	KLHL3	2.12	7.47E-25	8.41E-24
79993	3840	ELOVL7	2.12	1.07E-85	6.55E-84
1E+08	967	SHISAL2B	2.11	0.00037121	0.00097052
152078	1147	PQLC2L	2.11	1.37E-05	4.38E-05
51676	2374	ASB2	2.11	6.55E-07	2.41E-06
395	4127	ARHGAP6	2.11	2.37E-36	4.20E-35

387755	2385	INSC	2.11	0.000195526	0.000532429
8048	1464	CSRP3	2.11	0.000482616	0.001238642
283848	2426	CES4A	2.11	4.49E-53	1.32E-51
59272	3519	ACE2	2.10	0.000373778	0.000976805
5317	5384	PKP1	2.10	3.67E-06	1.25E-05
5588	3295	PRKCQ	2.10	5.62E-34	9.15E-33
5673	1598	PSG5	2.10	1.89E-12	1.10E-11
401024	20787	FSIP2	2.10	6.39E-05	0.000186428
7846	1934	TUBA1A	2.10	7.65E-143	1.18E-140
5027	6246	P2RX7	2.10	3.68E-09	1.67E-08
143903	2873	LAYN	2.09	1.03E-22	1.05E-21
6347	760	CCL2	2.09	1.37E-53	4.07E-52
1.08E+08	2697	LOC107984026	2.09	6.33E-55	1.95E-53
171019	5076	ADAMTS19	2.09	0.00053595	0.001365271
1.02E+08	1347	LOC101929141	2.09	3.09E-05	9.41E-05
342979	2303	PALM3	2.08	3.80E-06	1.29E-05
1134	2019	CHRNA1	2.08	0.000446839	0.001154156
23639	3479	LRRC6	2.08	2.79E-27	3.52E-26
54749	2325	EPDR1	2.08	2.23E-107	2.03E-105
401494	4242	HACD4	2.08	4.63E-36	8.10E-35
56833	2547	SLAMF8	2.08	3.94E-10	1.93E-09
254295	1528	PHYHD1	2.08	2.36E-90	1.54E-88
392255	3716	GDF6	2.07	1.37E-05	4.38E-05
440348	1291	NPIPB15	2.07	4.14E-29	5.66E-28
1E+08	4296	LOC100129940	2.07	2.83E-10	1.40E-09
256987	6245	SERINC5	2.07	5.45E-99	4.23E-97
79772	4142	MCTP1	2.06	5.94E-65	2.35E-63
107	10155	ADCY1	2.06	2.90E-10	1.44E-09
83539	3349	CHST9	2.06	8.74E-49	2.25E-47
57460	5730	PPM1H	2.06	1.21E-95	8.78E-94
51478	1518	HSD17B7	2.06	2.86E-49	7.51E-48
10561	1965	IFI44	2.06	1.00E-46	2.45E-45
56114	4602	PCDHGA1	2.05	1.87E-10	9.44E-10
1576	2792	CYP3A4	2.05	0.000551328	0.001403237
503835	3346	DUXA	2.05	4.61E-05	0.000137196
56138	6848	PCDHA11	2.05	0.000646225	0.00162562
752014	1374	CEMP1	2.05	1.25E-16	9.52E-16
7070	2259	THY1	2.05	0.000277805	0.000740036
53632	3412	PRKAG3	2.04	0.00068226	0.001710271
353219	1382	KAAG1	2.04	1.99E-09	9.27E-09
956	2805	ENTPD3	2.04	2.41E-15	1.71E-14
144110	3676	TMEM86A	2.04	4.77E-49	1.24E-47
3687	4196	ITGAX	2.04	6.32E-07	2.33E-06
144195	2782	SLC2A14	2.03	0.000750624	0.001870034
131368	3639	ZPLD1	2.03	0.000143351	0.000398539
55714	10128	TENM3	2.03	2.65E-46	6.42E-45
4060	2116	LUM	2.03	1.19E-92	8.26E-91
129607	2992	CMPK2	2.02	1.10E-17	8.87E-17

401145	14306	CCSER1	2.02	1.51E-23	1.61E-22
5021	4408	OXTR	2.02	5.67E-34	9.23E-33
2104	5444	ESRRG	2.02	2.02E-13	1.25E-12
23475	2187	QPRT	2.02	4.16E-135	5.92E-133
10826	3381	FAXDC2	2.02	5.08E-29	6.91E-28
26585	4114	GREM1	2.02	5.18E-102	4.28E-100
54716	5396	SLC6A20	2.02	6.19E-09	2.77E-08
84952	7253	CGNL1	2.02	9.79E-85	5.84E-83
51171	1233	HSD17B14	2.02	6.33E-12	3.55E-11
6289	714	SAA2	2.02	1.17E-163	2.14E-161
79054	5946	TRPM8	2.01	8.15E-05	0.000234366
6559	4688	SLC12A3	2.01	0.000332343	0.000875344
256815	2036	C10orf67	2.00	1.34E-06	4.79E-06
6236	1468	RRAD	2.00	1.08E-78	5.71E-77
155368	969	METTL27	2.00	2.79E-09	1.28E-08
5341	2866	PLEK	2.00	3.36E-09	1.53E-08
285513	12762	GPRIN3	2.00	2.56E-16	1.92E-15
1015	3698	CDH17	1.99	0.00038862	0.001013802
8170	3765	SLC14A2	1.99	1.82E-06	6.40E-06
29958	7079	DMGDH	1.99	3.30E-11	1.77E-10
90139	4241	TSPAN18	1.99	7.49E-51	2.05E-49
4129	2600	MAOB	1.99	1.94E-10	9.76E-10
161291	4443	TMEM30B	1.99	5.68E-05	0.000166837
9626	886	GUCA1C	1.99	0.000958292	0.00234337
387758	3024	FIBIN	1.99	1.77E-08	7.63E-08
84519	1886	ACRBP	1.98	4.23E-05	0.000126527
5042	2430	PABPC3	1.98	0.00100589	0.002450659
343990	4127	KIAA1211L	1.98	2.04E-32	3.14E-31
144453	2799	BEST3	1.98	4.38E-07	1.65E-06
79674	2383	VEPH1	1.98	6.72E-58	2.24E-56
23529	1860	CLCF1	1.98	2.78E-92	1.90E-90
1316	4671	KLF6	1.98	1.08E-191	2.63E-189
3078	1103	CFHR1	1.97	0.000244095	0.000655149
11250	1725	GPR45	1.97	0.000590384	0.001496426
164312	2910	LRRN4	1.97	1.27E-74	6.19E-73
1804	3918	DPP6	1.97	0.00106914	0.002594792
1646	3419	AKR1C2	1.97	7.68E-08	3.11E-07
4192	925	MDK	1.97	1.26E-14	8.49E-14
10158	894	PDZK1IP1	1.97	5.45E-198	1.47E-195
145581	4889	LRFN5	1.97	8.64E-08	3.48E-07
5153	3407	PDE1B	1.97	0.000479747	0.001231813
22917	1377	ZP1	1.97	2.41E-08	1.02E-07
83729	2453	INHBE	1.97	1.72E-06	6.06E-06
3663	2860	IRF5	1.96	1.49E-49	3.93E-48
1088	2333	CEACAM8	1.96	0.001031193	0.002506806
728215	3800	FAM155A	1.96	2.12E-34	3.49E-33
23562	1674	CLDN14	1.96	0.000516666	0.001319931
56134	6036	PCDHAC2	1.95	0.001117826	0.002701515

10418	5154	SPON1	1.95	9.51E-07	3.44E-06
4862	3772	NPAS2	1.95	4.40E-49	1.15E-47
53938	1142	PPIL3	1.95	9.10E-63	3.43E-61
6505	3771	SLC1A1	1.95	3.56E-47	8.80E-46
4314	1906	MMP3	1.94	0.001237917	0.002967848
9957	2432	HS3ST1	1.94	3.89E-19	3.38E-18
1272	5277	CNTN1	1.94	0.000962888	0.002352667
167838	4915	TXLNB	1.94	0.001310451	0.003126055
6317	1793	SERPINB3	1.94	4.02E-10	1.96E-09
83445	2497	GSG1	1.94	5.99E-05	0.000175277
56100	4599	PCDHGB6	1.93	2.55E-07	9.79E-07
23052	4693	ENDOD1	1.93	4.37E-96	3.21E-94
92483	1773	LDHAL6B	1.93	7.31E-05	0.000211631
26059	6272	ERC2	1.93	5.95E-08	2.44E-07
57412	2477	AS3MT	1.93	1.84E-59	6.36E-58
6768	3319	ST14	1.93	6.08E-44	1.39E-42
25801	1844	GCA	1.93	4.17E-34	6.80E-33
353324	2543	SPATA12	1.93	0.000673226	0.001689292
1.05E+08	1252	LOC105376678	1.93	5.38E-05	0.000158802
2329	2274	FMO4	1.93	1.23E-14	8.31E-14
91653	3765	BOC	1.92	1.98E-30	2.84E-29
139067	606	SPANXN3	1.92	0.001412299	0.003352088
1E+08	1866	ZNF705G	1.92	0.001468015	0.00347412
9737	5341	GPRASP1	1.92	4.39E-09	1.99E-08
9242	2075	MSC	1.91	6.29E-11	3.31E-10
94120	2812	SYTL3	1.91	4.87E-30	6.88E-29
284021	1503	MILR1	1.91	1.26E-18	1.07E-17
83937	2907	RASSF4	1.91	3.98E-99	3.10E-97
353088	4274	ZNF429	1.91	1.10E-14	7.44E-14
1308	5610	COL17A1	1.90	2.80E-14	1.86E-13
54858	6329	PGPEP1	1.90	1.13E-75	5.63E-74
8857	16407	FCGBP	1.90	1.44E-40	2.91E-39
1.08E+08	3002	LOC107985425	1.90	0.000463009	0.001192972
56135	5475	PCDHAC1	1.90	0.001597703	0.003752047
254228	9796	CALHM5	1.89	2.85E-05	8.72E-05
388	2387	RHOB	1.89	2.99E-186	6.90E-184
2272	1892	FHIT	1.89	8.24E-15	5.64E-14
145407	5638	C14orf37	1.89	2.55E-27	3.23E-26
124961	5011	ZFP3	1.89	2.54E-05	7.82E-05
80036	3370	TRPM3	1.89	2.06E-05	6.42E-05
9415	3181	FADS2	1.89	1.05E-128	1.32E-126
6364	849	CCL20	1.89	1.24E-09	5.85E-09
6335	9768	SCN9A	1.89	3.63E-16	2.69E-15
167127	2385	UGT3A2	1.89	0.001597953	0.003752137
9079	2636	LDB2	1.88	4.95E-06	1.66E-05
284076	3559	TTLL6	1.88	1.38E-43	3.10E-42
222584	6269	FAM83B	1.88	3.23E-13	1.97E-12
6299	5155	SALL1	1.88	4.57E-127	5.48E-125

285671	5430	RNF180	1.88	7.01E-08	2.85E-07
1.08E+08	2559	LOC107986113	1.88	1.97E-10	9.92E-10
7093	6788	TLL2	1.87	4.36E-49	1.14E-47
1.01E+08	2298	LOC100505650	1.87	1.35E-42	2.96E-41
283463	25332	MUC19	1.87	6.76E-07	2.49E-06
5924	7711	RASGRF2	1.87	1.17E-62	4.41E-61
4485	2723	MST1	1.87	1.29E-44	2.98E-43
54894	5318	RNF43	1.87	4.16E-16	3.06E-15
133923	1979	ZNF474	1.86	0.000769732	0.00191415
389376	462	SFTA2	1.86	0.001681502	0.003932727
1.08E+08	1049	LOC107985384	1.86	0.002023593	0.00466496
84911	2819	ZNF382	1.86	0.00036578	0.000956886
4856	2601	NOV	1.86	2.49E-29	3.43E-28
6318	1724	SERPINB4	1.86	1.13E-06	4.05E-06
57571	5062	CARNS1	1.86	5.53E-13	3.31E-12
10631	3225	POSTN	1.85	0.001209767	0.002907107
130940	2301	CCDC148	1.85	3.58E-13	2.18E-12
80157	1925	CWH43	1.85	0.002016227	0.004650094
8572	2303	PDLIM4	1.85	2.43E-107	2.20E-105
3880	1490	KRT19	1.85	8.47E-107	7.60E-105
55630	731	SLC39A4	1.85	2.89E-35	4.92E-34
130367	4863	SGPP2	1.85	6.54E-110	6.28E-108
4072	1731	EPCAM	1.84	2.20E-74	1.06E-72
8991	1731	SELENBP1	1.84	1.59E-32	2.47E-31
64332	3889	NFKBIZ	1.84	5.31E-24	5.75E-23
2841	2471	GPR18	1.84	0.002173622	0.004980132
1.05E+08	1685	LOC105369972	1.83	4.44E-06	1.50E-05
440345	5447	NPIPB4	1.83	7.27E-16	5.29E-15
221692	5424	PHACTR1	1.83	8.38E-20	7.52E-19
2212	2286	FCGR2A	1.83	0.000619826	0.001563468
113763	2896	ZBED6CL	1.83	1.01E-68	4.32E-67
57604	9800	TRMT9B	1.83	3.19E-14	2.11E-13
22891	4052	ZNF365	1.83	6.82E-14	4.41E-13
51361	5865	HOOK1	1.82	1.69E-73	7.99E-72
80017	2916	DGLUCY	1.82	8.89E-85	5.32E-83
22941	9301	SHANK2	1.82	7.15E-86	4.40E-84
285498	1181	RNF212	1.82	0.002419246	0.005496824
56097	4641	PCDHGC5	1.82	1.79E-06	6.29E-06
339965	4346	CCDC158	1.82	0.00244572	0.005553425
1950	5435	EGF	1.82	5.55E-13	3.32E-12
134265	5730	AFAP1L1	1.82	1.07E-60	3.84E-59
27071	2716	DAPP1	1.82	0.000219806	0.000593724
57379	2794	AICDA	1.81	0.001192051	0.002867248
259294	1002	TAS2R19	1.81	0.00183171	0.004255469
56899	5907	ANKS1B	1.81	1.06E-06	3.81E-06
9388	4188	LIPG	1.81	5.52E-63	2.09E-61
10203	5543	CALCRL	1.81	4.41E-11	2.35E-10
1154	2548	CISH	1.80	2.24E-32	3.45E-31

1622	637	DBI	1.80	3.64E-91	2.40E-89
1282	6532	COL4A1	1.80	1.40E-149	2.30E-147
595	4304	CCND1	1.80	1.01E-91	6.85E-90
29119	10063	CTNNA3	1.80	0.002912245	0.006522725
387264	942	KRTAP5-1	1.80	0.001969954	0.004549278
440603	4986	BCL2L15	1.79	1.76E-27	2.25E-26
6271	607	S100A1	1.79	5.34E-22	5.29E-21
5822	3277	PWP2	1.79	5.63E-23	5.84E-22
9586	8427	CREB5	1.79	8.91E-52	2.51E-50
126410	2620	CYP4F22	1.79	0.002792424	0.006270953
8437	4695	RASAL1	1.79	9.14E-40	1.82E-38
999	4808	CDH1	1.79	7.18E-22	7.08E-21
646643	1568	SBK2	1.79	0.001219865	0.002927807
57482	6562	KIAA1211	1.79	1.14E-12	6.69E-12
1953	7128	MEGF6	1.79	1.86E-52	5.38E-51
126868	3232	MAB21L3	1.79	1.30E-18	1.10E-17
64881	4926	PCDH20	1.79	5.58E-13	3.34E-12
1890	1689	TYMP	1.78	9.77E-11	5.05E-10
284297	4899	SSC5D	1.78	5.97E-39	1.16E-37
221806	5088	VWDE	1.78	2.40E-14	1.60E-13
196446	3142	MYRFL	1.78	0.000363719	0.000951917
6584	2748	SLC22A5	1.78	1.21E-51	3.39E-50
128344	2340	PIFO	1.77	4.31E-10	2.10E-09
2053	2228	EPHX2	1.77	2.75E-07	1.05E-06
92359	1026	CRB3	1.77	1.12E-20	1.05E-19
6565	4524	SLC15A2	1.77	0.000176785	0.000484664
285386	6029	TPRG1	1.77	0.000686284	0.001719386
3750	4185	KCND1	1.77	6.78E-30	9.55E-29
11145	1215	PLA2G16	1.77	6.33E-122	6.94E-120
79071	3052	ELOVL6	1.77	1.42E-103	1.21E-101
2099	2888	ESR1	1.77	1.45E-12	8.50E-12
2982	6529	GUCY1A1	1.77	1.86E-23	1.97E-22
10873	2203	ME3	1.77	2.23E-28	2.94E-27
388698	8690	FLG2	1.76	0.003283164	0.007285533
3816	886	KLK1	1.76	0.00127457	0.003045529
8291	6777	DYSF	1.76	1.25E-69	5.44E-68
6876	1182	TAGLN	1.76	2.59E-85	1.57E-83
307	2196	ANXA4	1.76	2.37E-132	3.29E-130
148808	3827	MFSD4A	1.76	1.71E-06	6.04E-06
70	3693	ACTC1	1.76	0.001624318	0.003809012
1.08E+08	2988	LOC107984115	1.76	5.51E-14	3.59E-13
84451	5793	MAP3K21	1.76	5.79E-49	1.50E-47
1258	4914	CNGB1	1.76	2.01E-07	7.82E-07
9514	1732	GAL3ST1	1.76	8.58E-11	4.46E-10
1.05E+08	3120	LOC105379177	1.76	7.18E-05	0.000208124
116211	1053	TM4SF19	1.76	3.19E-05	9.70E-05
8876	3844	VNN1	1.75	9.30E-11	4.81E-10
149628	1666	PYHIN1	1.75	0.000222562	0.000600619

56103	4475	PCDHGB2	1.75	3.49E-22	3.50E-21
6579	7695	SLCO1A2	1.75	0.00083139	0.002052255
81035	3134	COLEC12	1.75	1.68E-64	6.60E-63
5343	3011	PLGLB1	1.75	0.001224498	0.002937737
4938	1885	OAS1	1.75	1.32E-27	1.69E-26
55160	4082	ARHGEF10L	1.74	6.14E-76	3.08E-74
89795	9285	NAV3	1.74	1.87E-11	1.01E-10
729873	2260	TBC1D3	1.74	0.00286567	0.006426507
7114	657	TMSB4X	1.74	1.18E-81	6.55E-80
11213	8273	IRAK3	1.74	4.36E-40	8.73E-39
3224	2290	HOXC8	1.74	6.28E-35	1.06E-33
348938	3279	NIPAL4	1.74	1.77E-39	3.49E-38
196996	3564	GRAMD2A	1.74	2.05E-07	7.97E-07
128434	1981	VSTM2L	1.74	2.05E-25	2.38E-24
440738	1367	MAP1LC3C	1.73	3.54E-06	1.21E-05
9985	2478	REC8	1.73	2.80E-22	2.82E-21
4544	2935	MTNR1B	1.73	0.003768784	0.008284517
10361	1163	NPM2	1.73	4.12E-10	2.01E-09
283710	885	LOC283710	1.72	0.004173565	0.009095788
84418	926	CYSTM1	1.72	2.31E-55	7.19E-54
26166	2732	RGS22	1.72	0.004338615	0.009433248
503834	648	DPRX	1.72	0.003932806	0.008608859
283284	4360	IGSF22	1.72	0.003911925	0.008568427
79366	1941	HMGN5	1.72	2.59E-39	5.08E-38
58528	4965	RRAGD	1.72	3.86E-63	1.47E-61
387597	8053	ILDR2	1.72	6.07E-66	2.46E-64
80270	2193	HSD3B7	1.72	1.29E-34	2.14E-33
117153	2351	NA	1.72	8.93E-15	6.10E-14
1379	1837	CR1L	1.71	0.00259168	0.005860138
51176	2554	LEF1	1.71	2.86E-94	2.04E-92
167681	2527	PRSS35	1.71	9.37E-05	0.000267424
1649	1051	DDIT3	1.71	1.73E-32	2.68E-31
114793	5178	FMNL2	1.71	1.15E-115	1.18E-113
60506	3059	NYX	1.71	0.004508706	0.009785108
10154	7023	PLXNC1	1.71	3.22E-10	1.59E-09
333926	1762	PPM1J	1.71	8.26E-10	3.95E-09
8187	2783	ZNF239	1.70	4.66E-19	4.02E-18
5169	3247	ENPP3	1.70	2.18E-05	6.79E-05
84842	1803	HPDL	1.70	2.33E-06	8.09E-06
80255	8592	SLC35F5	1.70	1.87E-100	1.50E-98
3316	858	HSPB2	1.70	5.56E-05	0.000163692
51146	1830	A4GNT	1.70	0.001478307	0.003497545
375061	1516	FAM89A	1.70	7.93E-06	2.61E-05
59307	1701	SIGIRR	1.70	2.34E-28	3.09E-27
10129	10390	FRY	1.69	2.44E-11	1.32E-10
79413	2226	ZBED2	1.69	4.83E-25	5.51E-24
415	2130	ARSE	1.69	3.79E-41	7.85E-40
8878	2694	SQSTM1	1.69	1.79E-125	2.09E-123

341346	4154	SMCO2	1.68	8.18E-05	0.000235202
8519	733	IFITM1	1.68	1.61E-43	3.60E-42
51635	2042	DHRS7	1.68	1.89E-82	1.08E-80
27094	1811	KCNMB3	1.68	4.24E-13	2.56E-12
596	5588	BCL2	1.68	5.68E-36	9.92E-35
92558	3039	BICDL1	1.68	1.31E-43	2.94E-42
1.03E+08	1139	LOC102725009	1.68	4.43E-05	0.000132319
1489	1678	CTF1	1.68	1.10E-11	6.06E-11
5334	5977	PLCL1	1.68	0.004829497	0.010422749
7097	4197	TLR2	1.68	3.37E-31	4.99E-30
2537	838	IFI6	1.68	3.95E-09	1.79E-08
7915	5144	ALDH5A1	1.67	9.24E-32	1.40E-30
133396	3903	IL31RA	1.67	0.000903252	0.002217934
60676	9604	PAPPA2	1.67	0.003566436	0.007872833
154215	3031	NKAIN2	1.67	0.005575849	0.011884798
84870	2322	RSPO3	1.67	7.96E-29	1.08E-27
79780	3124	CCDC82	1.67	3.93E-81	2.17E-79
414157	1264	C10orf62	1.67	0.00342026	0.007562366
338440	2769	ANO9	1.67	1.96E-44	4.51E-43
56271	1282	BEX4	1.67	1.21E-29	1.69E-28
132989	1398	C4orf36	1.67	1.41E-10	7.17E-10
1.01E+08	1680	LOC100652901	1.67	5.22E-13	3.13E-12
79815	4508	NIPAL2	1.67	5.38E-27	6.73E-26
84766	3600	CRACR2A	1.66	0.002740183	0.006162983
148641	3049	SLC35F3	1.66	1.58E-15	1.13E-14
6695	4841	SPOCK1	1.66	7.53E-140	1.13E-137
58472	1942	SQOR	1.66	3.40E-65	1.35E-63
3398	1402	ID2	1.66	1.07E-54	3.28E-53
10083	2180	USH1C	1.66	0.005933816	0.012573886
1.08E+08	1918	LOC107984341	1.66	0.000206521	0.000560138
10123	4014	ARL4C	1.66	2.88E-117	3.03E-115
158880	4436	USP51	1.66	1.15E-16	8.82E-16
1767	13300	DNAH5	1.66	8.64E-10	4.13E-09
134526	3271	ACOT12	1.66	0.000184947	0.000505531
255631	6500	COL24A1	1.65	0.000277076	0.000738204
387700	4523	SLC16A12	1.65	2.98E-56	9.52E-55
56123	3503	PCDHB13	1.65	6.71E-05	0.000195212
339768	3511	ESPNL	1.65	1.48E-08	6.41E-08
1491	2240	CTH	1.65	1.42E-15	1.02E-14
284307	4206	ZIK1	1.65	3.37E-10	1.66E-09
25903	3857	OLFML2B	1.65	6.14E-07	2.27E-06
11001	2408	SLC27A2	1.65	1.35E-18	1.14E-17
80731	4566	THSD7B	1.65	0.005585373	0.011902244
1794	5926	DOCK2	1.64	2.45E-18	2.04E-17
5624	1776	PROC	1.64	0.001124421	0.002716343
1.03E+08	1581	LOC102724720	1.64	0.005268566	0.011293508
57088	3282	PLSCR4	1.64	1.83E-18	1.54E-17
477	5496	ATP1A2	1.64	0.006184223	0.013071766

199699	1732	DAND5	1.64	5.90E-05	0.000172888
941	2433	CD80	1.64	0.005818775	0.012359594
58189	1479	WFDC1	1.64	0.006655759	0.013984908
340371	3605	NRBP2	1.64	1.82E-59	6.33E-58
132014	2407	IL17RE	1.64	4.60E-13	2.77E-12
7139	1165	TNNT2	1.63	0.006733394	0.014127474
389434	3706	IYD	1.63	0.00666336	0.01399794
4065	6936	LY75	1.63	1.65E-08	7.12E-08
29091	3922	STXBP6	1.63	4.42E-06	1.50E-05
1.07E+08	1715	PRH1-TAS2R14	1.63	0.000154886	0.000427997
4131	11830	MAP1B	1.63	1.13E-125	1.33E-123
401944	3990	LDLRAD2	1.63	0.005359669	0.011466659
352999	1201	C6orf58	1.63	0.005513757	0.011758094
440107	3267	PLEKHG7	1.63	0.00499433	0.01073935
132160	2108	PPM1M	1.63	1.30E-32	2.03E-31
57624	5104	NYAP2	1.63	0.001233565	0.002958293
79152	2148	FA2H	1.63	2.06E-05	6.43E-05
3760	4523	KCNJ3	1.63	0.006553539	0.013783532
124739	4185	USP43	1.62	3.67E-29	5.02E-28
222235	4429	FBXL13	1.62	0.000201465	0.000547344
9162	8025	DGKI	1.62	2.96E-21	2.84E-20
10247	1011	RIDA	1.62	1.26E-23	1.36E-22
200132	2372	TCTEX1D1	1.62	0.006082061	0.012871136
2745	1116	GLRX	1.62	3.81E-58	1.28E-56
151871	1224	DPPA2	1.62	0.006281171	0.013249893
120892	6507	LRRK2	1.62	2.62E-30	3.75E-29
7504	5089	XK	1.62	0.006725673	0.014113825
340547	3187	VSIG1	1.62	1.95E-10	9.82E-10
89866	4024	SEC16B	1.61	4.46E-11	2.37E-10
11077	3882	HSF2BP	1.61	5.73E-16	4.20E-15
8999	4623	CDKL2	1.61	1.31E-34	2.17E-33
11343	4268	MGLL	1.61	7.14E-79	3.80E-77
1E+08	1701	PRSS43	1.61	0.006268026	0.013226875
2846	4208	LPAR4	1.61	8.06E-07	2.94E-06
174	2057	AFP	1.61	0.007090518	0.014797313
221687	3392	RNF182	1.61	4.40E-13	2.65E-12
54848	2703	ARHGEF38	1.61	0.002677743	0.006040901
23255	6297	MTCL1	1.61	1.53E-81	8.51E-80
10451	3127	VAV3	1.61	2.71E-18	2.26E-17
201176	3565	ARHGAP27	1.61	1.88E-63	7.18E-62
238	6267	ALK	1.61	0.00722354	0.015050171
3898	2869	LAD1	1.60	2.95E-06	1.02E-05
1601	4615	DAB2	1.60	1.79E-79	9.64E-78
25961	2044	NUDT13	1.60	1.51E-09	7.06E-09
2915	7855	GRM5	1.60	0.006855481	0.014354017
56311	1358	ANKRD7	1.60	0.000627014	0.001580029
9881	10352	TRANK1	1.60	2.05E-47	5.11E-46
165631	4182	PARP15	1.59	1.78E-65	7.12E-64

79081	1357	LBHD1	1.59	6.78E-14	4.38E-13
23705	4397	CADM1	1.59	1.50E-23	1.59E-22
116372	3633	LYPD1	1.59	4.84E-44	1.11E-42
55679	1926	LIMS2	1.59	5.98E-42	1.27E-40
80201	3689	HKDC1	1.59	3.88E-71	1.74E-69
6262	16051	RYR2	1.59	0.008521611	0.017506299
55530	6425	SVOP	1.59	3.52E-05	0.000106528
445328	2474	ARHGEF35	1.59	1.86E-34	3.08E-33
4056	680	LTC4S	1.58	0.00126098	0.003016709
166012	1792	CHST13	1.58	0.007361681	0.015318228
147719	4116	LYPD4	1.58	0.008865444	0.018160158
64922	3624	LRRC19	1.58	0.006826097	0.014300918
114770	2599	PGLYRP2	1.57	0.00897271	0.018356604
84707	912	BEX2	1.57	6.39E-12	3.58E-11
441457	3193	NUTM2G	1.57	0.000248839	0.000667175
642587	978	MIR205HG	1.57	5.31E-07	1.97E-06
10647	454	SCGB1D2	1.57	0.007764868	0.016083723
55314	3047	TMEM144	1.57	3.16E-17	2.50E-16
1.01E+08	508	LOC100996747	1.56	0.009594082	0.019515472
26353	2056	HSPB8	1.56	1.55E-57	5.15E-56
361	5236	AQP4	1.56	0.000299188	0.00079272
150590	1545	C2orf15	1.56	3.41E-07	1.30E-06
27132	2531	CPNE7	1.56	2.70E-60	9.55E-59
9752	6237	PCDHA9	1.56	0.008417087	0.017303574
1235	3216	CCR6	1.56	0.001523183	0.003593191
440712	1704	RHEX	1.56	4.59E-49	1.19E-47
1.03E+08	1761	LOC102723872	1.56	1.00E-05	3.24E-05
64063	1391	PRSS22	1.55	0.008383357	0.017242213
946	3847	SIGLEC6	1.55	0.002208677	0.005053931
4015	4898	LOX	1.55	3.40E-115	3.48E-113
2888	2151	GRB14	1.55	1.97E-28	2.61E-27
140458	3624	ASB5	1.55	0.008342467	0.017168051
8715	2880	NOL4	1.55	0.008642431	0.017738097
64081	2562	PBLD	1.55	1.32E-35	2.27E-34
133522	10525	PPARGC1B	1.55	7.08E-12	3.95E-11
79587	1880	CARS2	1.54	2.11E-70	9.30E-69
6327	4922	SCN2B	1.54	0.008825718	0.018087123
57804	1749	POLD4	1.54	1.89E-50	5.12E-49
64080	1328	RBKS	1.54	3.87E-11	2.07E-10
5025	1701	P2RX4	1.54	1.78E-41	3.71E-40
1E+08	1146	SMIM6	1.54	9.51E-06	3.09E-05
1E+08	1318	LIMS4	1.54	4.44E-13	2.67E-12
399726	2006	CASC10	1.54	1.87E-07	7.29E-07
1.08E+08	3361	LOC107984512	1.54	0.000796751	0.001973881
84978	5034	FRMD5	1.54	1.98E-26	2.40E-25
642938	3536	FAM196A	1.53	0.000444578	0.001149484
81788	3212	NUAK2	1.53	4.24E-76	2.14E-74
8714	4634	ABCC3	1.53	1.18E-78	6.20E-77

55283	2756	MCOLN3	1.53	8.88E-43	1.95E-41
9495	6487	AKAP5	1.53	2.43E-59	8.39E-58
1.03E+08	2520	LOC102724994	1.53	0.000818918	0.002024283
3976	3916	LIF	1.53	3.19E-55	9.88E-54
55064	3810	SPATA6L	1.53	9.75E-13	5.77E-12
64506	3150	CPEB1	1.53	1.57E-09	7.34E-09
1.05E+08	4017	LOC105371419	1.53	0.00637755	0.013442029
1075	2057	CTSC	1.52	8.33E-99	6.41E-97
1.08E+08	963	LOC107986877	1.52	0.008681909	0.017810896
1287	6348	COL4A5	1.52	1.70E-72	7.84E-71
1586	1895	CYP17A1	1.52	0.010336463	0.020889248
79056	4654	PRRG4	1.52	3.39E-32	5.19E-31
817	5284	CAMK2D	1.52	1.36E-44	3.14E-43
23007	6114	PLCH1	1.52	1.37E-17	1.11E-16
23780	2590	APOL2	1.52	4.09E-127	4.95E-125
1.08E+08	1146	LOC107985770	1.52	3.45E-06	1.18E-05
8620	854	NPFF	1.52	1.13E-05	3.65E-05
55092	1960	TMEM51	1.52	2.88E-59	9.92E-58
123103	5821	KLHL33	1.51	3.55E-15	2.48E-14
27098	2643	CLUL1	1.51	0.000687412	0.00172197
22822	5913	PHLDA1	1.51	2.26E-55	7.05E-54
160419	3879	C12orf50	1.51	2.40E-32	3.69E-31
26011	11114	TENM4	1.51	7.03E-93	4.92E-91
5328	2396	PLAU	1.51	2.40E-56	7.69E-55
4810	8352	NHS	1.51	2.01E-42	4.36E-41
112609	2146	MRAP2	1.51	0.001664797	0.003897248
6376	3338	CX3CL1	1.51	7.79E-41	1.59E-39
162993	3410	ZNF846	1.51	8.43E-20	7.57E-19
339559	2412	ZFP69	1.51	1.60E-08	6.91E-08
1262	3271	CNGA4	1.51	0.011961575	0.023837288
7164	1249	TPD52L1	1.50	7.41E-44	1.69E-42
6582	2512	SLC22A2	1.50	0.01037782	0.020965674
4588	8003	MUC6	1.50	0.004022213	0.008792653
54798	6527	DCHS2	1.50	9.90E-06	3.21E-05
343505	1611	NBPF7	1.50	0.00020666	0.000560428
57761	2263	TRIB3	1.50	2.97E-35	5.05E-34
57821	1899	CCDC181	1.50	0.000123483	0.000345628
22837	9414	COBLL1	1.50	1.43E-70	6.30E-69
1.01E+08	1208	HERC2P11	1.50	0.000282483	0.000751822
1E+08	1176	NA	1.49	0.012936686	0.025612912
89848	3394	FCHSD1	1.49	6.37E-68	2.70E-66
9050	3026	PSTPIP2	1.49	3.20E-50	8.61E-49
5788	1545	PTPRC	1.49	0.012325922	0.024505649
10891	6358	PPARGC1A	1.49	6.51E-31	9.54E-30
4642	5501	MYO1D	1.49	2.95E-57	9.71E-56
29760	2641	BLNK	1.49	5.70E-14	3.70E-13
728841	4624	NBPF8	1.49	1.74E-42	3.78E-41
2150	2883	F2RL1	1.49	2.68E-72	1.23E-70

51561	1090	IL23A	1.49	0.000139179	0.000387424
9687	5671	GREB1	1.49	2.40E-12	1.38E-11
9229	4391	DLGAP1	1.49	0.000457655	0.001180547
11067	2060	DEPP1	1.48	2.11E-18	1.77E-17
64859	3212	NABP1	1.48	2.19E-39	4.30E-38
132864	6785	CPEB2	1.48	1.02E-37	1.89E-36
1813	2626	DRD2	1.48	0.013678473	0.026977368
146857	5263	SLFN13	1.48	1.50E-44	3.46E-43
80117	1335	ARL14	1.48	0.007971977	0.016460905
55507	1227	GPRC5D	1.48	0.007158329	0.014928309
84851	2721	TRIM52	1.48	9.67E-32	1.46E-30
6553	3586	SLC9A5	1.48	6.69E-16	4.88E-15
10625	4351	IVNS1ABP	1.48	2.82E-105	2.49E-103
144608	1168	C12orf60	1.48	0.000937773	0.002298254
8398	2847	PLA2G6	1.48	2.66E-22	2.67E-21
7784	1462	ZP3	1.48	5.60E-06	1.88E-05
10673	2654	TNFSF13B	1.47	2.46E-05	7.61E-05
79703	2146	C11orf80	1.47	2.82E-54	8.59E-53
647309	3624	GMNC	1.47	0.010304537	0.020836578
140876	3793	RIPOR3	1.47	5.75E-28	7.50E-27
6792	9936	CDKL5	1.47	1.00E-21	9.79E-21
11000	2430	SLC27A3	1.47	3.11E-13	1.90E-12
1.08E+08	1253	LOC107986084	1.47	0.000789206	0.00195628
241	921	ALOX5AP	1.47	1.51E-08	6.54E-08
5629	8268	PROX1	1.47	0.000772885	0.001920914
126069	3100	ZNF491	1.47	0.000490263	0.001256816
79750	2382	ZNF385D	1.47	7.13E-07	2.61E-06
3437	2524	IFIT3	1.47	8.20E-29	1.11E-27
123920	1995	CMTM3	1.47	6.95E-67	2.86E-65
1.05E+08	1963	LOC105370399	1.47	0.014751679	0.028907888
2352	603	FOLR3	1.46	0.011862646	0.023642792
139411	12949	PTCHD1	1.46	0.012333077	0.024517131
5325	3351	PLAGL1	1.46	2.09E-43	4.66E-42
1E+08	3127	CTAGE8	1.46	3.54E-15	2.48E-14
8807	1897	IL18RAP	1.46	0.01542765	0.030136158
135656	5306	DPCR1	1.46	0.006724011	0.014112003
23245	3440	ASTN2	1.46	6.85E-39	1.32E-37
339416	2652	ANKRD45	1.46	0.000661567	0.001661915
91526	6901	ANKRD44	1.46	1.51E-28	2.01E-27
3695	2738	ITGB7	1.45	3.59E-05	0.000108267
23117	3638	NPIPB3	1.45	3.43E-07	1.30E-06
1.03E+08	2394	LOC102723360	1.45	0.005955745	0.01261885
10791	694	VAMP5	1.45	0.002444315	0.005550943
10060	8325	ABCC9	1.45	2.56E-34	4.20E-33
57864	2518	SLC46A2	1.45	0.011267514	0.022583326
338645	4181	LUZP2	1.45	0.013055899	0.0258269
7363	1899	UGT2B4	1.45	0.013055899	0.0258269
6446	2423	SGK1	1.45	5.31E-82	2.98E-80

66000	3843	TMEM108	1.45	0.00703871	0.014703009
123876	3351	ACSM2A	1.45	0.008898965	0.018214123
6514	2734	SLC2A2	1.45	0.013150305	0.025999179
92285	6210	ZNF585B	1.45	0.004074375	0.008896828
1E+08	560	TSTD1	1.44	4.11E-24	4.47E-23
79083	2825	MLPH	1.44	1.58E-35	2.72E-34
27122	2568	DKK3	1.44	9.61E-89	6.14E-87
1.01E+08	2490	C5orf66	1.44	4.16E-08	1.73E-07
51380	2572	CSAD	1.44	1.89E-26	2.29E-25
7718	1379	ZNF165	1.44	1.42E-11	7.74E-11
8690	3366	JRKL	1.44	5.28E-40	1.06E-38
1E+08	3827	NPIPB5	1.44	2.00E-06	7.00E-06
5274	1711	SERPINI1	1.44	6.20E-07	2.29E-06
2857	2157	GPR34	1.44	0.009574549	0.019482432
9982	1369	FGFBP1	1.43	0.006872341	0.01438254
5480	1241	PPIC	1.43	5.72E-56	1.81E-54
28971	587	AAMDC	1.43	3.02E-25	3.47E-24
3620	1944	IDO1	1.43	0.000733375	0.00182937
6326	8667	SCN2A	1.43	3.43E-08	1.44E-07
83959	3326	SLC4A11	1.43	3.03E-38	5.75E-37
90249	3759	UNC5A	1.43	1.42E-20	1.32E-19
341880	1563	SLC35F4	1.43	0.017565688	0.033913631
3586	1629	IL10	1.43	0.017278008	0.033412673
3049	653	HBQ1	1.43	0.016169476	0.03145727
1.03E+08	700	LOC102724813	1.42	9.69E-05	0.000276118
147710	1179	IGSF23	1.42	0.006987814	0.014608715
12	1629	SERPINA3	1.42	6.82E-07	2.51E-06
3777	4407	KCNK3	1.42	1.54E-20	1.43E-19
27286	2206	SRPX2	1.42	9.14E-05	0.000261076
81872	776	KRTAP2-1	1.42	0.010493152	0.021172192
1.05E+08	2782	LOC105370641	1.42	0.018794638	0.036039621
728047	4847	GOLGA8O	1.42	2.53E-08	1.07E-07
23101	5598	MCF2L2	1.42	4.23E-11	2.25E-10
4053	8307	LTBP2	1.42	2.70E-53	7.98E-52
7069	1182	THRSP	1.42	0.014832789	0.029060425
285489	2307	DOK7	1.42	1.39E-11	7.63E-11
286343	2112	LURAP1L	1.42	1.30E-05	4.15E-05
253512	3579	SLC25A30	1.42	5.05E-45	1.19E-43
118788	4515	PIK3AP1	1.42	6.05E-39	1.17E-37
57715	4243	SEMA4G	1.41	7.10E-14	4.58E-13
55282	2370	LRRC36	1.41	3.19E-09	1.46E-08
53905	5041	DUOX1	1.41	0.000271733	0.00072473
6833	3137	ABCC8	1.41	0.015050421	0.029453911
55803	2757	ADAP2	1.41	0.000206217	0.000559399
389941	2493	C1QL3	1.41	3.46E-05	0.00010457
2983	3252	GUCY1B1	1.41	1.56E-33	2.50E-32
6150	758	MRPL23	1.41	1.88E-31	2.81E-30
9762	4354	LZTS3	1.41	2.27E-43	5.05E-42

57674	17504	RNF213	1.41	2.48E-82	1.41E-80
1803	3513	DPP4	1.41	3.79E-84	2.23E-82
5149	1822	PDE6H	1.41	0.011096898	0.022279059
389118	2514	CDHR4	1.41	0.010207945	0.020653013
55024	3087	BANK1	1.41	7.09E-06	2.35E-05
284071	4688	MEIOC	1.40	1.39E-12	8.12E-12
945	1807	CD33	1.40	0.018290709	0.035149181
1.08E+08	4984	LOC107986062	1.40	2.08E-09	9.66E-09
57194	4800	ATP10A	1.40	1.58E-37	2.91E-36
29995	1836	LMCD1	1.40	1.23E-42	2.68E-41
64167	5191	ERAP2	1.40	2.49E-95	1.81E-93
84217	1782	ZMYND12	1.40	0.019766453	0.03770374
56901	1445	NDUFA4L2	1.40	0.004164756	0.009080793
8470	5114	SORBS2	1.39	2.11E-25	2.45E-24
203447	8042	NRK	1.39	1.08E-72	4.99E-71
2635	3001	GBP3	1.39	3.15E-37	5.73E-36
9404	2295	LPXN	1.39	9.39E-15	6.40E-14
80004	4021	ESRP2	1.39	3.49E-16	2.58E-15
4758	2088	NEU1	1.39	2.01E-54	6.15E-53
147	2839	ADRA1B	1.39	2.65E-06	9.16E-06
3219	2711	HOXB9	1.39	6.08E-53	1.78E-51
118663	1877	BTBD16	1.39	0.018175765	0.034950972
84439	4419	HHIPL1	1.39	0.006880213	0.014395621
29015	2695	SLC43A3	1.39	1.91E-80	1.05E-78
64420	3051	SUSD1	1.39	2.12E-44	4.87E-43
55863	1167	TMEM126B	1.38	5.44E-42	1.16E-40
80128	2934	TRIM46	1.38	1.90E-09	8.88E-09
3698	3198	ITIH2	1.38	0.020531602	0.038987444
481	2212	ATP1B1	1.38	4.38E-84	2.57E-82
7145	9894	TNS1	1.38	1.67E-63	6.38E-62
84171	3639	LOXL4	1.38	6.16E-13	3.68E-12
11063	3938	SOX30	1.38	0.00166561	0.003898637
1E+08	1626	DPH3P1	1.38	1.60E-06	5.67E-06
200879	3411	LIPH	1.38	1.91E-10	9.64E-10
79931	6810	TNIP3	1.38	2.79E-46	6.76E-45
1.01E+08	1122	FXYD6-FXYD2	1.38	0.009051759	0.018499156
387911	1377	C1QTNF9B	1.38	0.021870774	0.041278937
6772	3928	STAT1	1.38	1.69E-100	1.36E-98
283209	8516	PGM2L1	1.38	7.45E-41	1.53E-39
11320	6890	MGAT4A	1.38	2.99E-16	2.22E-15
1.01E+08	700	LOC100996414	1.37	2.11E-07	8.18E-07
54596	3894	L1TD1	1.37	0.00346863	0.007664546
64127	4063	NOD2	1.37	0.015636762	0.030514441
134549	3461	SHROOM1	1.37	5.87E-36	1.02E-34
150365	3767	MEI1	1.37	0.002735858	0.006154036
10846	8194	PDE10A	1.37	4.77E-61	1.73E-59
26022	1798	TMEM98	1.37	5.13E-55	1.58E-53
219790	5468	RTKN2	1.37	2.30E-31	3.43E-30

9615	4524	GDA	1.37	1.45E-41	3.03E-40
8034	2634	SLC25A16	1.37	3.01E-32	4.61E-31
257144	3760	GCSAM	1.37	3.81E-05	0.000114797
9980	7338	DOPEY2	1.37	1.43E-42	3.11E-41
3570	5816	IL6R	1.37	1.69E-21	1.64E-20
10874	830	NMU	1.37	0.000868228	0.00213725
4606	3598	MYBPC2	1.37	0.001268119	0.003031339
54963	2334	UCKL1	1.36	1.53E-40	3.08E-39
10507	4742	SEMA4D	1.36	2.73E-81	1.51E-79
3909	6770	LAMA3	1.36	6.90E-80	3.75E-78
7923	1002	HSD17B8	1.36	1.24E-08	5.39E-08
221458	2677	KIF6	1.36	0.023830345	0.044617236
1E+08	1072	LOC100287036	1.36	0.023517138	0.044109839
80736	2517	SLC44A4	1.36	0.012933492	0.025610418
26503	3278	SLC17A5	1.36	1.51E-39	2.97E-38
85441	8336	HELZ2	1.36	4.58E-30	6.50E-29
7366	2264	UGT2B15	1.36	0.02391265	0.044747758
8345	425	HIST1H2BH	1.36	0.018345429	0.035246711
23704	3190	KCNE4	1.36	0.023869229	0.04467121
22875	4697	ENPP4	1.36	1.05E-24	1.17E-23
55359	2836	STYK1	1.36	5.21E-14	3.40E-13
400950	3721	C2orf91	1.36	0.002923845	0.006546229
353500	4535	BMP8A	1.36	6.83E-05	0.000198319
4739	4235	NEDD9	1.36	6.45E-60	2.26E-58
84561	3449	SLC12A8	1.35	2.52E-34	4.14E-33
6906	2384	SERPINA7	1.35	0.019134827	0.036624755
9235	1057	IL32	1.35	1.38E-62	5.18E-61
80032	1647	ZNF556	1.35	0.023562803	0.044181499
8483	4157	CILP	1.35	0.003349568	0.007417139
5066	3815	PAM	1.35	5.98E-118	6.33E-116
1102	2885	RCBTB2	1.35	8.59E-23	8.83E-22
1.08E+08	2785	LOC107986805	1.35	4.74E-19	4.08E-18
165140	1819	OXER1	1.35	0.022121845	0.041712931
54437	4523	SEMA5B	1.35	0.012807061	0.025396858
55137	10316	FIGN	1.35	2.36E-31	3.51E-30
83935	1866	NA	1.35	2.20E-08	9.40E-08
25854	2922	FAM149A	1.34	1.52E-26	1.86E-25
388886	1177	LRRC75B	1.34	5.75E-14	3.74E-13
51301	3436	GCNT4	1.34	0.001628461	0.003818223
8869	2381	ST3GAL5	1.34	1.40E-15	1.00E-14
8082	4385	SSPN	1.34	2.80E-14	1.86E-13
2204	2291	FCAR	1.34	0.026258245	0.048767272
23586	4759	DDX58	1.34	3.87E-34	6.33E-33
158471	5680	PRUNE2	1.34	9.97E-68	4.19E-66
8564	3708	KMO	1.34	0.000167169	0.000460006
28986	1485	MAGEH1	1.34	9.85E-15	6.71E-14
2170	1528	FABP3	1.34	0.000744281	0.001856053
84436	4000	ZNF528	1.34	1.33E-13	8.37E-13

1E+08	3803	LOC100130705	1.34	2.74E-07	1.05E-06
6095	11028	RORA	1.33	9.01E-06	2.94E-05
389177	1902	TMEM212	1.33	4.76E-29	6.48E-28
5167	7086	ENPP1	1.33	7.23E-26	8.55E-25
137994	2324	LETM2	1.33	2.79E-10	1.38E-09
646019	2252	CBY3	1.33	0.017654542	0.034077774
23213	5047	SULF1	1.33	0.006110193	0.012922975
8988	787	HSPB3	1.33	0.020800676	0.039443586
8742	1407	TNFSF12	1.33	9.61E-14	6.13E-13
2892	2519	GRIA3	1.33	0.002887297	0.006469293
10307	2155	APBB3	1.33	2.24E-26	2.71E-25
653149	4432	NBPF6	1.33	0.026272442	0.048783441
23682	1488	RAB38	1.33	1.18E-15	8.53E-15
84293	1927	FAM213A	1.33	6.10E-49	1.58E-47
11274	2043	USP18	1.33	1.60E-13	1.00E-12
2822	5609	GPLD1	1.33	0.000950375	0.002327214
9729	8124	KIAA0408	1.32	0.020692438	0.039259288
645426	1325	TMEM191C	1.32	0.006187118	0.013076331
7498	5720	XDH	1.32	7.10E-14	4.58E-13
80310	3993	PDGFD	1.32	7.51E-14	4.83E-13
4988	15427	OPRM1	1.32	0.013328033	0.026330053
3638	2944	INSIG1	1.32	3.77E-47	9.30E-46
10475	6371	TRIM38	1.32	3.24E-54	9.84E-53
26137	26951	ZBTB20	1.32	2.91E-52	8.33E-51
57578	8607	UNC79	1.32	0.020996564	0.039764117
760	1666	CA2	1.32	5.64E-22	5.58E-21
57730	5014	ANKRD36B	1.31	4.35E-23	4.53E-22
340120	3729	ANKRD34B	1.31	1.18E-20	1.10E-19
140700	2237	SAMD10	1.31	1.82E-13	1.13E-12
8671	7682	SLC4A4	1.31	7.20E-27	8.94E-26
389602	2050	LOC389602	1.31	0.020013001	0.038116733
6383	3327	SDC2	1.31	2.88E-71	1.30E-69
57562	6977	CEP126	1.31	5.86E-17	4.57E-16
166336	8145	PRICKLE2	1.31	1.18E-41	2.48E-40
22806	9092	IKZF3	1.31	2.33E-06	8.12E-06
114299	9500	PALM2	1.31	5.63E-08	2.32E-07
79608	2464	RIC3	1.31	8.20E-12	4.55E-11
151126	2710	ZNF385B	1.31	3.28E-08	1.38E-07
55083	11566	KIF26B	1.31	1.10E-19	9.79E-19
3375	1923	IAPP	1.31	0.022745739	0.042762279
23554	2446	TSPAN12	1.31	1.35E-25	1.58E-24
1.05E+08	10052	LOC105376204	1.31	6.40E-07	2.36E-06
5365	6211	PLXNB3	1.31	5.55E-13	3.32E-12
5800	7274	PTPRO	1.31	0.010122385	0.020489238
80263	3683	TRIM45	1.31	1.66E-20	1.54E-19
8218	4375	CLTCL1	1.31	1.33E-25	1.56E-24
55001	3645	TTC22	1.30	6.06E-07	2.24E-06
653567	5454	TMEM236	1.30	0.012511376	0.024846555

80737	4348	VWA7	1.30	1.70E-16	1.28E-15
1E+08	2626	CTAGE4	1.30	3.60E-09	1.64E-08
79086	1343	SMIM7	1.30	2.73E-54	8.33E-53
3908	9696	LAMA2	1.30	0.000155128	0.000428532
622	3824	BDH1	1.30	2.35E-22	2.37E-21
130872	4046	AHSA2P	1.30	8.29E-34	1.34E-32
7482	2689	WNT2B	1.30	1.64E-10	8.33E-10
1.05E+08	2492	LOC105379547	1.30	1.98E-05	6.20E-05
2121	6299	EVC	1.30	2.52E-52	7.24E-51
132299	789	OCIAD2	1.30	2.20E-30	3.17E-29
285220	3456	EPHA6	1.30	1.15E-07	4.57E-07
257019	4894	FRMD3	1.30	8.00E-06	2.63E-05
23138	4676	N4BP3	1.29	7.97E-19	6.80E-18
1.08E+08	3377	LOC107984745	1.29	0.001825316	0.004242279
26999	6427	CYFIP2	1.29	2.97E-28	3.91E-27
374407	1344	DNAJB13	1.29	6.76E-06	2.25E-05
115273	1896	RAB42	1.29	2.17E-21	2.09E-20
5874	6168	RAB27B	1.29	5.08E-23	5.28E-22
8992	894	ATP6V0E1	1.29	2.44E-64	9.56E-63
28970	3058	C11orf54	1.29	4.63E-39	9.01E-38
83641	3238	FAM107B	1.29	1.20E-56	3.88E-55
1.03E+08	1156	LOC102724770	1.29	5.84E-14	3.80E-13
1717	2668	DHCR7	1.29	4.58E-42	9.78E-41
196403	2441	DTX3	1.29	3.80E-29	5.20E-28
282679	2153	AQP11	1.29	0.000341404	0.000897809
3112	1388	HLA-DOB	1.29	0.001389128	0.003299766
80258	3269	EFHC2	1.29	0.02477583	0.046231563
54540	3280	FAM193B	1.29	1.68E-36	2.99E-35
388559	4005	ZNF888	1.29	7.61E-27	9.44E-26
51310	2318	SLC22A17	1.28	2.19E-50	5.90E-49
1.08E+08	3008	LOC107984203	1.28	1.94E-07	7.56E-07
57393	1580	CLTRN	1.28	0.000155966	0.000430713
79668	6000	PARP8	1.28	1.73E-35	2.97E-34
126695	1826	KDF1	1.28	0.000304144	0.00080513
2950	986	GSTP1	1.28	2.41E-62	8.98E-61
55748	2667	CNDP2	1.28	1.03E-84	6.14E-83
80781	5445	COL18A1	1.28	1.26E-104	1.08E-102
158046	1332	NXNL2	1.27	4.27E-08	1.77E-07
10379	1699	IRF9	1.27	7.13E-35	1.20E-33
3955	2163	LFNG	1.27	5.68E-42	1.21E-40
1.01E+08	5280	GVQW3	1.27	1.92E-11	1.04E-10
11226	4628	GALNT6	1.27	6.99E-28	9.06E-27
26154	8416	ABCA12	1.27	2.37E-22	2.38E-21
8301	3670	PICALM	1.27	5.73E-90	3.72E-88
6277	650	S100A6	1.27	1.62E-52	4.68E-51
2166	2105	FAAH	1.27	5.88E-10	2.85E-09
1368	6667	CPM	1.27	1.43E-59	4.96E-58
51560	5561	RAB6B	1.27	9.78E-20	8.76E-19

65999	2043	LRRC61	1.27	1.36E-25	1.59E-24
117177	7302	RAB3IP	1.27	9.53E-45	2.22E-43
55423	1194	SIRPG	1.27	0.018201568	0.034989231
132430	4944	PABPC4L	1.26	1.82E-13	1.14E-12
57489	4537	ODF2L	1.26	1.54E-31	2.32E-30
6920	1761	TCEA3	1.26	1.31E-19	1.17E-18
286006	1710	LSMEM1	1.26	0.000781197	0.001939118
282890	3077	ZNF311	1.26	1.02E-09	4.84E-09
56950	1689	SMYD2	1.26	3.40E-39	6.64E-38
7169	1207	TPM2	1.26	8.51E-60	2.98E-58
1E+08	5635	CCDC180	1.26	3.25E-09	1.49E-08
163255	3237	ZNF540	1.26	0.00379261	0.008331737
2707	1925	GJB3	1.26	2.57E-10	1.28E-09
83733	2090	SLC25A18	1.26	0.009332406	0.019028942
7130	1439	TNFAIP6	1.26	0.011720822	0.023386364
203522	3851	INTS6L	1.26	1.61E-24	1.79E-23
3162	1606	HMOX1	1.26	4.02E-37	7.28E-36
51390	1655	AIG1	1.26	3.67E-61	1.33E-59
51168	11876	MYO15A	1.25	0.017276286	0.033412673
1.03E+08	1369	LOC102724993	1.25	3.04E-25	3.49E-24
83851	9410	SYT16	1.25	2.20E-08	9.38E-08
200035	1486	NUDT17	1.25	6.25E-06	2.08E-05
6948	2092	TCN2	1.25	3.11E-51	8.58E-50
1.02E+08	3480	LOC101928212	1.25	0.002961953	0.006623203
492311	2900	IGIP	1.25	4.34E-13	2.62E-12
347736	2763	NME9	1.25	0.000383771	0.001002034
1E+08	582	FAM86KP	1.25	0.025665937	0.04775208
219293	3859	ATAD3C	1.25	1.18E-08	5.16E-08
150094	4706	SIK1	1.25	2.48E-08	1.05E-07
84258	2813	SYT3	1.25	0.000842899	0.00207922
5002	1555	SLC22A18	1.25	1.33E-21	1.30E-20
1.01E+08	1800	LOC101060341	1.24	0.000185783	0.000507456
1.01E+08	3807	NBPF19	1.24	2.66E-35	4.53E-34
5764	1551	PTN	1.24	1.34E-37	2.47E-36
10402	3378	ST3GAL6	1.24	1.64E-14	1.10E-13
55607	9366	PPP1R9A	1.24	1.64E-22	1.66E-21
534	1587	ATP6V1G2	1.24	7.30E-08	2.97E-07
79946	1469	C10orf95	1.24	0.002972567	0.006646101
63951	2302	DMRTA1	1.24	1.67E-06	5.90E-06
2027	1696	ENO3	1.24	9.78E-12	5.42E-11
55277	1648	FGGY	1.24	4.35E-09	1.97E-08
54625	7707	PARP14	1.24	2.99E-48	7.55E-47
2744	4635	GLS	1.24	1.27E-73	6.04E-72
80144	15643	FRAS1	1.24	9.86E-20	8.82E-19
284406	5564	ZFP82	1.24	0.001256529	0.003007275
143872	6863	ARHGAP42	1.24	3.57E-30	5.08E-29
23224	5227	SYNE2	1.24	2.12E-56	6.81E-55
90990	3274	KIFC2	1.24	6.17E-59	2.10E-57

127255	1162	LRRIQ3	1.24	0.017494451	0.033801806
51166	2195	AADAT	1.24	9.56E-21	8.99E-20
441273	4899	SPDYE2	1.23	5.87E-10	2.84E-09
4324	4268	MMP15	1.23	3.10E-38	5.87E-37
1636	3806	ACE	1.23	0.008487893	0.0174451
629	2646	CFB	1.23	1.58E-17	1.27E-16
54675	2673	CRLS1	1.23	9.15E-56	2.89E-54
613037	4270	NPIPB13	1.23	1.55E-05	4.93E-05
5918	1535	RARRES1	1.23	6.46E-07	2.38E-06
23321	6303	TRIM2	1.23	1.74E-45	4.13E-44
114780	1769	PKD1L2	1.23	0.020977757	0.039732735
1958	3136	EGR1	1.23	5.60E-09	2.51E-08
4299	8650	AFF1	1.22	1.54E-47	3.83E-46
246721	1987	POLR2J2	1.22	2.66E-13	1.64E-12
10584	3096	COLEC10	1.22	0.021798764	0.041151767
7280	1622	TUBB2A	1.22	7.64E-34	1.24E-32
9175	9106	MAP3K13	1.22	5.35E-17	4.17E-16
280	2253	AMY2B	1.22	3.50E-13	2.14E-12
23564	1394	DDAH2	1.22	4.68E-39	9.10E-38
29103	2792	DNAJC15	1.22	1.31E-49	3.46E-48
642658	2092	SCX	1.22	7.84E-14	5.03E-13
1010	3714	CDH12	1.22	0.01534096	0.029976705
1.08E+08	2573	LOC107987276	1.22	0.004659739	0.010091935
9723	6615	SEMA3E	1.22	4.65E-07	1.74E-06
283130	2828	SLC25A45	1.22	2.19E-12	1.27E-11
89832	5439	CHRFAM7A	1.22	1.76E-16	1.33E-15
2047	3910	EPHB1	1.22	1.27E-10	6.48E-10
23491	3182	CES3	1.22	8.11E-14	5.19E-13
56895	7830	AGPAT4	1.22	1.70E-25	1.97E-24
201163	3675	FLCN	1.21	1.56E-30	2.25E-29
10723	5315	SLC12A7	1.21	9.36E-39	1.80E-37
3430	1243	IFI35	1.21	1.74E-28	2.32E-27
2256	2541	FGF11	1.21	5.27E-08	2.17E-07
494513	2083	PJVK	1.21	6.23E-07	2.30E-06
2644	713	GCHFR	1.21	1.87E-12	1.09E-11
57001	2068	SDHAF3	1.21	4.60E-12	2.60E-11
1.08E+08	2973	LOC107986800	1.21	6.69E-11	3.51E-10
9699	4871	RIMS2	1.21	1.24E-28	1.65E-27
158747	2768	MOSPD2	1.21	7.36E-25	8.30E-24
199745	1825	THAP8	1.21	2.38E-10	1.19E-09
5140	4383	PDE3B	1.21	0.020146108	0.038333266
90592	2897	ZNF700	1.21	3.60E-26	4.32E-25
728	2331	C5AR1	1.21	1.36E-06	4.83E-06
388849	1913	CCDC188	1.21	0.000646296	0.00162562
54914	5682	FOCAD	1.21	1.02E-29	1.43E-28
126432	3056	RINL	1.21	7.83E-09	3.48E-08
57834	2973	CYP4F11	1.21	3.94E-08	1.64E-07
342918	765	C19orf81	1.21	2.62E-05	8.06E-05

25837	1679	RAB26	1.21	4.91E-13	2.95E-12
2153	9179	F5	1.20	0.008901921	0.018218076
1E+08	3748	FAM157B	1.20	9.11E-05	0.000260234
6447	1241	SCG5	1.20	0.002531372	0.005736184
1.05E+08	1080	LOC105376752	1.20	0.000798182	0.001976598
26140	3662	TTLL3	1.20	4.16E-08	1.73E-07
64582	8231	GPR135	1.20	1.66E-09	7.79E-09
4322	2735	MMP13	1.20	3.43E-11	1.84E-10
118881	1041	COMTD1	1.20	3.21E-15	2.25E-14
761	1753	CA3	1.20	0.00146334	0.003464441
93145	1804	OLFM2	1.20	0.007906011	0.016343689
96459	6599	FNIP1	1.20	3.69E-37	6.69E-36
4935	1711	GPR143	1.20	7.97E-08	3.22E-07
26472	1038	PPP1R14B	1.20	7.74E-45	1.81E-43
55738	3292	ARFGAP1	1.20	1.38E-51	3.85E-50
8651	1216	SOCS1	1.19	7.24E-11	3.79E-10
7710	6914	ZNF154	1.19	0.004173681	0.009095788
93099	1111	DMKN	1.19	2.16E-31	3.23E-30
3176	2837	HNMT	1.19	3.56E-21	3.40E-20
22798	2771	LAMB4	1.19	0.013282187	0.026245319
8676	4224	STX11	1.19	2.88E-08	1.22E-07
1E+08	7650	ZNF737	1.19	1.96E-05	6.13E-05
30011	3066	SH3KBP1	1.19	1.59E-38	3.03E-37
91523	2184	PCED1B	1.19	6.78E-06	2.25E-05
1E+08	1567	FAM47E	1.19	0.003284295	0.007287132
6609	2485	SMPD1	1.19	2.30E-42	4.98E-41
55349	5259	CHDH	1.19	9.70E-20	8.70E-19
375248	5522	ANKRD36	1.18	2.31E-22	2.33E-21
7006	3724	TEC	1.18	5.15E-12	2.90E-11
5243	4718	ABCB1	1.18	7.82E-35	1.31E-33
79924	4045	ADM2	1.18	1.14E-13	7.26E-13
10110	1994	SGK2	1.18	1.12E-10	5.76E-10
1.08E+08	1624	LOC107984648	1.18	0.00029402	0.000780073
144402	3692	CPNE8	1.18	1.54E-24	1.71E-23
5593	3656	PRKG2	1.18	0.002302939	0.005253385
79094	1639	CHAC1	1.18	3.70E-15	2.58E-14
55769	2840	ZNF83	1.18	4.92E-29	6.69E-28
285676	3481	ZNF454	1.18	6.80E-05	0.000197657
87769	2935	GGACT	1.18	1.17E-06	4.20E-06
5000	6512	ORC4	1.18	4.62E-38	8.70E-37
9866	9519	TRIM66	1.18	6.57E-11	3.45E-10
141	3431	ADPRH	1.18	1.07E-13	6.82E-13
128178	2891	EDARADD	1.18	0.008209319	0.016909712
23231	4488	SEL1L3	1.18	1.62E-58	5.49E-57
747	5678	DAGLA	1.18	4.62E-28	6.05E-27
404220	1914	C6orf201	1.18	0.025264992	0.047080061
10251	9106	SPRY3	1.17	1.26E-05	4.04E-05
8507	5564	ENC1	1.17	4.62E-32	7.05E-31

10007	2334	GNPDA1	1.17	2.85E-45	6.75E-44
10287	1553	RGS19	1.17	7.44E-14	4.79E-13
146779	1545	EFCAB3	1.17	0.015873468	0.030925415
26548	1453	ITGB1BP2	1.17	0.007595129	0.015756013
10493	2758	VAT1	1.17	2.60E-58	8.74E-57
79758	1392	DHRS12	1.17	2.40E-10	1.20E-09
3897	5021	L1CAM	1.17	1.00E-48	2.57E-47
8553	3061	BHLHE40	1.17	7.86E-47	1.93E-45
25894	4590	PLEKHG4	1.17	1.32E-34	2.19E-33
79669	2284	C3orf52	1.17	4.08E-13	2.47E-12
1.01E+08	3190	LOC101060022	1.17	3.19E-06	1.09E-05
306	1634	ANXA3	1.17	2.76E-51	7.63E-50
27063	1994	ANKRD1	1.17	2.72E-23	2.86E-22
440270	6156	GOLGA8B	1.17	8.00E-09	3.54E-08
3786	10665	KCNQ3	1.17	3.44E-10	1.70E-09
91351	5421	DDX60L	1.16	9.38E-23	9.62E-22
7436	5211	VLDLR	1.16	6.24E-37	1.12E-35
55191	2453	NADSYN1	1.16	2.00E-39	3.93E-38
2101	2329	ESRRA	1.16	1.37E-26	1.68E-25
150709	4023	ANKAR	1.16	0.000411437	0.001069399
728642	3202	CDK11A	1.16	3.12E-30	4.45E-29
22902	3824	RUFY3	1.16	8.60E-27	1.06E-25
1.08E+08	5108	LOC107987163	1.16	0.000169554	0.000466135
150946	3938	GAREM2	1.16	1.46E-17	1.17E-16
81932	1605	HDHD3	1.16	1.59E-17	1.27E-16
4897	6357	NRCAM	1.16	3.47E-43	7.71E-42
3767	3378	KCNJ11	1.16	0.010963798	0.022036737
161247	1064	FITM1	1.16	0.022892288	0.043010486
81493	3465	SYNC	1.16	1.02E-30	1.48E-29
25890	5163	ABI3BP	1.16	2.40E-12	1.38E-11
9914	4393	ATP2C2	1.16	0.003341412	0.007400924
10457	2778	GPNMB	1.16	1.69E-21	1.64E-20
56243	7020	KIAA1217	1.15	3.29E-41	6.85E-40
6515	3938	SLC2A3	1.15	6.40E-25	7.24E-24
7407	3728	VARS	1.15	1.58E-50	4.30E-49
9026	4431	HIP1R	1.15	2.16E-45	5.11E-44
10763	5591	NES	1.15	3.51E-08	1.47E-07
199221	3271	DZIP1L	1.15	2.11E-20	1.94E-19
2852	2691	GPER1	1.15	0.000356904	0.000935459
134429	4092	STARD4	1.15	1.16E-36	2.07E-35
4047	4222	LSS	1.15	9.24E-40	1.83E-38
81856	4412	ZNF611	1.15	8.92E-16	6.48E-15
51765	3210	STK26	1.15	9.64E-18	7.81E-17
643911	674	CRNDE	1.15	2.70E-07	1.03E-06
8406	1908	SRPX	1.15	1.05E-19	9.41E-19
27185	6456	DISC1	1.15	1.01E-08	4.46E-08
643680	1161	MS4A4E	1.15	4.45E-11	2.36E-10
80243	10122	PREX2	1.15	1.67E-23	1.78E-22

389084	1589	SNORC	1.15	0.005825515	0.01237243
1.08E+08	3326	LOC107986987	1.15	0.001445128	0.003424974
387849	1139	REP15	1.14	0.023621346	0.044267912
4636	1350	MYL5	1.14	1.31E-13	8.25E-13
120227	3225	CYP2R1	1.14	5.40E-14	3.53E-13
347902	3470	AMIGO2	1.14	4.55E-37	8.22E-36
114899	3644	C1QTNF3	1.14	7.39E-06	2.44E-05
1.08E+08	1448	LOC107984156	1.14	0.000119366	0.000335055
5436	852	POLR2G	1.14	2.65E-40	5.33E-39
1290	6770	COL5A2	1.14	6.07E-08	2.49E-07
388403	5242	YPEL2	1.14	7.39E-20	6.65E-19
84830	2005	ADTRP	1.14	0.009360016	0.019083048
10045	2061	SH2D3A	1.14	7.52E-08	3.05E-07
6581	3372	SLC22A3	1.14	6.68E-06	2.22E-05
9734	4854	HDAC9	1.14	4.81E-16	3.54E-15
4864	4839	NPC1	1.14	7.40E-52	2.09E-50
10962	2180	MLLT11	1.14	4.40E-25	5.03E-24
8310	2909	ACOX3	1.14	1.69E-17	1.35E-16
51761	9289	ATP8A2	1.14	3.47E-05	0.0001049
166785	5800	MMAA	1.14	6.19E-12	3.47E-11
58157	1885	NGB	1.14	0.013794358	0.027193848
1962	3919	EHHADH	1.14	1.47E-21	1.42E-20
9636	685	ISG15	1.14	3.42E-13	2.08E-12
114823	5503	LENG8	1.14	1.47E-06	5.23E-06
55902	2944	ACSS2	1.14	2.33E-53	6.91E-52
23767	4208	FLRT3	1.14	6.41E-07	2.36E-06
132321	1645	C4orf33	1.13	6.67E-17	5.18E-16
50853	2889	VILL	1.13	2.37E-06	8.25E-06
23228	4155	PLCL2	1.13	1.89E-05	5.93E-05
368	1396	ABCC6	1.13	3.00E-18	2.50E-17
254552	771	NUDT8	1.13	1.76E-09	8.25E-09
84125	4110	LRRIQ1	1.13	3.44E-09	1.57E-08
2100	4228	ESR2	1.13	0.000572889	0.001454155
9788	4654	MTSS1	1.13	1.08E-36	1.92E-35
7168	1889	TPM1	1.13	5.92E-54	1.78E-52
5155	3113	PDGFB	1.13	9.09E-22	8.92E-21
6296	2146	ACSM3	1.12	5.61E-06	1.88E-05
284040	2515	CDRT4	1.12	0.020773815	0.039401061
112849	2504	L3HYPDH	1.12	1.27E-17	1.03E-16
340277	1384	FAM221A	1.12	1.25E-06	4.48E-06
729220	2100	FLJ45513	1.12	0.012153268	0.024189455
57538	10915	ALPK3	1.12	0.003123064	0.00696156
26973	3211	CHORDC1	1.12	1.04E-26	1.28E-25
339977	5373	LRRC66	1.12	0.010734022	0.021609121
4218	2163	RAB8A	1.12	3.04E-62	1.13E-60
1.08E+08	2575	LOC107987457	1.12	3.23E-05	9.80E-05
57473	5522	ZNF512B	1.12	1.06E-26	1.30E-25
2526	6063	FUT4	1.12	2.75E-19	2.41E-18

8313	4045	AXIN2	1.12	4.48E-12	2.53E-11
553	1773	AVPR1B	1.12	0.02136632	0.040404084
253461	6904	ZBTB38	1.12	7.70E-45	1.80E-43
3824	3160	KLRD1	1.12	1.72E-05	5.44E-05
83707	1003	TRPT1	1.12	6.59E-24	7.12E-23
91624	2826	NEXN	1.12	2.93E-30	4.19E-29
408186	4121	OVOS	1.11	0.005467589	0.011670846
4439	2783	MSH5	1.11	1.92E-13	1.20E-12
29933	3788	GPR132	1.11	0.000128907	0.00035971
1513	1825	CTSK	1.11	4.30E-07	1.62E-06
1350	448	COX7C	1.11	3.99E-38	7.55E-37
11282	2538	MGAT4B	1.11	1.32E-60	4.72E-59
79098	5392	C1orf116	1.11	6.26E-10	3.02E-09
55049	837	REX1BD	1.11	1.30E-15	9.35E-15
154	2058	ADRB2	1.11	1.86E-06	6.55E-06
79644	2722	SRD5A3	1.11	3.74E-19	3.24E-18
5031	2245	P2RY6	1.11	1.66E-12	9.70E-12
56245	3976	C21orf62	1.11	0.000984655	0.002403207
2026	2423	ENO2	1.10	2.19E-66	8.94E-65
728239	2613	MAGED4	1.10	1.82E-18	1.53E-17
55605	6492	KIF21A	1.10	1.64E-30	2.36E-29
2354	3668	FOSB	1.10	1.31E-05	4.20E-05
1.03E+08	4724	SIK1B	1.10	5.42E-43	1.20E-41
4126	3050	MANBA	1.10	2.32E-29	3.21E-28
80146	1997	UXS1	1.10	4.33E-50	1.16E-48
11030	1867	RBPMS	1.10	6.80E-31	9.96E-30
56624	3213	ASAH2	1.10	0.000316172	0.000834856
378884	2134	NHLRC1	1.10	4.55E-05	0.000135494
374739	1701	TEPP	1.10	0.004443721	0.009648789
283	1167	ANG	1.10	0.000211302	0.000571709
10161	2512	LPAR6	1.10	7.03E-11	3.68E-10
255928	11318	SYT14	1.10	1.38E-14	9.33E-14
90342	5868	FER1L5	1.10	0.001124036	0.002715783
220992	3063	ZNF485	1.10	6.28E-05	0.000183291
50855	1270	PARD6A	1.09	6.70E-06	2.23E-05
92105	3335	INTS4	1.09	6.37E-26	7.55E-25
9635	4000	CLCA2	1.09	2.14E-07	8.30E-07
166348	5533	KBTBD12	1.09	0.000474498	0.001219921
90019	1535	SYT8	1.09	0.000732852	0.001828578
26240	1673	FAM50B	1.09	2.49E-07	9.57E-07
3613	1521	IMPA2	1.09	9.67E-29	1.30E-27
401251	917	SAPCD1	1.09	7.59E-06	2.50E-05
28966	2211	SNX24	1.09	6.20E-27	7.72E-26
6274	738	S100A3	1.09	0.000242944	0.000652255
9246	1291	UBE2L6	1.09	5.03E-33	7.91E-32
400745	3693	SH2D5	1.09	1.09E-07	4.36E-07
10381	1794	TUBB3	1.08	1.48E-28	1.97E-27
3417	2403	IDH1	1.08	3.12E-35	5.30E-34

58513	3105	EPS15L1	1.08	8.60E-44	1.95E-42
9209	2651	LRRFIP2	1.08	2.18E-49	5.75E-48
401720	4982	FIGNL2	1.08	0.007022917	0.014676718
1E+08	2299	CD24	1.08	3.91E-54	1.18E-52
10924	1910	SMPDL3A	1.08	3.77E-10	1.85E-09
23649	3006	POLA2	1.08	1.38E-47	3.44E-46
23263	5174	MCF2L	1.08	1.10E-08	4.83E-08
1946	5681	EFNA5	1.08	2.31E-56	7.44E-55
6520	1953	SLC3A2	1.08	3.22E-47	7.98E-46
54986	4002	ULK4	1.08	1.76E-10	8.89E-10
80230	2277	RUFY1	1.08	2.20E-38	4.20E-37
11330	3342	CTRC	1.08	0.013024151	0.025772704
22881	4990	ANKRD6	1.08	1.87E-12	1.09E-11
390927	7179	ZNF793	1.08	9.84E-08	3.94E-07
1.03E+08	2412	TBC1D3E	1.07	1.47E-15	1.05E-14
10263	1269	CDK2AP2	1.07	5.52E-25	6.29E-24
51454	3407	GULP1	1.07	2.67E-23	2.80E-22
127845	883	GOLT1A	1.07	0.022502139	0.042349116
84327	1437	ZBED3	1.07	1.19E-06	4.26E-06
3569	1376	IL6	1.07	4.89E-17	3.82E-16
122509	838	IFI27L1	1.07	1.89E-17	1.51E-16
3983	6919	ABLIM1	1.07	2.15E-48	5.45E-47
8536	1484	CAMK1	1.07	1.03E-26	1.27E-25
4883	6569	NPR3	1.07	1.28E-15	9.18E-15
6242	2236	RTKN	1.07	4.14E-26	4.95E-25
219970	1488	GLYATL2	1.07	2.93E-07	1.12E-06
79191	2546	IRX3	1.07	4.42E-21	4.20E-20
4908	1280	NTF3	1.07	0.000638337	0.001607421
2628	2742	GATM	1.07	0.015545276	0.030349244
22854	4535	NTNG1	1.06	1.54E-30	2.22E-29
387882	1383	C12orf75	1.06	1.36E-29	1.89E-28
146712	2119	B3GNTL1	1.06	5.98E-07	2.22E-06
23414	4029	ZFPM2	1.06	0.007253147	0.015104773
29121	5249	CLEC2D	1.06	7.69E-14	4.94E-13
4026	17866	LPP	1.06	4.72E-67	1.96E-65
84619	1926	ZGPAT	1.06	5.53E-17	4.31E-16
3574	2293	IL7	1.06	0.000235289	0.000632755
65084	4152	TMEM135	1.06	4.33E-21	4.12E-20
81557	2582	MAGED4B	1.06	4.32E-26	5.16E-25
84105	2395	PCBD2	1.06	2.60E-11	1.40E-10
79612	3862	NAA16	1.06	2.25E-20	2.07E-19
6480	4235	ST6GAL1	1.06	2.38E-23	2.50E-22
84109	2370	QRFPR	1.06	4.65E-06	1.57E-05
54209	882	TREM2	1.06	0.024622905	0.045965515
57053	2349	CHRNA10	1.06	0.002870278	0.006435218
134637	8505	ADAT2	1.06	2.74E-14	1.82E-13
338376	1501	IFNE	1.06	0.012817693	0.025415107
6374	2505	CXCL5	1.06	8.37E-25	9.40E-24

6231	699	RPS26	1.06	1.87E-46	4.57E-45
284266	1523	SIGLEC15	1.06	0.000533087	0.001359536
1612	5587	DAPK1	1.06	1.66E-46	4.06E-45
4661	5550	MYT1	1.06	0.01856861	0.035652374
55796	9711	MBNL3	1.06	9.16E-17	7.04E-16
283537	3262	SLC46A3	1.06	1.66E-07	6.52E-07
1.01E+08	2370	TBC1D3L	1.06	1.35E-20	1.26E-19
206412	3357	C6orf163	1.06	0.000144702	0.000402043
7084	4789	TK2	1.05	1.74E-26	2.11E-25
6648	4069	SOD2	1.05	6.19E-38	1.16E-36
160851	16923	DGKH	1.05	1.79E-18	1.50E-17
89884	5140	LHX4	1.05	0.000225643	0.000608104
9764	7217	KIAA0513	1.05	7.31E-21	6.89E-20
7274	3814	TTPA	1.05	6.35E-07	2.34E-06
387712	4489	ENO4	1.05	0.004835149	0.010432413
58191	2259	CXCL16	1.05	3.38E-23	3.54E-22
83992	5979	CTTNBP2	1.05	3.84E-16	2.84E-15
81029	2184	WNT5B	1.05	2.76E-09	1.27E-08
1843	2040	DUSP1	1.05	6.69E-43	1.47E-41
57698	4138	SHTN1	1.05	2.75E-30	3.93E-29
834	1766	CASP1	1.05	1.10E-16	8.42E-16
285464	4467	CRIPAK	1.05	4.47E-11	2.38E-10
197259	2680	MLKL	1.05	1.97E-13	1.23E-12
23034	6477	SAMD4A	1.05	1.14E-30	1.66E-29
23461	8630	ABCA5	1.04	2.17E-20	2.00E-19
641371	1099	ACOT1	1.04	2.50E-05	7.70E-05
22898	4625	DENND3	1.04	8.05E-26	9.51E-25
3491	2295	CYR61	1.04	1.53E-63	5.89E-62
55715	2747	DOK4	1.04	4.66E-29	6.36E-28
57577	3956	CCDC191	1.04	3.04E-09	1.39E-08
9900	3087	SV2A	1.04	3.39E-30	4.82E-29
1.08E+08	1471	LOC107983998	1.04	7.44E-06	2.45E-05
84286	1923	TMEM175	1.04	4.13E-21	3.94E-20
222236	4156	NAPEPLD	1.04	3.40E-16	2.52E-15
653073	4170	GOLGA8J	1.04	0.004826347	0.010417862
1.08E+08	915	LOC107986794	1.04	0.000850129	0.0020956
6775	2835	STAT4	1.04	1.34E-11	7.33E-11
26229	1694	B3GAT3	1.04	2.89E-38	5.48E-37
192668	2719	CYS1	1.04	1.21E-21	1.18E-20
29945	2034	ANAPC4	1.04	1.52E-24	1.69E-23
9781	5428	RNF144A	1.04	2.64E-10	1.31E-09
1.08E+08	1043	LOC107985355	1.04	0.001136205	0.002742947
1528	859	CYB5A	1.04	1.89E-27	2.40E-26
5563	9444	PRKAA2	1.04	9.77E-20	8.75E-19
8997	5107	KALRN	1.04	1.12E-10	5.78E-10
3431	2629	SP110	1.04	1.81E-27	2.30E-26
2668	3571	GDNF	1.03	1.93E-05	6.05E-05
727800	1095	RNF208	1.03	8.94E-07	3.25E-06

7050	1590	TGIF1	1.03	2.27E-33	3.62E-32
341676	2963	NEK5	1.03	0.020650308	0.039187725
5122	5166	PCSK1	1.03	0.011148726	0.022373001
23254	3731	KAZN	1.03	6.42E-34	1.04E-32
84334	1790	APOPT1	1.03	2.52E-21	2.42E-20
54923	1385	LIME1	1.03	2.26E-07	8.74E-07
201799	3230	TMEM154	1.03	3.54E-12	2.01E-11
221491	988	SMIM29	1.03	2.29E-08	9.76E-08
9317	3483	PTER	1.03	8.39E-22	8.26E-21
1.02E+08	1731	LOC101929400	1.03	0.002166113	0.004964207
2912	2534	GRM2	1.03	0.017950495	0.034585165
1.01E+08	2022	PTGES3L	1.03	0.000867141	0.002135166
54951	1485	COMMD8	1.03	2.09E-12	1.21E-11
8566	6828	PDXK	1.03	8.37E-54	2.51E-52
5872	1162	RAB13	1.03	4.16E-41	8.61E-40
57477	8955	SHROOM4	1.03	1.90E-11	1.03E-10
7204	10327	TRIO	1.03	7.59E-46	1.81E-44
4124	7090	MAN2A1	1.02	9.39E-40	1.86E-38
27089	1585	UQCRQ	1.02	7.39E-33	1.16E-31
79841	3099	AGBL2	1.02	0.000722533	0.001804605
84033	22729	OBSCN	1.02	2.40E-17	1.91E-16
79632	3912	FAM184A	1.02	1.31E-05	4.18E-05
10588	2342	MTHFS	1.02	2.01E-10	1.01E-09
9884	4688	LRRC37A	1.02	1.45E-12	8.48E-12
357	5380	SHROOM2	1.02	4.22E-21	4.01E-20
9168	482	TMSB10	1.02	8.75E-35	1.46E-33
3426	2201	CFI	1.02	4.20E-39	8.17E-38
1.08E+08	660	LOC107984841	1.02	0.000107508	0.000304075
80164	4518	PRR36	1.02	9.75E-07	3.53E-06
3690	4894	ITGB3	1.02	4.68E-29	6.38E-28
728734	1626	NPIPB8	1.02	1.26E-08	5.48E-08
3171	2046	FOXA3	1.02	0.000649328	0.001632553
6307	2218	MSMO1	1.01	1.77E-21	1.71E-20
9170	1845	LPAR2	1.01	6.37E-14	4.12E-13
3756	7978	KCNH1	1.01	5.56E-10	2.70E-09
285989	3857	ZNF789	1.01	3.57E-13	2.17E-12
8649	4280	LAMTOR3	1.01	7.07E-28	9.16E-27
29911	3276	HOOK2	1.01	7.72E-14	4.95E-13
7171	2683	TPM4	1.01	1.47E-49	3.90E-48
92591	2139	ASB16	1.01	4.21E-05	0.000126154
117608	2200	ZNF354B	1.01	1.43E-13	9.04E-13
3992	4432	FADS1	1.01	3.04E-59	1.05E-57
10057	4687	ABCC5	1.01	9.96E-25	1.12E-23
80023	2132	NRSN2	1.01	5.74E-23	5.95E-22
4199	3428	ME1	1.01	9.49E-19	8.07E-18
79710	3749	MORC4	1.01	1.91E-30	2.75E-29
11098	3953	PRSS23	1.01	1.60E-48	4.09E-47
284656	2152	EPHA10	1.01	0.010599048	0.02136645

400916	718	CHCHD10	1.01	1.09E-14	7.41E-14
80206	5088	FHOD3	1.01	1.26E-50	3.43E-49
85462	6499	FHDC1	1.01	3.07E-24	3.37E-23
54795	3687	TRPM4	1.01	7.56E-20	6.80E-19
2182	5056	ACSL4	1.01	2.56E-34	4.20E-33
7849	3930	PAX8	1.01	1.18E-33	1.89E-32
93953	2798	GCNA	1.00	0.003045605	0.006799988
126823	1329	KLHDC9	1.00	0.001357278	0.003230558
51747	2948	LUC7L3	1.00	1.51E-27	1.93E-26
4069	1516	LYZ	1.00	0.005325351	0.011406133
158399	10324	ZNF483	1.00	2.55E-08	1.08E-07
51807	2124	TUBA8	1.00	5.07E-07	1.89E-06
57545	3070	CC2D2A	1.00	9.53E-13	5.64E-12
344387	3124	CDKL4	1.00	0.001462997	0.003464091
79064	954	TMEM223	1.00	5.35E-15	3.70E-14
4835	1245	NQO2	1.00	1.60E-16	1.21E-15
200162	6734	SPAG17	1.00	0.025885312	0.048129994
10449	1952	ACAA2	1.00	1.33E-27	1.71E-26
3716	4985	JAK1	1.00	2.11E-56	6.80E-55
10312	2677	TCIRG1	1.00	3.52E-48	8.85E-47
55356	4544	SLC22A15	1.00	6.99E-14	4.51E-13
147011	1513	PROCA1	1.00	0.000749954	0.001869151
152756	2174	FAM218A	1.00	0.000995363	0.002427342
389015	4074	SLC9A4	1.00	0.00117743	0.002834381
63917	2682	GALNT11	1.00	4.59E-26	5.47E-25
1040	4493	CDS1	1.00	1.01E-07	4.06E-07
26575	1415	RGS17	1.00	0.000152124	0.000421478
286527	683	TMSB15B	1.00	6.74E-05	0.000196002
255231	2926	MCOLN2	1.00	0.00029452	0.000781165
114960	2058	TSGA13	1.00	0.012772065	0.025333115
5359	1960	PLSCR1	0.99	5.77E-27	7.20E-26
3801	3504	KIFC3	0.99	5.37E-32	8.18E-31
51005	3101	AMDHD2	0.99	5.31E-22	5.26E-21
197021	2567	LCTL	0.99	0.021168743	0.040073112
245973	2867	ATP6V1C2	0.99	0.001981785	0.00457422
6002	3979	RGS12	0.99	2.93E-28	3.85E-27
400986	8072	ANKRD36C	0.99	4.64E-10	2.26E-09
1.08E+08	1121	LOC107985911	0.99	0.001524109	0.003594898
1.05E+08	1599	LOC105374378	0.99	0.016375276	0.031836734
3993	2673	LLGL2	0.99	1.12E-14	7.61E-14
4982	2354	TNFRSF11B	0.99	1.49E-19	1.32E-18
646588	1343	LOC646588	0.99	0.016392599	0.031866925
8974	2768	P4HA2	0.99	1.21E-41	2.53E-40
55784	6732	MCTP2	0.99	4.16E-24	4.53E-23
80975	2321	TMPRSS5	0.99	0.025556122	0.047567691
8502	4140	PKP4	0.99	4.42E-46	1.06E-44
143570	4704	XRRA1	0.99	8.11E-09	3.59E-08
151242	1079	PPP1R1C	0.99	0.022377292	0.04213647

27040	1680	LAT	0.99	0.00029356	0.00077897
90060	4130	CCDC120	0.99	5.15E-16	3.79E-15
8792	4458	TNFRSF11A	0.99	4.11E-09	1.86E-08
5732	2494	PTGER2	0.99	1.57E-23	1.67E-22
4983	7481	OPHN1	0.99	4.41E-22	4.40E-21
5251	5091	PHEX	0.99	0.002072205	0.004764972
8907	2373	AP1M1	0.98	5.75E-39	1.11E-37
387921	3526	NHLRC3	0.98	1.51E-20	1.41E-19
84803	2616	GPAT3	0.98	3.36E-07	1.28E-06
28978	1015	TMEM14A	0.98	7.95E-19	6.78E-18
6352	1249	CCL5	0.98	3.20E-05	9.72E-05
3373	2734	HYAL1	0.98	1.20E-10	6.15E-10
25956	4631	SEC31B	0.98	2.17E-13	1.35E-12
221336	3113	BEND6	0.98	8.19E-07	2.98E-06
4494	456	MT1F	0.98	2.59E-15	1.83E-14
132884	4372	EVC2	0.98	1.84E-13	1.15E-12
9443	1140	MED7	0.98	2.64E-14	1.76E-13
144363	1180	ETFRF1	0.98	2.74E-11	1.48E-10
60492	2335	CCDC90B	0.98	5.95E-28	7.76E-27
51131	1504	PHF11	0.98	4.30E-13	2.59E-12
65109	2371	UPF3B	0.98	2.37E-19	2.09E-18
727866	3046	FAM156B	0.98	2.41E-18	2.02E-17
1269	5679	CNR2	0.98	0.021656851	0.04092155
7295	886	TXN	0.98	1.07E-28	1.43E-27
253639	2979	ZNF620	0.98	6.34E-08	2.59E-07
79026	13763	AHNAK	0.98	2.92E-62	1.08E-60
1.05E+08	4282	LOC105369199	0.98	0.000638647	0.001607975
1E+08	1672	GOLGA6L3	0.98	0.021661222	0.040922552
1.01E+08	3987	ARPC4-TTLL3	0.98	6.67E-15	4.59E-14
7786	3592	MAP3K12	0.98	2.05E-22	2.08E-21
55558	6386	PLXNA3	0.98	3.90E-24	4.25E-23
81786	1575	TRIM7	0.97	6.28E-08	2.57E-07
32	8887	ACACB	0.97	7.21E-12	4.02E-11
2444	3097	FRK	0.97	1.17E-05	3.78E-05
284	3741	ANGPT1	0.97	0.019543296	0.037326161
5054	3151	SERPINE1	0.97	4.34E-30	6.16E-29
51347	4433	TAOK3	0.97	1.66E-27	2.12E-26
51020	1615	HDDC2	0.97	5.55E-18	4.55E-17
822	1321	CAPG	0.97	4.50E-31	6.65E-30
56990	3616	CDC42SE2	0.97	7.26E-34	1.18E-32
10929	4345	SRSF8	0.97	6.95E-25	7.85E-24
284001	4543	CCDC57	0.97	3.22E-19	2.81E-18
1029	1091	CDKN2A	0.97	2.55E-31	3.79E-30
79868	2992	ALG13	0.97	2.07E-22	2.09E-21
55251	3929	PCMTD2	0.97	5.72E-23	5.94E-22
112483	973	SAT2	0.97	1.64E-19	1.45E-18
55567	9045	DNAH3	0.97	0.01507692	0.029493198
6659	4912	SOX4	0.97	6.16E-38	1.15E-36

4703	25046	NEB	0.97	0.022829648	0.042915487
2571	2989	GAD1	0.97	3.89E-07	1.47E-06
55227	3261	LRRC1	0.97	7.85E-18	6.39E-17
10903	2996	MTMR11	0.97	8.04E-14	5.15E-13
84514	2506	GHDC	0.97	3.19E-21	3.05E-20
26017	1489	FAM32A	0.97	1.18E-34	1.96E-33
9441	3184	MED26	0.96	1.77E-17	1.41E-16
147166	2049	TRIM16L	0.96	8.87E-17	6.82E-16
91433	2694	RCCD1	0.96	3.12E-18	2.59E-17
3918	5558	LAMC2	0.96	1.16E-27	1.50E-26
57616	4995	TSHZ3	0.96	1.05E-14	7.15E-14
54504	1656	CPVL	0.96	0.0055007	0.011731658
414236	2516	C10orf55	0.96	0.001740055	0.004058452
2760	3690	GM2A	0.96	8.31E-31	1.21E-29
79960	5471	JADE1	0.96	9.72E-41	1.99E-39
55608	2070	ANKRD10	0.96	3.82E-27	4.80E-26
9920	6744	KBTBD11	0.96	1.84E-21	1.77E-20
63901	3638	FAM111A	0.96	5.08E-45	1.19E-43
5813	5304	PURA	0.96	3.38E-25	3.88E-24
147645	3674	VSIG10L	0.96	1.29E-05	4.14E-05
8767	2364	RIPK2	0.96	1.57E-16	1.19E-15
114569	2818	MAL2	0.96	5.91E-27	7.37E-26
258010	942	SVIP	0.96	5.05E-18	4.14E-17
56898	2827	BDH2	0.96	5.15E-23	5.36E-22
91662	3709	NLRP12	0.95	0.017913907	0.034529648
51657	1214	STYXL1	0.95	7.73E-18	6.30E-17
140885	3917	SIRPA	0.95	5.80E-52	1.64E-50
6712	9303	SPTBN2	0.95	1.07E-33	1.72E-32
11154	4027	AP4S1	0.95	7.99E-09	3.54E-08
80194	1060	TMEM134	0.95	4.55E-15	3.16E-14
80176	3130	SPSB1	0.95	4.91E-20	4.46E-19
11118	3765	BTN3A2	0.95	3.80E-26	4.55E-25
9467	2603	SH3BP5	0.95	2.38E-16	1.78E-15
55556	3641	ENOSF1	0.95	1.13E-19	1.01E-18
4837	1579	NNMT	0.95	5.50E-33	8.63E-32
80167	3218	ABHD18	0.95	4.47E-14	2.93E-13
26007	2839	TKFC	0.95	2.68E-25	3.08E-24
2774	5568	GNAL	0.95	0.002111422	0.004849501
259295	1914	TAS2R20	0.95	0.024784856	0.046243549
64121	2746	RRAGC	0.95	1.17E-20	1.09E-19
23308	3029	ICOSLG	0.95	0.002712701	0.006110456
252983	5055	STXBP4	0.95	1.09E-11	6.03E-11
79745	3924	CLIP4	0.95	8.00E-20	7.20E-19
715	2497	C1R	0.95	4.33E-57	1.42E-55
4214	7533	MAP3K1	0.95	3.13E-23	3.28E-22
27294	1098	DHDH	0.95	0.002610179	0.005897464
1.08E+08	1924	UPK3BL2	0.95	5.21E-10	2.53E-09
144406	3560	WDR66	0.95	0.00355978	0.007859117

25780	6004	RASGRP3	0.94	0.002141183	0.004911508
9111	1496	NMI	0.94	4.43E-21	4.21E-20
56853	3670	CELF4	0.94	0.021332579	0.040353163
9510	4670	ADAMTS1	0.94	1.07E-28	1.44E-27
5921	3885	RASA1	0.94	1.45E-24	1.62E-23
400818	4813	NBPF9	0.94	3.27E-16	2.43E-15
50614	2222	GALNT9	0.94	0.02383669	0.044624413
51750	5060	RTEL1	0.94	2.39E-20	2.20E-19
339883	2668	C3orf35	0.94	0.010000439	0.020263178
25989	2652	ULK3	0.94	2.19E-20	2.02E-19
23555	1723	TSPAN15	0.94	5.87E-14	3.81E-13
2017	3139	CTTN	0.94	1.04E-42	2.27E-41
720	5441	C4A	0.94	8.08E-07	2.94E-06
768211	3622	RELL1	0.94	2.08E-22	2.11E-21
9130	1369	FAM50A	0.94	7.73E-25	8.69E-24
93233	3508	CCDC114	0.94	0.024923779	0.046478348
2180	3794	ACSL1	0.94	1.27E-33	2.04E-32
2634	4148	GBP2	0.94	1.19E-28	1.59E-27
26355	838	FAM162A	0.94	6.78E-21	6.40E-20
7423	1798	VEGFB	0.94	1.06E-25	1.24E-24
1.08E+08	4607	LOC107985914	0.94	0.004033543	0.008815251
135295	3553	SRSF12	0.94	0.000213067	0.000576221
23174	6584	ZCCHC14	0.94	1.96E-27	2.49E-26
79695	2539	GALNT12	0.94	2.59E-16	1.94E-15
11083	7416	DIDO1	0.93	2.66E-34	4.35E-33
127018	1892	LYPLAL1	0.93	3.96E-12	2.25E-11
285955	2924	SPDYE1	0.93	0.004860476	0.010483027
203427	2552	SLC25A43	0.93	1.75E-13	1.10E-12
79939	5109	SLC35E1	0.93	5.94E-38	1.11E-36
2633	3050	GBP1	0.93	1.03E-26	1.27E-25
92017	8029	SNX29	0.93	8.95E-26	1.05E-24
5452	6907	POU2F2	0.93	1.44E-10	7.31E-10
10111	6597	RAD50	0.93	8.22E-36	1.42E-34
51523	2323	CXXC5	0.93	5.13E-46	1.23E-44
54841	3709	BIVM	0.93	1.01E-26	1.25E-25
5523	5525	PPP2R3A	0.93	1.57E-22	1.60E-21
55604	5438	CARMIL1	0.93	1.47E-27	1.88E-26
51313	4854	FAM198B	0.93	3.86E-08	1.61E-07
57480	7237	PLEKHG1	0.93	3.58E-06	1.22E-05
116983	3736	ACAP3	0.93	2.85E-29	3.92E-28
79621	1522	RNASEH2B	0.93	1.33E-16	1.01E-15
10437	1034	IFI30	0.93	4.36E-22	4.35E-21
5145	4092	PDE6A	0.92	4.31E-06	1.46E-05
23229	4978	ARHGEF9	0.92	1.67E-19	1.47E-18
3710	8902	ITPR3	0.92	1.14E-35	1.96E-34
349667	2220	RTN4RL2	0.92	6.97E-06	2.31E-05
29943	3749	PADI1	0.92	0.005855685	0.012430564
3226	2017	HOXC10	0.92	4.04E-16	2.98E-15

144321	1795	GLIPR1L2	0.92	4.15E-05	0.00012449
4144	3022	MAT2A	0.92	2.07E-32	3.19E-31
4356	2189	MPP3	0.92	7.81E-07	2.85E-06
83872	17961	HMCN1	0.92	8.84E-06	2.89E-05
147968	3129	CAPN12	0.92	0.000729118	0.001820029
25816	2042	TNFAIP8	0.92	7.68E-23	7.92E-22
84525	1117	HOPX	0.92	0.001157101	0.002789473
10221	3053	TRIB1	0.92	1.21E-23	1.29E-22
64116	2785	SLC39A8	0.92	1.06E-27	1.37E-26
25824	899	PRDX5	0.92	2.51E-34	4.13E-33
80723	2080	SLC35G2	0.92	2.59E-06	8.96E-06
266977	4996	ADGRF1	0.92	1.91E-06	6.71E-06
59338	3809	PLEKHA1	0.92	6.42E-36	1.12E-34
79776	13767	ZFHX4	0.92	1.08E-27	1.39E-26
64146	1180	PDF	0.91	5.09E-10	2.48E-09
10861	2869	SLC26A1	0.91	0.00011904	0.000334191
157773	1463	C8orf48	0.91	0.001088067	0.002636766
11147	872	HHLA3	0.91	7.16E-08	2.91E-07
9949	5395	AMMECR1	0.91	2.00E-24	2.21E-23
387104	11818	SOGA3	0.91	1.96E-13	1.22E-12
10318	2854	TNIP1	0.91	1.75E-35	3.00E-34
157869	4584	SBSPON	0.91	0.002727497	0.006139114
1429	1898	CRYZ	0.91	2.03E-32	3.14E-31
91862	2348	MARVELD3	0.91	0.002388522	0.005434659
27293	1901	SMPDL3B	0.91	9.70E-12	5.37E-11
153396	2395	TMEM161B	0.91	7.13E-16	5.20E-15
4118	1099	MAL	0.91	0.001330305	0.003170606
84304	1063	NUDT22	0.91	2.91E-13	1.78E-12
4338	3746	MOCS2	0.91	3.36E-19	2.93E-18
57035	1560	RSRP1	0.91	1.94E-16	1.46E-15
80380	2429	PDCD1LG2	0.91	2.37E-06	8.23E-06
7352	2372	UCP3	0.91	0.00207407	0.004768196
114801	3054	TMEM200A	0.91	6.74E-07	2.48E-06
10149	4692	ADGRG2	0.91	9.25E-07	3.35E-06
25937	5050	WWTR1	0.91	1.37E-48	3.50E-47
10616	2016	RBCK1	0.91	3.74E-27	4.70E-26
1406	4482	CRX	0.91	9.91E-06	3.22E-05
23092	8261	ARHGAP26	0.91	6.23E-17	4.85E-16
25906	880	ANAPC15	0.91	8.09E-17	6.24E-16
26013	3233	L3MBTL1	0.90	1.89E-06	6.64E-06
402	990	ARL2	0.90	2.08E-15	1.47E-14
2286	713	FKBP2	0.90	3.07E-22	3.08E-21
2791	964	GNG11	0.90	2.40E-17	1.91E-16
157697	3104	ERICH1	0.90	1.46E-13	9.22E-13
58494	3967	JAM2	0.90	1.18E-08	5.14E-08
55727	5856	BTBD7	0.90	1.85E-26	2.25E-25
4833	1083	NME4	0.90	5.88E-14	3.82E-13
286256	1945	LCN12	0.90	0.004585845	0.009941593

169200	4796	TMEM64	0.90	1.52E-15	1.09E-14
261729	6557	STEAP2	0.90	1.56E-09	7.31E-09
388697	9632	HRNR	0.90	0.004479177	0.009722211
55785	9288	FGD6	0.90	9.57E-19	8.14E-18
9208	4042	LRRFIP1	0.90	2.67E-37	4.86E-36
55005	1963	RMND1	0.90	1.99E-13	1.24E-12
1627	2943	DBN1	0.90	1.39E-44	3.21E-43
64112	2435	MOAP1	0.90	1.22E-20	1.14E-19
670	1905	BPHL	0.90	3.64E-10	1.79E-09
8120	3746	AP3B2	0.90	7.22E-09	3.21E-08
57116	3392	ZNF695	0.90	0.000796916	0.001974014
117144	2634	CATSPER1	0.90	0.000893172	0.002194701
132671	3938	SPATA18	0.90	5.53E-08	2.27E-07
5608	5288	MAP2K6	0.90	4.16E-08	1.73E-07
146206	4514	CARMIL2	0.90	0.002141943	0.004912178
6773	4389	STAT2	0.89	5.35E-30	7.55E-29
4253	2841	MIA2	0.89	3.77E-21	3.59E-20
4940	6609	OAS3	0.89	6.06E-31	8.91E-30
439921	2110	MXRA7	0.89	7.17E-53	2.10E-51
1.01E+08	1410	TGFBR3L	0.89	0.008058675	0.016624466
11054	2423	OGFR	0.89	2.08E-16	1.56E-15
57146	1943	TMEM159	0.89	8.01E-10	3.84E-09
80864	1311	EGFL8	0.89	5.75E-05	0.000168727
51205	2023	ACP6	0.89	1.48E-10	7.52E-10
51397	1645	COMMD10	0.89	1.28E-12	7.51E-12
4286	5393	MITF	0.89	1.26E-19	1.12E-18
153733	2479	CCDC112	0.89	4.84E-12	2.73E-11
655	4049	BMP7	0.89	0.016491456	0.032041574
79823	1629	CAMKMT	0.89	7.86E-07	2.87E-06
94121	4060	SYTL4	0.89	3.02E-15	2.13E-14
80832	3272	APOL4	0.89	0.000293401	0.000778664
388722	697	C1orf53	0.89	0.001314772	0.003135686
51186	1089	TCEAL9	0.89	8.34E-32	1.26E-30
89849	2709	ATG16L2	0.89	4.03E-24	4.39E-23
25798	837	BRI3	0.89	3.76E-17	2.96E-16
10560	3289	SLC19A2	0.89	5.44E-09	2.44E-08
2512	889	FTL	0.89	9.00E-30	1.26E-28
7867	2561	MAPKAPK3	0.89	5.66E-25	6.44E-24
1.05E+08	2645	LOC105371763	0.89	0.00138201	0.00328429
1415	781	CRYBB2	0.89	0.012887038	0.025535507
55129	2598	ANO10	0.89	6.85E-21	6.47E-20
5639	1441	PRRG2	0.89	0.004551169	0.009868827
9829	5803	DNAJC6	0.89	4.27E-09	1.93E-08
2762	1594	GMDS	0.88	1.06E-13	6.75E-13
84264	1402	HAGHL	0.88	7.07E-08	2.87E-07
28996	15176	HIPK2	0.88	3.40E-39	6.64E-38
1270	1914	CNTF	0.88	0.009209456	0.01879334
27147	3747	DENND2A	0.88	3.30E-24	3.61E-23

54830	1775	NUP62CL	0.88	1.59E-05	5.02E-05
1212	1195	CLTB	0.88	2.36E-23	2.49E-22
653784	757	MZT2A	0.88	5.94E-21	5.62E-20
1519	2943	CTSO	0.88	2.46E-17	1.96E-16
1.01E+08	5418	FSBP	0.88	7.54E-08	3.06E-07
375287	4840	RBM43	0.88	6.07E-11	3.20E-10
9220	2110	TIAF1	0.88	0.001243416	0.002979503
10636	2430	RGS14	0.88	2.37E-09	1.10E-08
83852	6059	SETDB2	0.88	6.41E-10	3.09E-09
140701	1776	ABHD16B	0.88	4.31E-05	0.000128994
80830	10161	APOL6	0.88	2.27E-25	2.62E-24
153222	7696	CREBRF	0.88	6.90E-15	4.75E-14
8503	5311	PIK3R3	0.88	1.47E-21	1.42E-20
823	3025	CAPN1	0.88	6.57E-38	1.23E-36
400581	6816	GRAPL	0.88	0.004688927	0.010142787
51099	5377	ABHD5	0.88	1.93E-15	1.37E-14
54961	2897	SSH3	0.88	5.67E-25	6.45E-24
7552	4487	ZNF711	0.88	3.02E-06	1.04E-05
1E+08	2360	WASHC1	0.87	4.87E-22	4.83E-21
3636	4783	INPPL1	0.87	7.64E-34	1.24E-32
64064	1824	OXCT2	0.87	0.006810143	0.014274227
474170	5466	LRRC37A2	0.87	7.18E-17	5.56E-16
255738	3731	PCSK9	0.87	0.000163888	0.000451679
55540	2025	IL17RB	0.87	1.18E-10	6.06E-10
115004	1951	CGAS	0.87	1.24E-08	5.42E-08
221468	1978	TMEM217	0.87	0.000728391	0.001818471
219539	2133	YPEL4	0.87	0.00219251	0.005019523
26648	1020	OR7E24	0.87	0.008527673	0.017516074
8497	5135	PPFIA4	0.87	1.96E-09	9.11E-09
26038	9663	CHD5	0.87	0.017175562	0.033239886
9314	2919	KLF4	0.87	0.000120004	0.00033658
84288	2669	EFCAB2	0.87	1.78E-12	1.04E-11
374659	944	HDDC3	0.87	4.65E-07	1.74E-06
283635	2960	FAM177A1	0.87	7.33E-28	9.48E-27
64374	1910	SIL1	0.87	3.09E-16	2.30E-15
54874	4713	FNBP1L	0.87	1.28E-26	1.56E-25
255488	4923	RNF144B	0.87	1.66E-35	2.84E-34
27246	3106	RNF115	0.87	3.70E-27	4.65E-26
26175	1019	TMEM251	0.87	2.52E-08	1.07E-07
10085	4752	EDIL3	0.87	4.00E-27	5.02E-26
55255	3815	WDR41	0.87	2.55E-19	2.24E-18
55285	6495	RBM41	0.87	1.39E-20	1.30E-19
3425	2331	IDUA	0.87	9.86E-14	6.28E-13
346653	5195	FAM71F2	0.87	0.000290836	0.000772435
728747	3517	ANKRD20A4	0.87	0.004329449	0.009416776
27345	4780	KCNMB4	0.87	2.05E-05	6.39E-05
337876	3131	CHSY3	0.87	4.59E-05	0.000136814
9955	4232	HS3ST3A1	0.86	4.52E-13	2.72E-12

51566	3428	ARMCX3	0.86	2.89E-17	2.29E-16
3665	1545	IRF7	0.86	2.00E-13	1.24E-12
7587	6950	ZNF37A	0.86	7.13E-19	6.10E-18
22982	6819	DIP2C	0.86	4.17E-17	3.27E-16
1E+08	1019	LOC100128108	0.86	5.51E-05	0.000162227
285600	5196	KIAA0825	0.86	0.000188204	0.000513753
730098	1041	LOC730098	0.86	0.000185113	0.000505701
83858	2403	ATAD3B	0.86	3.69E-19	3.21E-18
149013	7109	NBPF12	0.86	4.87E-12	2.75E-11
8630	1629	HSD17B6	0.86	0.001940669	0.004486315
254778	3471	C8orf46	0.86	0.022253293	0.04192075
10058	3021	ABCB6	0.86	1.81E-09	8.45E-09
8935	4013	SKAP2	0.86	2.52E-27	3.19E-26
23094	8074	SIPA1L3	0.86	2.21E-25	2.56E-24
56731	2296	SLC2A4RG	0.86	7.76E-17	6.00E-16
728741	2209	NPIPB6	0.86	2.68E-06	9.27E-06
9456	4322	HOMER1	0.86	3.81E-14	2.51E-13
9189	4549	ZBED1	0.86	9.46E-22	9.26E-21
55331	6825	ACER3	0.85	8.44E-26	9.95E-25
139818	5566	DOCK11	0.85	4.03E-19	3.49E-18
1525	5155	CXADR	0.85	3.66E-13	2.23E-12
80279	2671	CDK5RAP3	0.85	2.19E-21	2.11E-20
3108	1122	HLA-DMA	0.85	4.02E-09	1.82E-08
734	4275	OSGIN2	0.85	4.71E-22	4.68E-21
3074	1920	HEXB	0.85	5.97E-41	1.23E-39
54332	3853	GDAP1	0.85	1.43E-12	8.38E-12
1.03E+08	3489	LOC102723996	0.85	0.000395061	0.001029849
4891	4091	SLC11A2	0.85	9.34E-22	9.16E-21
7165	2329	TPD52L2	0.85	9.13E-30	1.28E-28
23253	7135	ANKRD12	0.85	1.48E-28	1.97E-27
2246	3745	FGF1	0.85	0.006560334	0.01379619
10717	3299	AP4B1	0.85	1.55E-11	8.44E-11
3156	4561	HMGCR	0.85	4.25E-18	3.51E-17
3216	1979	HOXB6	0.85	4.26E-22	4.26E-21
140738	1687	TMEM37	0.85	4.00E-07	1.51E-06
7678	2222	ZNF124	0.85	3.36E-05	0.000101779
84153	2785	RNASEH2C	0.85	3.04E-21	2.92E-20
497661	1557	C18orf32	0.84	2.80E-21	2.69E-20
85027	2344	SMIM3	0.84	1.17E-19	1.04E-18
55004	1198	LAMTOR1	0.84	1.03E-20	9.67E-20
25874	1164	MPC2	0.84	5.35E-16	3.93E-15
10409	1881	BASP1	0.84	6.51E-23	6.74E-22
25923	7116	ATL3	0.84	3.14E-31	4.67E-30
57453	6190	DSCAML1	0.84	1.04E-07	4.16E-07
84240	1401	ZCCHC9	0.84	2.52E-13	1.55E-12
116966	7298	WDR17	0.84	6.92E-05	0.000200788
79875	8105	THSD4	0.84	2.00E-28	2.65E-27
65065	16150	NBEAL1	0.84	4.77E-16	3.51E-15

11010	3921	GLIPR1	0.84	2.54E-30	3.65E-29
7593	2795	MZF1	0.84	2.18E-11	1.18E-10
1.01E+08	9812	TMEM178B	0.84	2.54E-24	2.80E-23
5833	3559	PCYT2	0.84	1.44E-14	9.73E-14
9973	1068	CCS	0.84	2.94E-13	1.80E-12
29953	5599	TRHDE	0.84	7.63E-09	3.39E-08
22993	5171	HMGXB3	0.84	7.71E-27	9.55E-26
130271	6977	PLEKHH2	0.84	0.000257404	0.000688686
345757	1363	FAM174A	0.84	9.14E-10	4.36E-09
54879	3945	ST7L	0.84	2.60E-09	1.20E-08
4671	4776	NAIP	0.84	1.05E-10	5.43E-10
9149	2532	DYRK1B	0.84	8.94E-14	5.72E-13
65123	4369	INTS3	0.84	1.85E-22	1.88E-21
22932	1564	POMZP3	0.84	1.04E-06	3.77E-06
23309	4762	SIN3B	0.84	6.03E-23	6.25E-22
8417	4068	STX7	0.84	1.87E-24	2.07E-23
266747	2897	RGL4	0.84	2.44E-05	7.54E-05
1847	2545	DUSP5	0.84	7.70E-15	5.29E-14
9472	9888	AKAP6	0.84	0.004860967	0.010483027
646851	7548	FAM227A	0.83	0.000360594	0.000944571
2161	1885	F12	0.83	0.001711803	0.003998858
7416	1866	VDAC1	0.83	2.19E-33	3.49E-32
51557	6376	LGSN	0.83	0.011561416	0.023103035
54760	2643	PCSK4	0.83	9.22E-05	0.00026328
7837	6661	PXDN	0.83	8.58E-44	1.95E-42
81789	3988	TIGD6	0.83	4.33E-06	1.47E-05
474343	1188	SPIN2B	0.83	4.73E-06	1.59E-05
3227	2100	HOXC11	0.83	0.000288684	0.000767062
115207	6255	KCTD12	0.83	5.36E-12	3.01E-11
23305	6316	ACSL6	0.83	0.023602722	0.044239081
119016	3685	AGAP4	0.83	4.76E-10	2.32E-09
6715	2335	SRD5A1	0.83	2.51E-11	1.35E-10
51149	1224	MRNIP	0.83	0.000446801	0.001154156
51109	2578	RDH11	0.83	1.38E-26	1.68E-25
272	4110	AMPD3	0.83	7.18E-08	2.92E-07
79884	5102	MAP9	0.83	1.69E-19	1.50E-18
729092	2426	AGAP5	0.83	4.72E-05	0.000140419
29893	1468	PSMC3IP	0.83	6.50E-12	3.63E-11
26034	6775	IPCEF1	0.83	0.016756349	0.032517127
643382	1415	TMEM253	0.82	0.015946404	0.0310607
9604	4189	RNF14	0.82	2.10E-18	1.76E-17
5587	3675	PRKD1	0.82	5.48E-12	3.08E-11
142684	3290	RAB40A	0.82	0.004106165	0.008958539
1471	932	CST3	0.82	2.67E-29	3.68E-28
284565	4015	NBPF15	0.82	4.94E-15	3.43E-14
23438	2463	HARS2	0.82	3.33E-14	2.20E-13
10397	3130	NDRG1	0.82	6.55E-30	9.23E-29
1.02E+08	3863	LOC101927506	0.82	0.000105295	0.00029829

8717	1607	TRADD	0.82	4.37E-08	1.81E-07
123096	3579	SLC25A29	0.82	2.62E-20	2.40E-19
57162	3726	PELI1	0.82	4.91E-11	2.60E-10
63920	2900	ZBED8	0.82	0.000812647	0.002009338
729993	6178	SHISA9	0.82	1.26E-28	1.68E-27
284114	2093	TMEM102	0.82	2.03E-05	6.34E-05
54972	3602	TMEM132A	0.82	3.69E-33	5.82E-32
23408	3250	SIRT5	0.82	2.43E-10	1.21E-09
6728	1515	SRP19	0.82	6.39E-19	5.48E-18
2617	2448	GARS	0.82	2.15E-36	3.82E-35
256329	2186	LMNTD2	0.82	3.10E-06	1.06E-05
440	1949	ASNS	0.82	1.92E-15	1.37E-14
1466	936	CSRP2	0.82	2.81E-20	2.57E-19
4707	433	NDUFB1	0.82	2.38E-16	1.78E-15
55240	3971	STEAP3	0.82	2.40E-18	2.00E-17
9367	1345	RAB9A	0.81	6.34E-14	4.11E-13
828	2135	CAPS	0.81	5.39E-05	0.000159165
7538	1752	ZFP36	0.81	2.51E-10	1.25E-09
84441	6111	MAML2	0.81	2.52E-15	1.78E-14
80745	1700	THUMPD2	0.81	1.29E-10	6.61E-10
83943	1417	IMMP2L	0.81	1.60E-05	5.07E-05
8611	1687	PLPP1	0.81	3.84E-10	1.88E-09
3123	1235	HLA-DRB1	0.81	0.000582923	0.001478992
26118	3453	WSB1	0.81	1.65E-17	1.33E-16
221178	7072	SPATA13	0.81	1.17E-12	6.89E-12
51071	1819	DERA	0.81	5.33E-21	5.05E-20
51192	807	CKLF	0.81	7.93E-14	5.08E-13
60489	1839	APOBEC3G	0.81	1.72E-07	6.73E-07
729920	4401	ISPD	0.81	0.000446291	0.001153075
643836	4068	ZFP62	0.81	2.21E-11	1.19E-10
79713	1217	IGFLR1	0.81	5.66E-05	0.000166218
55268	2338	ECHDC2	0.81	3.50E-12	1.99E-11
55728	9318	N4BP2	0.81	2.31E-12	1.33E-11
10227	1881	MFSD10	0.81	4.25E-26	5.08E-25
5896	6582	RAG1	0.81	0.017126731	0.033156216
83714	3350	NRIP2	0.81	0.01977469	0.037711355
9414	4449	TJP2	0.81	6.36E-28	8.28E-27
25897	4113	RNF19A	0.81	9.99E-24	1.07E-22
50804	5789	MYEF2	0.81	2.70E-21	2.59E-20
727957	4878	MROH1	0.81	2.25E-14	1.50E-13
1.08E+08	2122	LOC107986724	0.81	0.000304201	0.000805159
10040	2310	TOM1L1	0.81	2.91E-17	2.30E-16
55552	2433	ZNF823	0.81	1.06E-06	3.83E-06
151613	4238	TTC14	0.81	9.47E-12	5.25E-11
10626	2920	TRIM16	0.81	8.00E-12	4.45E-11
8111	2902	GPR68	0.80	0.000535914	0.001365271
401548	7409	SNX30	0.80	7.88E-17	6.08E-16
64770	4033	CCDC14	0.80	5.36E-15	3.70E-14

23314	5158	SATB2	0.80	1.14E-11	6.25E-11
92092	2813	ZC3HAV1L	0.80	3.82E-13	2.32E-12
23162	6339	MAPK8IP3	0.80	3.95E-17	3.10E-16
147184	2079	TMEM99	0.80	2.72E-05	8.36E-05
653319	3416	KIAA0895L	0.80	7.40E-10	3.56E-09
8092	1319	ALX1	0.80	0.00027865	0.000742065
10257	5782	ABCC4	0.80	1.28E-23	1.37E-22
25957	4419	PNISR	0.80	4.29E-21	4.08E-20
23710	1865	GABARAPL1	0.80	4.67E-21	4.43E-20
57175	1930	CORO1B	0.80	1.28E-21	1.24E-20
57553	8098	MICAL3	0.80	4.78E-20	4.35E-19
220064	2550	ORAOV1	0.80	1.20E-08	5.23E-08
10559	1875	SLC35A1	0.80	3.67E-13	2.23E-12
1191	2878	CLU	0.80	2.41E-29	3.34E-28
6672	2801	SP100	0.80	2.34E-22	2.36E-21
10008	2958	KCNE3	0.80	0.012837358	0.025451259
1850	4581	DUSP8	0.80	9.47E-09	4.18E-08
51287	912	COA4	0.80	2.41E-22	2.42E-21
116150	4811	NUS1	0.80	4.84E-26	5.77E-25
25827	2603	FBXL2	0.80	1.58E-06	5.58E-06
388282	1607	LOC388282	0.80	0.000272324	0.00072609
201895	5443	SMIM14	0.79	5.51E-22	5.45E-21
4300	6545	MLLT3	0.79	6.11E-12	3.43E-11
2184	1810	FAH	0.79	3.75E-14	2.47E-13
80114	5392	BICC1	0.79	9.99E-36	1.73E-34
342908	1868	ZNF404	0.79	0.000149716	0.000415519
51303	768	FKBP11	0.79	0.003989131	0.008723554
200810	2988	ALG1L	0.79	0.025950826	0.048246759
5774	6918	PTPN3	0.79	9.71E-20	8.70E-19
79925	3883	SPEF2	0.79	0.000601712	0.001521883
64847	2774	SPATA20	0.79	5.86E-26	6.96E-25
10656	1855	KHDRBS3	0.79	1.60E-07	6.29E-07
51195	3644	RAPGEFL1	0.79	1.82E-07	7.10E-07
5293	5264	PIK3CD	0.79	1.82E-15	1.30E-14
1.01E+08	2234	LOC100996721	0.79	8.70E-06	2.84E-05
324	10785	APC	0.79	9.15E-20	8.21E-19
55577	1374	NAGK	0.79	2.60E-20	2.38E-19
387914	2889	SHISA2	0.79	8.38E-13	4.98E-12
1.05E+08	4310	LOC105374013	0.79	0.00131765	0.003142129
64283	5366	ARHGEF28	0.79	3.87E-23	4.04E-22
284996	2983	RNF149	0.79	3.76E-18	3.11E-17
152687	2901	ZNF595	0.78	1.81E-06	6.37E-06
55111	1301	PLEKHJ1	0.78	6.91E-14	4.46E-13
26040	8810	SETBP1	0.78	9.59E-14	6.12E-13
65983	2689	GRAMD2B	0.78	1.43E-16	1.08E-15
1353	2809	COX11	0.78	3.77E-23	3.93E-22
27242	3646	TNFRSF21	0.78	2.51E-17	2.00E-16
10063	423	COX17	0.78	1.65E-10	8.36E-10

114822	3834	RHPN1	0.78	1.59E-13	9.96E-13
7345	1141	UCHL1	0.78	6.27E-25	7.12E-24
728492	911	SERF1B	0.78	1.24E-06	4.43E-06
55601	6091	DDX60	0.78	1.49E-10	7.58E-10
8794	1512	TNFRSF10C	0.78	0.006069969	0.012850138
58527	940	ABRACL	0.78	6.64E-12	3.71E-11
3157	3596	HMGCS1	0.78	2.38E-19	2.09E-18
54853	3701	WDR55	0.78	4.48E-22	4.47E-21
6535	3264	SLC6A8	0.78	4.70E-22	4.68E-21
119391	1680	GSTO2	0.78	1.54E-10	7.83E-10
11144	2153	DMC1	0.78	8.61E-05	0.00024684
321	3230	APBA2	0.78	1.46E-06	5.19E-06
91	4478	ACVR1B	0.78	2.76E-27	3.49E-26
8835	2484	SOCS2	0.78	0.010218595	0.020669854
65258	2211	MPPE1	0.78	1.19E-10	6.10E-10
51106	2491	TFB1M	0.77	3.26E-09	1.49E-08
151194	4118	METTL21A	0.77	4.35E-18	3.59E-17
871	2142	SERPINH1	0.77	2.75E-30	3.93E-29
55008	3721	HERC6	0.77	5.48E-10	2.66E-09
220074	2813	LRTOMT	0.77	1.23E-06	4.41E-06
529	1404	ATP6V1E1	0.77	1.16E-22	1.19E-21
161436	7500	EML5	0.77	0.000192486	0.000524714
154214	10731	RNF217	0.77	1.31E-12	7.70E-12
51466	2240	EVL	0.77	9.90E-20	8.86E-19
3706	1726	ITPKA	0.77	1.62E-07	6.35E-07
84833	447	ATP5MD	0.77	3.47E-15	2.43E-14
445815	7504	PALM2-AKAP2	0.77	0.020169173	0.038373045
393	3285	ARHGAP4	0.77	0.000144084	0.000400451
160622	2252	GRASP	0.77	0.021337999	0.040359118
57724	12524	EPG5	0.77	2.08E-17	1.66E-16
53917	1609	RAB24	0.77	3.44E-14	2.27E-13
1774	3286	DNASE1L1	0.77	2.49E-12	1.43E-11
29964	1646	PRICKLE4	0.77	2.55E-07	9.81E-07
6810	1512	STX4	0.77	1.19E-10	6.10E-10
8743	1947	TNFSF10	0.77	5.64E-30	7.96E-29
977	1554	CD151	0.77	9.32E-29	1.26E-27
79017	1191	GGCT	0.77	2.51E-16	1.88E-15
6319	5473	SCD	0.77	1.06E-24	1.18E-23
3945	1336	LDHB	0.77	7.54E-37	1.35E-35
65078	1924	RTN4R	0.76	2.86E-09	1.31E-08
5883	2195	RAD9A	0.76	4.53E-09	2.05E-08
1657	11600	DMXL1	0.76	1.82E-20	1.68E-19
1014	2895	CDH16	0.76	1.46E-21	1.41E-20
11221	2676	DUSP10	0.76	2.14E-05	6.65E-05
56929	5814	FEM1C	0.76	4.12E-15	2.87E-14
79080	2022	CCDC86	0.76	1.29E-19	1.15E-18
23125	4474	CAMTA2	0.76	2.58E-15	1.82E-14
23230	11068	VPS13A	0.76	5.61E-14	3.65E-13

85444	3404	LRRCC1	0.76	1.32E-11	7.25E-11
51135	4290	IRAK4	0.76	1.64E-08	7.08E-08
9223	7200	MAGI1	0.76	6.23E-13	3.72E-12
7292	8104	TNFSF4	0.76	0.017134218	0.033167098
10523	4105	CHERP	0.76	4.06E-19	3.51E-18
231	1415	AKR1B1	0.76	3.14E-24	3.45E-23
147660	6741	ZNF578	0.76	0.003702573	0.008150065
84259	3497	DCUN1D5	0.76	1.80E-18	1.51E-17
349565	2103	NMNAT3	0.76	0.000378804	0.000989793
51309	2141	ARMCX1	0.76	1.77E-06	6.24E-06
26152	4201	ZNF337	0.76	9.47E-11	4.90E-10
1E+08	1056	LOC100289561	0.76	6.21E-06	2.07E-05
10072	3088	DPP3	0.76	2.83E-20	2.59E-19
54915	3277	YTHDF1	0.76	6.97E-20	6.29E-19
4301	7733	AFDN	0.76	4.86E-24	5.27E-23
4857	2363	NOVA1	0.76	9.78E-08	3.92E-07
4638	3585	MYLK	0.76	1.85E-33	2.95E-32
338692	2131	ANKRD13D	0.75	5.35E-14	3.49E-13
79144	794	PPDPF	0.75	0.000269742	0.000719853
7351	1646	UCP2	0.75	3.26E-19	2.84E-18
80759	1435	KHDC1	0.75	0.002091698	0.004807307
8831	6011	SYNGAP1	0.75	3.33E-09	1.52E-08
84812	2663	PLCD4	0.75	2.17E-06	7.57E-06
56954	1271	NIT2	0.75	4.57E-15	3.18E-14
10577	921	NPC2	0.75	4.82E-30	6.82E-29
112399	2513	EGLN3	0.75	1.44E-05	4.60E-05
716	2795	C1S	0.75	2.28E-24	2.51E-23
79958	2600	DENND1C	0.75	0.002980662	0.006663362
386618	2133	KCTD4	0.75	0.004824338	0.010416677
4694	502	NDUFA1	0.75	1.21E-10	6.20E-10
58487	6246	CREBZF	0.75	7.12E-13	4.25E-12
55374	1818	TMCO6	0.75	3.85E-07	1.46E-06
51429	3890	SNX9	0.75	1.08E-23	1.16E-22
51428	2503	DDX41	0.75	3.47E-22	3.48E-21
55856	4126	ACOT13	0.75	2.90E-13	1.78E-12
3215	1830	HOXB5	0.75	2.64E-10	1.31E-09
84532	3447	ACSS1	0.75	1.77E-05	5.58E-05
50848	4830	F11R	0.75	3.03E-23	3.18E-22
91373	3292	UAP1L1	0.75	3.17E-15	2.23E-14
7566	2530	ZNF18	0.75	6.31E-06	2.10E-05
1183	6762	CLCN4	0.75	1.44E-06	5.11E-06
23043	9771	TNIK	0.75	5.40E-14	3.52E-13
5870	3045	RAB6A	0.75	1.56E-24	1.73E-23
51090	1514	PLLP	0.75	0.000427926	0.00110885
441869	1645	ANKRD65	0.75	0.003306239	0.007331243
8906	2372	AP1G2	0.75	6.68E-20	6.04E-19
79609	1965	VCPKMT	0.75	5.11E-06	1.72E-05
151056	4972	PLB1	0.75	0.007684629	0.01593052

54994	4440	GID8	0.74	3.08E-17	2.44E-16
7124	1686	TNF	0.74	8.19E-06	2.69E-05
84167	3108	C19orf44	0.74	1.72E-08	7.39E-08
7580	1319	ZNF32	0.74	9.35E-09	4.13E-08
4820	7758	NKTR	0.74	0.000282324	0.000751623
8934	3130	RAB29	0.74	2.50E-13	1.54E-12
340348	2089	TSPAN33	0.74	6.33E-15	4.36E-14
23499	17903	MACF1	0.74	6.90E-32	1.05E-30
800	4452	CALD1	0.74	1.92E-29	2.67E-28
66035	3036	SLC2A11	0.74	6.99E-08	2.84E-07
347252	3559	IGFBPL1	0.74	0.004944649	0.010644528
1.01E+08	891	LOC100653061	0.74	2.42E-05	7.47E-05
8741	1927	TNFSF13	0.74	2.39E-13	1.47E-12
138639	4846	PTPDC1	0.74	2.87E-09	1.32E-08
84572	1251	GNPTG	0.74	4.39E-18	3.61E-17
51134	3626	CEP83	0.74	4.26E-08	1.77E-07
10536	2690	P3H3	0.74	1.22E-19	1.09E-18
5411	3606	PNN	0.74	6.89E-14	4.45E-13
51057	2901	WDPCP	0.74	1.39E-06	4.95E-06
158431	4404	ZNF782	0.74	1.62E-05	5.11E-05
23367	6538	LARP1	0.74	2.61E-38	4.97E-37
400793	4146	C1orf226	0.74	3.72E-07	1.41E-06
57657	3516	HCN3	0.74	5.37E-08	2.21E-07
29126	2933	CD274	0.74	1.69E-08	7.26E-08
221895	3068	JAZF1	0.74	6.12E-10	2.95E-09
55568	5966	GALNT10	0.74	4.55E-25	5.20E-24
3241	1692	HPCAL1	0.74	4.47E-13	2.69E-12
3275	3097	PRMT2	0.73	4.48E-22	4.47E-21
30008	2096	EFEMP2	0.73	9.41E-31	1.37E-29
57142	2055	RTN4	0.73	1.58E-29	2.20E-28
390595	6252	UBAP1L	0.73	0.009194519	0.018768687
113452	1074	TMEM54	0.73	4.56E-14	2.99E-13
56109	4605	PCDHGA6	0.73	0.010338928	0.020891854
2593	1181	GAMT	0.73	9.83E-09	4.33E-08
728498	5261	GOLGA8H	0.73	1.48E-05	4.72E-05
167153	2842	PAPD4	0.73	2.55E-15	1.80E-14
7762	3675	ZNF215	0.73	1.78E-05	5.59E-05
1.02E+08	1558	LOC101927789	0.73	0.009855949	0.020000057
6303	1158	SAT1	0.73	9.27E-22	9.10E-21
23001	12775	WDFY3	0.73	1.88E-15	1.34E-14
10497	5901	UNC13B	0.73	1.06E-14	7.21E-14
10039	2517	PARP3	0.73	2.69E-14	1.79E-13
201229	1516	LYRM9	0.73	0.004529173	0.009825928
5699	1019	PSMB10	0.73	1.17E-16	8.92E-16
10205	2718	MPZL2	0.73	2.86E-06	9.86E-06
642987	4580	TMEM232	0.73	0.025336921	0.047194298
54893	4948	MTMR10	0.73	5.33E-17	4.16E-16
5777	2250	PTPN6	0.73	4.74E-09	2.14E-08

404281	2741	YY2	0.73	0.001737588	0.00405323
9905	4496	SGSM2	0.73	3.79E-17	2.98E-16
125150	1971	ZSWIM7	0.73	2.23E-06	7.76E-06
1.01E+08	3043	NPIPB9	0.73	3.90E-05	0.000117136
57494	6611	RIMKLB	0.73	4.27E-11	2.27E-10
51426	5134	POLK	0.73	5.44E-16	3.99E-15
25841	4919	ABTB2	0.73	2.10E-12	1.21E-11
25943	6816	C20orf194	0.72	7.74E-11	4.04E-10
4646	8665	MYO6	0.72	2.36E-20	2.17E-19
210	3214	ALAD	0.72	3.80E-17	2.99E-16
79829	3675	NAA40	0.72	6.09E-18	4.98E-17
1176	1689	AP3S1	0.72	1.66E-18	1.39E-17
23287	4973	AGTPBP1	0.72	1.12E-17	9.06E-17
57475	6361	PLEKHH1	0.72	8.03E-06	2.64E-05
140564	3152	APOBEC3D	0.72	0.000111672	0.000315352
29109	3092	FHOD1	0.72	4.07E-14	2.67E-13
440295	4241	GOLGA6L9	0.72	1.79E-07	6.99E-07
1.08E+08	6396	LOC107986211	0.72	0.006686499	0.014043229
80216	5090	ALPK1	0.72	7.01E-09	3.12E-08
728294	2114	D2HGDH	0.72	2.51E-08	1.07E-07
9600	4249	PITPNM1	0.72	7.27E-16	5.29E-15
57060	2175	PCBP4	0.72	1.53E-15	1.10E-14
5069	9509	PAPPA	0.72	9.96E-06	3.23E-05
285590	7793	SH3PXD2B	0.72	1.88E-17	1.51E-16
151556	7405	GPR155	0.72	0.000486288	0.001247346
79627	8798	OGFRL1	0.72	2.24E-23	2.37E-22
80765	1462	STARD5	0.72	0.010532006	0.021248176
1.01E+08	1707	ARL17B	0.72	0.009288346	0.018947799
29903	1640	CCDC106	0.72	6.23E-08	2.55E-07
7422	2991	VEGFA	0.72	1.00E-18	8.51E-18
7172	3289	TPMT	0.72	3.66E-15	2.56E-14
51015	1942	ISOC1	0.72	5.33E-12	3.00E-11
1.01E+08	923	RBM14-RBM4	0.72	0.015748175	0.03070488
23635	2266	SSBP2	0.72	1.16E-10	5.97E-10
81706	2215	PPP1R14C	0.72	5.16E-11	2.73E-10
23142	4468	DCUN1D4	0.72	1.21E-19	1.07E-18
9721	3659	GPRIN2	0.72	0.000162068	0.000447007
283237	1118	TTC9C	0.72	5.69E-07	2.11E-06
643338	2503	C15orf62	0.71	0.000547897	0.001394704
2887	4826	GRB10	0.71	4.01E-13	2.43E-12
10139	1821	ARFRP1	0.71	7.45E-10	3.58E-09
89932	5802	PAPLN	0.71	3.28E-16	2.44E-15
10682	1191	EBP	0.71	3.11E-15	2.19E-14
10404	1953	CPQ	0.71	4.20E-08	1.75E-07
1947	3344	EFNB1	0.71	5.92E-09	2.65E-08
55163	3348	PNPO	0.71	2.66E-16	1.99E-15
9953	5184	HS3ST3B1	0.71	0.001339338	0.003189994
9315	1995	NREP	0.71	2.16E-24	2.38E-23

53343	2746	NUDT9	0.71	1.24E-13	7.87E-13
84817	2075	TXNDC17	0.71	2.15E-06	7.51E-06
10998	2129	SLC27A5	0.71	0.003929932	0.008603627
90933	3504	TRIM41	0.71	7.46E-14	4.80E-13
219927	717	MRPL21	0.71	2.63E-08	1.12E-07
5095	2555	PCCA	0.71	2.95E-10	1.46E-09
340481	9733	ZDHHC21	0.71	2.42E-08	1.03E-07
285362	2168	SUMF1	0.71	7.21E-11	3.77E-10
55260	2545	TMEM143	0.71	2.71E-06	9.35E-06
64403	3502	CDH24	0.71	5.47E-11	2.89E-10
83699	4379	SH3BGRL2	0.71	3.89E-10	1.91E-09
572	1004	BAD	0.71	1.16E-13	7.33E-13
91749	3862	MFSD4B	0.71	3.11E-07	1.18E-06
80705	4883	TSGA10	0.71	0.000535928	0.001365271
285605	5731	DTWD2	0.70	6.90E-09	3.08E-08
119032	2392	BORCS7	0.70	2.76E-09	1.27E-08
8916	4505	HERC3	0.70	9.62E-11	4.97E-10
10476	627	ATP5PD	0.70	1.51E-17	1.21E-16
7431	2151	VIM	0.70	9.07E-29	1.22E-27
85025	882	TMEM60	0.70	1.09E-06	3.91E-06
80326	2100	WNT10A	0.70	0.007269477	0.015137005
83637	5031	ZMIZ2	0.70	1.54E-11	8.42E-11
1284	6264	COL4A2	0.70	7.59E-32	1.15E-30
55132	3680	LARP1B	0.70	3.29E-16	2.44E-15
85456	5818	TNKS1BP1	0.70	8.31E-21	7.82E-20
5460	1936	POU5F1	0.70	2.92E-08	1.23E-07
960	4588	CD44	0.70	1.67E-23	1.78E-22
51547	1913	SIRT7	0.70	2.10E-09	9.76E-09
284086	3542	NEK8	0.70	3.34E-05	0.000101325
55743	3222	CHFR	0.70	6.54E-12	3.66E-11
5371	3880	PML	0.70	6.98E-20	6.29E-19
79891	2720	ZNF671	0.70	0.003246857	0.007214876
1.01E+08	990	C16orf95	0.70	0.006075347	0.012859991
1349	456	COX7B	0.70	5.53E-10	2.68E-09
5366	1954	PMAIP1	0.70	1.24E-15	8.90E-15
5229	4503	PGGT1B	0.70	9.70E-15	6.61E-14
90843	1212	TCEAL8	0.70	1.29E-17	1.04E-16
5602	5341	MAPK10	0.70	0.002734097	0.006150853
116844	1780	LRG1	0.70	7.14E-06	2.36E-05
10133	3402	OPTN	0.70	1.23E-20	1.15E-19
7767	4037	ZNF224	0.70	5.46E-11	2.89E-10
3685	6875	ITGAV	0.70	1.68E-16	1.27E-15
113402	1109	SFT2D1	0.70	2.63E-11	1.42E-10
55180	2559	LINS1	0.70	8.11E-06	2.66E-05
4515	2329	MTCP1	0.70	0.009873705	0.0200338
729440	2017	CCDC61	0.70	5.07E-06	1.71E-05
148266	4016	ZNF569	0.70	0.000634999	0.001599244
63915	2673	BLOC1S5	0.70	9.20E-09	4.06E-08

132001	1527	TAMM41	0.70	0.000233122	0.000627117
64773	1993	PCED1A	0.70	4.75E-14	3.11E-13
10282	1523	BET1	0.69	8.78E-12	4.87E-11
130502	948	TTC32	0.69	0.000723219	0.001806065
9463	2099	PICK1	0.69	5.75E-11	3.03E-10
8682	2444	PEA15	0.69	6.00E-22	5.93E-21
56063	1922	TMEM234	0.69	1.33E-07	5.26E-07
6041	4258	RNASEL	0.69	2.51E-05	7.73E-05
4695	729	NDUFA2	0.69	0.001737418	0.00405323
22889	3009	KHDC4	0.69	1.97E-10	9.90E-10
546	10339	ATRX	0.69	2.08E-24	2.30E-23
4259	779	MGST3	0.69	3.81E-18	3.15E-17
56288	5165	PARD3	0.69	8.73E-24	9.41E-23
6282	595	S100A11	0.69	2.14E-13	1.33E-12
134145	2276	FAM173B	0.69	1.47E-06	5.22E-06
90362	3691	FAM110B	0.69	0.000152962	0.000423471
9411	5506	ARHGAP29	0.69	7.14E-27	8.87E-26
1E+08	3356	ZNF587B	0.69	0.000242464	0.000651066
27152	2292	INTU	0.69	0.000511493	0.001307858
9849	8330	ZNF518A	0.69	5.33E-12	3.00E-11
254887	4877	ZDHHC23	0.69	1.23E-07	4.88E-07
4683	4513	NBN	0.69	8.74E-17	6.73E-16
435	1947	ASL	0.69	3.35E-09	1.53E-08
617	1891	BCS1L	0.69	9.65E-08	3.86E-07
57186	9420	RALGAPA2	0.68	1.03E-11	5.67E-11
55589	6244	BMP2K	0.68	3.56E-16	2.64E-15
9158	1433	FIBP	0.68	1.88E-15	1.34E-14
1836	8053	SLC26A2	0.68	2.86E-17	2.27E-16
26094	2579	DCAF4	0.68	3.14E-07	1.20E-06
53916	1200	RAB4B	0.68	1.96E-05	6.15E-05
10186	2135	LHFPL6	0.68	5.43E-10	2.64E-09
23536	5103	ADAT1	0.68	4.14E-14	2.72E-13
9581	4952	PREPL	0.68	1.42E-14	9.57E-14
283234	3516	CCDC88B	0.68	0.000191283	0.000521676
5817	5633	PVR	0.68	7.77E-21	7.32E-20
8908	3177	GYG2	0.68	5.69E-05	0.0001669
4327	3294	MMP19	0.68	2.47E-05	7.62E-05
93627	5023	TBCK	0.68	3.56E-08	1.49E-07
1845	4139	DUSP3	0.68	2.34E-23	2.47E-22
29984	1145	RHOD	0.68	2.36E-09	1.09E-08
7127	4311	TNFAIP2	0.68	7.29E-17	5.64E-16
1666	1170	DECR1	0.68	1.40E-10	7.13E-10
54822	7535	TRPM7	0.68	6.88E-15	4.73E-14
54741	4474	LEPROT	0.68	1.96E-23	2.07E-22
801	4282	CALM1	0.68	1.31E-24	1.47E-23
92565	1809	FANK1	0.68	0.003953949	0.008653008
373156	1090	GSTK1	0.68	2.85E-19	2.49E-18
51248	1233	PDZD11	0.68	3.31E-11	1.78E-10

25994	2785	HIGD1A	0.68	7.46E-18	6.09E-17
3433	3505	IFIT2	0.68	1.43E-06	5.08E-06
116984	7429	ARAP2	0.68	8.57E-09	3.79E-08
80833	2155	APOL3	0.68	0.002407756	0.005472817
1119	3168	CHKA	0.68	1.02E-10	5.25E-10
5252	2136	PHF1	0.68	4.20E-15	2.92E-14
10811	1621	NOXA1	0.68	1.63E-05	5.15E-05
80778	2816	ZNF34	0.68	0.000397457	0.001035487
91319	2136	DERL3	0.67	9.59E-06	3.12E-05
97	643	ACYP1	0.67	0.000331433	0.000873078
4832	1041	NME3	0.67	3.79E-08	1.58E-07
4886	3111	NPY1R	0.67	4.50E-07	1.69E-06
55297	2412	CCDC91	0.67	9.01E-11	4.67E-10
91355	2477	LRP5L	0.67	0.000827955	0.002044344
342184	11396	FMN1	0.67	1.09E-09	5.15E-09
10781	3573	ZNF266	0.67	4.44E-12	2.51E-11
2805	2140	GOT1	0.67	6.66E-14	4.31E-13
54677	2559	CROT	0.67	1.54E-06	5.46E-06
866	1510	SERPINA6	0.67	0.020403453	0.038777254
1E+08	3158	NEMP2	0.67	4.04E-08	1.68E-07
127602	2591	DNAH14	0.67	0.000194393	0.000529669
5074	5862	PAWR	0.67	6.30E-15	4.35E-14
1028	1512	CDKN1C	0.67	3.26E-10	1.61E-09
64429	1645	ZDHHC6	0.67	5.18E-16	3.80E-15
79717	2080	PPCS	0.67	7.78E-13	4.63E-12
490	6194	ATP2B1	0.67	3.39E-20	3.09E-19
2647	590	BLOC1S1	0.67	5.59E-11	2.95E-10
84976	4803	DISP1	0.67	7.28E-06	2.41E-05
57106	1368	NAT14	0.67	1.70E-11	9.22E-11
30833	967	NT5C	0.67	1.23E-09	5.81E-09
116985	4873	ARAP1	0.67	9.05E-22	8.89E-21
4130	10308	MAP1A	0.67	1.09E-06	3.91E-06
192286	628	HIGD2A	0.67	5.21E-12	2.94E-11
151050	3828	KANSL1L	0.67	7.64E-07	2.79E-06
338382	2476	RAB7B	0.67	0.004542008	0.009851366
79073	2412	TMEM109	0.67	2.36E-23	2.48E-22
51503	1455	CWC15	0.67	8.77E-16	6.37E-15
440026	3954	TMEM41B	0.67	9.10E-14	5.82E-13
729830	10266	FAM160A1	0.67	4.58E-07	1.72E-06
140680	1611	C20orf96	0.67	0.00022911	0.000617166
3727	1963	JUND	0.67	5.00E-12	2.82E-11
8809	3753	IL18R1	0.67	0.004862684	0.010484184
3122	1312	HLA-DRA	0.67	0.000234236	0.000630019
1.08E+08	3534	LOC107985971	0.66	0.000384839	0.001004675
85007	2347	PHYKPL	0.66	9.99E-10	4.76E-09
23261	1073	CAMTA1	0.66	6.63E-15	4.56E-14
2197	579	FAU	0.66	3.51E-13	2.14E-12
51097	2482	SCCPDH	0.66	1.30E-18	1.10E-17

54487	3743	DGCR8	0.66	2.34E-15	1.65E-14
10413	5304	YAP1	0.66	7.22E-20	6.51E-19
84102	4481	SLC41A2	0.66	2.92E-09	1.34E-08
10435	2049	CDC42EP2	0.66	3.74E-06	1.27E-05
3174	3894	HNF4G	0.66	0.009313571	0.018992715
4601	3219	MXI1	0.66	9.29E-11	4.81E-10
89894	1656	TMEM116	0.66	0.001591816	0.003738716
388121	2020	TNFAIP8L3	0.66	0.00629471	0.013276877
4919	5211	ROR1	0.66	1.87E-07	7.30E-07
54953	3478	ODR4	0.66	8.17E-11	4.25E-10
3315	914	HSPB1	0.66	1.89E-11	1.03E-10
135398	2364	C6orf141	0.66	0.01218119	0.024239599
1.01E+08	5821	OCLN	0.66	5.50E-09	2.47E-08
23250	8683	ATP11A	0.66	2.41E-13	1.49E-12
400569	878	MED11	0.66	9.32E-06	3.03E-05
64135	3617	IFIH1	0.66	6.95E-07	2.55E-06
582	3370	BBS1	0.66	4.94E-11	2.62E-10
6322	3194	SCML1	0.66	1.85E-10	9.35E-10
5797	4855	PTPRM	0.66	1.46E-18	1.23E-17
3084	3498	NRG1	0.66	1.18E-05	3.81E-05
51569	2735	UFM1	0.66	4.32E-18	3.56E-17
145957	3928	NRG4	0.66	0.002557983	0.005789841
7741	2622	ZSCAN26	0.66	8.40E-07	3.06E-06
2335	8291	FN1	0.66	5.93E-29	8.05E-28
64430	5482	PCNX4	0.66	1.14E-17	9.21E-17
1.01E+08	2130	BCL2L2-PABPN1	0.66	8.90E-19	7.58E-18
143879	4754	KBTBD3	0.66	0.002331831	0.005313835
26872	1330	STEAP1	0.66	0.002516815	0.005706107
2073	4091	ERCC5	0.66	1.97E-13	1.22E-12
26090	2057	ABHD12	0.66	6.94E-18	5.67E-17
159195	6387	USP54	0.66	1.08E-10	5.58E-10
8673	791	VAMP8	0.66	2.34E-10	1.17E-09
22981	4329	NINL	0.66	1.70E-09	7.94E-09
219931	4823	TPCN2	0.66	8.35E-08	3.37E-07
8473	6575	OGT	0.66	0.001745059	0.004068522
55692	2069	LUC7L	0.66	1.88E-13	1.17E-12
80034	11788	CSRNP3	0.66	0.008194517	0.01688118
55815	2244	TSNAXIP1	0.66	0.016892815	0.032764058
6932	2962	TCF7	0.65	2.48E-10	1.23E-09
573	3885	BAG1	0.65	8.05E-10	3.86E-09
55286	4205	C4orf19	0.65	1.49E-05	4.73E-05
83759	2199	RBM4B	0.65	2.32E-08	9.86E-08
5099	6223	PCDH7	0.65	5.51E-05	0.000162227
253152	1443	EPHX4	0.65	0.000995604	0.002427595
3912	5914	LAMB1	0.65	1.17E-22	1.20E-21
146330	3543	FBXL16	0.65	0.023503636	0.044089169
22992	5920	KDM2A	0.65	3.85E-17	3.02E-16

644538	1552	SMIM10	0.65	1.55E-07	6.09E-07
5789	8684	PTPRD	0.65	4.89E-13	2.94E-12
4582	1828	MUC1	0.65	0.002127851	0.004885972
475	498	ATOX1	0.65	2.53E-08	1.08E-07
84909	4448	C9orf3	0.65	1.59E-12	9.31E-12
3223	1837	HOXC6	0.65	2.84E-08	1.20E-07
3338	1318	DNAJC4	0.65	6.55E-08	2.67E-07
83547	1793	RILP	0.65	0.000583966	0.001481216
93058	1492	COQ10A	0.65	5.53E-06	1.85E-05
119392	1560	SFR1	0.65	7.96E-05	0.000229307
79729	3388	SH3D21	0.65	7.56E-05	0.00021856
115123	4188	MARCH3	0.65	0.000951694	0.002330123
55640	3137	FLVCR2	0.65	0.0007597	0.001890791
57522	8200	SRGAP1	0.65	7.52E-12	4.19E-11
9399	2052	STOML1	0.65	6.44E-07	2.37E-06
10313	2603	RTN3	0.65	7.66E-24	8.26E-23
25796	1045	PGLS	0.65	1.09E-11	6.00E-11
9904	4357	RBM19	0.65	1.50E-10	7.61E-10
421	3503	ARVCF	0.65	1.26E-07	5.00E-07
51315	1815	KRCC1	0.65	9.41E-09	4.15E-08
79661	1749	NEIL1	0.65	0.000305939	0.000809159
9988	3994	DMTF1	0.65	7.91E-11	4.12E-10
1690	2625	COCH	0.65	7.63E-10	3.66E-09
83982	469	IFI27L2	0.65	5.33E-09	2.40E-08
3914	4239	LAMB3	0.64	4.56E-09	2.06E-08
92912	3045	UBE2Q2	0.64	8.40E-13	4.99E-12
4354	1988	MPP1	0.64	1.02E-08	4.46E-08
2273	2315	FHL1	0.64	8.73E-17	6.72E-16
51619	3635	UBE2D4	0.64	1.43E-05	4.55E-05
1211	1105	CLTA	0.64	3.94E-14	2.59E-13
7108	1610	TM7SF2	0.64	1.08E-11	5.94E-11
116228	2546	COX20	0.64	3.23E-12	1.85E-11
57719	4906	ANO8	0.64	2.13E-09	9.90E-09
6576	1631	SLC25A1	0.64	7.74E-17	5.99E-16
6607	1595	SMN2	0.64	2.09E-12	1.21E-11
84069	2423	PLEKHN1	0.64	0.000661984	0.001662727
401052	1897	NA	0.64	0.009624608	0.019573083
6894	3379	TARBP1	0.64	1.65E-07	6.48E-07
2065	4939	ERBB3	0.64	0.00011287	0.000318464
7098	3947	TLR3	0.64	4.77E-07	1.78E-06
252839	1641	TMEM9	0.64	1.65E-14	1.11E-13
55243	7559	KIRREL1	0.64	3.62E-18	2.99E-17
79716	2211	NPEPL1	0.64	1.06E-11	5.87E-11
440498	766	HSBP1L1	0.64	8.35E-06	2.74E-05
79877	2020	DCAKD	0.64	2.25E-13	1.39E-12
79441	2239	HAUS3	0.64	2.38E-07	9.16E-07
493861	1486	EID3	0.64	0.00402827	0.008804811
51527	2265	GSKIP	0.64	8.21E-09	3.63E-08

154796	6621	AMOT	0.64	3.59E-09	1.64E-08
9569	3460	GTF2IRD1	0.64	3.30E-14	2.18E-13
7533	1807	YWHAH	0.64	1.21E-17	9.80E-17
4728	826	NDUFS8	0.64	1.02E-14	6.92E-14
2274	1571	FHL2	0.64	1.88E-10	9.46E-10
124989	3690	EFCAB13	0.64	0.00257766	0.005832148
6843	2866	VAMP1	0.64	3.09E-06	1.06E-05
414189	2154	AGAP6	0.64	1.70E-08	7.34E-08
153768	3625	PRELID2	0.64	1.61E-07	6.32E-07
684	1048	BST2	0.64	1.42E-06	5.05E-06
175	2112	AGA	0.64	1.10E-09	5.21E-09
644353	7227	ZCCHC18	0.64	0.001044861	0.002538294
8926	1556	SNURF	0.63	1.98E-10	9.95E-10
1.05E+08	1874	LOC105371303	0.63	0.000213899	0.000578382
55328	1709	RNLS	0.63	3.58E-05	0.000108136
54497	5807	HEATR5B	0.63	1.23E-09	5.81E-09
121260	2785	SLC15A4	0.63	7.88E-11	4.11E-10
79800	4873	CARF	0.63	0.000135761	0.000378266
414918	2608	DENND6B	0.63	9.24E-05	0.000263788
84141	1866	EVA1A	0.63	7.52E-07	2.75E-06
390940	1517	PINLYP	0.63	0.010177449	0.020596005
647024	6742	C6orf132	0.63	1.11E-07	4.44E-07
4139	4502	MARK1	0.63	3.42E-05	0.000103539
55651	863	NHP2	0.63	2.84E-11	1.53E-10
90121	1353	TSR2	0.63	1.12E-08	4.89E-08
6387	2450	CXCL12	0.63	0.00041367	0.001074731
23167	5390	EFR3A	0.63	1.47E-16	1.12E-15
55805	4757	LRP2BP	0.63	0.017255778	0.033383219
79969	2470	ATAT1	0.63	8.39E-06	2.75E-05
5058	3100	PAK1	0.63	7.25E-16	5.29E-15
5892	2311	RAD51D	0.63	1.48E-05	4.71E-05
3182	1791	HNRNPAB	0.63	8.96E-22	8.81E-21
10087	5306	COL4A3BP	0.63	2.19E-13	1.35E-12
6251	3755	RSU1	0.63	2.27E-14	1.52E-13
125988	897	C19orf70	0.63	3.60E-08	1.51E-07
57696	2694	DDX55	0.63	1.19E-13	7.53E-13
51260	1213	PBDC1	0.63	2.45E-08	1.04E-07
2776	5846	GNAQ	0.63	3.87E-20	3.52E-19
51678	8128	MPP6	0.63	5.61E-09	2.51E-08
55500	6453	ETNK1	0.63	4.70E-11	2.49E-10
60682	3197	SMAP1	0.63	3.49E-12	1.99E-11
6638	1575	SNRPN	0.63	2.63E-10	1.31E-09
4649	10869	MYO9A	0.63	7.11E-14	4.58E-13
594	2650	BCKDHB	0.63	4.58E-11	2.44E-10
56339	2016	METTL3	0.63	5.28E-10	2.57E-09
57396	2524	CLK4	0.63	1.25E-05	4.00E-05
140576	1235	S100A16	0.63	8.15E-13	4.85E-12
57519	15136	STARD9	0.63	1.02E-08	4.50E-08

55733	3200	HHAT	0.63	1.87E-07	7.29E-07
254359	1361	ZDHHC24	0.63	5.63E-05	0.000165275
10336	6071	PCGF3	0.63	2.43E-11	1.32E-10
6737	1946	TRIM21	0.63	8.23E-07	3.00E-06
6094	1894	ROM1	0.63	0.008385783	0.017245208
200844	2950	C3orf67	0.63	0.000959964	0.002346491
839	1650	CASP6	0.63	2.14E-08	9.14E-08
10806	2635	SDCCAG8	0.63	6.80E-07	2.50E-06
4660	7164	PPP1R12B	0.63	1.88E-06	6.59E-06
3693	3450	ITGB5	0.63	1.09E-15	7.89E-15
329	3037	BIRC2	0.63	8.32E-17	6.41E-16
4478	3981	MSN	0.63	3.22E-14	2.13E-13
3280	1475	HES1	0.63	1.67E-06	5.91E-06
8464	2901	SUPT3H	0.63	7.04E-06	2.33E-05
5137	6799	PDE1C	0.62	0.000445165	0.0011505
57224	7165	NHSL1	0.62	2.37E-07	9.15E-07
837	1319	CASP4	0.62	2.41E-13	1.49E-12
858	3114	CAV2	0.62	8.37E-16	6.08E-15
23670	6156	TMEM2	0.62	1.06E-19	9.50E-19
133418	4259	EMB	0.62	1.42E-08	6.17E-08
139596	2518	UPRT	0.62	7.04E-05	0.000204221
217	2074	ALDH2	0.62	5.45E-12	3.06E-11
146705	2777	TEPSIN	0.62	1.13E-06	4.06E-06
112495	957	GTF3C6	0.62	1.05E-11	5.79E-11
9021	2737	SOCS3	0.62	1.71E-15	1.22E-14
4208	5816	MEF2C	0.62	0.010610054	0.021383785
5495	2940	PPM1B	0.62	3.12E-15	2.19E-14
92675	1428	DTD1	0.62	1.64E-11	8.93E-11
51188	1063	SS18L2	0.62	1.90E-07	7.40E-07
387680	3492	WASHC2A	0.62	1.87E-14	1.25E-13
79629	1090	OCEL1	0.62	2.63E-06	9.10E-06
54470	1874	ARMCX6	0.62	4.58E-06	1.55E-05
11034	1887	DSTN	0.62	3.12E-21	2.98E-20
195827	1188	AAED1	0.62	0.000193529	0.000527395
57664	3048	PLEKHA4	0.62	1.11E-12	6.52E-12
9770	5462	RASSF2	0.62	2.90E-05	8.86E-05
11284	1721	PNKP	0.62	2.09E-11	1.13E-10
29896	2107	TRA2A	0.62	2.62E-15	1.85E-14
3490	1136	IGFBP7	0.62	6.28E-17	4.88E-16
23507	6648	LRRC8B	0.62	2.34E-06	8.13E-06
4437	4472	MSH3	0.62	8.51E-11	4.42E-10
651746	8935	ANKRD33B	0.62	1.64E-15	1.17E-14
79869	3598	CPSF7	0.62	1.94E-14	1.30E-13
10725	13034	NFAT5	0.62	0.000801977	0.001984891
219749	4111	ZNF25	0.62	3.65E-05	0.00011001
54985	784	HCFC1R1	0.61	5.89E-08	2.42E-07
1.01E+08	7433	LOC100996517	0.61	0.00981387	0.019921251
8542	2883	APOL1	0.61	2.62E-19	2.30E-18

51078	1237	THAP4	0.61	7.16E-12	3.99E-11
6646	5636	SOAT1	0.61	5.04E-18	4.14E-17
10160	4784	FARP1	0.61	1.59E-20	1.47E-19
55851	832	PSENEN	0.61	3.06E-11	1.64E-10
7046	6404	TGFBR1	0.61	5.67E-13	3.39E-12
8038	7374	ADAM12	0.61	4.61E-06	1.56E-05
3958	1016	LGALS3	0.61	3.15E-15	2.21E-14
2941	1347	GSTA4	0.61	0.004643929	0.010060147
51634	1528	RBMX2	0.61	1.41E-09	6.64E-09
27250	3642	PDCD4	0.61	6.11E-15	4.21E-14
8263	1720	F8A1	0.61	0.002695572	0.006077264
1120	1648	CHKB	0.61	4.85E-09	2.19E-08
1838	3024	DTNB	0.61	9.99E-10	4.76E-09
57179	2627	KIAA1191	0.61	2.57E-17	2.04E-16
283229	2354	CRACR2B	0.61	0.009768499	0.019838461
1196	2441	CLK2	0.61	6.78E-11	3.55E-10
1.02E+08	899	LOC101929950	0.61	0.001658328	0.003883126
51257	1306	MARCH2	0.61	2.00E-06	7.02E-06
56951	2298	C5orf15	0.61	2.81E-21	2.70E-20
1E+08	349	PET100	0.61	5.23E-07	1.95E-06
9324	1001	HMGN3	0.61	5.47E-15	3.78E-14
2483	1006	FRG1	0.61	2.21E-12	1.28E-11
1487	2921	CTBP1	0.61	5.22E-21	4.95E-20
27166	1287	PRELID1	0.61	1.41E-13	8.86E-13
83856	6792	FSD1L	0.61	0.000127085	0.000355152
114134	6813	SLC2A13	0.61	1.42E-06	5.06E-06
5331	5564	PLCB3	0.61	9.33E-15	6.37E-14
5498	1914	PPOX	0.61	7.61E-06	2.51E-05
64097	4732	EPB41L4A	0.61	4.17E-09	1.89E-08
57018	3029	CCNL1	0.61	5.71E-11	3.02E-10
8805	3946	TRIM24	0.61	3.82E-11	2.04E-10
55937	722	APOM	0.61	0.01778956	0.03431642
11015	1712	KDELR3	0.61	1.24E-13	7.87E-13
91050	4003	CCDC149	0.60	1.53E-05	4.84E-05
9326	1142	ZNHIT3	0.60	7.85E-09	3.48E-08
1E+08	4438	ZNF783	0.60	4.52E-05	0.000134759
205428	3295	C3orf58	0.60	4.94E-07	1.84E-06
6239	8378	RREB1	0.60	1.49E-09	6.97E-09
150472	2725	CBWD2	0.60	2.33E-07	8.99E-07
8820	1901	HESX1	0.60	0.020177514	0.038384805
84443	6798	FRMPD3	0.60	0.023603472	0.044239081
154141	2847	MBOAT1	0.60	1.34E-09	6.30E-09
10513	6107	APPBP2	0.60	2.24E-16	1.68E-15
8837	7277	CFLAR	0.60	4.73E-13	2.85E-12
826	1233	CAPNS1	0.60	5.39E-11	2.85E-10
79088	7125	ZNF426	0.60	1.36E-07	5.39E-07
40	3470	ASIC2	0.60	0.013946985	0.027467306
219	3084	ALDH1B1	0.60	3.74E-09	1.70E-08

80021	2150	TMEM62	0.60	9.03E-06	2.94E-05
55509	987	BATF3	0.60	0.002617563	0.005912645
64777	1882	RMND5B	0.60	1.73E-09	8.10E-09
124817	3254	CNTD1	0.60	0.012476497	0.024782828
23530	4443	NNT	0.60	1.18E-15	8.52E-15
404672	7502	GTF2H5	0.60	1.14E-08	5.00E-08
1130	12631	LYST	0.60	2.61E-06	9.03E-06
1E+08	18450	NBPF20	0.60	2.13E-08	9.09E-08
7453	2674	WARS	0.60	9.05E-16	6.57E-15
150223	1361	YDJC	0.60	1.76E-07	6.87E-07
5092	1014	PCBD1	0.60	1.39E-10	7.08E-10
115290	2289	FBXO17	0.60	1.19E-12	6.99E-12
58155	3247	PTBP2	0.60	1.47E-10	7.46E-10
1.02E+08	977	AK6	0.60	8.83E-08	3.55E-07
9669	4698	EIF5B	0.60	1.51E-19	1.34E-18
79874	2280	RABEP2	0.60	8.62E-07	3.14E-06
9986	1488	RCE1	0.60	8.72E-09	3.85E-08
10982	4281	MAPRE2	0.60	3.69E-10	1.81E-09
7905	3115	REEP5	0.60	2.57E-20	2.35E-19
445571	3084	CBWD3	0.60	7.13E-07	2.61E-06
55298	2563	RNF121	0.60	4.05E-11	2.16E-10
9853	5402	RUSC2	0.60	2.35E-13	1.45E-12
23331	11808	TTC28	0.60	1.09E-10	5.60E-10
9863	6688	MAGI2	0.60	0.017002059	0.032947168
162966	3241	ZNF600	0.60	0.000248253	0.000665704
11019	1596	LIAS	0.60	0.000761909	0.001896022
23022	4503	PALLD	0.60	1.47E-18	1.24E-17
125061	1448	AFMID	0.60	3.73E-06	1.27E-05
3304	2551	HSPA1B	0.60	3.57E-17	2.82E-16
113179	1643	ADAT3	0.60	0.007785984	0.016123701
7597	2622	ZBTB25	0.60	1.65E-05	5.20E-05
84062	1225	DTNBP1	0.60	4.20E-08	1.75E-07
3705	3325	ITPK1	0.60	1.77E-09	8.27E-09
55199	2053	FAM86C1	0.59	0.000724174	0.001808196
143684	3871	FAM76B	0.59	8.81E-11	4.57E-10
63933	4586	MCUR1	0.59	1.17E-12	6.90E-12
55667	7822	DENND4C	0.59	7.08E-13	4.22E-12
4723	1630	NDUFV1	0.59	7.41E-16	5.40E-15
284349	6927	ZNF283	0.59	0.000293013	0.00077775
5305	3714	PIP4K2A	0.59	1.43E-10	7.29E-10
83463	1530	MXD3	0.59	1.84E-09	8.57E-09
972	1520	CD74	0.59	2.98E-19	2.61E-18
3187	3010	HNRNPH1	0.59	6.50E-11	3.42E-10
9552	1044	SPAG7	0.59	6.49E-09	2.90E-08
9440	3497	MED17	0.59	1.34E-08	5.83E-08
55599	1901	RNPC3	0.59	0.000115188	0.000324403
9984	2311	THOC1	0.59	1.56E-10	7.93E-10
1351	521	COX8A	0.59	2.58E-12	1.48E-11

55076	1634	TMEM45A	0.59	5.84E-05	0.000171281
84914	6945	ZNF587	0.59	7.32E-07	2.68E-06
521	373	ATP5ME	0.59	4.20E-07	1.58E-06
257415	2432	FAM133B	0.59	1.03E-09	4.89E-09
10553	1365	HTATIP2	0.59	2.25E-07	8.72E-07
2054	3326	STX2	0.59	6.39E-14	4.14E-13
170622	590	COMMD6	0.59	6.25E-08	2.56E-07
81889	1683	FAHD1	0.59	2.38E-10	1.19E-09
55780	2508	ERMARD	0.59	1.35E-06	4.82E-06
55596	3295	ZCCHC8	0.59	2.18E-12	1.26E-11
54093	3077	SETD4	0.59	1.10E-05	3.55E-05
90624	6240	LYRM7	0.58	1.55E-07	6.09E-07
3858	2162	KRT10	0.58	3.73E-07	1.41E-06
54882	7732	ANKHD1	0.58	4.12E-13	2.49E-12
140890	6841	SREK1	0.58	1.10E-13	7.01E-13
11107	5073	PRDM5	0.58	0.001418822	0.003367121
8605	2649	PLA2G4C	0.58	0.007083687	0.014784793
717	2384	C2	0.58	0.000261297	0.000698049
1663	3970	DDX11	0.58	1.89E-11	1.03E-10
9620	8437	CELSR1	0.58	1.28E-15	9.17E-15
3841	4640	KPNA5	0.58	8.54E-06	2.79E-05
25970	3211	SH2B1	0.58	2.49E-09	1.15E-08
201158	2493	TVP23C	0.58	0.002294176	0.005234068
23220	5434	DTX4	0.58	4.19E-05	0.00012537
90	2892	ACVR1	0.58	7.06E-09	3.14E-08
2495	1245	FTH1	0.58	1.80E-11	9.76E-11
57037	2569	ANKMY2	0.58	4.88E-08	2.02E-07
65997	1979	RASL11B	0.58	0.017040751	0.033005722
115817	1495	DHRS1	0.58	5.60E-06	1.88E-05
7692	2917	ZNF133	0.58	4.87E-05	0.000144672
152485	6186	ZNF827	0.58	6.61E-12	3.69E-11
377677	3855	CA13	0.58	0.000104218	0.000295477
53840	2255	TRIM34	0.58	0.000787908	0.001953881
5720	1024	PSME1	0.58	2.59E-15	1.83E-14
1892	1350	ECHS1	0.58	4.40E-12	2.49E-11
6692	2430	SPINT1	0.58	4.29E-10	2.10E-09
30844	2904	EHD4	0.58	1.72E-10	8.70E-10
54883	3112	CWC25	0.58	2.75E-09	1.27E-08
115201	2321	ATG4A	0.58	4.93E-05	0.000146345
55118	2848	CRTAC1	0.58	0.01256311	0.024943718
1500	5880	CTNND1	0.58	7.77E-15	5.33E-14
3303	2445	HSPA1A	0.58	1.46E-14	9.83E-14
127262	2440	TPRG1L	0.58	0.000235959	0.000634461
3177	2458	SLC29A2	0.57	2.46E-06	8.54E-06
56965	2839	PARP6	0.57	8.59E-08	3.46E-07
54995	1948	OXSM	0.57	0.000266362	0.000711152
6578	4171	SLCO2A1	0.57	1.17E-07	4.67E-07
3340	7419	NDST1	0.57	3.77E-21	3.60E-20

51367	778	POP5	0.57	3.09E-05	9.43E-05
9260	1576	PDLIM7	0.57	5.45E-10	2.65E-09
84661	756	DPY30	0.57	1.61E-08	6.95E-08
2195	14805	FAT1	0.57	1.91E-20	1.77E-19
200030	5032	NBPF11	0.57	1.66E-05	5.23E-05
79038	1471	ZFYVE21	0.57	2.87E-08	1.21E-07
493753	1767	COA5	0.57	8.58E-07	3.12E-06
90736	1306	FAM104B	0.57	2.63E-05	8.08E-05
51586	3401	MED15	0.57	3.22E-12	1.84E-11
1294	9195	COL7A1	0.57	5.72E-10	2.77E-09
79944	5300	L2HGDH	0.57	9.36E-09	4.13E-08
11177	5585	BAZ1A	0.57	7.06E-16	5.15E-15
196074	4207	METTL15	0.57	7.94E-06	2.61E-05
29082	1372	CHMP4A	0.57	1.88E-07	7.32E-07
90102	5950	PHLDB2	0.57	2.47E-14	1.64E-13
10608	3212	MXD4	0.57	6.13E-11	3.23E-10
57415	1538	C3orf14	0.57	2.41E-05	7.47E-05
51773	6477	RSF1	0.57	1.91E-18	1.60E-17
30818	2761	KCNIP3	0.57	5.73E-10	2.77E-09
9686	3548	VGLL4	0.57	2.45E-12	1.41E-11
9488	1852	PIGB	0.57	0.000455408	0.001175094
8986	3000	RPS6KA4	0.57	2.16E-09	1.00E-08
9140	4324	ATG12	0.57	1.38E-11	7.54E-11
27032	4040	ATP2C1	0.57	8.63E-13	5.12E-12
54802	2142	TRIT1	0.57	4.13E-05	0.00012374
26298	5413	EHF	0.57	5.48E-10	2.66E-09
2247	6774	FGF2	0.57	1.76E-12	1.03E-11
55040	3944	EPN3	0.57	0.019717407	0.037622303
2683	4207	B4GALT1	0.57	4.92E-19	4.23E-18
57801	1077	HES4	0.57	1.66E-07	6.50E-07
57558	3434	USP35	0.57	0.000183416	0.00050176
114881	3452	OSBPL7	0.57	5.16E-06	1.73E-05
5261	4499	PHKG2	0.56	1.32E-09	6.23E-09
163486	7246	DENND1B	0.56	1.33E-06	4.75E-06
1E+08	877	SYCE1L	0.56	0.004633579	0.010040174
331	8416	XIAP	0.56	7.99E-15	5.48E-14
10207	7016	PATJ	0.56	1.59E-08	6.87E-08
145482	2561	PTGR2	0.56	2.44E-07	9.40E-07
9187	4021	SLC24A1	0.56	1.27E-05	4.08E-05
24148	3120	PRPF6	0.56	4.22E-11	2.25E-10
1495	3515	CTNNA1	0.56	3.88E-17	3.05E-16
10346	3111	TRIM22	0.56	4.55E-14	2.98E-13
340390	4978	WDR97	0.56	0.002059403	0.004739828
151636	5768	DTX3L	0.56	2.40E-12	1.38E-11
10658	6009	CELF1	0.56	1.82E-16	1.37E-15
85459	7906	CEP295	0.56	1.59E-10	8.04E-10
4155	4769	MBP	0.56	1.89E-07	7.35E-07
79660	5536	PPP1R3B	0.56	1.15E-08	5.01E-08

92421	1847	CHMP4C	0.56	0.000260023	0.000694958
9709	1943	HERPUD1	0.56	1.52E-13	9.52E-13
3295	2712	HSD17B4	0.56	9.98E-15	6.79E-14
64105	1919	CENPK	0.56	1.50E-12	8.76E-12
9910	5692	RABGAP1L	0.56	7.78E-09	3.45E-08
23086	10061	EXPH5	0.56	0.002063617	0.004748296
121642	1084	ALKBH2	0.56	0.000174419	0.000478548
80184	7937	CEP290	0.56	3.26E-09	1.49E-08
9755	4104	TBKBP1	0.56	1.00E-05	3.26E-05
117283	2717	IP6K3	0.56	1.16E-05	3.73E-05
9891	6821	NUAK1	0.56	8.89E-08	3.58E-07
5201	1323	PFDN1	0.56	2.01E-13	1.25E-12
150274	1021	HSCB	0.56	0.001402915	0.00333115
58500	4018	ZNF250	0.56	4.03E-05	0.00012102
10654	1277	PMVK	0.56	6.21E-07	2.30E-06
79169	1448	C1orf35	0.56	1.99E-06	6.97E-06
90634	2646	N4BP2L1	0.56	1.13E-05	3.65E-05
8815	900	BANF1	0.56	6.99E-11	3.66E-10
80198	2360	MUS81	0.56	7.40E-10	3.56E-09
55819	2323	RNF130	0.55	2.75E-09	1.27E-08
54851	1925	ANKRD49	0.55	5.48E-05	0.000161602
1.01E+08	2116	TMEM265	0.55	3.08E-05	9.40E-05
163131	7612	ZNF780B	0.55	0.000270812	0.000722383
23761	2693	PISD	0.55	6.21E-11	3.27E-10
9941	3744	EXOG	0.55	2.39E-07	9.22E-07
427	2576	ASAH1	0.55	6.81E-10	3.28E-09
79828	8012	METTL8	0.55	1.88E-05	5.91E-05
55109	4514	AGGF1	0.55	1.43E-10	7.27E-10
51256	1256	TBC1D7	0.55	1.50E-05	4.77E-05
55016	5405	MARCH1	0.55	0.000360372	0.00094413
1.01E+08	684	LOC100506248	0.55	6.11E-05	0.000178584
6284	664	S100A13	0.55	5.40E-08	2.22E-07
9555	2055	H2AFY	0.55	1.20E-18	1.02E-17
5438	885	POLR2I	0.55	6.25E-07	2.31E-06
116412	2233	ZNF837	0.55	0.021075936	0.039905929
8500	5091	PPFIA1	0.55	1.95E-12	1.13E-11
6571	3885	SLC18A2	0.55	0.000310669	0.000820691
285343	2816	TCAIM	0.55	7.78E-07	2.84E-06
374393	3476	FAM111B	0.55	4.90E-18	4.03E-17
2188	3309	FANCF	0.55	0.000188378	0.000514146
121512	7289	FGD4	0.55	3.16E-07	1.20E-06
340719	4036	NANOS1	0.55	0.010120864	0.020488494
5936	1910	RBM4	0.55	8.79E-15	6.01E-14
7697	2641	ZNF138	0.55	0.000325717	0.000858782
26002	2852	MOXD1	0.55	1.53E-14	1.03E-13
10534	790	SSSCA1	0.55	7.72E-06	2.54E-05
26272	1519	FBXO4	0.55	3.29E-05	9.98E-05
9848	6427	MFAP3L	0.55	0.001514875	0.003576443

2014	801	EMP3	0.55	1.94E-14	1.30E-13
388591	3970	RNF207	0.55	1.22E-05	3.93E-05
81537	3382	SGPP1	0.55	5.65E-10	2.74E-09
741	1358	ZNHIT2	0.55	0.001028218	0.0025006
80150	2305	ASRGL1	0.55	9.16E-11	4.75E-10
169841	7132	ZNF169	0.55	0.011753939	0.023447176
2896	2357	GRN	0.55	7.32E-17	5.66E-16
9789	2731	SPCS2	0.55	9.26E-16	6.71E-15
58504	2529	ARHGAP22	0.55	0.002719445	0.006123318
51382	1823	ATP6V1D	0.55	6.35E-12	3.56E-11
64342	2374	HS1BP3	0.55	6.52E-06	2.17E-05
55871	1725	CBWD1	0.55	1.11E-06	3.99E-06
9751	5086	SNPH	0.54	0.000103102	0.000292731
55337	2136	C19orf66	0.54	1.33E-08	5.79E-08
113146	18171	AHNAK2	0.54	6.63E-09	2.96E-08
3035	2280	HARS	0.54	1.08E-14	7.35E-14
388115	5344	CCDC9B	0.54	3.05E-08	1.28E-07
57404	6077	CYP20A1	0.54	4.30E-09	1.95E-08
10827	3451	FAM114A2	0.54	1.01E-06	3.65E-06
80195	2092	TMEM254	0.54	9.53E-05	0.000271527
3206	2648	HOXA10	0.54	2.04E-10	1.02E-09
81855	3129	SFXN3	0.54	1.37E-10	6.99E-10
2622	3179	GAS8	0.54	1.58E-06	5.59E-06
57205	6094	ATP10D	0.54	5.34E-10	2.60E-09
9295	3545	SRSF11	0.54	5.82E-12	3.26E-11
54518	2595	APBB1IP	0.54	0.002100775	0.004827545
120103	5579	SLC36A4	0.54	1.30E-08	5.64E-08
26519	684	TIMM10	0.54	6.51E-05	0.000189668
831	4147	CAST	0.54	6.80E-17	5.27E-16
4860	2438	PNP	0.54	3.19E-09	1.46E-08
57210	6591	SLC45A4	0.54	0.000606226	0.00153199
10360	904	NPM3	0.54	0.000252589	0.000676821
1.01E+08	2341	NDUFC2-KCTD14	0.54	2.47E-07	9.48E-07
23015	4489	GOLGA8A	0.54	0.014021858	0.027596408
64065	4319	PERP	0.54	1.03E-13	6.58E-13
468	1486	ATF4	0.54	7.99E-20	7.19E-19
81671	2197	VMP1	0.54	3.88E-12	2.20E-11
79649	3555	MAP7D3	0.54	2.32E-10	1.16E-09
53838	1869	C11orf24	0.54	6.19E-07	2.29E-06
10589	1022	DRAP1	0.54	8.26E-11	4.30E-10
78999	2505	LRFN4	0.54	2.18E-09	1.01E-08
10542	855	LAMTOR5	0.54	1.39E-10	7.10E-10
6785	3085	ELOVL4	0.54	0.001308587	0.003122193
1486	3152	CTBS	0.54	1.99E-05	6.23E-05
27013	2106	CNPPD1	0.54	6.98E-08	2.84E-07
10610	2105	ST6GALNAC2	0.54	0.000684013	0.001714181
22927	2593	HABP4	0.54	7.08E-07	2.60E-06

282991	2637	BLOC1S2	0.54	2.03E-06	7.11E-06
6228	3325	RPS23	0.54	8.29E-10	3.96E-09
5631	2156	PRPS1	0.53	2.63E-10	1.31E-09
3675	4764	ITGA3	0.53	4.12E-14	2.71E-13
137075	1972	CLDN23	0.53	0.023136368	0.043436918
23061	5141	TBC1D9B	0.53	2.20E-15	1.56E-14
440574	3728	MINOS1	0.53	7.17E-09	3.19E-08
57118	6893	CAMK1D	0.53	2.32E-06	8.07E-06
202	7562	CRYBG1	0.53	4.36E-06	1.48E-05
55974	1278	SLC50A1	0.53	1.17E-07	4.65E-07
124446	1014	TMEM219	0.53	7.58E-09	3.37E-08
57605	6804	PITPNM2	0.53	4.30E-06	1.46E-05
85359	1217	DGCR6L	0.53	6.18E-07	2.29E-06
64852	2844	TUT1	0.53	8.91E-07	3.24E-06
1515	4539	CTSV	0.53	9.38E-08	3.76E-07
821	4316	CANX	0.53	6.06E-24	6.57E-23
84267	2431	C9orf64	0.53	2.19E-06	7.65E-06
259282	10579	BOD1L1	0.53	1.65E-15	1.18E-14
71	2005	ACTG1	0.53	3.00E-14	1.99E-13
51022	699	GLRX2	0.53	0.000715834	0.001789635
54832	13321	VPS13C	0.53	6.48E-10	3.13E-09
6574	3363	SLC20A1	0.53	7.37E-15	5.06E-14
1783	4251	DYNC1LI2	0.53	3.27E-14	2.16E-13
55152	1849	DALRD3	0.53	1.55E-06	5.48E-06
11337	924	GABARAP	0.53	5.04E-13	3.03E-12
6643	6566	SNX2	0.53	6.13E-12	3.43E-11
84133	6570	ZNRF3	0.53	3.01E-07	1.15E-06
378108	1675	TRIM74	0.53	0.000177437	0.000486302
1.03E+08	937	SMIM11B	0.53	0.000170749	0.000468914
7049	6338	TGFBR3	0.53	0.000221272	0.00059741
2821	4211	GPI	0.53	1.09E-19	9.71E-19
51101	3461	ZC2HC1A	0.53	8.60E-06	2.82E-05
3033	1984	HADH	0.53	6.49E-09	2.90E-08
64787	3165	EPS8L2	0.53	6.02E-17	4.69E-16
57462	6455	MYORG	0.53	4.49E-07	1.69E-06
27018	1038	BEX3	0.53	5.17E-12	2.91E-11
207063	2596	DHRSX	0.52	8.79E-05	0.000251719
83452	3899	RAB33B	0.52	6.97E-06	2.31E-05
83451	1563	ABHD11	0.52	6.52E-08	2.66E-07
130340	4005	AP1S3	0.52	6.05E-08	2.48E-07
7405	5154	UVRAG	0.52	2.05E-06	7.17E-06
93663	3735	ARHGAP18	0.52	2.00E-10	1.01E-09
404734	8349	ANKHD1-EIF4EBP3	0.52	5.71E-09	2.56E-08
10524	2166	KAT5	0.52	1.09E-08	4.76E-08
65009	3146	NDRG4	0.52	3.35E-05	0.000101614
9902	6004	MRC2	0.52	1.58E-19	1.40E-18
4791	3158	NFKB2	0.52	1.13E-09	5.36E-09

84233	811	TMEM126A	0.52	0.000463601	0.00119398
113277	3266	TMEM106A	0.52	0.008063739	0.016632983
10884	1686	MRPS30	0.52	1.45E-06	5.15E-06
79675	4537	FASTKD1	0.52	6.88E-06	2.28E-05
79631	3671	EFL1	0.52	8.15E-08	3.29E-07
90293	3215	KLHL13	0.52	2.30E-07	8.91E-07
9020	4499	MAP3K14	0.52	2.20E-06	7.69E-06
1185	5699	CLCN6	0.52	2.46E-05	7.60E-05
83930	1752	STARD3NL	0.52	5.10E-07	1.90E-06
161582	1930	DNAAF4	0.52	0.001208994	0.00290564
80014	7796	WWC2	0.52	1.57E-12	9.16E-12
23262	5579	PPIP5K2	0.52	1.04E-09	4.96E-09
39	1569	ACAT2	0.52	2.21E-08	9.43E-08
83638	1568	C11orf68	0.52	2.13E-05	6.63E-05
10857	1930	PGRMC1	0.52	1.68E-13	1.05E-12
80317	4509	ZKSCAN3	0.52	0.004881198	0.010521548
339487	1379	ZBTB8OS	0.52	3.65E-06	1.25E-05
54460	1256	MRPS21	0.52	1.11E-09	5.25E-09
7265	1499	TTC1	0.52	1.66E-10	8.40E-10
128710	6103	SLX4IP	0.52	0.000290263	0.000771141
90864	1559	SPSB3	0.52	5.38E-09	2.42E-08
9907	3630	AP5Z1	0.52	1.45E-07	5.71E-07
4595	1635	MUTYH	0.52	0.000452849	0.001169
80152	2290	CENPT	0.52	2.53E-10	1.26E-09
6897	2803	TARS	0.52	3.39E-15	2.38E-14
256364	3233	EML3	0.52	1.57E-08	6.78E-08
7629	2670	ZNF76	0.52	6.22E-08	2.54E-07
81669	3783	CCNL2	0.52	1.41E-09	6.64E-09
4669	2348	NAGLU	0.52	5.16E-08	2.13E-07
6470	2415	SHMT1	0.52	1.98E-16	1.49E-15
23376	4219	UFL1	0.52	1.61E-09	7.56E-09
163033	2173	ZNF579	0.52	0.000349078	0.000916161
2629	2322	GBA	0.52	5.62E-14	3.66E-13
2098	1228	ESD	0.52	2.36E-10	1.18E-09
8718	1537	TNFRSF25	0.52	0.00110765	0.002678744
27037	2739	TRMT2A	0.52	5.50E-09	2.47E-08
445	1605	ASS1	0.52	0.013649208	0.026922639
114883	2788	OSBPL9	0.52	5.05E-14	3.31E-13
8462	3972	KLF11	0.52	1.57E-07	6.18E-07
60487	1936	TRMT11	0.52	0.000178718	0.000489511
7320	2634	UBE2B	0.52	1.62E-10	8.18E-10
285440	4657	CYP4V2	0.52	0.000486592	0.001247769
81622	2214	UNC93B1	0.52	7.38E-09	3.28E-08
8774	3503	NAPG	0.52	1.82E-07	7.11E-07
1.08E+08	2691	LOC107984138	0.51	0.000824846	0.002038084
126133	3107	ALDH16A1	0.51	3.52E-09	1.60E-08
528	5704	ATP6V1C1	0.51	1.50E-11	8.17E-11
81576	1797	CCDC130	0.51	8.60E-06	2.82E-05

4752	2396	NEK3	0.51	0.002204239	0.00504493
129285	3180	PPP1R21	0.51	0.000100799	0.000286469
51491	1219	NOP16	0.51	1.89E-05	5.92E-05
51389	1473	RWDD1	0.51	5.29E-09	2.38E-08
957	5317	ENTPD5	0.51	0.000114256	0.000321881
81	3946	ACTN4	0.51	6.20E-17	4.82E-16
54587	2919	MXRA8	0.51	0.000541627	0.001379337
9556	675	ATP5MPL	0.51	5.57E-08	2.29E-07
5357	3734	PLS1	0.51	2.61E-07	1.00E-06
135932	2177	TMEM139	0.51	1.86E-05	5.84E-05
7318	3250	UBA7	0.51	1.32E-08	5.73E-08
1E+08	5850	ZNF316	0.51	1.69E-07	6.64E-07
8987	2964	STBD1	0.51	0.001679624	0.003928853
1E+08	1042	CMC4	0.51	0.000460948	0.001188181
728340	1560	GTF2H2C	0.51	2.89E-05	8.83E-05
91283	1810	MSANTD3	0.51	5.49E-06	1.84E-05
51559	6965	NT5DC3	0.51	0.000292094	0.000775542
3725	3338	JUN	0.51	7.65E-11	4.00E-10
9873	4496	FCHSD2	0.51	3.12E-09	1.43E-08
8440	2182	NCK2	0.51	2.00E-07	7.79E-07
8569	2993	MKNK1	0.51	9.26E-08	3.72E-07
6919	1773	TCEA2	0.51	5.92E-05	0.000173373
5575	2507	PRKAR1B	0.51	5.09E-05	0.000150842
51175	2314	TUBE1	0.51	0.001077304	0.002612465
23181	6725	DIP2A	0.51	6.01E-08	2.46E-07
9	1905	NAT1	0.51	0.012920986	0.025594213
1535	824	CYBA	0.51	7.55E-14	4.85E-13
10916	2171	MAGED2	0.51	1.17E-08	5.12E-08
150678	456	COPS9	0.51	0.001215381	0.002917964
53344	6813	CHIC1	0.51	0.00014989	0.000415937
55669	4927	MFN1	0.51	4.45E-10	2.17E-09
29954	4804	POMT2	0.50	3.57E-06	1.22E-05
79979	4262	TRMT2B	0.50	2.92E-06	1.01E-05
7581	6141	ZNF33A	0.50	3.85E-06	1.31E-05
28512	4006	NKIRAS1	0.50	0.000196993	0.000536014
51660	1004	MPC1	0.50	8.83E-05	0.000252824
275	2115	AMT	0.50	0.023867383	0.04467121
54468	3882	MIOS	0.50	3.69E-08	1.54E-07
124936	1501	CYB5D2	0.50	1.22E-05	3.93E-05
54981	1608	NMRK1	0.50	0.001009513	0.002459147
57820	1425	CCNB1IP1	0.50	1.88E-06	6.62E-06
950	4769	SCARB2	0.50	2.41E-16	1.81E-15
83666	3566	PARP9	0.50	2.04E-07	7.94E-07
51330	1048	TNFRSF12A	0.50	3.25E-12	1.86E-11
10352	2751	WARS2	0.50	2.11E-05	6.57E-05
93349	2578	SP140L	0.50	1.14E-07	4.55E-07
643155	2895	SMIM15	0.50	1.23E-10	6.29E-10
3422	3017	IDI1	0.50	4.36E-09	1.97E-08

148327	1676	CREB3L4	0.50	0.000587505	0.001489552
9540	1637	TP53I3	0.50	6.67E-07	2.45E-06
93185	2291	IGSF8	0.50	6.96E-08	2.83E-07
91289	2662	LMF2	0.50	2.90E-15	2.05E-14
79777	2303	ACBD4	0.50	0.000123885	0.0003467
64834	1527	ELOVL1	0.50	4.42E-09	2.00E-08
57176	3524	VARS2	0.50	5.46E-10	2.65E-09
128	2652	ADH5	0.50	3.72E-12	2.12E-11
132158	2467	GLYCTK	0.50	0.006249314	0.013192089
115426	3126	UHRF2	0.50	6.76E-08	2.75E-07
23474	971	ETHE1	0.50	8.10E-05	0.000233136
83933	2717	HDAC10	0.50	1.52E-05	4.83E-05
256586	1360	LYSMD2	0.50	0.020831475	0.039497776
738	2761	VPS51	0.50	1.33E-09	6.28E-09
2589	3856	GALNT1	0.50	1.91E-11	1.04E-10
6666	4658	SOX12	0.50	7.42E-09	3.29E-08
23091	7118	ZC3H13	0.50	3.80E-14	2.50E-13
79783	1578	SUGCT	0.50	0.007572411	0.015710718
10966	1863	RAB40B	0.50	0.001291973	0.003085453
55690	4154	PACS1	0.50	4.91E-08	2.03E-07
57449	4737	PLEKHG5	0.50	3.13E-06	1.07E-05
2803	7582	GOLGA4	0.50	1.31E-09	6.17E-09
2055	6134	CLN8	0.50	0.001745674	0.004069423
112	6118	ADCY6	0.50	2.08E-09	9.65E-09
239	4182	ALOX12	0.50	0.011328045	0.02268929
284184	560	NDUFAF8	0.50	2.09E-05	6.52E-05
168507	15565	PKD1L1	0.50	0.003263327	0.007247849
81894	1627	SLC25A28	0.50	1.76E-06	6.19E-06
1E+08	1951	ZNF717	0.50	0.015253314	0.029818558
51065	1084	RPS27L	0.50	2.21E-07	8.58E-07
94240	3176	EPSTI1	0.50	7.22E-05	0.000209172
55825	1874	PECR	0.49	7.10E-06	2.35E-05
389	1373	RHOC	0.49	2.30E-08	9.80E-08
6697	1466	SPR	0.49	6.37E-05	0.000185884
955	2739	ENTPD6	0.49	1.56E-07	6.13E-07
58478	2185	ENOPH1	0.49	7.17E-10	3.45E-09
64417	2825	TMEM267	0.49	0.000363675	0.000951917
84268	1663	RPAIN	0.49	5.40E-06	1.81E-05
55296	2949	TBC1D19	0.49	0.005410605	0.011561143
9445	1896	ITM2B	0.49	1.40E-16	1.06E-15
746	420	TMEM258	0.49	0.000124551	0.000348506
91137	4652	SLC25A46	0.49	1.05E-09	5.01E-09
57486	4729	NLN	0.49	3.20E-06	1.10E-05
1E+08	5633	SPDYE2B	0.49	0.002604092	0.005885209
338699	3553	ANKRD42	0.49	0.000503003	0.001287618
81853	984	TMEM14B	0.49	1.49E-09	6.97E-09
24145	4652	PANX1	0.49	2.76E-09	1.27E-08
23405	10296	DICER1	0.49	1.67E-08	7.21E-08

124790	1604	HEXIM2	0.49	0.00753447	0.015644789
79053	1708	ALG8	0.49	5.87E-08	2.41E-07
9667	3028	SAFB2	0.49	3.59E-11	1.92E-10
57458	5886	TMCC3	0.49	0.000722479	0.001804605
124565	3927	SLC38A10	0.49	8.65E-11	4.49E-10
953	12376	ENTPD1	0.49	2.23E-06	7.76E-06
388962	534	BOLA3	0.49	0.000520994	0.001330032
23446	9817	SLC44A1	0.49	2.73E-12	1.57E-11
54891	14048	INO80D	0.49	4.35E-06	1.47E-05
646	4585	BNC1	0.49	6.67E-05	0.000194167
23232	4423	TBC1D12	0.49	4.11E-05	0.000123143
51599	2190	LSR	0.49	4.16E-09	1.89E-08
23240	4428	TMEM131L	0.49	0.000848518	0.002092501
124512	1036	METTL23	0.49	0.000104032	0.000294994
81566	4351	CSRNP2	0.49	6.91E-08	2.82E-07
79155	1875	TNIP2	0.49	1.36E-05	4.35E-05
662	1259	BNIP1	0.49	0.003305385	0.007330264
55779	8285	CFAP44	0.49	0.000391704	0.001021397
2591	3326	GALNT3	0.49	0.000922662	0.002262782
26191	3678	PTPN22	0.48	0.000502851	0.001287415
50618	6031	ITSN2	0.48	9.07E-07	3.29E-06
1E+08	1360	UPK3BL1	0.48	0.000100563	0.000285842
4718	2490	NDUFC2	0.48	2.64E-10	1.31E-09
7582	6353	ZNF33B	0.48	4.32E-07	1.63E-06
9812	4107	KIAA0141	0.48	1.32E-07	5.24E-07
10252	2755	SPRY1	0.48	0.018858104	0.036134024
151393	1956	RMDN2	0.48	0.026986675	0.049968658
166614	4310	DCLK2	0.48	0.014695476	0.028813642
10928	4368	RALBP1	0.48	2.18E-11	1.18E-10
55625	2865	ZDHHC7	0.48	4.32E-12	2.45E-11
6895	1535	TARBP2	0.48	0.000204065	0.000553984
158158	5662	RASEF	0.48	3.38E-07	1.28E-06
51529	918	ANAPC11	0.48	0.000113921	0.000321141
4337	3634	MOCS1	0.48	4.36E-05	0.00013031
54205	5544	CYCS	0.48	4.98E-11	2.64E-10
9069	3641	CLDN12	0.48	4.35E-08	1.80E-07
28956	696	LAMTOR2	0.48	0.000167534	0.000460867
3212	1373	HOXB2	0.48	4.32E-07	1.63E-06
64418	4969	TMEM168	0.48	1.12E-05	3.60E-05
55142	3904	HAUS2	0.48	1.34E-07	5.29E-07
55120	1602	FANCL	0.48	7.15E-07	2.62E-06
5569	3969	PKIA	0.48	5.87E-06	1.96E-05
79739	7653	TTLL7	0.48	1.81E-05	5.70E-05
7700	4385	ZNF141	0.48	0.00453306	0.00983316
26030	4698	PLEKHG3	0.48	4.01E-10	1.96E-09
84292	1272	WDR83	0.48	0.003649909	0.008044106
171586	2000	ABHD3	0.48	4.06E-07	1.53E-06
56940	2179	DUSP22	0.48	1.85E-05	5.81E-05

6901	1831	TAZ	0.48	0.000258215	0.000690749
541578	2211	CXorf40B	0.48	6.68E-05	0.000194368
284371	4330	ZNF841	0.48	0.002274595	0.005194731
80336	2135	PABPC1L	0.48	2.83E-05	8.68E-05
1.08E+08	2515	LOC107983997	0.48	0.022473168	0.042299073
374986	6249	MIGA1	0.48	2.40E-06	8.33E-06
351	3593	APP	0.47	1.09E-14	7.41E-14
91452	4052	ACBD5	0.47	9.11E-06	2.97E-05
1629	5654	DBT	0.47	5.44E-05	0.0001605
7105	2230	TSPAN6	0.47	1.49E-07	5.87E-07
5638	4330	PRRG1	0.47	5.56E-05	0.000163585
8790	4722	FPGT	0.47	0.001539197	0.003625674
6778	3991	STAT6	0.47	9.87E-13	5.84E-12
117583	7790	PARD3B	0.47	0.000190391	0.000519401
280636	1447	SELENOH	0.47	2.61E-06	9.04E-06
255783	849	INAFM1	0.47	0.000304526	0.000805902
84081	2335	NSRP1	0.47	2.45E-09	1.13E-08
7082	6865	TJP1	0.47	4.18E-10	2.04E-09
84527	2961	ZNF559	0.47	0.001444132	0.00342307
6197	7057	RPS6KA3	0.47	2.82E-10	1.40E-09
1.01E+08	9687	LOC100996724	0.47	0.001867113	0.004331488
5478	2289	PPIA	0.47	3.17E-10	1.57E-09
84437	3093	MSANTD4	0.47	8.29E-06	2.72E-05
4665	2689	NAB2	0.47	3.82E-06	1.30E-05
283219	3950	KCTD21	0.47	0.002004051	0.004624097
10382	2501	TUBB4A	0.47	0.000134399	0.000374588
80298	2076	MTERF2	0.47	0.000608129	0.001536144
51280	3097	GOLM1	0.47	7.60E-13	4.52E-12
3622	1228	ING2	0.47	0.00158817	0.003731632
10398	1180	MYL9	0.47	5.00E-09	2.25E-08
118812	2411	MORN4	0.47	0.009565519	0.019468515
58517	4312	RBM25	0.47	2.07E-10	1.04E-09
1611	2385	DAP	0.47	6.98E-11	3.66E-10
5090	2721	PBX3	0.47	1.06E-07	4.22E-07
54885	5422	TBC1D8B	0.47	0.001917382	0.004437676
94101	2166	ORMDL1	0.47	3.55E-07	1.35E-06
291	4420	SLC25A4	0.47	2.11E-05	6.58E-05
6902	688	TBCA	0.47	3.54E-08	1.48E-07
65003	1067	MRPL11	0.47	1.28E-07	5.06E-07
91947	4074	ARRDC4	0.47	2.86E-05	8.75E-05
7220	4157	TRPC1	0.47	8.67E-05	0.000248389
4677	2789	NARS	0.47	5.48E-14	3.58E-13
10935	1638	PRDX3	0.47	8.18E-10	3.92E-09
5906	1538	RAP1A	0.47	3.88E-08	1.62E-07
56172	6438	ANKH	0.47	0.00010336	0.00029337
64860	2755	ARMCX5	0.46	0.001605403	0.003768636
11180	4150	WDR6	0.46	1.01E-11	5.57E-11
125170	2609	MIEF2	0.46	0.000587886	0.001490306

57732	3118	ZFYVE28	0.46	0.02242179	0.042215786
54629	9235	MINDY2	0.46	2.69E-06	9.30E-06
677	3211	ZFP36L1	0.46	7.36E-11	3.85E-10
4696	421	NDUFA3	0.46	1.28E-05	4.09E-05
256281	887	NUDT14	0.46	0.000166793	0.000459184
126789	1249	PUSL1	0.46	0.001457332	0.003452055
51574	2235	LARP7	0.46	1.08E-08	4.76E-08
16	3344	AARS	0.46	2.43E-12	1.40E-11
51351	6404	ZNF117	0.46	0.002382872	0.005424582
92579	1534	G6PC3	0.46	3.59E-09	1.64E-08
51019	974	WASHC3	0.46	1.86E-05	5.84E-05
3094	690	HINT1	0.46	1.80E-09	8.39E-09
345778	7803	MTX3	0.46	0.000107439	0.000303929
149473	1307	CCDC24	0.46	0.006396044	0.01347941
29956	2491	CERS2	0.46	1.99E-14	1.33E-13
51123	2779	ZNF706	0.46	3.34E-06	1.14E-05
57798	4597	GATAD1	0.46	1.79E-07	6.99E-07
90441	1710	ZNF622	0.46	0.00017362	0.000476431
9961	2912	MVP	0.46	1.51E-10	7.65E-10
54566	4041	EPB41L4B	0.46	0.007696092	0.015952423
8028	4239	MLLT10	0.46	4.49E-06	1.52E-05
10957	2133	PNRC1	0.46	1.06E-08	4.64E-08
653464	6081	SRGAP2C	0.46	5.97E-10	2.89E-09
51596	982	CUTA	0.46	4.15E-05	0.000124275
51027	911	BOLA1	0.46	0.017350961	0.0335428
5893	2981	RAD52	0.46	0.003765093	0.008277429
26010	2364	SPATS2L	0.46	8.32E-09	3.68E-08
94134	4964	ARHGAP12	0.46	4.39E-06	1.49E-05
55186	4820	SLC25A36	0.46	4.46E-08	1.85E-07
4194	9880	MDM4	0.46	2.84E-05	8.69E-05
3964	5471	LGALS8	0.46	1.23E-07	4.88E-07
23215	10453	PRRC2C	0.46	1.21E-15	8.72E-15
51075	1710	TMX2	0.46	1.36E-09	6.40E-09
754	2841	PTTG1IP	0.46	7.70E-14	4.94E-13
8091	3741	HMGA2	0.46	1.07E-06	3.86E-06
1039	2702	CDR2	0.46	2.82E-08	1.19E-07
8898	4646	MTMR2	0.46	1.78E-09	8.33E-09
1072	1260	CFL1	0.46	1.15E-07	4.59E-07
784	2701	CACNB3	0.46	3.48E-09	1.59E-08
3508	3136	IGHMBP2	0.46	4.82E-05	0.000143257
4071	1711	TM4SF1	0.46	3.39E-08	1.42E-07
23033	9268	DOPEY1	0.46	0.001253186	0.003000891
51035	1162	UBXN1	0.46	1.07E-08	4.70E-08
5721	868	PSME2	0.46	1.64E-09	7.67E-09
23524	9030	SRRM2	0.46	0.019086871	0.036544775
23503	9470	ZFYVE26	0.46	1.31E-06	4.67E-06
9145	1720	SYNGR1	0.46	0.000139158	0.000387424
81792	7683	ADAMTS12	0.45	5.42E-05	0.000159822

10169	3041	SERF2	0.45	2.67E-06	9.24E-06
7430	3001	EZR	0.45	3.59E-15	2.51E-14
7746	1815	ZSCAN9	0.45	0.011808015	0.023541833
54520	6639	CCDC93	0.45	3.00E-07	1.14E-06
79161	1570	TMEM243	0.45	1.85E-05	5.81E-05
90576	2604	ZNF799	0.45	0.01231719	0.024493769
79101	1323	TAF1D	0.45	1.27E-05	4.08E-05
54737	4284	MPHOSPH8	0.45	2.93E-08	1.24E-07
80331	5344	DNAJC5	0.45	7.05E-09	3.14E-08
5954	2529	RCN1	0.45	3.13E-14	2.07E-13
3704	1205	ITPA	0.45	4.92E-05	0.000146061
26115	11858	TANC2	0.45	4.72E-07	1.77E-06
376940	10703	ZC3H6	0.45	0.001503273	0.00355141
84283	2215	TMEM79	0.45	0.021296079	0.040292698
59286	472	UBL5	0.45	2.54E-06	8.79E-06
353	764	APRT	0.45	5.74E-05	0.000168359
283659	10563	PRTG	0.45	0.000140593	0.000391054
51582	4249	AZIN1	0.45	4.35E-10	2.12E-09
967	885	CD63	0.45	4.13E-12	2.34E-11
79572	7181	ATP13A3	0.45	1.93E-12	1.12E-11
1.01E+08	7604	LOC100996763	0.45	6.77E-06	2.25E-05
80205	10609	CHD9	0.45	6.98E-09	3.11E-08
2653	1532	GCSH	0.45	3.60E-06	1.23E-05
51506	978	UFC1	0.45	2.89E-08	1.22E-07
64780	3603	MICAL1	0.45	4.24E-07	1.60E-06
3659	2187	IRF1	0.45	2.88E-08	1.22E-07
23078	5575	VWA8	0.45	6.90E-06	2.29E-05
2585	2893	GALK2	0.45	0.005435745	0.011609846
79918	2960	SETD6	0.45	0.009734079	0.019779862
5890	2241	RAD51B	0.45	0.011520734	0.023028508
9521	1097	EEF1E1	0.45	0.000147335	0.000409102
9648	6818	GCC2	0.45	8.98E-10	4.28E-09
81545	4064	FBXO38	0.45	9.35E-07	3.39E-06
6342	1593	SCP2	0.45	1.42E-08	6.17E-08
8225	1792	GTPBP6	0.45	2.25E-05	6.98E-05
51000	2031	SLC35B3	0.45	0.000122863	0.000344002
167227	9306	DCP2	0.45	5.96E-06	1.99E-05
7322	2594	UBE2D2	0.45	1.09E-10	5.63E-10
5204	737	PFDN5	0.45	3.24E-06	1.11E-05
117854	3254	TRIM6	0.45	0.004957093	0.010667025
113263	4811	GLCCI1	0.45	0.002457229	0.005578845
220213	3125	OTUD1	0.45	0.002396565	0.005451565
10094	961	ARPC3	0.44	1.52E-08	6.56E-08
64921	3594	CASD1	0.44	4.93E-05	0.000146256
129531	988	MITD1	0.44	3.05E-05	9.29E-05
92335	2280	STRADA	0.44	6.01E-06	2.01E-05
6281	1069	S100A10	0.44	2.33E-08	9.92E-08
10749	6016	KIF1C	0.44	6.00E-11	3.16E-10

79037	1586	PVRIG	0.44	0.000605978	0.0015318
5526	2445	PPP2R5B	0.44	6.59E-05	0.00019208
92181	3015	UBTD2	0.44	2.79E-08	1.18E-07
9270	1411	ITGB1BP1	0.44	8.72E-07	3.17E-06
10327	1449	AKR1A1	0.44	1.05E-06	3.78E-06
51699	1152	VPS29	0.44	1.34E-08	5.84E-08
10272	2525	FSTL3	0.44	2.83E-09	1.30E-08
134553	3934	C5orf24	0.44	2.92E-11	1.57E-10
51504	1308	TRMT112	0.44	1.25E-07	4.97E-07
5917	2154	RARS	0.44	4.04E-09	1.83E-08
55291	5049	PPP6R3	0.44	2.68E-12	1.54E-11
53918	2941	PELO	0.44	0.000181642	0.000497215
6490	2142	PMEL	0.44	0.012545755	0.024912045
84932	2850	RAB2B	0.44	3.76E-05	0.000113095
79818	2352	ZNF552	0.44	0.01651727	0.032084712
1E+08	509	OST4	0.44	1.12E-05	3.61E-05
339231	1207	ARL16	0.44	7.43E-05	0.000215061
92935	3102	MARS2	0.44	0.016563675	0.032171335
84940	2311	CORO6	0.44	0.000430047	0.001113859
9577	1825	BABAM2	0.44	1.18E-06	4.25E-06
156	3302	GRK2	0.44	3.08E-08	1.29E-07
1021	11649	CDK6	0.44	2.78E-06	9.58E-06
55763	3444	EXOC1	0.44	1.39E-06	4.94E-06
54059	1524	YBEY	0.44	0.000245477	0.000658558
10200	1105	MPHOSPH6	0.44	0.000178269	0.000488433
55611	2310	OTUB1	0.44	3.70E-06	1.26E-05
56834	1889	GPR137	0.44	6.44E-05	0.000187863
51300	1450	TIMMDC1	0.44	4.00E-06	1.36E-05
6048	1176	RNF5	0.44	5.86E-07	2.18E-06
9274	1131	BCL7C	0.44	1.34E-06	4.78E-06
25847	1342	ANAPC13	0.44	4.39E-07	1.65E-06
4637	795	MYL6	0.44	1.77E-07	6.92E-07
83734	2147	ATG10	0.44	0.00571474	0.012153142
7324	1732	UBE2E1	0.44	8.17E-08	3.30E-07
126626	2606	GABPB2	0.44	0.010091532	0.020435741
170463	1893	SSBP4	0.44	2.82E-07	1.08E-06
7045	2805	TGFBI	0.44	3.02E-09	1.39E-08
29801	4963	ZDHHC8	0.44	4.83E-06	1.63E-05
201255	2741	LRRC45	0.44	1.63E-05	5.16E-05
64743	1985	WDR13	0.44	1.54E-07	6.05E-07
79567	4115	RIPOR1	0.44	1.45E-05	4.60E-05
65990	872	FAM173A	0.44	0.003338822	0.007396108
27309	1911	ZNF330	0.44	1.74E-05	5.49E-05
389203	1021	SMIM20	0.44	0.001491522	0.003525993
25921	4454	ZDHHC5	0.44	1.90E-11	1.03E-10
65975	2353	STK33	0.44	0.00075441	0.001878413
54103	3058	GSAP	0.44	0.001064242	0.002583259
84910	5091	TMEM87B	0.43	5.05E-05	0.000149649

256643	6692	BCLAF3	0.43	0.000794897	0.001970112
729540	6979	RGPD6	0.43	0.000232428	0.000625345
23603	3596	CORO1C	0.43	6.23E-09	2.78E-08
54477	3948	PLEKHA5	0.43	1.11E-05	3.57E-05
51524	1312	TMEM138	0.43	0.000191465	0.000522011
4817	1724	NIT1	0.43	6.42E-05	0.000187303
10908	4451	PNPLA6	0.43	1.08E-07	4.30E-07
114781	8675	BTBD9	0.43	0.002713925	0.006112437
23623	2350	RUSC1	0.43	6.17E-08	2.52E-07
6482	6812	ST3GAL1	0.43	1.08E-07	4.30E-07
5437	1344	POLR2H	0.43	5.85E-06	1.96E-05
3728	3351	JUP	0.43	1.28E-06	4.56E-06
84964	990	ALKBH6	0.43	0.001455345	0.00344781
64928	980	MRPL14	0.43	5.35E-05	0.000158049
60592	1942	SCOC	0.43	1.29E-06	4.60E-06
64210	3627	MMS19	0.43	2.05E-08	8.78E-08
29761	4511	USP25	0.43	5.84E-08	2.40E-07
134266	4105	GRPEL2	0.43	4.08E-06	1.38E-05
283899	1334	INO80E	0.43	1.07E-05	3.47E-05
8798	974	DYRK4	0.43	0.005301456	0.011358527
89781	3331	HPS4	0.43	1.83E-05	5.77E-05
54558	4323	SPATA6	0.43	0.009737388	0.019784323
55151	2857	TMEM38B	0.43	0.000209894	0.000568246
151525	1694	WDSUB1	0.43	0.003740326	0.008230106
8614	5361	STC2	0.43	7.32E-07	2.68E-06
3486	2617	IGFBP3	0.43	5.77E-11	3.04E-10
10629	1978	TAF6L	0.43	0.000151517	0.000420057
84274	1538	COQ5	0.43	0.000304859	0.000806541
27075	1912	TSPAN13	0.43	7.75E-05	0.000223609
51024	785	FIS1	0.43	3.91E-05	0.000117505
219988	3824	PATL1	0.43	3.65E-10	1.79E-09
22832	4285	CEP162	0.43	0.000285478	0.000759111
1.03E+08	4047	LOC102724200	0.43	0.00037017	0.000967944
54938	2077	SARS2	0.43	1.30E-05	4.16E-05
11217	6857	AKAP2	0.43	7.60E-11	3.97E-10
64359	2825	NXN	0.43	8.98E-09	3.97E-08
26031	6547	OSBPL3	0.43	1.65E-08	7.10E-08
50865	1221	HEBP1	0.43	2.27E-05	7.04E-05
79786	7535	KLHL36	0.43	3.43E-06	1.17E-05
8463	2027	TEAD2	0.43	7.68E-07	2.81E-06
58533	2978	SNX6	0.43	2.71E-09	1.25E-08
2296	3452	FOXC1	0.43	2.91E-05	8.89E-05
79096	1740	C11orf49	0.43	6.51E-06	2.17E-05
79863	5669	RBFA	0.43	0.000238453	0.000640972
3682	4974	ITGAE	0.43	0.020055325	0.038189156
55119	3787	PRPF38B	0.43	3.34E-08	1.40E-07
1997	3646	ELF1	0.43	2.14E-07	8.29E-07
91851	3987	CHRDL1	0.43	8.61E-06	2.82E-05

7748	3181	ZNF195	0.43	5.46E-05	0.000161117
90874	5143	ZNF697	0.42	0.003386778	0.007492999
5364	7573	PLXNB1	0.42	4.60E-07	1.73E-06
1207	1369	CLNS1A	0.42	1.14E-07	4.53E-07
3475	2583	IFRD1	0.42	0.000126671	0.000354049
5499	1481	PPP1CA	0.42	6.92E-08	2.82E-07
5742	5107	PTGS1	0.42	0.002872932	0.006440356
10981	1227	RAB32	0.42	0.001097118	0.002656526
129880	3196	BBS5	0.42	0.000297311	0.000787981
55048	2564	VPS37C	0.42	0.000300968	0.000797079
83986	2981	FAM234A	0.42	1.07E-09	5.07E-09
80179	3919	MYO19	0.42	3.86E-08	1.61E-07
81553	4615	FAM49A	0.42	6.50E-05	0.000189494
10138	2562	YAF2	0.42	5.98E-05	0.00017491
117584	4137	RFFL	0.42	5.58E-05	0.000164184
10296	2232	MAEA	0.42	1.16E-06	4.16E-06
586	8469	BCAT1	0.42	8.91E-07	3.23E-06
29952	1630	DPP7	0.42	2.19E-08	9.36E-08
55615	1878	PRR5	0.42	0.011255602	0.022561996
54949	1227	SDHAF2	0.42	1.82E-05	5.74E-05
339229	1178	OXLD1	0.42	0.001174334	0.002828079
8763	3024	CD164	0.42	2.33E-12	1.34E-11
9061	2556	PAPSS1	0.42	1.24E-07	4.92E-07
339366	2580	ADAMTSL5	0.42	0.000634715	0.001598756
22852	7411	ANKRD26	0.42	6.57E-06	2.18E-05
93974	611	ATP5IF1	0.42	1.96E-07	7.63E-07
965	1273	CD58	0.42	1.98E-05	6.18E-05
84080	1628	ENKD1	0.42	0.001224458	0.002937737
84331	928	MCRIP2	0.42	0.010843876	0.021815443
27030	6698	MLH3	0.42	0.000196325	0.000534441
83636	4345	C19orf12	0.42	0.000395316	0.001030212
3313	3506	HSPA9	0.42	1.57E-11	8.56E-11
9532	2068	BAG2	0.42	0.000139629	0.000388508
79184	3238	BRCC3	0.42	2.43E-06	8.43E-06
1.01E+08	2655	RPL36A-HNRNPH2	0.42	0.002343098	0.005338826
7978	1989	MTERF1	0.42	0.007928759	0.01638309
7381	4839	UQCRB	0.42	1.00E-06	3.63E-06
152100	827	CMC1	0.42	0.010328553	0.020875637
55967	588	NDUFA12	0.42	8.75E-06	2.86E-05
1.02E+08	1918	LOC101928589	0.42	0.02427964	0.045362844
84190	2375	METTL25	0.42	0.024628994	0.045972052
2011	4534	MARK2	0.42	8.36E-07	3.04E-06
9658	8389	ZNF516	0.42	0.000695894	0.001741989
51264	728	MRPL27	0.42	0.000275935	0.000735384
134353	6591	LSM11	0.42	0.007067989	0.01475723
285672	6860	SREK1IP1	0.42	4.35E-06	1.47E-05
9289	3810	ADGRG1	0.42	6.26E-07	2.31E-06

84864	3823	RIOX2	0.41	0.000292348	0.000776101
84193	2728	SETD3	0.41	4.22E-08	1.75E-07
10325	1816	RRAGB	0.41	0.004957086	0.010667025
3672	4811	ITGA1	0.41	4.70E-05	0.000139788
10184	4869	LHFPL2	0.41	2.71E-06	9.37E-06
5468	1894	PPARG	0.41	0.024158747	0.045160718
145553	756	MDP1	0.41	0.018716317	0.035908813
29990	1438	PILRB	0.41	0.000515692	0.001317632
79971	2692	WLS	0.41	1.73E-06	6.12E-06
6793	5789	STK10	0.41	0.000207889	0.000563505
23189	5336	KANK1	0.41	5.51E-05	0.000162367
4598	2041	MVK	0.41	0.001603204	0.00376397
84447	3071	SYVN1	0.41	2.23E-05	6.94E-05
10428	1274	CFDP1	0.41	5.95E-06	1.99E-05
55017	1527	C14orf119	0.41	2.32E-06	8.06E-06
23216	2726	TBC1D1	0.41	2.62E-07	1.00E-06
201254	836	CENPX	0.41	0.000212071	0.000573614
54462	6944	CCSER2	0.41	4.84E-05	0.000143693
80755	1377	AARSD1	0.41	0.000381331	0.00099581
28982	5484	FLVCR1	0.41	0.005103767	0.010954788
22890	3861	ZBTB1	0.41	6.47E-07	2.38E-06
25832	10084	NBPF14	0.41	1.37E-08	5.93E-08
4771	5419	NF2	0.41	2.88E-09	1.32E-08
2260	5047	FGFR1	0.41	4.70E-07	1.76E-06
112611	4051	RWDD2A	0.41	0.006140102	0.012981597
9958	4535	USP15	0.41	1.20E-05	3.85E-05
55102	11099	ATG2B	0.41	3.85E-05	0.000115748
4750	5361	NEK1	0.41	3.58E-05	0.000108136
55009	912	C19orf24	0.41	0.002708561	0.00610345
58499	7867	ZNF462	0.41	5.14E-05	0.000152135
57515	3218	SERINC1	0.41	1.38E-11	7.58E-11
5834	4131	PYGB	0.41	2.04E-07	7.94E-07
1983	5947	EIF5	0.41	5.97E-10	2.89E-09
256471	4779	MFSD8	0.41	0.000154563	0.000427237
51501	1141	HIKESHI	0.41	0.00046785	0.001204398
7011	9893	TEP1	0.41	0.000169974	0.000467173
23593	1277	HEBP2	0.41	9.87E-06	3.21E-05
10569	2770	SLU7	0.41	4.64E-08	1.92E-07
57561	4178	ARRDC3	0.41	1.31E-07	5.18E-07
7542	1400	ZFPL1	0.41	0.000224059	0.000604383
285550	4402	FAM200B	0.40	0.002715401	0.006114986
4708	509	NDUFB2	0.40	0.002233949	0.005107155
91942	719	NDUFAF2	0.40	0.001292934	0.003087334
23173	2623	METAP1	0.40	2.28E-07	8.81E-07
60496	2880	AASDHPPT	0.40	2.55E-06	8.83E-06
84987	754	COX14	0.40	0.003009819	0.006724313
84246	1059	MED10	0.40	0.000390335	0.001017977
10209	1326	EIF1	0.40	8.23E-09	3.64E-08

25950	1289	RWDD3	0.40	0.01907594	0.036527784
7325	1660	UBE2E2	0.40	0.00038869	0.001013835
29994	7764	BAZ2B	0.40	6.01E-06	2.01E-05
84221	1263	SPATC1L	0.40	0.001515491	0.003577421
81631	2304	MAP1LC3B	0.40	1.37E-07	5.40E-07
2171	751	FABP5	0.40	0.000209813	0.000568112
89941	2538	RHOT2	0.40	3.03E-08	1.28E-07
23596	2632	OPN3	0.40	0.000881564	0.002168577
23412	942	COMMD3	0.40	0.00109549	0.002652946
10519	999	CIB1	0.40	8.02E-05	0.000230893
57326	3285	PBXIP1	0.40	5.13E-07	1.91E-06
65250	8732	C5orf42	0.40	0.000146942	0.000408074
10069	1715	RWDD2B	0.40	0.000827539	0.002043886
26056	4913	RAB11FIP5	0.40	4.65E-06	1.57E-05
1122	7082	CHML	0.40	0.000279204	0.000743427
9767	4741	JADE3	0.40	2.67E-05	8.22E-05
1.01E+08	1999	LOC100996709	0.40	0.000606211	0.00153199
126382	1291	NR2C2AP	0.40	0.001841144	0.004275707
144100	4795	PLEKHA7	0.40	0.002482087	0.005632404
116238	1016	TLCD1	0.40	0.00483165	0.010426131
2237	2308	FEN1	0.40	1.53E-08	6.63E-08
8871	6501	SYNJ2	0.40	1.89E-07	7.35E-07
79075	2248	DSCC1	0.40	0.001574369	0.003702144
4361	4989	MRE11	0.40	2.78E-05	8.53E-05
394	8415	ARHGAP5	0.40	2.96E-07	1.13E-06
283431	5519	GAS2L3	0.40	6.95E-06	2.30E-05
79760	1045	GEMIN7	0.40	0.008109564	0.016719742
128387	2453	TATDN3	0.40	0.005750117	0.012223986
149041	10942	RC3H1	0.40	1.37E-05	4.37E-05
493	8761	ATP2B4	0.40	1.40E-07	5.51E-07
84263	3393	HSDL2	0.40	8.31E-08	3.35E-07
4710	622	NDUFB4	0.40	5.12E-07	1.91E-06
847	2300	CAT	0.40	1.21E-07	4.81E-07
26091	4283	HERC4	0.40	1.94E-06	6.79E-06
52	1558	ACP1	0.40	4.36E-07	1.64E-06
139322	6337	APOOL	0.40	0.000885173	0.002176248
9147	4486	NEMF	0.40	5.20E-07	1.94E-06
6652	2813	SORD	0.40	5.56E-05	0.000163495
5654	2138	HTRA1	0.40	1.22E-06	4.36E-06
6940	2629	ZNF354A	0.40	0.001299238	0.00310072
79921	1326	TCEAL4	0.40	7.10E-08	2.89E-07
8896	1078	BUD31	0.40	8.35E-06	2.74E-05
84669	6356	USP32	0.40	2.07E-07	8.03E-07
55906	2827	ZC4H2	0.40	0.002106429	0.004839911
2491	5477	CENPI	0.40	6.26E-05	0.000182952
64901	3376	RANBP17	0.39	0.008068863	0.01664162
83861	5338	RSPH3	0.39	0.001382071	0.00328429
23036	10268	ZNF292	0.39	2.68E-07	1.03E-06

84869	3454	CBR4	0.39	0.004728475	0.010219626
11044	3832	PAPD7	0.39	5.52E-05	0.000162529
54532	6076	USP53	0.39	0.000245176	0.00065785
928	1321	CD9	0.39	1.81E-10	9.12E-10
4709	775	NDUFB3	0.39	7.75E-05	0.000223609
221545	1583	C6orf136	0.39	0.001511787	0.003570101
526	3054	ATP6V1B2	0.39	7.41E-06	2.45E-05
27072	3936	VPS41	0.39	2.56E-05	7.88E-05
10144	5574	FAM13A	0.39	0.000714931	0.001787629
84307	9709	ZNF397	0.39	0.00609309	0.012891406
54875	4424	CNTLN	0.39	1.26E-05	4.03E-05
80762	3599	NDFIP1	0.39	1.61E-09	7.53E-09
51274	5502	KLF3	0.39	0.000116979	0.000329081
64215	2206	DNAJC1	0.39	0.000255474	0.000683932
55179	1079	FAIM	0.39	0.000341575	0.000898062
10311	3223	DSCR3	0.39	0.000122883	0.000344003
5795	7383	PTPRJ	0.39	4.15E-09	1.88E-08
196441	7158	ZFC3H1	0.39	0.000223789	0.000603748
126374	2173	WTIP	0.39	0.002479638	0.005627566
476	3736	ATP1A1	0.39	3.71E-12	2.11E-11
348013	1549	TMEM255B	0.39	0.018082474	0.034805481
29796	942	UQCR10	0.39	1.56E-05	4.93E-05
4548	9846	MTR	0.39	5.54E-06	1.86E-05
1.02E+08	666	C11orf98	0.39	3.46E-05	0.000104797
9146	2980	HGS	0.39	1.05E-07	4.21E-07
80115	2070	BAIAP2L2	0.39	0.003711898	0.008169579
5076	3841	PAX2	0.39	0.016755993	0.032517127
9842	4958	PLEKHM1	0.39	1.20E-05	3.87E-05
4179	3296	CD46	0.39	2.02E-06	7.09E-06
4343	3852	MOV10	0.39	2.23E-07	8.63E-07
79640	5004	C22orf46	0.39	0.000202445	0.000549755
55695	2035	NSUN5	0.39	5.85E-05	0.000171351
1809	5203	DPYSL3	0.39	1.13E-07	4.49E-07
5147	1218	PDE6D	0.39	0.000624601	0.00157425
11116	3701	FGFR1OP	0.39	0.000229255	0.000617369
121551	4299	BTBD11	0.39	0.00849144	0.017449326
636	6501	BICD1	0.39	8.19E-05	0.000235394
51324	1743	SPG21	0.39	2.51E-06	8.69E-06
25855	1452	BRMS1	0.39	3.34E-05	0.000101293
513	821	ATP5F1D	0.39	6.15E-06	2.05E-05
10424	3790	PGRMC2	0.39	3.41E-08	1.43E-07
1184	9728	CLCN5	0.39	0.016254774	0.03161283
79893	2672	GGNBP2	0.39	3.98E-09	1.81E-08
285521	3209	COX18	0.39	0.003080207	0.006871201
221078	2859	NSUN6	0.39	0.010537221	0.021255227
818	3641	CAMK2G	0.39	3.10E-05	9.43E-05
10943	2713	MSL3	0.39	0.000624015	0.001573141
664	1661	BNIP3	0.39	1.38E-06	4.92E-06

9603	3720	NFE2L3	0.39	2.94E-05	8.97E-05
84279	1105	PRADC1	0.38	0.004371253	0.009499562
9465	2384	AKAP7	0.38	0.017685175	0.034133195
51655	1814	RASD1	0.38	0.009693474	0.019701859
93134	4570	ZNF561	0.38	0.001025309	0.002494206
1153	1438	CIRBP	0.38	4.89E-07	1.83E-06
54901	3264	CDKAL1	0.38	0.000445111	0.0011505
55930	6937	MYO5C	0.38	1.48E-05	4.69E-05
3217	1377	HOXB7	0.38	1.73E-05	5.44E-05
2595	3110	GANC	0.38	0.002289605	0.005224981
9194	11725	SLC16A7	0.38	0.000144794	0.000402237
3554	4911	IL1R1	0.38	1.43E-10	7.30E-10
145389	1603	SLC38A6	0.38	0.010084573	0.020424335
2920	1234	CXCL2	0.38	0.009182641	0.018749386
1.02E+08	9516	LOC101928841	0.38	0.023133777	0.043436643
11152	1683	WDR45	0.38	0.000309704	0.000818384
8859	1621	STK19	0.38	0.018877641	0.036167557
200765	2636	TIGD1	0.38	0.007435327	0.015458798
1609	4653	DGKQ	0.38	0.000955271	0.00233726
51306	5352	FAM13B	0.38	8.98E-07	3.26E-06
9704	4530	DHX34	0.38	0.004685987	0.010137662
90799	3070	CEP95	0.38	0.000197584	0.000537539
79899	3886	PRR5L	0.38	0.001839356	0.004272673
153129	2455	SLC38A9	0.38	0.001171485	0.002822749
5372	1267	PMM1	0.38	0.001025607	0.00249459
10653	1814	SPINT2	0.38	1.72E-05	5.42E-05
55793	2474	MINDY1	0.38	0.003678318	0.008102697
5922	5512	RASA2	0.38	0.000173558	0.000476333
80212	2011	CCDC92	0.38	0.000180033	0.000492961
4017	3810	LOXL2	0.38	9.47E-08	3.80E-07
80381	3041	CD276	0.38	3.95E-07	1.49E-06
1759	3857	DNM1	0.38	3.96E-05	0.000118968
9019	4914	MPZL1	0.38	1.49E-08	6.45E-08
91689	1585	SMDT1	0.38	0.0019639	0.004535887
51091	5658	SEPSECS	0.38	0.009750652	0.019806744
23016	1097	EXOSC7	0.38	0.001254335	0.003003237
87	3752	ACTN1	0.38	3.54E-08	1.48E-07
55833	3691	UBAP2	0.38	8.73E-05	0.000250232
92999	5524	ZBTB47	0.38	0.000415278	0.001078436
1465	1970	CSRP1	0.38	1.64E-06	5.78E-06
1340	590	COX6B1	0.38	1.17E-06	4.19E-06
1956	5616	EGFR	0.37	0.000290837	0.000772435
166929	6080	SGMS2	0.37	3.78E-05	0.000113824
389541	641	LAMTOR4	0.37	0.001531331	0.003610016
148156	3397	ZNF558	0.37	0.001760148	0.004099397
10399	1125	RACK1	0.37	4.08E-08	1.70E-07
55719	5685	SLF2	0.37	5.93E-06	1.98E-05
29099	2643	COMMD9	0.37	9.15E-06	2.98E-05

9702	3147	CEP57	0.37	1.09E-06	3.93E-06
9553	493	MRPL33	0.37	0.000986131	0.002406243
2534	2880	FYN	0.37	5.95E-05	0.000174319
7072	5877	TIA1	0.37	3.01E-05	9.19E-05
50854	760	C6orf48	0.37	4.68E-05	0.000139407
55650	2461	PIGV	0.37	0.00754274	0.015658303
9685	3696	CLINT1	0.37	1.18E-07	4.68E-07
27109	1668	ATP5S	0.37	0.002781126	0.006247162
79590	907	MRPL24	0.37	5.60E-05	0.000164636
11179	2299	ZNF277	0.37	0.004280509	0.009316031
4267	1317	CD99	0.37	4.14E-05	0.000124182
8764	1964	TNFRSF14	0.37	0.010710003	0.02156321
8045	1710	RASSF7	0.37	3.44E-06	1.18E-05
4008	4953	LMO7	0.37	2.11E-05	6.58E-05
83989	5146	FAM172A	0.37	2.48E-05	7.65E-05
64848	6119	YTHDC2	0.37	2.57E-06	8.92E-06
6813	1904	STXBP2	0.37	1.91E-05	5.99E-05
2175	4705	FANCA	0.37	6.08E-05	0.000177896
23141	4389	ANKLE2	0.37	5.94E-07	2.20E-06
130557	1977	ZNF513	0.37	0.002388321	0.005434659
84936	2288	ZFYVE19	0.37	0.000483001	0.001239451
1509	2141	CTSD	0.37	7.18E-09	3.20E-08
84520	1191	GON7	0.37	0.000651541	0.001637423
84280	2422	BTBD10	0.37	0.000142836	0.000397232
1192	1251	CLIC1	0.37	2.35E-07	9.08E-07
79624	2619	ARMT1	0.37	0.000128423	0.000358552
10897	1168	YIF1A	0.37	0.000119524	0.000335446
9497	6771	SLC4A7	0.37	5.47E-05	0.00016132
1326	4183	MAP3K8	0.37	0.02370751	0.044415334
3621	2174	ING1	0.37	0.001337566	0.003186627
2580	4376	GAK	0.37	6.19E-06	2.07E-05
55329	2023	MNS1	0.37	0.000443937	0.001148162
527	1180	ATP6V0C	0.37	0.000118766	0.000333529
92922	2450	CCDC102A	0.37	0.017828833	0.034380584
26052	5008	DNM3	0.37	0.010569656	0.021312034
7769	2063	ZNF226	0.37	0.002812621	0.006313918
4717	746	NDUFC1	0.37	0.000138747	0.000386344
81844	10542	TRIM56	0.37	1.84E-06	6.46E-06
9392	5375	TGFBRAP1	0.37	3.01E-06	1.03E-05
51259	1311	TMEM216	0.37	0.021109018	0.039964308
200014	4036	CC2D1B	0.37	6.30E-06	2.10E-05
1781	3360	DYNC1I2	0.37	2.11E-06	7.37E-06
8270	964	LAGE3	0.37	0.005190877	0.011135036
8216	4332	LZTR1	0.37	6.86E-05	0.000199089
81890	1352	QTRT1	0.37	0.002604918	0.005886327
10627	1158	MYL12A	0.37	1.81E-07	7.07E-07
6993	787	DYNLT1	0.37	7.73E-05	0.00022305
92979	2985	MARCH9	0.37	0.002819573	0.006328724

9179	1811	AP4M1	0.37	0.003142455	0.006999517
221749	2209	PXDC1	0.37	9.39E-05	0.000267867
9554	2622	SEC22B	0.37	2.75E-08	1.17E-07
1155	1045	TBCB	0.37	0.000102522	0.000291179
55015	2894	PRPF39	0.37	3.13E-05	9.53E-05
27086	6017	FOXP1	0.37	1.07E-06	3.84E-06
5868	2548	RAB5A	0.37	2.88E-05	8.80E-05
10915	3969	TCERG1	0.36	9.09E-06	2.96E-05
84875	3525	PARP10	0.36	0.000318194	0.000839945
64599	6069	GIGYF1	0.36	0.000105285	0.00029829
84250	4523	SLF1	0.36	0.001211602	0.002909941
7360	2140	UGP2	0.36	7.90E-06	2.60E-05
11216	3816	AKAP10	0.36	9.32E-05	0.000266016
22862	5991	FNDC3A	0.36	3.62E-06	1.23E-05
2720	2607	GLB1	0.36	8.83E-07	3.21E-06
55824	10471	PAG1	0.36	0.003212805	0.007143676
54939	944	COMMD4	0.36	0.000303484	0.000803504
192670	6607	ago-04	0.36	0.001064034	0.002583106
26580	1596	BSCL2	0.36	7.84E-05	0.000226318
10289	1014	EIF1B	0.36	0.000183528	0.00050199
10494	2314	STK25	0.36	4.46E-07	1.67E-06
5080	4434	PAX6	0.36	0.000868171	0.00213725
10380	2422	BPNT1	0.36	0.000797353	0.001974822
5826	2260	ABCD4	0.36	0.000158709	0.000438015
900	2429	CCNG1	0.36	1.18E-06	4.23E-06
55711	3874	FAR2	0.36	0.005841378	0.012403156
29901	1584	SAC3D1	0.36	0.00098617	0.002406243
51326	3999	ARL17A	0.36	0.000475649	0.001222351
26751	1817	SH3YL1	0.36	0.00473508	0.010232657
1327	835	COX4I1	0.36	9.48E-05	0.000270159
10540	2108	DCTN2	0.36	3.10E-06	1.07E-05
57099	1379	AVEN	0.36	0.025311312	0.04715154
254531	1901	LPCAT4	0.36	0.001688213	0.003947385
23126	6365	POGZ	0.36	6.07E-06	2.02E-05
4282	561	MIF	0.36	0.000199428	0.000542059
92106	2777	OXNAD1	0.36	0.007252874	0.015104773
7358	3113	UGDH	0.36	0.000814093	0.002012635
203054	2006	ADCK5	0.36	0.020941107	0.039680238
51115	2772	RMDN1	0.36	0.000112882	0.000318464
29063	2880	ZCCHC4	0.36	0.005087553	0.010922625
585	2519	BBS4	0.36	0.007994167	0.016502888
29966	4124	STRN3	0.36	0.00010516	0.000298051
8725	3141	URI1	0.36	6.30E-05	0.000183876
54884	3328	RETSAT	0.36	0.000676879	0.001697979
9590	8201	AKAP12	0.36	3.42E-07	1.30E-06
131076	726	CCDC58	0.36	0.00541099	0.011561143
255967	5011	PAN3	0.36	0.000557348	0.00141734
80011	3118	FAM192A	0.36	9.35E-07	3.39E-06

374395	1054	TMEM179B	0.36	0.000209058	0.000566329
29894	3614	CPSF1	0.36	5.70E-07	2.12E-06
1877	2665	E4F1	0.36	0.001078639	0.002615346
8897	7614	MTMR3	0.35	1.49E-05	4.72E-05
5052	1044	PRDX1	0.35	2.83E-05	8.68E-05
196	6247	AHR	0.35	5.42E-05	0.000159775
440400	830	RNASEK	0.35	0.000263903	0.000704798
64772	4012	ENGASE	0.35	0.003613488	0.007969768
153527	1644	ZMAT2	0.35	2.70E-06	9.31E-06
10367	2338	MICU1	0.35	8.98E-06	2.93E-05
54960	3071	GEMIN8	0.35	0.009996764	0.020258041
1337	593	COX6A1	0.35	1.13E-05	3.64E-05
94081	4084	SFXN1	0.35	1.07E-05	3.47E-05
29781	2726	NCAPH2	0.35	6.73E-05	0.000195733
9454	1553	HOMER3	0.35	0.000932071	0.002284672
5600	2404	MAPK11	0.35	0.007049958	0.014721313
323	8301	APBB2	0.35	3.18E-06	1.09E-05
205	6802	AK4	0.35	1.32E-06	4.69E-06
1906	1923	EDN1	0.35	0.001076201	0.002610502
51088	7076	KLHL5	0.35	3.14E-07	1.20E-06
51427	5751	ZNF107	0.35	0.007096443	0.014807938
92689	3924	FAM114A1	0.35	6.61E-05	0.000192369
9121	3698	SLC16A5	0.35	0.000246522	0.000661263
8558	1857	CDK10	0.35	4.60E-05	0.000136978
7267	7337	TTC3	0.35	4.40E-09	1.99E-08
8933	1254	RTL8C	0.35	0.000259446	0.000693626
23583	1432	SMUG1	0.35	0.004082584	0.008912564
5007	5083	OSBP	0.35	9.19E-07	3.33E-06
3097	9715	HIVEP2	0.35	1.24E-05	3.98E-05
121274	4999	ZNF641	0.35	0.009893049	0.02006584
56952	2002	PRTFDC1	0.35	0.014479013	0.028436288
6015	1744	RING1	0.35	3.21E-05	9.75E-05
6272	6868	SORT1	0.35	1.29E-08	5.62E-08
7368	4104	UGT8	0.35	4.35E-05	0.000130068
84324	941	SARNP	0.35	0.000139718	0.000388682
26005	6255	C2CD3	0.35	8.87E-05	0.000253831
9400	3694	RECQL5	0.35	0.001636226	0.003833899
29074	995	MRPL18	0.35	0.000605434	0.001530641
9296	748	ATP6V1F	0.35	0.000643491	0.001619025
9847	3860	C2CD5	0.35	5.44E-05	0.000160558
55593	2703	OTUD5	0.35	1.03E-05	3.32E-05
286144	3644	TRIQK	0.35	0.001534247	0.003614972
23113	6693	CUL9	0.35	0.000822644	0.002033208
51571	2043	FAM49B	0.35	0.000102096	0.000290015
284325	2589	C19orf54	0.35	0.002556059	0.005786402
152815	3700	THAP6	0.35	0.008175599	0.016844161
10483	3271	SEC23B	0.35	2.49E-05	7.69E-05
28985	9796	MCTS1	0.35	0.000750312	0.00186952

22921	1774	MSRB2	0.35	0.013892619	0.027372373
27	11522	ABL2	0.35	2.82E-05	8.66E-05
26233	1797	FBXL6	0.34	0.006228204	0.013156904
51128	6558	SAR1B	0.34	7.85E-06	2.58E-05
23241	5070	PACS2	0.34	2.09E-05	6.52E-05
2766	1517	GMPR	0.34	0.013843627	0.027284921
53834	3129	FGFRL1	0.34	0.000150518	0.000417483
535	4139	ATP6V0A1	0.34	0.000269023	0.000718042
22920	3011	KIFAP3	0.34	0.000958585	0.002343764
399512	2220	SLC25A35	0.34	0.009604832	0.019535102
126003	710	TRAPPC5	0.34	0.001254901	0.003004188
55745	3709	AP5M1	0.34	2.83E-05	8.68E-05
8195	2960	MKKS	0.34	0.001743195	0.004065243
2773	4748	GNAI3	0.34	1.69E-06	5.95E-06
8323	3450	FZD6	0.34	4.08E-07	1.54E-06
37	2291	ACADVL	0.34	1.85E-08	7.96E-08
83787	2213	ARMC10	0.34	0.000826152	0.002040743
7286	3069	TUFT1	0.34	0.000288615	0.000766993
2287	1295	FKBP3	0.34	4.97E-05	0.000147474
6748	734	SSR4	0.34	0.001090964	0.002643066
8493	4790	PPM1D	0.34	0.000357376	0.000936558
84326	798	METTL26	0.34	0.006906372	0.014445253
10181	3694	RBM5	0.34	0.000232308	0.000625212
10952	583	SEC61B	0.34	0.000198833	0.000540606
901	2651	CCNG2	0.34	3.89E-05	0.000117008
2530	3801	FUT8	0.34	1.50E-05	4.76E-05
114991	8901	ZNF618	0.34	8.20E-06	2.69E-05
23325	5737	WASHC4	0.34	1.58E-05	4.99E-05
51014	4063	TMED7	0.34	7.89E-07	2.88E-06
3214	2042	HOXB4	0.34	0.019351731	0.036992098
23327	8363	NEDD4L	0.34	6.80E-06	2.26E-05
9906	6136	SLC35E2	0.34	0.000454313	0.001172438
9219	3007	MTA2	0.34	2.32E-07	8.95E-07
6879	2310	TAF7	0.34	8.60E-06	2.82E-05
90410	937	IFT20	0.34	0.008282448	0.01705639
10848	3162	PPP1R13L	0.34	0.000220132	0.000594513
57563	5168	KLHL8	0.34	0.000198077	0.000538797
129303	1684	TMEM150A	0.34	0.021754842	0.04107758
64745	1639	METTL17	0.34	0.000224311	0.00060479
7855	6584	FZD5	0.34	0.00136991	0.003258006
7163	4035	TPD52	0.34	6.63E-05	0.000192938
51070	1152	NOSIP	0.34	0.002554466	0.005784089
28958	808	COA3	0.34	0.000514865	0.001315898
5993	3620	RFX5	0.34	8.98E-06	2.93E-05
51	7565	ACOX1	0.34	0.000285029	0.000758143
51575	3446	ESF1	0.34	0.018822921	0.03608996
79020	1866	C7orf25	0.34	0.006315394	0.013317341
5691	809	PSMB3	0.34	4.79E-05	0.000142425

284459	3128	HKR1	0.34	0.002108502	0.004844047
65992	1335	DDRGK1	0.34	8.16E-05	0.000234658
220002	2771	CYB561A3	0.34	0.000307222	0.000812309
6451	2059	SH3BGRL	0.34	1.03E-05	3.34E-05
9913	4637	SUPT7L	0.34	0.000179281	0.000490977
1329	523	COX5B	0.34	0.000349718	0.000917434
84954	1638	MPND	0.34	0.01485859	0.029102329
51111	3715	KMT5B	0.34	5.34E-05	0.00015764
2631	2013	NIPSNAP2	0.33	0.000463514	0.001193929
253982	1502	ASPHD1	0.33	0.019953649	0.038018062
3959	2277	LGALS3BP	0.33	1.41E-05	4.50E-05
153642	3330	ARSK	0.33	0.007820323	0.016179728
1.02E+08	1591	LOC101929959	0.33	0.011026379	0.022149996
3953	4675	LEPR	0.33	0.00156421	0.00368069
51368	1380	TEX264	0.33	0.000591733	0.001499204
116447	1962	TOP1MT	0.33	0.008791679	0.018021522
125488	3369	TTC39C	0.33	5.12E-05	0.000151635
64400	2479	AKTIP	0.33	0.006775981	0.014209326
84967	939	LSM10	0.33	0.022449811	0.042259588
57688	4631	ZSWIM6	0.33	0.001056143	0.002565
81848	5067	SPRY4	0.33	0.022216256	0.041868735
283232	1574	TMEM80	0.33	0.010210653	0.020656142
283871	3136	PGP	0.33	0.001263508	0.003021942
143279	3936	HECTD2	0.33	0.001957273	0.004521755
3070	3141	HELLS	0.33	5.57E-05	0.000163882
5352	3747	PLOD2	0.33	4.91E-08	2.03E-07
51013	1125	EXOSC1	0.33	0.007549707	0.015670935
9092	2500	SART1	0.33	3.25E-06	1.11E-05
27236	2995	ARFIP1	0.33	0.000205888	0.000558591
84445	2982	LZTS2	0.33	3.92E-06	1.33E-05
89797	9091	NAV2	0.33	5.08E-05	0.000150544
51430	5443	SUCO	0.33	9.14E-05	0.000261076
55668	5120	GPATCH2L	0.33	0.000284619	0.000757166
9063	5911	PIAS2	0.33	0.000928216	0.002275776
6237	1003	RRAS	0.33	0.001192559	0.002868081
92344	2653	GORAB	0.33	0.023254458	0.043640178
54758	1981	KLHDC4	0.33	0.003361229	0.007441106
7390	1349	UROS	0.33	0.000346305	0.00090969
147339	5250	C18orf25	0.33	0.000362881	0.000950142
666	2535	BOK	0.33	0.000339476	0.000892941
51650	705	MRPS33	0.33	0.006917192	0.01446618
8517	2213	IKBKG	0.33	0.002128232	0.004886215
1595	3046	CYP51A1	0.33	0.000496545	0.001272003
148534	6845	TMEM56	0.33	0.001037088	0.0025201
4641	4746	MYO1C	0.33	4.71E-08	1.94E-07
3155	1563	HMGCL	0.33	0.003893692	0.008530595
11112	2012	HIBADH	0.33	8.93E-05	0.000255409
10096	5708	ACTR3	0.33	2.20E-06	7.67E-06

9462	10156	RASAL2	0.33	0.00029812	0.000790008
55758	3868	RCOR3	0.33	0.000160874	0.000443852
669	1804	BPGM	0.33	0.008528341	0.017516074
114803	7703	MYSM1	0.33	0.007781317	0.016115916
4205	5393	MEF2A	0.33	5.86E-05	0.000171834
2023	2062	ENO1	0.33	1.49E-07	5.85E-07
85014	875	TMEM141	0.33	0.007832015	0.016198259
1647	1384	GADD45A	0.33	5.69E-05	0.000167059
9860	9928	LRIG2	0.33	0.01468366	0.02879683
55093	1593	WDYHV1	0.33	0.024186645	0.045203357
80324	1462	PUS1	0.33	0.016929704	0.032824856
3987	4252	LIMS1	0.33	1.77E-05	5.58E-05
55907	1814	CMAS	0.33	0.001211387	0.002909818
9013	3819	TAF1C	0.32	0.001716125	0.004007901
79789	8883	CLMN	0.32	0.000894854	0.002198225
4725	581	NDUFS5	0.32	0.000719323	0.001797598
5510	1436	PPP1R7	0.32	0.000199117	0.000541297
80013	2599	MINDY3	0.32	0.001572509	0.003698747
56180	2350	MOSPD1	0.32	0.019175405	0.036690566
2650	5550	GCNT1	0.32	0.000133474	0.000372128
55257	2845	MRGBP	0.32	0.002939247	0.006577397
1.01E+08	2168	TIMM23B	0.32	0.01725714	0.033383219
119710	884	C11orf74	0.32	0.012901004	0.02556033
64423	4302	INF2	0.32	4.73E-06	1.59E-05
51084	1469	CRYL1	0.32	0.014592614	0.028643567
95	1678	ACY1	0.32	0.008380393	0.017240109
7015	4018	TERT	0.32	5.05E-05	0.000149601
5828	4389	PEX2	0.32	0.001914679	0.004431998
90313	1593	TP53I13	0.32	0.000156599	0.000432394
10521	4806	DDX17	0.32	0.000203338	0.000552095
27095	1343	TRAPPC3	0.32	0.00163564	0.003833032
10772	5740	SRSF10	0.32	0.000773985	0.001922842
219402	1065	MTIF3	0.32	0.004720996	0.010205648
10011	1780	SRA1	0.32	0.000127681	0.000356647
26268	2332	FBXO9	0.32	0.000513124	0.001311636
10142	12797	AKAP9	0.32	0.000103139	0.000292791
308	1623	ANXA5	0.32	1.40E-07	5.51E-07
83862	1494	TMEM120A	0.32	0.014343674	0.028186066
84542	4523	KIAA1841	0.32	0.00309214	0.006896089
5696	1188	PSMB8	0.32	0.000344726	0.000905944
80005	9623	DOCK5	0.32	0.001891925	0.004385855
55644	1642	OSGEP	0.32	0.006092635	0.012891406
4697	2058	NDUFA4	0.32	0.000330623	0.000871071
10651	1422	MTX2	0.32	0.001483233	0.003508264
64432	4602	MRPS25	0.32	0.002732322	0.006148417
91582	942	RPS19BP1	0.32	0.018702211	0.0358895
5876	1531	RABGGTB	0.32	0.005277179	0.011309241
56996	3015	SLC12A9	0.32	0.005803406	0.012331209

2717	1418	GLA	0.32	0.00953884	0.01942089
171546	2538	SPTSSA	0.32	0.00037925	0.000990813
5209	4357	PFKFB3	0.32	7.11E-07	2.61E-06
326625	2071	MMAB	0.32	0.011548174	0.02308076
8850	4322	KAT2B	0.32	0.001646197	0.003856247
79646	3404	PANK3	0.32	0.001195483	0.002874724
84266	1098	ALKBH7	0.32	0.008556194	0.01757125
824	3368	CAPN2	0.32	8.47E-08	3.42E-07
376497	3612	SLC27A1	0.32	0.02091443	0.039633916
92170	1614	MTG1	0.32	0.001622602	0.00380549
4016	1991	LOXL1	0.32	0.00048836	0.001252119
7518	1714	XRCC4	0.32	0.020530998	0.038987444
8844	5769	KSR1	0.32	0.007756288	0.016067824
23030	5602	KDM4B	0.31	2.50E-05	7.70E-05
9828	6585	ARHGEF17	0.31	4.27E-05	0.000127631
8609	7263	KLF7	0.31	0.000248236	0.000665704
221092	5156	HNRNPUL2	0.31	3.48E-07	1.32E-06
10471	613	PFDN6	0.31	0.00231172	0.005270709
8828	6502	NRP2	0.31	0.00012491	0.000349403
26578	1304	OSTF1	0.31	0.026393439	0.048992752
1514	1640	CTSL	0.31	0.00034674	0.000910699
301	1555	ANXA1	0.31	2.11E-06	7.37E-06
84220	6904	RGPD5	0.31	0.003976482	0.008700177
285527	8899	FRYL	0.31	0.000108824	0.000307651
27327	8156	TNRC6A	0.31	0.000241013	0.000647266
4715	735	NDUFB9	0.31	0.001464331	0.003465862
1019	2020	CDK4	0.31	2.57E-05	7.92E-05
124401	2310	ANKS3	0.31	0.016638183	0.032305456
23143	4739	LRCH1	0.31	0.000827736	0.002044088
9616	2008	RNF7	0.31	0.001172684	0.002824871
80777	4286	CYB5B	0.31	1.24E-05	3.97E-05
6647	981	SOD1	0.31	1.51E-05	4.79E-05
54458	1260	PRR13	0.31	0.00126709	0.003029694
124540	5873	MSI2	0.31	1.58E-06	5.60E-06
154810	8925	AMOTL1	0.31	5.71E-05	0.000167554
3077	2435	HFE	0.31	0.016040001	0.031232738
55971	3682	BAIAP2L1	0.31	9.84E-05	0.000280029
55293	3656	UEVLD	0.31	0.002352726	0.005359389
29915	5806	HCFC2	0.31	0.026290168	0.048811254
55316	2550	RSAD1	0.31	0.002129213	0.004887205
283643	1664	TEDC1	0.31	0.022232529	0.04189496
118813	2874	ZFYVE27	0.31	0.001611866	0.003782308
54936	1629	ADPRHL2	0.31	0.002558942	0.005791274
8412	3126	BCAR3	0.31	0.003245417	0.007212579
4502	466	MT2A	0.31	0.000805766	0.001993991
80821	12999	DDHD1	0.31	0.012868521	0.025507349
1030	3878	CDKN2B	0.31	0.004445805	0.009651989
7159	4309	TP53BP2	0.31	3.37E-05	0.000102094

22944	3921	KIN	0.31	0.014727731	0.028867327
10892	5005	MALT1	0.31	0.00015427	0.000426626
57560	4258	IFT80	0.31	0.000677071	0.001698221
29093	3187	MRPL22	0.31	0.002525344	0.005723255
284695	7108	ZNF326	0.31	0.00064959	0.001632981
9479	3234	MAPK8IP1	0.31	0.022162666	0.041781035
79582	1537	SPAG16	0.31	0.002932812	0.006564652
55768	2453	NGLY1	0.31	0.004857252	0.010477558
159013	3755	CXorf38	0.31	0.004940929	0.010638685
51626	1377	DYNC2LI1	0.31	0.019839905	0.037827602
3661	1514	IRF3	0.31	0.000177947	0.000487626
54541	1752	DDIT4	0.31	0.000154075	0.00042622
121227	3930	LRIG3	0.31	0.003655175	0.008053714
9529	4876	BAG5	0.31	0.000388272	0.001013042
80169	6949	CTC1	0.31	0.005859317	0.012435301
10146	4493	G3BP1	0.31	2.65E-07	1.02E-06
6388	1423	SDF2	0.31	0.001849899	0.004294917
54732	1402	TMED9	0.31	8.75E-05	0.00025073
9867	4878	PJA2	0.31	7.01E-06	2.32E-05
22796	2977	COG2	0.31	0.006629237	0.013931198
28232	4532	SLCO3A1	0.31	0.000710981	0.001778253
57478	10200	USP31	0.31	0.00194183	0.004488413
1203	2817	CLN5	0.31	0.004666758	0.010104675
56948	1240	SDR39U1	0.31	0.000533452	0.001360222
220869	1143	CBWD5	0.30	0.006526645	0.013731844
5160	2160	PDHA1	0.30	1.93E-05	6.03E-05
5034	2596	P4HB	0.30	1.47E-07	5.80E-07
121457	2219	IKBIP	0.30	5.24E-05	0.000154955
6827	1545	SUPT4H1	0.30	0.000600367	0.001519129
8674	5152	VAMP4	0.30	0.009362599	0.019086125
54989	5416	ZNF770	0.30	7.29E-05	0.000210959
55362	3333	TMEM63B	0.30	0.000409175	0.001063832
54676	3008	GTPBP2	0.30	5.36E-05	0.000158132
10755	1812	GIPC1	0.30	0.000251418	0.000673888
9527	5327	GOSR1	0.30	7.99E-05	0.000230291
26088	3314	GGA1	0.30	0.001736622	0.004052041
10239	5934	AP3S2	0.30	0.000667635	0.001676447
4848	3102	CNOT2	0.30	9.16E-06	2.98E-05
51651	997	PTRH2	0.30	0.0021342	0.004897388
253769	4076	WDR27	0.30	0.013926951	0.027436974
57666	4610	FBRSL1	0.30	0.004898052	0.010551478
10994	2376	ILVBL	0.30	0.001890981	0.00438457
5877	3227	RABIF	0.30	0.018186668	0.034968154
984	3100	CDK11B	0.30	4.80E-05	0.000142838
3920	4557	LAMP2	0.30	1.52E-06	5.39E-06
57728	4336	WDR19	0.30	0.011452794	0.022913325
56204	4211	FAM214A	0.30	0.005639408	0.012002995
7468	6343	NSD2	0.30	2.72E-05	8.34E-05

80097	966	MZT2B	0.30	0.000515213	0.001316597
840	2521	CASP7	0.30	0.0032516	0.007223609
6611	1867	SMS	0.30	4.37E-06	1.48E-05
1911	5045	PHC1	0.30	0.001226754	0.002942355
83594	3514	NUDT12	0.30	0.022625328	0.042558419
10558	2790	SPTLC1	0.30	0.000105501	0.000298745
5214	2484	PFKP	0.30	6.22E-06	2.07E-05
79706	2181	PRKRIP1	0.30	0.001918728	0.004440215
6788	2901	STK3	0.30	0.007233159	0.015068445
5271	3109	SERPINB8	0.30	0.015119694	0.029567106
153339	4608	TMEM167A	0.30	5.97E-05	0.000174819
9204	5159	ZMYM6	0.30	0.01962245	0.037461233
55294	3884	FBXW7	0.30	0.002951472	0.00660143
7328	4990	UBE2H	0.30	2.10E-06	7.36E-06
7776	7973	ZNF236	0.30	0.004887317	0.010533461
4522	3466	MTHFD1	0.30	2.72E-06	9.38E-06
55082	1772	ARGLU1	0.30	0.000325522	0.000858525
516	600	ATP5MC1	0.30	0.002869926	0.006435218
57038	2177	RARS2	0.30	0.000602017	0.001522437
353376	3203	TICAM2	0.30	0.026474651	0.04910759
118487	844	CHCHD1	0.30	0.019356404	0.036997052
54776	2964	PPP1R12C	0.30	0.000285117	0.000758264
10092	2085	ARPC5	0.30	0.002376561	0.005412297
84191	1129	FAM96A	0.29	0.000779381	0.001935166
51704	2927	GPRC5B	0.29	0.002482414	0.005632427
10488	1868	CREB3	0.29	0.00096434	0.002355566
79007	2204	DBNDD1	0.29	0.012016033	0.023935075
55636	10472	CHD7	0.29	0.000979808	0.002391705
463	13515	ZFHX3	0.29	0.001376205	0.003271663
2052	1712	EPHX1	0.29	0.000274765	0.000732377
1832	9142	DSP	0.29	2.65E-05	8.14E-05
10473	1991	HMGN4	0.29	8.00E-05	0.000230291
55207	3008	ARL8B	0.29	0.000117297	0.000329873
55862	2230	ECHDC1	0.29	0.000417073	0.001082781
1389	3823	CREBL2	0.29	0.001024593	0.002493148
65117	2301	RSRC2	0.29	0.000152293	0.000421814
57407	1312	NMRAL1	0.29	0.001041504	0.002530486
1E+08	11357	NBPF10	0.29	0.009683461	0.019683759
10643	4172	IGF2BP3	0.29	0.000189165	0.000516138
51643	1876	TMBIM4	0.29	0.005662718	0.012048278
9043	8167	SPAG9	0.29	0.000164769	0.000453964
55188	4569	RIC8B	0.29	0.009100063	0.018593596
84265	1189	POLR3GL	0.29	0.017343379	0.033531791
9668	4578	ZNF432	0.29	0.025433922	0.047365048
51633	3306	OTUD6B	0.29	0.01136376	0.022748004
199990	917	FAAP20	0.29	0.007011126	0.014654005
6093	5388	ROCK1	0.29	1.89E-05	5.92E-05
23443	5809	SLC35A3	0.29	0.00441292	0.009586596

26128	2589	KIF1BP	0.29	0.000512728	0.001310813
79659	12799	DYNC2H1	0.29	0.005931236	0.012572918
64857	7713	PLEKHG2	0.29	0.006129308	0.012961859
221443	1202	OARD1	0.29	0.027005878	0.049993794
1643	1848	DDB2	0.29	0.001519307	0.003585476
11285	1824	B4GALT7	0.29	0.007196647	0.014999416
833	2570	CARS	0.29	0.001805096	0.004198029
7376	2078	NR1H2	0.29	0.001537354	0.003621812
5832	3252	ALDH18A1	0.29	1.64E-05	5.17E-05
1345	874	COX6C	0.29	0.010095929	0.020440344
114825	3350	PWWP2A	0.29	0.005721098	0.012165207
9455	2896	HOMER2	0.29	0.000533509	0.001360222
51141	2505	INSIG2	0.29	0.00800002	0.016506366
144404	6361	TMEM120B	0.29	0.006252959	0.013198215
9692	2260	KIAA0391	0.29	0.001532387	0.003611547
1.01E+08	925	TMEM35B	0.29	0.020297895	0.038593151
159090	3473	FAM122B	0.29	0.002139975	0.004910004
54811	6163	ZNF562	0.29	0.004980429	0.010713348
9516	2418	LITAF	0.29	0.000209379	0.000567025
11132	2650	CAPN10	0.29	0.017794244	0.034321334
9185	7554	REPS2	0.29	0.003126372	0.006968061
29907	1937	SNX15	0.29	0.006039681	0.012793641
114908	3605	TMEM123	0.29	2.12E-06	7.40E-06
6120	3920	RPE	0.29	0.000642522	0.001616815
56927	1970	GPR108	0.29	0.001157204	0.002789473
23534	4403	TNPO3	0.29	0.000125932	0.000352095
8932	5190	MBD2	0.29	0.000128517	0.000358756
23788	2628	MTCH2	0.29	0.000239056	0.000642497
26499	1564	PLEK2	0.29	0.005113511	0.010973051
200185	577	KRTCAP2	0.29	0.001023665	0.002491232
4627	7553	MYH9	0.28	8.90E-07	3.23E-06
51292	1803	GMPR2	0.28	0.000747672	0.001863986
4643	5125	MYO1E	0.28	8.15E-06	2.67E-05
10949	2983	HNRNPA0	0.28	0.00025152	0.000674059
23731	7849	TMEM245	0.28	9.85E-06	3.20E-05
80227	1598	PAAF1	0.28	0.020260082	0.038533626
7128	4576	TNFAIP3	0.28	0.002580134	0.005837003
115825	5927	WDFY2	0.28	0.02606805	0.048449488
9520	4353	NPEPPS	0.28	7.34E-05	0.000212396
401505	774	TOMM5	0.28	0.001398205	0.003320409
4869	1432	NPM1	0.28	4.58E-07	1.72E-06
10606	3280	PAICS	0.28	4.11E-06	1.40E-05
64747	2375	MFSD1	0.28	0.002470368	0.005607244
120526	3162	DNAJC24	0.28	0.01224477	0.024357934
27125	8659	AFF4	0.28	1.40E-05	4.47E-05
79694	4633	MANEA	0.28	0.003067257	0.006845751
9453	2757	GGPS1	0.28	0.005347605	0.011446362
27243	952	CHMP2A	0.28	0.001502501	0.003550059

2987	1121	GUK1	0.28	0.003999076	0.008744225
4329	4634	ALDH6A1	0.28	0.009431663	0.019220299
10010	2045	TANK	0.28	0.002777107	0.006238922
63875	2366	MRPL17	0.28	0.003268028	0.007257382
84450	3569	ZNF512	0.28	0.00378583	0.008317872
2729	3573	GCLC	0.28	0.008382219	0.017241869
22838	3894	RNF44	0.28	0.004821865	0.010412603
599	3576	BCL2L2	0.28	0.004896131	0.010549898
54482	3766	TRMT13	0.28	0.01954321	0.037326161
8603	4742	FAM193A	0.28	0.002725627	0.006135682
10611	5599	PDLIM5	0.28	0.000108993	0.00030808
285203	3702	EOGT	0.28	0.010639452	0.021435738
1603	711	DAD1	0.28	8.04E-05	0.00023139
80344	3939	DCAF11	0.28	0.000709647	0.001775418
3073	2751	HEXA	0.28	4.80E-05	0.000142846
129787	2138	TMEM18	0.28	0.003659687	0.008062656
57679	4685	ALS2	0.28	0.005236368	0.011228553
5279	1514	PIGC	0.28	0.007946701	0.016414436
966	7649	CD59	0.28	3.12E-05	9.51E-05
5873	3467	RAB27A	0.28	0.000176715	0.000484547
29081	849	METTL5	0.28	0.009814871	0.019921251
9255	3167	AIMP1	0.28	0.000962807	0.002352667
23176	4270	SEPT8	0.28	8.78E-05	0.000251541
509	1185	ATP5F1C	0.28	5.17E-05	0.000152922
91612	3623	CHURC1	0.28	0.009670575	0.019662065
694	4704	BTG1	0.28	0.000543817	0.001384716
51522	1187	TMEM14C	0.28	3.26E-05	9.89E-05
55869	1837	HDAC8	0.28	0.010135364	0.020513172
255520	4735	ELMOD2	0.28	0.002220123	0.005077505
1266	2114	CNN3	0.28	0.000254098	0.000680556
51631	2656	LUC7L2	0.28	0.004691725	0.010146786
55250	2622	ELP2	0.28	0.011295167	0.022630546
10443	3152	N4BP2L2	0.28	0.001264018	0.003022755
30001	5484	ERO1A	0.28	1.84E-05	5.80E-05
117246	3586	FTSJ3	0.28	0.001216427	0.002919952
4609	2379	MYC	0.28	0.001114347	0.002694206
5601	3792	MAPK9	0.28	0.001177394	0.002834381
253260	9505	RICTOR	0.28	0.004120264	0.008985989
10899	1574	JTB	0.28	0.000768784	0.00191206
5315	2402	PKM	0.27	2.10E-05	6.54E-05
51720	2066	UIMC1	0.27	0.00466763	0.010105331
23029	1828	RBM34	0.27	0.001214433	0.002916347
2317	9479	FLNB	0.27	9.13E-06	2.97E-05
57648	3819	KIAA1522	0.27	6.39E-05	0.00018639
27173	2187	SLC39A1	0.27	3.75E-05	0.000112825
55621	2181	TRMT1	0.27	0.002755152	0.006194298
7706	5744	TRIM25	0.27	0.00088593	0.00217781
5515	2643	PPP2CA	0.27	9.31E-05	0.000265815

2060	4908	EPS15	0.27	0.001116925	0.002699703
9131	2280	AIFM1	0.27	0.001632465	0.003826602
200894	3582	ARL13B	0.27	0.003256433	0.007233442
9489	4236	PGS1	0.27	0.008402109	0.017274782
23175	5103	LPIN1	0.27	0.00593253	0.012573886
6601	4946	SMARCC2	0.27	0.002180251	0.004993388
57003	3345	CCDC47	0.27	0.000570238	0.001448048
8565	2410	YARS	0.27	8.53E-05	0.00024466
51495	3213	HACD3	0.27	6.23E-05	0.000181943
23184	4292	MESD	0.27	0.000202023	0.000548775
57192	2118	MCOLN1	0.27	0.020275955	0.03855935
91445	3189	RNF185	0.27	0.00134133	0.003193883
5445	1633	PON2	0.27	8.81E-05	0.000252291
57017	1699	COQ9	0.27	0.005318543	0.011393763
80020	4841	FOXRED2	0.27	0.001520075	0.003586337
3308	3385	HSPA4	0.27	3.21E-05	9.74E-05
55735	3316	DNAJC11	0.27	0.000310334	0.000819928
55505	552	NOP10	0.27	0.010547018	0.021271217
522	920	ATP5PF	0.27	0.002520823	0.005713738
1848	3395	DUSP6	0.27	0.010605404	0.021376838
55737	4231	VPS35	0.27	3.85E-05	0.000115833
55620	1463	STAP2	0.27	0.009895143	0.02006584
283450	15450	HECTD4	0.27	0.010744692	0.021625702
1891	1275	ECH1	0.27	0.001888419	0.004379201
7347	972	UCHL3	0.27	0.024213602	0.045248978
8625	1169	RFXANK	0.27	0.004676517	0.010120872
729991	1457	BORCS8	0.27	0.019256163	0.036825262
29904	7343	EEF2K	0.27	0.006832385	0.014310718
257397	6575	TAB3	0.27	0.002182549	0.004998005
26205	4221	GMEB2	0.27	0.012406448	0.024657469
23201	6684	FAM168A	0.27	5.81E-05	0.000170421
7485	1513	WRB	0.27	0.005499561	0.011730637
9652	5704	TTC37	0.27	0.00037131	0.000970638
8996	1838	NOL3	0.27	0.020570918	0.039057926
5019	3572	OXCT1	0.27	0.000329125	0.000867382
27434	2516	POLM	0.27	0.018853671	0.036129425
2037	4236	EPB41L2	0.27	0.000335209	0.00088224
91683	3444	SYT12	0.27	0.009854378	0.019999154
81892	419	SLIRP	0.27	0.014972084	0.029317168
5037	1688	PEBP1	0.27	0.000187169	0.000511003
60558	4189	GUF1	0.27	0.003384618	0.007490086
81847	2681	RNF146	0.27	0.012470783	0.024774249
8816	5637	DCAF5	0.27	0.001558629	0.003668614
169792	6801	GLIS3	0.27	0.001820959	0.004233192
4074	2556	M6PR	0.27	0.000221637	0.000598304
6016	3401	RIT1	0.27	0.003146309	0.007006344
56994	1567	CHPT1	0.27	0.02140853	0.040475289
7534	3050	YWHAZ	0.27	8.43E-06	2.76E-05

2870	2570	GRK6	0.27	0.003128915	0.00697198
55236	6341	UBA6	0.27	0.000326377	0.000860396
113246	778	C12orf57	0.27	0.004446282	0.009651989
83640	1588	FAM103A1	0.27	0.012467137	0.024769773
8861	2976	LDB1	0.26	0.00293811	0.006575682
51160	1001	VPS28	0.26	0.010455008	0.02110002
388969	4023	C2orf68	0.26	0.002711825	0.006109922
644	1094	BLVRA	0.26	0.001640612	0.003843669
10605	2539	PAIP1	0.26	0.000270088	0.000720667
79735	2359	TBC1D17	0.26	0.005626279	0.011982228
55238	3869	SLC38A7	0.26	0.007492568	0.01556688
5613	6084	PRKX	0.26	0.006681564	0.014034523
9909	5129	DENND4B	0.26	0.004173535	0.009095788
3099	6419	HK2	0.26	0.011444004	0.022900896
84888	4576	SPPL2A	0.26	0.000719928	0.001798857
9526	1547	MPDU1	0.26	0.000503797	0.001289465
11193	2353	WBP4	0.26	0.007173044	0.014951976
2125	6629	EVPL	0.26	0.007038154	0.014703009
10714	3325	POLD3	0.26	0.001444019	0.00342307
147991	5750	DPY19L3	0.26	0.007358516	0.015313436
10623	2640	POLR3C	0.26	0.004613975	0.009998915
54680	4887	ZNHIT6	0.26	0.007942482	0.016407629
85015	5522	USP45	0.26	0.024181692	0.045198853
79085	3423	SLC25A23	0.26	0.010395625	0.020990742
283638	6132	CEP170B	0.26	0.00498261	0.010716743
311	2531	ANXA11	0.26	0.000681496	0.001708596
3257	2735	HPS1	0.26	0.015079238	0.029494485
5451	13881	POU2F1	0.26	0.006081242	0.012870936
353116	2064	RILPL1	0.26	0.025133477	0.046859561
84458	8284	LCOR	0.26	0.005135679	0.011019291
8086	1848	AAAS	0.26	0.002144846	0.004916736
2965	3210	GTF2H1	0.26	0.006534591	0.013746934
90338	4669	ZNF160	0.26	0.013241263	0.026170274
9522	4801	SCAMP1	0.26	0.001091609	0.002644268
4092	2468	SMAD7	0.26	0.010042798	0.020342048
1E+08	881	TOMM6	0.26	0.00756779	0.015706634
1785	3674	DNM2	0.26	4.25E-05	0.000127162
79158	5595	GNPTAB	0.26	0.001422274	0.003373961
9541	1809	CIR1	0.26	0.000597699	0.001513239
91748	7496	ELMSAN1	0.26	0.002217561	0.005072301
9879	4178	DDX46	0.26	9.41E-05	0.000268501
56672	1472	AKIP1	0.26	0.020461264	0.038874651
4792	1579	NFKBIA	0.25	0.000674987	0.001693471
64866	5925	CDCP1	0.25	0.000468908	0.001206772
2519	2548	FUCA2	0.25	0.000153655	0.000425257
103910	1021	MYL12B	0.25	6.71E-05	0.000195233
5792	7463	PTPRF	0.25	5.52E-05	0.000162605
9706	5138	ULK2	0.25	0.017373928	0.033579892

11103	3357	KRR1	0.25	0.001269454	0.003034121
10961	1381	ERP29	0.25	0.002258393	0.005159717
55311	2364	ZNF444	0.25	0.014859091	0.029102329
79001	1060	VKORC1	0.25	0.000686115	0.001719207
51422	2650	PRKAG2	0.25	0.000899825	0.002209827
64769	4780	MEAF6	0.25	0.004934172	0.010626711
55657	1960	ZNF692	0.25	0.026811615	0.049685939
51312	4151	SLC25A37	0.25	0.006612655	0.013897996
57020	3446	C16orf62	0.25	0.000939226	0.002301499
2959	1648	GTF2B	0.25	0.024661599	0.046028077
9557	2835	CHD1L	0.25	0.007077021	0.014772616
55749	4758	CCAR1	0.25	8.32E-05	0.000239035
7316	2594	UBC	0.25	0.000272265	0.00072604
51155	1611	JPT1	0.25	0.001897149	0.004396576
79622	1103	SNRNP25	0.25	0.018948749	0.036292053
9266	4364	CYTH2	0.25	0.005913818	0.01254105
28974	934	C19orf53	0.25	0.008162522	0.016823071
10644	3326	IGF2BP2	0.25	0.001901763	0.004405543
27339	2342	PRPF19	0.25	0.001696705	0.003965675
55275	7620	VPS53	0.25	0.002268031	0.005180405
51372	491	TMA7	0.25	0.017957581	0.034595067
8555	4896	CDC14B	0.25	0.012355187	0.024558335
9851	4465	KIAA0753	0.25	0.019962729	0.038029138
23243	6131	ANKRD28	0.25	0.002177999	0.004989517
3321	6790	IGSF3	0.25	0.001154932	0.00278513
274	2026	BIN1	0.25	0.000457845	0.001180868
1476	940	CSTB	0.25	0.009469566	0.019288691
27350	1127	APOBEC3C	0.25	0.018536531	0.035598474
10550	2135	ARL6IP5	0.25	0.000239753	0.000644273
55900	2575	ZNF302	0.25	0.011561919	0.023103035
51371	1477	POMP	0.25	0.007306975	0.01521152
7388	632	UQCRH	0.25	0.00742158	0.015433829
221035	5410	REEP3	0.25	0.001185618	0.002852548
5286	7618	PIK3C2A	0.25	0.00373876	0.008227679
11214	9966	AKAP13	0.25	0.000463393	0.001193791
29965	2745	CDIP1	0.25	0.009186921	0.018755971
79886	2839	CAAP1	0.25	0.007806235	0.016155612
1.03E+08	4007	LOC102724985	0.25	0.001689246	0.003949279
23567	3384	ZNF346	0.25	0.019836431	0.037825038
8409	631	UXT	0.25	0.01062143	0.021401856
873	1331	CBR1	0.25	0.005769576	0.012263886
9380	1257	GRHPR	0.25	0.002047567	0.004715085
64343	2545	AZI2	0.25	0.015764088	0.030732533
5211	2921	PFKL	0.24	0.000524508	0.001338617
7041	1812	TGFB1I1	0.24	0.005456709	0.011649021
9337	2466	CNOT8	0.24	0.002016891	0.00465102
119504	3411	ANAPC16	0.24	0.002287247	0.005220942
79585	3854	CORO7	0.24	0.018853235	0.036129425

25852	4182	ARMC8	0.24	0.00307044	0.006851995
5184	2015	PEPD	0.24	0.005651651	0.012026171
26234	3188	FBXL5	0.24	0.001572852	0.003699064
63891	4345	RNF123	0.24	0.011316956	0.022669636
3488	6333	IGFBP5	0.24	0.021731084	0.041041444
399664	2822	MEX3D	0.24	0.016923228	0.032815881
53349	3726	ZFYVE1	0.24	0.025491917	0.047468075
57507	5325	ZNF608	0.24	0.001370468	0.003258897
7071	3116	KLF10	0.24	0.002770721	0.006225363
23234	2385	DNAJC9	0.24	0.00239302	0.005444196
79672	1844	FN3KRP	0.24	0.003807066	0.008357295
10432	5415	RBM14	0.24	0.001578405	0.003709669
348995	4327	NUP43	0.24	0.002569868	0.00581526
29803	3192	REPIN1	0.24	0.00127019	0.003035471
11072	1523	DUSP14	0.24	0.026420557	0.049037966
5791	4967	PTPRE	0.24	0.015950377	0.031065034
56850	3110	GRIPAP1	0.24	0.007378763	0.015350176
3856	1802	KRT8	0.24	0.000863758	0.002127132
327	2474	APEH	0.24	0.0033209	0.007360996
5719	1756	PSMD13	0.24	0.02169287	0.040973628
10489	2947	LRRC41	0.24	0.001705477	0.003984604
4714	761	NDUFB8	0.24	0.008943105	0.018300253
10093	1564	ARPC4	0.24	0.003760335	0.008269014
253143	10914	PRR14L	0.24	0.011363005	0.022748004
54978	3912	SLC35F6	0.24	0.003061569	0.006833913
9678	3901	PHF14	0.24	0.010021665	0.020301555
51520	4893	LARS	0.24	0.000469173	0.001207104
118	3985	ADD1	0.24	0.000250687	0.00067203
5612	3342	THAP12	0.24	0.00271212	0.006109922
9360	2966	PPIG	0.24	0.001077299	0.002612465
5292	2751	PIM1	0.24	0.008610531	0.017674667
158427	3951	TSTD2	0.24	0.01888682	0.036181243
51608	2106	GET4	0.24	0.011055583	0.022203639
6749	2829	SSRP1	0.24	4.41E-05	0.000131703
412	6451	STS	0.24	0.012959711	0.025648045
80145	1026	THOC7	0.23	0.007492395	0.01556688
56674	1933	TMEM9B	0.23	0.009126322	0.018642963
56851	1075	EMC7	0.23	0.002031579	0.004681854
518	2684	ATP5MC3	0.23	0.001924807	0.004453122
23582	1864	CCNDBP1	0.23	0.025751931	0.047907057
84056	7526	KATNAL1	0.23	0.007976837	0.016469027
10628	2888	TXNIP	0.23	0.000423787	0.001098766
4141	2862	MARS	0.23	0.001079174	0.002616286
54663	1472	WDR74	0.23	0.019218133	0.036760447
1312	1459	COMT	0.23	0.005387747	0.011517023
8777	7629	MPDZ	0.23	0.010667405	0.021482314
9322	2037	TRIP10	0.23	0.002705785	0.006098742
10960	1853	LMAN2	0.23	0.000288005	0.000765485

392	3458	ARHGAP1	0.23	0.001310557	0.003126055
11140	1696	CDC37	0.23	0.000450691	0.001163935
85415	3546	RHPN2	0.23	0.003793642	0.008332975
22848	21283	AAK1	0.23	0.00299566	0.006695206
23786	3489	BCL2L13	0.23	0.003359349	0.007437871
26263	3424	FBXO22	0.23	0.018149851	0.034908699
6238	3739	RRBP1	0.23	0.004683079	0.010132605
5927	10836	KDM5A	0.23	0.001612781	0.003783455
10645	4601	CAMKK2	0.23	0.013883218	0.027359917
4061	1192	LY6E	0.23	0.020277947	0.03855935
284352	2947	PPP1R37	0.23	0.011153909	0.022380876
28976	2559	ACAD9	0.23	0.006845689	0.014336893
5599	5972	MAPK8	0.23	0.014089796	0.027717837
152006	4892	RNF38	0.23	0.011210465	0.022481663
57140	3047	RNPEPL1	0.23	0.008862066	0.018155331
10120	2247	ACTR1B	0.23	0.00556006	0.011852567
159	2796	ADSS	0.23	0.00952396	0.019392817
5926	5783	ARID4A	0.23	0.009134955	0.018656309
2621	2551	GAS6	0.23	0.002883588	0.006462613
83990	5118	BRIP1	0.23	0.011992718	0.023894067
22985	2867	ACIN1	0.23	0.001010158	0.002460382
4731	1630	NDUFV3	0.23	0.017271008	0.033406409
11342	2702	RNF13	0.23	0.012087633	0.024069602
112398	2174	EGLN2	0.23	0.012871255	0.025509923
473	6698	RERE	0.23	0.014766885	0.028934496
7375	4076	USP4	0.23	0.008348278	0.01717802
55860	1726	ACTR10	0.23	0.012923003	0.025595353
9267	3602	CYTH1	0.23	0.011243632	0.022540546
25828	1356	TXN2	0.23	0.012108743	0.024108937
5165	10345	PDK3	0.23	0.008270395	0.017033543
6443	4295	SGCB	0.23	0.001650254	0.003864731
83440	2547	ADPGK	0.23	0.007817797	0.016176385
79581	1937	SLC52A2	0.22	0.017945318	0.034582691
23432	7909	GPR161	0.22	0.023087212	0.043353792
4924	2581	NUCB1	0.22	0.000311545	0.000822884
10490	1357	VTI1B	0.22	0.022037636	0.041571794
6687	3017	SPG7	0.22	0.015601253	0.030451835
5429	7316	POLH	0.22	0.003914085	0.008572102
10455	1411	ECI2	0.22	0.011365675	0.022749274
30850	3542	CDR2L	0.22	0.023868351	0.04467121
50717	3901	DCAF8	0.22	0.002010481	0.004638047
6429	2486	SRSF4	0.22	0.00250386	0.005678909
120	4387	ADD3	0.22	0.000210933	0.000570883
78992	2040	YIPF2	0.22	0.021326695	0.040346328
6164	576	RPL34	0.22	0.002282432	0.005210619
10299	5685	MARCH6	0.22	0.015117639	0.029566341
79811	3865	SLTM	0.22	0.001215435	0.002917964
11311	2434	VPS45	0.22	0.016599678	0.032237739

1936	1374	EEF1D	0.22	0.000611365	0.001543221
22795	5059	NID2	0.22	0.004423711	0.009608863
64853	3020	AIDA	0.22	0.005206635	0.011167491
64968	1017	MRPS6	0.22	0.01061252	0.021386328
25875	1868	LETMD1	0.22	0.026889393	0.049809293
10412	1212	NSA2	0.22	0.003271462	0.007263193
8775	1800	NAPA	0.22	0.005609645	0.011951099
10922	1916	FASTK	0.22	0.014493811	0.028459061
1540	8288	CYLD	0.22	0.013146567	0.025994681
64285	2925	RHBDF1	0.22	0.010903073	0.021928109
91252	2331	SLC39A13	0.22	0.004172921	0.009095788
6990	2230	DYNLT3	0.22	0.024055128	0.044976482
79036	1490	KXD1	0.22	0.011481469	0.022955186
376267	3314	RAB15	0.22	0.024218344	0.04525308
23089	6629	PEG10	0.22	0.002143645	0.004914616
3927	4134	LASP1	0.22	0.004108487	0.008962503
79650	2300	USB1	0.22	0.003082779	0.006876076
27336	2819	HTATSF1	0.22	0.002999522	0.006702995
1386	4150	ATF2	0.22	0.004802004	0.010373496
9948	3138	WDR1	0.21	0.001769665	0.004120484
1855	2939	DVL1	0.21	0.012146538	0.024178769
64976	1192	MRPL40	0.21	0.019412064	0.037095457
8615	4189	USO1	0.21	0.002853777	0.006401454
51009	4237	DERL2	0.21	0.006193493	0.013088247
7182	8294	NR2C2	0.21	0.011208434	0.022480127
3269	4333	HRH1	0.21	0.003858507	0.008459766
493869	3786	GPX8	0.21	0.008397376	0.017267049
79089	2251	TMUB2	0.21	0.023935173	0.044780473
5836	2849	PYGL	0.21	0.003855333	0.008453851
27020	2100	NPTN	0.21	0.001852421	0.004300212
153241	4505	CEP120	0.21	0.022599104	0.042513592
10956	2783	OS9	0.21	0.001499391	0.003543182
4711	1061	NDUFB5	0.21	0.022308938	0.042017987
6118	1813	RPA2	0.21	0.006270315	0.013228564
55332	3453	DRAM1	0.21	0.013340936	0.026349683
51538	1639	ZCCHC17	0.21	0.020215786	0.038453494
25782	7329	RAB3GAP2	0.21	0.010409464	0.021015265
29957	3342	SLC25A24	0.21	0.007789696	0.016127765
5424	3461	POLD1	0.21	0.016471433	0.032007474
10130	2349	PDIA6	0.21	0.000530753	0.001353972
1937	1552	EEF1G	0.21	0.000276115	0.000735753
1718	4286	DHCR24	0.21	0.000799392	0.001979044
25839	2843	COG4	0.21	0.018028259	0.034716171
27430	2172	MAT2B	0.21	0.009298673	0.018964511
54431	5875	DNAJC10	0.21	0.003286927	0.007292061
10548	2471	TM9SF1	0.21	0.00946604	0.01928372
8697	3169	CDC23	0.21	0.012250594	0.024366791
8562	2731	DENR	0.21	0.004152047	0.009054195

1195	1936	CLK1	0.21	0.026646897	0.0494013
10844	2347	TUBGCP2	0.21	0.007443503	0.015473986
7991	3367	TUSC3	0.20	0.004635065	0.010042171
7297	3862	TYK2	0.20	0.004597191	0.009963757
1017	2293	CDK2	0.20	0.004668706	0.010106429
5426	7622	POLE	0.20	0.011493091	0.022975838
81876	1959	RAB1B	0.20	0.009590449	0.019510316
25941	2692	TPGS2	0.20	0.011479299	0.022953432
860	4226	RUNX2	0.20	0.005374083	0.011490578
84164	2950	ASCC2	0.20	0.015142447	0.029608341
51126	1101	NAA20	0.20	0.018773014	0.036013411
55011	1244	PIH1D1	0.20	0.012913787	0.025582804
6905	1977	TBCE	0.20	0.026471454	0.049106785
139231	7302	FAM199X	0.20	0.011280467	0.022604188
4926	7191	NUMA1	0.20	0.002804333	0.006296106
51026	3269	GOLT1B	0.20	0.017524486	0.033841437
55366	5248	LGR4	0.20	0.008871342	0.018169647
10114	7195	HIPK3	0.20	0.011078489	0.022247128
4190	1646	MDH1	0.20	0.003876963	0.008496038
9138	3252	ARHGEF1	0.20	0.007736451	0.016032337
6747	4331	SSR3	0.20	0.001101436	0.002665529
6726	1533	SRP9	0.20	0.004288541	0.009331226
51808	3641	PHAX	0.20	0.009139359	0.018663157
9894	3307	TELO2	0.20	0.014674503	0.028782048
3032	2197	HADHB	0.20	0.006452735	0.013587339
51421	4491	AMOTL2	0.20	0.00379689	0.008338048
5589	2107	PRKCSH	0.20	0.007075005	0.014770142
9064	4016	MAP3K6	0.20	0.023582187	0.044213179
6625	1664	SNRNP70	0.19	0.002743378	0.006169389
27332	5313	ZNF638	0.19	0.010387039	0.020977144
4041	4422	LRP5	0.19	0.020433787	0.038826599
1635	2069	DCTD	0.19	0.015000738	0.029360335
7077	3670	TIMP2	0.19	0.003861241	0.008464654
23362	9474	PSD3	0.19	0.020776133	0.039401252
2597	1421	GAPDH	0.19	0.002035867	0.004690518
4659	5302	PPP1R12A	0.19	0.008705939	0.017858131
1186	4236	CLCN7	0.19	0.003689636	0.008125613
56937	4698	PMEPA1	0.19	0.019847163	0.03783738
57552	4286	NCEH1	0.19	0.009772685	0.019844694
57222	2837	ERGIC1	0.19	0.004281276	0.00931656
85437	1277	ZCRB1	0.19	0.026220545	0.048702346
51768	4369	TM7SF3	0.19	0.022898734	0.04301805
3516	5508	RBPJ	0.18	0.006854757	0.014354017
4779	4697	NFE2L1	0.18	0.001339066	0.003189774
1729	5699	DIAPH1	0.18	0.004840514	0.010442721
113829	2954	SLC35A4	0.18	0.018269642	0.035115837
3837	4016	KPNB1	0.18	0.001512754	0.003571908
6786	4064	STIM1	0.18	0.016506588	0.032067467

7536	3423	SF1	0.18	0.018271382	0.035115837
8729	5978	GBF1	0.18	0.024356875	0.045497577
7414	5444	VCL	0.18	0.001526108	0.003598658
6166	592	RPL36AL	0.18	0.008741718	0.017923246
23787	2291	MTCH1	0.18	0.005058441	0.010865375
207	2817	AKT1	0.18	0.006246058	0.013186783
10134	1468	BCAP31	0.18	0.017789765	0.03431642
51592	7824	TRIM33	0.18	0.016043185	0.031235515
7879	2240	RAB7A	0.18	0.017805924	0.034340134
4134	5969	MAP4	0.18	0.009019036	0.018440763
8496	5523	PPFIBP1	0.18	0.017962935	0.034601629
10695	1765	CNPY3	0.18	0.022855498	0.042950449
23317	6512	DNAJC13	0.18	0.023819173	0.044601019
10743	7651	RAI1	0.17	0.026652568	0.049405396
51752	5131	ERAP1	0.17	0.012697183	0.025193025
5713	1686	PSMD7	0.17	0.025167877	0.046913852
2181	4138	ACSL3	0.17	0.012421004	0.024683638
51164	3845	DCTN4	0.17	0.020965078	0.039717187
6301	1956	SARS	0.17	0.011809674	0.023542499
832	1788	CAPZB	0.17	0.02230964	0.042017987
6629	1656	SNRPB2	0.17	0.023662163	0.04433973
9989	3932	PPP4R1	0.17	0.015641246	0.03051984
135228	8893	CD109	0.17	0.013981145	0.027525427
9747	5236	TCAF1	0.17	0.011140359	0.022358737
57187	5987	THOC2	0.16	0.010811107	0.021754444
23344	4257	ESYT1	0.16	0.012432444	0.024703609
10923	3522	SUB1	0.16	0.021352048	0.040381393
81619	5399	TSPAN14	0.16	0.018056569	0.03476315
29968	2266	PSAT1	0.16	0.022005675	0.041515909
9076	3452	CLDN1	0.16	0.017035984	0.03300211
3428	2761	IFI16	0.16	0.018127335	0.034873836
51727	2956	CMPK1	0.16	0.015420644	0.030125783
8663	3111	EIF3C	0.15	0.018455028	0.035449614
55740	9129	ENAH	0.15	0.021903939	0.041332751
7357	3923	UGCG	0.14	0.026654667	0.049405396
3939	2225	LDHA	0.14	0.012746866	0.02528878
55752	5406	SEPT11	0.14	0.019954775	0.038018062
23193	3747	GANAB	0.14	0.007970711	0.016460203
3181	2932	HNRNPA2B1	0.14	0.017030352	0.032994799
4628	7698	MYH10	0.14	0.026271403	0.048783441
22883	5202	CLSTN1	0.14	0.017762402	0.034274801
4735	3276	SEPT2	-0.13	0.023702795	0.044411184
718	5148	C3	-0.14	0.010926893	0.021970017
1108	6462	CHD4	-0.14	0.022875076	0.042982694
1000	3947	CDH2	-0.14	0.020598113	0.039097028
1362	8549	CPD	-0.15	0.023549614	0.044161429
4174	2568	MCM5	-0.15	0.019773574	0.037711355
81552	2834	VOPP1	-0.15	0.025555952	0.047567691

8073	3857	PTP4A2	-0.16	0.012318866	0.024494362
6132	917	RPL8	-0.16	0.025298333	0.047132304
6651	4820	SON	-0.16	0.006471907	0.01362313
5728	8720	PTEN	-0.16	0.026999064	0.049986388
8669	2572	EIF3J	-0.16	0.025561169	0.047572102
29980	2538	DONSON	-0.16	0.02138569	0.040436409
54765	6077	TRIM44	-0.16	0.010383202	0.020971779
6400	7897	SEL1L	-0.16	0.021685279	0.040963644
2355	6477	FOSL2	-0.16	0.007108005	0.014830322
55353	2242	LAPTM4B	-0.16	0.006697453	0.014061248
6426	5524	SRSF1	-0.16	0.016955049	0.032866824
26064	4901	RAI14	-0.16	0.011769668	0.023470647
975	1324	CD81	-0.16	0.018778943	0.036013411
908	2679	CCT6A	-0.17	0.01607741	0.031291863
10762	4953	NUP50	-0.17	0.0181278	0.034873836
57492	6485	ARID1B	-0.17	0.025513464	0.047498243
1173	1956	AP2M1	-0.17	0.014687288	0.028800765
9993	4497	DGCR2	-0.17	0.015051863	0.029453911
2665	2232	GDI2	-0.17	0.009944549	0.020159126
5531	1420	PPP4C	-0.17	0.026548306	0.049233932
1292	3395	COL6A2	-0.17	0.015493463	0.030261389
9761	6337	MLEC	-0.17	0.004558859	0.009884297
7029	7658	TFDP2	-0.17	0.022922872	0.04305884
6427	2274	SRSF2	-0.17	0.023203003	0.043552816
6497	5706	SKI	-0.17	0.021179689	0.040081021
2590	4476	GALNT2	-0.17	0.015334247	0.029966883
9868	4381	TOMM70	-0.17	0.01949305	0.037242206
7707	9573	ZNF148	-0.17	0.018309845	0.035182149
55771	6487	PRR11	-0.17	0.023259469	0.043644972
23168	5038	RTF1	-0.17	0.008799104	0.018034662
57645	4359	POGK	-0.17	0.023427423	0.043955486
57132	3060	CHMP1B	-0.17	0.02596783	0.048273321
9601	2969	PDIA4	-0.17	0.020397924	0.038770893
6642	8325	SNX1	-0.17	0.02121589	0.040145254
27316	2089	RBMX	-0.18	0.002680456	0.006046255
8914	5015	TIMELESS	-0.18	0.025834142	0.048049935
9870	5438	AREL1	-0.18	0.026907787	0.04983817
9611	6261	NCOR1	-0.18	0.008000497	0.016506366
11333	2707	PDAP1	-0.18	0.022245928	0.041911318
23451	3998	SF3B1	-0.18	0.014994717	0.029351783
51441	2789	YTHDF2	-0.18	0.009018263	0.018440763
81929	3464	SEH1L	-0.18	0.024527484	0.045801822
8761	3199	PABPC4	-0.18	0.006323518	0.013332891
5361	9108	PLXNA1	-0.18	0.023166734	0.043489333
91039	4279	DPP9	-0.18	0.025502323	0.047482477
6175	1233	RPLP0	-0.18	0.006687965	0.014044648
10526	5030	IPO8	-0.18	0.019877718	0.037887501
2969	4475	GTF2I	-0.18	0.001275111	0.003046413

51187	1487	RSL24D1	-0.18	0.012940955	0.025616632
6738	7712	TROVE2	-0.18	0.014572058	0.028606377
51234	1036	EMC4	-0.18	0.020855177	0.039538495
22820	3114	COPG1	-0.18	0.004865745	0.010489512
2067	2569	ERCC1	-0.18	0.019908987	0.037938962
9640	7700	ZNF592	-0.18	0.024059421	0.044979776
29123	9272	ANKRD11	-0.18	0.004271282	0.009297088
57092	2386	PCNP	-0.18	0.018155302	0.034915403
64759	7575	TNS3	-0.18	0.003763473	0.008274891
79718	6957	TBL1XR1	-0.18	0.004200457	0.009148531
78996	2364	C7orf49	-0.19	0.01924653	0.036810802
262	3364	AMD1	-0.19	0.015517052	0.0303008
6135	643	RPL11	-0.19	0.002918328	0.006535525
10724	4956	MGEA5	-0.19	0.020597846	0.039097028
23248	6776	RPRD2	-0.19	0.020101612	0.038260894
4089	8789	SMAD4	-0.19	0.010316367	0.020855753
790	7091	CAD	-0.19	0.009417806	0.019196464
25912	1819	C1orf43	-0.19	0.009022527	0.018445778
103	6464	ADAR	-0.19	0.001647209	0.003858107
7106	1449	TSPAN4	-0.19	0.020597172	0.039097028
9782	4789	MATR3	-0.19	0.001036731	0.002519582
92799	2373	SHKBP1	-0.19	0.019723807	0.037626433
10274	4798	STAG1	-0.19	0.026499407	0.049148379
51637	1064	RTRAF	-0.19	0.006799346	0.014253278
57410	2651	SCYL1	-0.19	0.00898875	0.018385185
11078	3911	TRIOBP	-0.19	0.006231257	0.013161791
6205	646	RPS11	-0.19	0.019333141	0.036960539
5682	1248	PSMA1	-0.19	0.00903201	0.018460917
51692	2401	CPSF3	-0.19	0.012207803	0.024287117
10250	4048	SRRM1	-0.19	0.011580626	0.023137813
7353	1461	UFD1	-0.19	0.020520079	0.038973893
64710	6356	NUCKS1	-0.19	0.005017602	0.010782868
5977	2510	DPF2	-0.19	0.012877497	0.025519448
5701	2774	PSMC2	-0.19	0.008162507	0.016823071
11171	1919	STRAP	-0.19	0.013247627	0.02617994
29927	3656	SEC61A1	-0.19	0.002826512	0.006343498
5213	3049	PFKM	-0.19	0.02101961	0.039803521
1363	2648	CPE	-0.19	0.00349258	0.007714589
94056	3533	SYAP1	-0.19	0.009264883	0.018902107
4000	2335	LMNA	-0.19	0.014612478	0.028673054
161	4590	AP2A2	-0.19	0.010754097	0.021642179
4162	3325	MCAM	-0.19	0.00245903	0.005582221
79033	1309	ERI3	-0.19	0.019012508	0.036410244
26135	6722	SERBP1	-0.20	0.000796718	0.001973881
4125	3198	MAN2B1	-0.20	0.008838197	0.018110608
805	1395	CALM2	-0.20	0.003238131	0.007197288
5062	5850	PAK2	-0.20	0.007024471	0.014676718
90231	2797	KIAA2013	-0.20	0.022101735	0.041679434

27333	4220	GOLIM4	-0.20	0.023545037	0.044157507
9112	2824	MTA1	-0.20	0.01884082	0.036116484
80149	2730	ZC3H12A	-0.20	0.013946948	0.027467306
10376	1771	TUBA1B	-0.20	0.004091943	0.008929704
151987	4897	PPP4R2	-0.20	0.005480721	0.011696067
51691	12520	LSM8	-0.20	0.023723744	0.044441063
55700	3315	MAP7D1	-0.20	0.013601967	0.026832436
8498	3075	RANBP3	-0.20	0.014118286	0.027767733
26284	1919	ERAL1	-0.20	0.016982733	0.032913306
1786	5398	DNMT1	-0.20	0.00265152	0.005985543
55334	4590	SLC39A9	-0.20	0.006816756	0.014284924
31	9605	ACACA	-0.20	0.007162634	0.014935534
1209	2566	CLPTM1	-0.20	0.009541002	0.019423067
10121	2891	ACTR1A	-0.20	0.010903582	0.021928109
54439	6514	RBM27	-0.20	0.010856224	0.021837811
471	2105	ATIC	-0.20	0.017563843	0.033913631
4087	10440	SMAD2	-0.20	0.006427899	0.013541725
85379	10517	KIAA1671	-0.20	0.020725597	0.039313804
5714	1556	PSMD8	-0.20	0.011611908	0.023192489
3615	1716	IMPDH2	-0.20	0.009437239	0.019227252
6128	999	RPL6	-0.20	0.003054463	0.006818909
4705	4072	NDUFA10	-0.20	0.00733473	0.015265723
23413	4933	NCS1	-0.20	0.014957322	0.02929149
51433	2417	ANAPC5	-0.20	0.004181941	0.009111555
27258	695	LSM3	-0.20	0.026770162	0.049614294
5887	4062	RAD23B	-0.20	0.004074369	0.008896828
6432	1324	SRSF7	-0.20	0.006510554	0.013701235
3276	1401	PRMT1	-0.20	0.012800377	0.025386438
10425	4170	ARIH2	-0.20	0.004090788	0.008928279
23468	11564	CBX5	-0.20	0.000222122	0.000599524
57470	2694	LRRC47	-0.20	0.023941184	0.044782715
9950	2957	GOLGA5	-0.20	0.011581969	0.023137893
128637	4273	TBC1D20	-0.20	0.014131906	0.027788369
2926	2869	GRSF1	-0.20	0.004653137	0.010078866
51232	5127	CRIM1	-0.20	0.001463941	0.003465402
6141	893	RPL18	-0.20	0.003492361	0.007714589
23466	6140	CBX6	-0.21	0.005101445	0.010951128
79882	4234	ZC3H14	-0.21	0.004330743	0.009418437
22998	5942	LIMCH1	-0.21	0.007572282	0.015710718
8031	3533	NCOA4	-0.21	0.002556139	0.005786402
55421	4362	NCBP3	-0.21	0.008884348	0.018188397
9689	2869	BZW1	-0.21	0.001570083	0.003693529
6721	5006	SREBF2	-0.21	0.003782252	0.008311039
55662	6868	HIF1AN	-0.21	0.009633118	0.019588149
28972	1100	SPCS1	-0.21	0.004825247	0.010417375
64225	4071	ATL2	-0.21	0.017718527	0.034193851
80143	5508	SIKE1	-0.21	0.014465311	0.028412518
8175	1678	SF3A2	-0.21	0.02515539	0.046895496

89910	4474	UBE3B	-0.21	0.011449343	0.022909001
10945	1575	KDELR1	-0.21	0.003580372	0.007901633
1874	2100	E2F4	-0.21	0.021751126	0.04107493
6217	709	RPS16	-0.21	0.010321291	0.020863333
9646	4606	CTR9	-0.21	0.013107895	0.025926871
5757	1222	PTMA	-0.21	0.001154274	0.002784299
682	1759	BSG	-0.21	0.001199341	0.002883222
85461	6855	TANC1	-0.21	0.008891164	0.018200254
148362	3874	BROX	-0.21	0.00533613	0.011427305
124997	4835	WDR81	-0.21	0.022239692	0.041904013
55884	2724	WSB2	-0.21	0.016055932	0.031256908
79022	1515	TMEM106C	-0.21	0.015744909	0.030704565
10159	2044	ATP6AP2	-0.21	0.002209455	0.005055061
10856	1963	RUVBL2	-0.21	0.004411508	0.009584701
22828	4925	SCAF8	-0.21	0.005848636	0.012417083
54965	3052	PIGX	-0.21	0.019199271	0.036728321
6157	4906	RPL27A	-0.21	0.001012166	0.002464935
3337	2383	DNAJB1	-0.21	0.006244106	0.013184228
9749	9438	PHACTR2	-0.21	0.01967497	0.037549395
7465	3351	WEE1	-0.21	0.007747392	0.016053138
9811	5683	CTIF	-0.21	0.011466577	0.022933154
5128	3731	CDK17	-0.21	0.026124672	0.048537758
5250	1693	SLC25A3	-0.21	0.001867855	0.004332644
26003	2559	GORASP2	-0.21	0.005263567	0.011284153
55284	8428	UBE2W	-0.21	0.018136228	0.034886273
8648	6905	NCOA1	-0.21	0.0180633	0.034772342
7027	2591	TFDP1	-0.21	0.002024674	0.004666546
23645	2399	PPP1R15A	-0.21	0.009677372	0.019673631
11183	4285	MAP4K5	-0.22	0.004963167	0.010678803
9820	4960	CUL7	-0.22	0.011479053	0.022953432
10075	14397	HUWE1	-0.22	0.000892356	0.002193023
6161	1669	RPL32	-0.22	0.001577005	0.00370687
537	2105	ATP6AP1	-0.22	0.003870586	0.008483111
51465	4356	UBE2J1	-0.22	0.0071713	0.014951849
7384	1636	UQCRC1	-0.22	0.003145018	0.007004348
1399	5336	CRKL	-0.22	0.005024726	0.010796873
10401	2902	PIAS3	-0.22	0.02564264	0.047713732
3566	3184	IL4R	-0.22	0.007852325	0.016236484
7175	9727	TPR	-0.22	0.003585396	0.007910755
64841	3947	GNPNAT1	-0.22	0.026923433	0.049861951
51144	2302	HSD17B12	-0.22	0.00339309	0.007506029
8723	2534	SNX4	-0.22	0.018777729	0.036013411
10694	2008	CCT8	-0.22	0.000337874	0.000888988
55701	5132	ARHGEF40	-0.22	0.005917593	0.012546993
55660	4111	PRPF40A	-0.22	0.001238051	0.002967848
59345	6421	GNB4	-0.22	0.008516432	0.017497683
4176	2635	MCM7	-0.22	0.000334542	0.000880613
22980	2299	TCF25	-0.22	0.00168546	0.003941466

5690	4759	PSMB2	-0.22	0.002921248	0.006541238
81611	3429	ANP32E	-0.22	0.000429478	0.00111271
6136	674	RPL12	-0.22	0.000524024	0.001337574
8453	4244	CUL2	-0.22	0.00564548	0.012014479
375056	5887	MIA3	-0.22	0.006443907	0.013570621
6633	724	SNRPD2	-0.22	0.013486135	0.026618712
9342	4277	SNAP29	-0.22	0.020059828	0.038193637
79728	4096	PALB2	-0.22	0.018715155	0.035908813
54726	6944	OTUD4	-0.22	0.00872144	0.017885796
84146	3442	ZNF644	-0.22	0.008662988	0.017778236
1537	1276	CYC1	-0.22	0.010744349	0.021625702
55578	2937	SUPT20H	-0.22	0.005629366	0.011987365
10175	1423	CNIH1	-0.22	0.005368403	0.011479816
81620	2742	CDT1	-0.22	0.013189225	0.026070324
85377	4285	MICALL1	-0.22	0.018663562	0.035819201
9792	5442	SERTAD2	-0.22	0.017301544	0.033454547
26750	4026	RPS6KC1	-0.22	0.019262416	0.036833256
7531	1831	YWHAE	-0.22	0.000571576	0.001451238
5782	3168	PTPN12	-0.22	0.003346194	0.007410591
57102	3868	C12orf4	-0.22	0.024918547	0.046473469
55632	5465	G2E3	-0.22	0.026817751	0.049692127
80854	6466	SETD7	-0.22	0.003826118	0.008396007
399909	5500	PCNX3	-0.22	0.003137882	0.006990206
23272	8133	FAM208A	-0.22	0.002796825	0.006280043
54815	5464	GATAD2A	-0.22	0.002307789	0.005262422
990	3061	CDC6	-0.22	0.003270679	0.007262363
56984	1091	PSMG2	-0.22	0.007370776	0.015335358
10847	10491	SRCAP	-0.22	0.013810261	0.027222179
196527	6102	ANO6	-0.22	0.00189203	0.004385855
79269	7481	DCAF10	-0.22	0.006067067	0.012847056
1503	3009	CTPS1	-0.22	0.00412002	0.008985989
2079	801	ERH	-0.22	0.01436646	0.028224598
8731	4899	RNMT	-0.22	0.009435406	0.019225722
55631	2819	LRRC40	-0.22	0.012655202	0.025115338
4853	10827	NOTCH2	-0.22	0.001676868	0.003922922
57591	4208	MKL1	-0.22	0.014600536	0.028652786
2923	3060	PDIA3	-0.22	8.88E-05	0.00025404
5706	1690	PSMC6	-0.23	0.01691777	0.032808877
55773	3699	TBC1D23	-0.23	0.015867017	0.030916237
79719	3184	AAGAB	-0.23	0.026316646	0.04885531
8692	2143	HYAL2	-0.23	0.012009846	0.023925434
23523	6052	CABIN1	-0.23	0.026848149	0.049743266
9819	4761	TSC22D2	-0.23	0.0156134	0.030472199
51659	1196	GINS2	-0.23	0.015395265	0.03007951
79139	2866	DERL1	-0.23	0.000915619	0.00224706
60681	2867	FKBP10	-0.23	0.000326904	0.000861657
2271	1877	FH	-0.23	0.012618202	0.025044706
26509	6833	MYOF	-0.23	0.000342694	0.000900871

51605	2349	TRMT6	-0.23	0.022146552	0.041755087
7005	3023	TEAD3	-0.23	0.014598164	0.028651296
659	12086	BMPR2	-0.23	0.003288056	0.007293654
5373	2340	PMM2	-0.23	0.017891676	0.034494281
286077	5732	FAM83H	-0.23	0.00336766	0.007454414
26058	7839	GIGYF2	-0.23	0.005494178	0.011721969
8621	6906	CDK13	-0.23	0.005737821	0.012199307
317	5900	APAF1	-0.23	0.017522029	0.033840684
5496	2302	PPM1G	-0.23	0.000672855	0.001688599
55666	4370	NPLOC4	-0.23	0.000784891	0.001947215
387893	2761	KMT5A	-0.23	0.005111614	0.010970306
53	2153	ACP2	-0.23	0.013305299	0.026288064
9045	879	RPL14	-0.23	0.002687026	0.006059534
51729	2690	WBP11	-0.23	0.003466553	0.007660911
6605	2425	SMARCE1	-0.23	0.00059077	0.001497189
8804	2048	CREG1	-0.23	0.006374419	0.013437024
56257	2349	MEPCE	-0.23	0.007837984	0.016208717
2551	4859	GABPA	-0.23	0.004945126	0.010644528
214	4914	ALCAM	-0.23	0.000296201	0.00078539
8394	3687	PIP5K1A	-0.23	0.000344924	0.000906332
6654	7969	SOS1	-0.23	0.026928709	0.049866525
57136	2241	APMAP	-0.23	0.000554154	0.001410229
6627	1103	SNRPA1	-0.23	0.007447274	0.015480015
154007	4162	SNRNP48	-0.23	0.023468491	0.044027891
51479	7132	ANKFY1	-0.23	0.000954203	0.002335301
10971	2272	YWHAQ	-0.23	0.000322685	0.000851421
84376	14460	HOOK3	-0.23	0.008301639	0.017093931
6891	5598	TAP2	-0.23	0.002347544	0.005348271
55028	3611	C17orf80	-0.23	0.024510904	0.045775672
2934	2494	GSN	-0.23	0.00310214	0.006917523
54545	5145	MTMR12	-0.23	0.007649274	0.015864631
23265	4700	EXOC7	-0.23	0.001116477	0.002698987
1508	2756	CTSB	-0.23	0.00095305	0.0023328
57062	2967	DDX24	-0.23	0.000266197	0.000710818
6240	3012	RRM1	-0.23	0.001667779	0.0039032
9416	3288	DDX23	-0.23	0.005077023	0.010903969
25764	1350	HYPK	-0.23	0.020319229	0.038629581
22803	3460	XRN2	-0.23	0.000693647	0.001737099
9637	2016	FEZ2	-0.23	0.013777269	0.027166188
55210	2431	ATAD3A	-0.23	0.005386862	0.011516517
51125	1933	GOLGA7	-0.23	0.023816512	0.044600736
29941	3528	PKN3	-0.23	0.010379417	0.020966518
10576	1964	CCT2	-0.23	0.000541387	0.001378923
8812	2608	CCNK	-0.23	0.012928222	0.025602837
6500	2030	SKP1	-0.23	0.001250674	0.002995279
5718	4425	PSMD12	-0.23	0.001590005	0.003734955
55246	3651	CCDC25	-0.23	0.024616966	0.045959258
653121	7330	ZBTB8A	-0.23	0.019304281	0.036909337

3842	8515	TNPO1	-0.23	0.001712678	0.004000376
8260	1135	NAA10	-0.23	0.020706443	0.039281665
57532	10917	NUFIP2	-0.23	0.002553446	0.005782517
9883	6351	POM121	-0.23	0.026643488	0.049400135
2730	5070	GCLM	-0.23	0.006829133	0.014305592
55230	5079	USP40	-0.23	0.007046382	0.014715575
8315	4056	BRAP	-0.24	0.017072434	0.033058305
84612	4625	PARD6B	-0.24	0.015815714	0.030823034
25842	2525	ASF1A	-0.24	0.02402776	0.044930041
1977	3780	EIF4E	-0.24	0.007796933	0.016139465
4841	3110	NONO	-0.24	4.15E-05	0.000124467
6878	2480	TAF6	-0.24	0.011296168	0.022630546
26100	4438	WIPI2	-0.24	0.002949224	0.006598062
6764	3459	ST5	-0.24	0.0006172	0.001557287
9908	4256	G3BP2	-0.24	0.000434083	0.001123657
498	1928	ATP5F1A	-0.24	0.001015356	0.002472027
2889	6184	RAPGEF1	-0.24	0.00258782	0.005852901
6160	535	RPL31	-0.24	0.013784289	0.027177013
1994	6075	ELAVL1	-0.24	0.000781859	0.001939961
7936	1391	NELFE	-0.24	0.001240433	0.002973157
22870	3654	PPP6R1	-0.24	0.000640471	0.00161211
4040	9835	LRP6	-0.24	0.013144146	0.025992786
80347	2205	COASY	-0.24	0.010244346	0.020717226
79142	2033	PHF23	-0.24	0.008848998	0.018130649
221154	1947	MICU2	-0.24	0.003821904	0.008387795
23348	7461	DOCK9	-0.24	0.007919859	0.016366604
25870	2050	SUMF2	-0.24	0.00058588	0.001485856
9922	7154	IQSEC1	-0.24	0.021175152	0.040080974
8570	3816	KHSRP	-0.24	0.005357417	0.011464601
1460	1149	CSNK2B	-0.24	0.009937992	0.020148133
9134	2734	CCNE2	-0.24	0.00507993	0.010908895
23595	2584	ORC3	-0.24	0.005352571	0.011455611
11345	1031	GABARAPL2	-0.24	0.005364722	0.011474706
163	5723	AP2B1	-0.24	7.62E-05	0.000220114
81887	2504	LAS1L	-0.24	0.003033622	0.006775789
10657	2895	KHDRBS1	-0.24	5.64E-05	0.00016552
84916	2061	UTP4	-0.24	0.004721433	0.010205648
115024	1387	NT5C3B	-0.24	0.004375443	0.009507505
7374	2090	UNG	-0.24	0.003248253	0.007217077
11231	3610	SEC63	-0.24	0.000883034	0.002171892
23633	7386	KPNA6	-0.24	0.000195362	0.000532147
9500	2723	MAGED1	-0.24	5.36E-05	0.000158363
22887	5087	FOXJ3	-0.24	0.005299131	0.011354915
51669	2056	SARAF	-0.24	0.001176017	0.002831748
896	2049	CCND3	-0.24	0.002382272	0.005423912
3192	7085	HNRNPU	-0.24	5.07E-06	1.71E-05
10197	3454	PSME3	-0.24	0.001608105	0.00377398
131870	4251	NUDT16	-0.24	0.018195738	0.034981809

57448	15416	BIRC6	-0.24	0.002260649	0.005164209
3985	3581	LIMK2	-0.24	0.002623815	0.005926012
10000	6446	AKT3	-0.24	0.000959279	0.002345139
836	2556	CASP3	-0.24	0.00542519	0.011588696
51340	4048	CRNKL1	-0.24	0.016451736	0.03197489
151011	3608	SEPT10	-0.24	0.000321246	0.000847751
9475	7863	ROCK2	-0.24	0.002204459	0.00504493
5164	2411	PDK2	-0.25	0.008165252	0.016824793
9169	7618	SCAF11	-0.25	0.000833838	0.002057727
8886	3790	DDX18	-0.25	0.001113598	0.002692761
1073	3193	CFL2	-0.25	0.002537475	0.005748548
5162	1542	PDHB	-0.25	0.002532477	0.005737957
2050	4218	EPHB4	-0.25	0.007317078	0.01523077
10867	4311	TSPAN9	-0.25	0.001557297	0.003666365
90957	4838	DHX57	-0.25	0.018910656	0.036222999
7726	3323	TRIM26	-0.25	0.002047591	0.004715085
9730	7206	DCAF1	-0.25	0.006566051	0.013806578
6599	5598	SMARCC1	-0.25	7.06E-05	0.000204587
5786	3269	PTPRA	-0.25	0.000954601	0.002335953
10477	1500	UBE2E3	-0.25	0.001443532	0.003422561
11328	3176	FKBP9	-0.25	0.001088806	0.002638197
10613	3213	ERLIN1	-0.25	0.003433762	0.007591275
79657	3687	RPAP3	-0.25	0.009125319	0.018642963
3875	1483	KRT18	-0.25	0.003207809	0.007135247
1291	4246	COL6A1	-0.25	0.001263002	0.003021139
9612	8811	NCOR2	-0.25	0.002592362	0.005860935
79682	2554	CENPU	-0.25	0.005341009	0.011434999
26994	3082	RNF11	-0.25	0.001504692	0.00355429
998	2180	CDC42	-0.25	0.000610518	0.001541301
57589	6420	RIC1	-0.25	0.011014903	0.022131946
7158	9769	TP53BP1	-0.25	0.002707049	0.006100818
5048	5443	PAFAH1B1	-0.25	0.000300506	0.000796092
3925	1545	STMN1	-0.25	0.000511167	0.001307386
85360	3267	SYDE1	-0.25	0.006550738	0.013779272
5514	4579	PPP1R10	-0.25	0.000848055	0.00209165
4241	2948	MELTF	-0.25	9.68E-05	0.000275833
59342	1944	SCPEP1	-0.25	0.018832119	0.0361037
5725	3328	PTBP1	-0.25	2.97E-05	9.07E-05
3190	2928	HNRNPK	-0.25	9.70E-06	3.15E-05
91869	3404	RFT1	-0.25	0.021970275	0.041453525
79954	2690	NOL10	-0.25	0.011588136	0.023147612
25836	9284	NIPBL	-0.25	0.001247651	0.002988441
5297	5225	PI4KA	-0.25	0.000196183	0.000534138
9144	1711	SYNGR2	-0.25	0.004042692	0.008833072
57505	4756	AARS2	-0.25	0.022658029	0.042615418
23035	8336	PHLPP2	-0.25	0.025429163	0.04736115
91010	12428	FMNL3	-0.25	0.003805677	0.008355279
23765	8560	IL17RA	-0.25	0.009500295	0.019346847

65125	9967	WNK1	-0.25	0.000774402	0.001923608
126917	5916	IFFO2	-0.25	0.010178931	0.020596657
29128	3963	UHRF1	-0.25	0.000121752	0.000340944
7917	3669	BAG6	-0.25	0.000183962	0.000503098
55187	16285	VPS13D	-0.25	0.002124075	0.004877933
5689	938	PSMB1	-0.25	0.002884323	0.006463446
23568	2026	ARL2BP	-0.25	0.015597592	0.030448034
387	1941	RHOA	-0.25	0.000364991	0.000955105
9929	3542	JOSD1	-0.25	0.00260239	0.005882109
27044	3519	SND1	-0.25	0.000135649	0.000378012
10427	3798	SEC24B	-0.25	0.003204304	0.007129236
2677	3487	GGCX	-0.25	0.006050169	0.012812802
10486	3088	CAP2	-0.25	0.012859759	0.025492825
64434	6082	NOM1	-0.25	0.014167062	0.027851333
90324	3283	CCDC97	-0.25	0.017392367	0.033611876
84515	5954	MCM8	-0.25	0.002083616	0.004789351
2794	7411	GNL1	-0.25	0.000468033	0.001204696
200734	4202	SPRED2	-0.25	0.01094125	0.021996394
51118	1353	UTP11	-0.25	0.002412756	0.005483481
10480	1446	EIF3M	-0.25	0.000170365	0.000468048
7249	5450	TSC2	-0.25	0.003741638	0.008231973
8683	1204	SRSF9	-0.25	0.001331654	0.003173395
26515	2861	TIMM10B	-0.25	0.018089944	0.034814146
4240	2058	MFGE8	-0.25	0.000107583	0.000304238
5685	1222	PSMA4	-0.25	0.000198721	0.000540467
8662	3073	EIF3B	-0.25	0.00069578	0.00174195
8661	5305	EIF3A	-0.25	0.000130056	0.000362824
5119	2360	CHMP1A	-0.25	0.000957713	0.002342599
11321	1783	GPN1	-0.25	0.014041693	0.027632385
4904	3160	YBX1	-0.25	0.0007813	0.001939118
9373	4926	PLAA	-0.25	0.001825936	0.004243163
9939	4974	RBM8A	-0.25	0.000185015	0.000505531
23644	4811	EDC4	-0.25	0.000796079	0.001972765
9826	6199	ARHGEF11	-0.26	0.003405148	0.007530827
10102	2016	TSFM	-0.26	0.019130719	0.036620837
3654	3533	IRAK1	-0.26	0.002307026	0.005261359
113026	3486	PLCD3	-0.26	0.008491913	0.017449326
84255	3284	SLC37A3	-0.26	0.0152321	0.029780364
4836	4627	NMT1	-0.26	0.00015015	0.000416592
10933	1810	MORF4L1	-0.26	0.000215185	0.000581596
5189	4029	PEX1	-0.26	0.016315555	0.031727567
4430	4794	MYO1B	-0.26	7.90E-05	0.000227871
9874	4790	TLK1	-0.26	0.000926308	0.002271411
22882	4448	ZHX2	-0.26	0.009733788	0.019779862
9969	10142	MED13	-0.26	0.000450954	0.001164446
10056	2114	FARSB	-0.26	0.002399119	0.005455978
50	2744	ACO2	-0.26	0.000671749	0.001686153
3660	2302	IRF2	-0.26	0.018611262	0.035726544

57510	5378	XPO5	-0.26	0.001101823	0.002666104
84923	2854	FAM104A	-0.26	0.002490468	0.00564998
9727	3355	RAB11FIP3	-0.26	0.000856089	0.002109707
3300	2152	DNAJB2	-0.26	0.005621035	0.011973929
5223	1886	PGAM1	-0.26	0.002322805	0.005294625
54788	3465	DNAJB12	-0.26	0.000812511	0.002009283
8872	1441	CDC123	-0.26	0.001618495	0.00379636
204851	7430	HIPK1	-0.26	0.00107257	0.00260205
8925	15252	HERC1	-0.26	0.006870504	0.014380388
221937	11191	FOXK1	-0.26	0.000309495	0.000817955
65265	2617	C8orf33	-0.26	0.007732991	0.016027036
2146	2717	EZH2	-0.26	0.00091112	0.002236638
7329	1435	UBE2I	-0.26	0.00038528	0.001005678
6218	590	RPS17	-0.26	0.000258824	0.00069217
57647	4549	DHX37	-0.26	0.014103474	0.027741673
25844	1596	YIPF3	-0.26	0.00526987	0.011294939
8078	3141	USP5	-0.26	0.000868511	0.002137651
128239	5308	IQGAP3	-0.26	0.000907694	0.002228536
10771	4007	ZMYND11	-0.26	0.000452088	0.001167204
1616	2030	DAXX	-0.26	0.001244593	0.002981921
79691	3833	QTRT2	-0.26	0.017025243	0.032988496
79685	6243	SAP30L	-0.26	0.011731715	0.023405469
54801	5967	HAUS6	-0.26	0.001673983	0.003916687
8161	2649	COIL	-0.26	0.009892726	0.02006584
54926	4449	UBE2R2	-0.26	0.00085886	0.00211552
10269	3165	ZMPSTE24	-0.26	0.000826099	0.002040743
10492	4940	SYNCRIP	-0.26	2.45E-05	7.57E-05
5116	10515	PCNT	-0.26	0.000771542	0.001918115
359845	3895	RFLNB	-0.26	0.000874329	0.002151374
1455	2899	CSNK1G2	-0.26	0.000661243	0.001661335
5170	7550	PDPK1	-0.26	0.000788731	0.001955376
6493	4568	SIM2	-0.26	0.003627842	0.007998449
23223	4370	RRP12	-0.26	0.017895902	0.034498686
6122	1348	RPL3	-0.26	0.000414126	0.001075759
9813	5794	EFCAB14	-0.26	0.000283705	0.000754846
861	6534	RUNX1	-0.26	0.011614605	0.023195269
10726	1481	NUDC	-0.26	0.000482484	0.001238483
8985	2995	PLOD3	-0.26	0.001505192	0.003554997
55658	1663	RNF126	-0.26	0.003837276	0.008418412
1738	3612	DLD	-0.26	0.001462751	0.003463969
137964	3460	GPAT4	-0.26	0.001700992	0.003974648
10635	2231	RAD51AP1	-0.26	0.004976638	0.010706489
8273	2038	SLC10A3	-0.27	0.019198535	0.036728321
8883	1764	NAE1	-0.27	0.000599251	0.001516737
5598	2908	MAPK7	-0.27	0.011688084	0.023326141
1025	2476	CDK9	-0.27	0.000957977	0.002342922
5394	2827	EXOSC10	-0.27	0.000152738	0.000422916
55114	3526	ARHGAP17	-0.27	0.002232471	0.005104431

27154	4645	BRPF3	-0.27	0.003314687	0.007348143
5493	6255	PPL	-0.27	0.002067908	0.004756939
7290	4013	HIRA	-0.27	0.003162501	0.007038872
54539	1114	NDUFB11	-0.27	0.001420819	0.003371408
8314	3715	BAP1	-0.27	0.000260471	0.000695947
23774	3678	BRD1	-0.27	0.004670104	0.010108223
9202	6879	ZMYM4	-0.27	0.000493376	0.001264333
10025	2924	MED16	-0.27	0.00221132	0.005058676
253959	7277	RALGAPA1	-0.27	0.005395603	0.011532427
9107	4170	MTMR6	-0.27	0.00494544	0.010644528
9375	3515	TM9SF2	-0.27	3.30E-05	0.000100099
2947	4144	GSTM3	-0.27	0.011081554	0.022250768
79074	3500	C2orf49	-0.27	0.016397452	0.031872873
83879	2641	CDCA7	-0.27	0.003212517	0.007143676
56904	1905	SH3GLB2	-0.27	0.000918872	0.002253799
9898	3745	UBAP2L	-0.27	0.001234014	0.00295897
30836	2725	DNTTIP2	-0.27	0.000354378	0.000929249
1147	3533	CHUK	-0.27	0.010484198	0.021156527
253714	8232	MMS22L	-0.27	0.005013565	0.010776799
9711	6946	RUBCN	-0.27	0.009024202	0.018447081
2739	2071	GLO1	-0.27	0.000103451	0.000293536
84836	2213	ABHD14B	-0.27	0.007998013	0.016506366
23560	2537	GTPBP4	-0.27	0.001481998	0.00350581
197131	7758	UBR1	-0.27	0.006520977	0.013721544
7094	8434	TLN1	-0.27	4.54E-05	0.000135222
55633	3509	TBC1D22B	-0.27	0.014084109	0.027709716
55002	2997	TMCO3	-0.27	0.000406601	0.001057449
6942	7268	TCF20	-0.27	0.000649076	0.001632151
79876	3246	UBA5	-0.27	0.005859231	0.012435301
55095	4556	SAMD4B	-0.27	0.003698586	0.008142296
23259	4335	DDHD2	-0.27	0.005247374	0.011250796
10112	3471	KIF20A	-0.27	2.94E-05	8.97E-05
130916	2295	MTERF4	-0.27	0.010664338	0.021478572
79752	2105	ZFAND1	-0.27	0.012945424	0.025622623
292	1351	SLC25A5	-0.27	0.000358418	0.00093915
145864	1962	HAPLN3	-0.27	0.021658389	0.04092155
5984	1434	RFC4	-0.27	0.001488854	0.003520185
29079	2261	MED4	-0.27	0.000773234	0.001921514
51143	2517	DYNC1LI1	-0.27	0.003119679	0.006954889
22930	4844	RAB3GAP1	-0.27	0.000418014	0.001084907
54476	5771	RNF216	-0.27	0.000396377	0.001032825
51726	1698	DNAJB11	-0.27	7.49E-05	0.000216543
5998	3188	RGS3	-0.27	0.002198904	0.005033513
5793	8639	PTPRG	-0.27	0.002057926	0.004737043
441518	2038	RTL8B	-0.27	0.013887279	0.027364886
84687	3975	PPP1R9B	-0.27	0.003402236	0.007525324
23151	4348	GRAMD4	-0.27	0.012765458	0.025322837
11259	3308	FILIP1L	-0.27	0.006701501	0.014068085

84962	3727	AJUBA	-0.27	0.000231856	0.00062409
9924	5298	PAN2	-0.27	0.007041793	0.01470772
11196	4891	SEC23IP	-0.27	0.000596468	0.001510339
11218	3513	DDX20	-0.27	0.009053583	0.018500754
8312	3103	AXIN1	-0.27	0.007676132	0.015916619
23179	4853	RGL1	-0.27	0.025227333	0.047014816
79634	3658	SCRN3	-0.27	0.015726747	0.0306732
92703	2513	TMEM183A	-0.27	0.000518814	0.001325037
5709	2187	PSMD3	-0.27	0.000453426	0.00117032
25948	3412	KBTBD2	-0.27	0.000561722	0.001428101
84930	4197	MASTL	-0.27	0.001405888	0.003337763
6142	671	RPL18A	-0.27	0.001841135	0.004275707
2194	8481	FASN	-0.27	0.000287655	0.00076467
8506	5601	CNTNAP1	-0.27	0.009741842	0.019791109
79364	3830	ZXDC	-0.27	0.009762218	0.019827972
152559	3739	PAQR3	-0.27	0.00702379	0.014676718
57599	4031	WDR48	-0.27	0.001722403	0.004021505
64114	2388	TMBIM1	-0.27	0.000313294	0.000827379
2130	2320	EWSR1	-0.27	1.97E-06	6.91E-06
117178	5238	SSX2IP	-0.27	0.006453374	0.013587339
57479	6921	PRR12	-0.27	0.023746058	0.044473486
9712	4816	USP6NL	-0.27	0.010562729	0.021300484
26469	3317	PTPN18	-0.27	0.007007682	0.014648529
84272	1946	YIPF4	-0.27	0.007755719	0.016067824
9276	3277	COPB2	-0.27	5.42E-06	1.82E-05
3268	4352	AGFG2	-0.28	0.026458878	0.049088581
9968	6985	MED12	-0.28	0.00610262	0.012909414
4615	2710	MYD88	-0.28	0.011410408	0.02283624
22905	4356	EPN2	-0.28	0.008601435	0.017658035
375484	2841	SIMC1	-0.28	0.015773544	0.030747596
6950	2459	TCP1	-0.28	6.06E-05	0.000177308
7844	2634	RNF103	-0.28	0.003770761	0.008287839
8192	1194	CLPP	-0.28	0.002725277	0.006135673
80204	3925	FBXO11	-0.28	0.000350672	0.000919801
84747	4413	UNC119B	-0.28	0.001054949	0.00256245
1676	2511	DFFA	-0.28	0.001770497	0.004121881
10787	4550	NCKAP1	-0.28	3.30E-05	0.00010015
26985	5231	AP3M1	-0.28	0.000758142	0.001887177
11017	1452	SNRNP27	-0.28	0.003211979	0.007143627
3140	7726	MR1	-0.28	0.005828898	0.012378135
23070	4132	CMTR1	-0.28	0.000419427	0.001088098
9801	7827	MRPL19	-0.28	0.002860209	0.006415071
9972	5854	NUP153	-0.28	0.000135984	0.000378827
256302	4787	NATD1	-0.28	0.008332419	0.017153333
51449	5377	PCYOX1	-0.28	3.66E-05	0.000110282
10552	1623	ARPC1A	-0.28	0.000778421	0.001933051
25983	1163	NGDN	-0.28	0.019106903	0.036579189
55858	1995	TMEM165	-0.28	9.83E-05	0.000279713

90379	2274	DCAF15	-0.28	0.003455601	0.007637657
7733	4500	ZNF180	-0.28	0.017087426	0.033083729
8863	6171	PER3	-0.28	0.003308397	0.007335113
8533	1748	COPS3	-0.28	0.000204946	0.000556181
1478	2115	CSTF2	-0.28	0.019526824	0.037302722
23741	2096	EID1	-0.28	1.00E-05	3.25E-05
10001	2386	MED6	-0.28	0.006136626	0.012975792
1915	2557	EEF1A1	-0.28	4.50E-05	0.00013412
127253	3351	TYW3	-0.28	0.013344824	0.026354433
55122	1948	AKIRIN2	-0.28	0.004809702	0.010388863
10632	1343	ATP5MG	-0.28	0.001017324	0.002476479
51010	1821	EXOSC3	-0.28	0.013994091	0.027547863
6732	4380	SRPK1	-0.28	0.000182512	0.000499443
88455	3770	ANKRD13A	-0.28	0.00091713	0.002250148
6396	1385	SEC13	-0.28	0.000439421	0.001137143
3840	8981	KPNA4	-0.28	9.76E-05	0.000277811
54535	2642	CCHCR1	-0.28	0.001002449	0.002442946
6731	3884	SRP72	-0.28	8.29E-05	0.00023822
57786	6527	RBAK	-0.28	0.005339288	0.01143269
9031	6569	BAZ1B	-0.28	4.65E-06	1.57E-05
140739	2185	UBE2F	-0.28	0.017497084	0.033803218
58525	4478	WIZ	-0.28	0.000679977	0.001705028
5886	1815	RAD23A	-0.28	0.002044741	0.004710355
5980	11046	REV3L	-0.28	0.001256743	0.003007382
6207	529	RPS13	-0.28	0.000211136	0.000571347
25902	2943	MTHFD1L	-0.28	0.001670699	0.003909518
9128	2939	PRPF4	-0.28	0.005515274	0.011759917
6165	512	RPL35A	-0.28	0.001358948	0.003233667
9277	2340	WDR46	-0.28	0.002023723	0.00466496
7469	2483	NELFA	-0.28	0.008557461	0.01757182
283489	3820	CHAMP1	-0.28	0.001389141	0.003299766
79665	3599	DHX40	-0.28	0.000713616	0.001784594
79903	2674	NAA60	-0.28	0.000608874	0.001537589
3921	1186	RPSA	-0.28	0.000932102	0.002284672
55893	4807	ZNF395	-0.28	7.72E-05	0.000222885
8773	2637	SNAP23	-0.28	0.000876255	0.002155815
132660	5338	LIN54	-0.28	0.00235797	0.005370646
8826	7219	IQGAP1	-0.28	4.21E-06	1.43E-05
23380	6487	SRGAP2	-0.28	0.000619924	0.001563493
3030	3048	HADHA	-0.28	9.57E-06	3.11E-05
55323	1014	LARP6	-0.28	0.023734076	0.044455731
6945	2338	MLX	-0.28	0.00126792	0.00303127
92856	3085	IMP4	-0.28	0.002441035	0.005544205
112574	5164	SNX18	-0.28	0.012937634	0.025612912
55276	3240	PGM2	-0.29	0.005028979	0.010804705
4763	11680	NF1	-0.29	0.000423432	0.001098007
55830	2034	GLT8D1	-0.29	0.001771247	0.004123085
10262	2001	SF3B4	-0.29	0.005365957	0.011475966

254065	12666	BRWD3	-0.29	0.014977944	0.029325411
64755	2778	C16orf58	-0.29	0.002226732	0.005091965
55744	1485	COA1	-0.29	0.000750196	0.001869493
57703	3346	CWC22	-0.29	0.003331489	0.007383548
57606	5329	SLAIN2	-0.29	0.000496695	0.001272203
3691	5695	ITGB4	-0.29	0.000153802	0.000425596
51157	1289	ZNF580	-0.29	0.01794938	0.034585165
4801	3192	NFYB	-0.29	0.00100538	0.002449751
23353	3800	SUN1	-0.29	0.000198808	0.000540606
11130	1709	ZWINT	-0.29	6.53E-05	0.000190205
29928	1691	TIMM22	-0.29	0.019642682	0.0374918
10969	1401	EBNA1BP2	-0.29	0.002069526	0.004760045
10460	2746	TACC3	-0.29	7.54E-05	0.000218063
51061	3128	TXNDC11	-0.29	0.000962253	0.002351762
4116	772	MAGOH	-0.29	0.004083922	0.008914389
55182	2090	RNF220	-0.29	0.000344434	0.00090531
8449	2663	DHX16	-0.29	0.001424166	0.003377997
6049	3431	RNF6	-0.29	0.007130503	0.014873771
81614	3063	NIPA2	-0.29	0.000128919	0.00035971
9659	6240	PDE4DIP	-0.29	0.000773926	0.001922842
23196	5069	FAM120A	-0.29	3.19E-05	9.69E-05
402055	2395	SRRD	-0.29	0.010092505	0.020435741
10426	3860	TUBGCP3	-0.29	0.001492833	0.003528624
5929	4376	RBBP5	-0.29	0.003280988	0.007281614
79850	2255	FAM57A	-0.29	0.001245856	0.002984546
6478	2632	SIAH2	-0.29	0.02610795	0.048513498
211	2288	ALAS1	-0.29	0.006586762	0.013845213
8289	8278	ARID1A	-0.29	0.000995297	0.002427342
6428	3144	SRSF3	-0.29	8.86E-06	2.89E-05
1317	4797	SLC31A1	-0.29	0.001781215	0.004144118
80199	1678	FUZ	-0.29	0.006770129	0.014198731
91768	4433	CABLES1	-0.29	0.015647766	0.030529208
202052	5113	DNAJC18	-0.29	0.011119248	0.022318888
23333	4906	DPY19L1	-0.29	0.00025502	0.000682923
6455	2459	SH3GL1	-0.29	0.000493409	0.001264333
65005	1652	MRPL9	-0.29	0.000661194	0.001661335
284207	1636	METRNL	-0.29	0.010962666	0.022036737
30849	5078	PIK3R4	-0.29	0.00358935	0.007918497
4942	2170	OAT	-0.29	0.001550269	0.003650786
22826	1765	DNAJC8	-0.29	5.84E-06	1.95E-05
5527	2228	PPP2R5C	-0.29	8.45E-05	0.00024254
246175	8866	CNOT6L	-0.29	0.000670418	0.001683197
3326	2703	HSP90AB1	-0.29	1.23E-07	4.88E-07
10947	3868	AP3M2	-0.29	0.016727646	0.032468518
11200	1638	CHEK2	-0.29	0.009952951	0.020173858
22949	1300	PTGR1	-0.29	0.000425785	0.001103625
85021	2705	REPS1	-0.29	0.006656369	0.013984908
83732	2203	RIOK1	-0.29	0.014623251	0.028691024

205564	6299	SENP5	-0.29	0.003654199	0.008052562
56270	2624	WDR45B	-0.29	5.47E-05	0.000161289
7871	5119	SLMAP	-0.29	0.000181173	0.000496006
10285	2064	SMNDC1	-0.29	0.001409145	0.003345049
7089	2672	TLE2	-0.29	0.016154341	0.031431267
5339	12726	PLEC	-0.29	2.35E-05	7.29E-05
10188	4096	TNK2	-0.29	0.008679918	0.017808866
81562	2398	LMAN2L	-0.29	0.0068931	0.014419191
6309	6946	SC5D	-0.29	0.013051992	0.025824922
79701	4392	OGFOD3	-0.29	0.002140636	0.004910886
54809	6832	SAMD9	-0.29	0.005634594	0.011995622
5605	1751	MAP2K2	-0.29	0.0002585	0.00069141
23077	12937	MYCBP2	-0.29	0.002071468	0.004763893
5036	2643	PA2G4	-0.29	3.21E-05	9.76E-05
84295	4389	PHF6	-0.29	0.001430504	0.003392578
28987	1775	NOB1	-0.29	0.002128595	0.004886417
93624	4339	TADA2B	-0.29	0.007237525	0.015075773
9810	5527	RNF40	-0.30	0.000105217	0.000298164
5338	3425	PLD2	-0.30	0.005404198	0.011549409
80155	5529	NAA15	-0.30	4.92E-05	0.000146132
440275	5544	EIF2AK4	-0.30	0.000208053	0.000563848
60684	4422	TRAPPC11	-0.30	0.002611349	0.005899358
10043	2374	TOM1	-0.30	0.007213076	0.015031894
1031	1685	CDKN2C	-0.30	0.000117018	0.00032914
28981	2755	IFT81	-0.30	0.002379944	0.005419306
64132	3594	XYLT2	-0.30	0.000586489	0.001487189
9837	3289	GINS1	-0.30	0.002888579	0.00647135
2091	973	FBL	-0.30	0.001211057	0.002909419
10491	6668	CRTAP	-0.30	3.68E-06	1.25E-05
6602	3399	SMARCD1	-0.30	0.000648387	0.00163065
6502	3217	SKP2	-0.30	0.000952593	0.002332003
8754	4110	ADAM9	-0.30	3.84E-05	0.000115577
6390	1161	SDHB	-0.30	0.001435231	0.003403335
7003	9433	TEAD1	-0.30	4.40E-05	0.000131376
118980	2567	SFXN2	-0.30	0.010588643	0.021347896
26145	2589	IRF2BP1	-0.30	0.006235844	0.013169912
5422	5468	POLA1	-0.30	0.000148023	0.00041095
3678	4267	ITGA5	-0.30	5.10E-05	0.000150925
57587	4916	CFAP97	-0.30	0.00474576	0.010254489
23481	2335	PES1	-0.30	0.000404605	0.001052875
55299	1303	BRIX1	-0.30	0.000199782	0.000542939
2077	2695	ERF	-0.30	0.00060654	0.001532567
2674	9200	GFRA1	-0.30	1.86E-05	5.84E-05
23133	5690	PHF8	-0.30	0.015536973	0.030336367
9802	2158	DAZAP2	-0.30	0.000103621	0.000293971
10066	2479	SCAMP2	-0.30	0.000746994	0.001862558
219333	4413	USP12	-0.30	0.000214045	0.00057869
54531	2942	MIER2	-0.30	0.019158373	0.03666436

81034	3126	SLC25A32	-0.30	0.003386349	0.007492982
56261	5492	GPCPD1	-0.30	0.001821168	0.004233192
7739	3656	ZNF185	-0.30	0.000356019	0.000933276
55732	3201	C1orf112	-0.30	0.00742889	0.015447222
84061	4035	MAGT1	-0.30	4.49E-05	0.00013405
64927	3531	TTC23	-0.30	0.017874895	0.034465668
7798	8440	LUZP1	-0.30	7.93E-05	0.000228717
51154	2274	MRTO4	-0.30	0.000593681	0.001503709
56993	1396	TOMM22	-0.30	0.000128353	0.000358411
55308	3015	DDX19A	-0.30	0.000115658	0.000325573
92345	3740	NAF1	-0.30	0.009195262	0.018768687
2767	4145	GNA11	-0.30	0.000117388	0.000330077
322	2496	APBB1	-0.30	0.00259509	0.005866357
2245	4291	FGD1	-0.30	0.001728379	0.004034927
57089	8564	ENTPD7	-0.30	0.006196401	0.013092836
6300	1873	MAPK12	-0.30	0.010683831	0.021512955
4670	2492	HNRNPM	-0.30	5.95E-07	2.21E-06
10198	5270	MPHOSPH9	-0.30	0.00046517	0.001197846
3075	3942	CFH	-0.30	0.000436223	0.001129033
55898	3284	UNC45A	-0.30	0.000444706	0.001149649
4088	5845	SMAD3	-0.30	1.04E-05	3.38E-05
51202	1836	DDX47	-0.30	0.00040253	0.001047936
5934	4500	RBL2	-0.30	2.01E-05	6.28E-05
6908	1921	TBP	-0.30	0.014492273	0.028459061
84335	2014	AKT1S1	-0.30	0.002289238	0.005224814
23335	6202	WDR7	-0.30	0.006795799	0.014247523
57718	3592	PPP4R4	-0.30	0.022735585	0.042747712
7355	2018	SLC35A2	-0.30	0.009783281	0.01986167
580	4812	BARD1	-0.30	0.000857432	0.002112432
55508	3705	SLC35E3	-0.30	0.005213605	0.011181091
83786	3758	FRMD8	-0.30	0.000556979	0.001416605
51649	1498	MRPS23	-0.30	0.010358144	0.020928304
55193	7860	PBRM1	-0.30	3.13E-05	9.54E-05
81554	2425	RCC1L	-0.30	0.001174279	0.002828079
86	1885	ACTL6A	-0.30	0.000178555	0.00048914
55704	8637	CCDC88A	-0.30	1.72E-05	5.41E-05
1939	2051	EIF2D	-0.31	0.000530844	0.001354009
27042	4520	DIEXF	-0.31	0.007484419	0.015553587
23527	7187	ACAP2	-0.31	0.000695473	0.001741427
79894	3101	ZNF672	-0.31	0.006068513	0.012848587
54838	4272	WBP1L	-0.31	0.000296879	0.000786952
25978	2624	CHMP2B	-0.31	0.001388298	0.003298646
6890	2541	TAP1	-0.31	0.000535177	0.001364084
23294	5387	ANKS1A	-0.31	0.014222694	0.027957609
48	3531	ACO1	-0.31	2.51E-05	7.74E-05
11215	9822	AKAP11	-0.31	0.000307621	0.000813243
51231	2084	VRK3	-0.31	0.016087347	0.031307774
9753	8381	ZSCAN12	-0.31	0.008000062	0.016506366

26136	2785	TES	-0.31	0.000195438	0.000532273
55222	3025	LRRC20	-0.31	0.005080974	0.010909819
23594	1644	ORC6	-0.31	0.007522924	0.01562264
22916	2187	NCBP2	-0.31	4.94E-05	0.000146539
2737	8223	GLI3	-0.31	0.014845945	0.029082993
55379	2933	LRRC59	-0.31	8.94E-05	0.000255602
53635	1670	PTOV1	-0.31	0.000228554	0.000615762
10238	6394	DCAF7	-0.31	1.76E-05	5.54E-05
115416	774	MALSU1	-0.31	0.012064219	0.024025671
10950	1478	BTG3	-0.31	0.001576881	0.00370687
140609	4024	NEK7	-0.31	0.000204708	0.000555646
84790	1652	TUBA1C	-0.31	3.68E-05	0.000111054
9444	7186	QKI	-0.31	1.16E-05	3.75E-05
55343	3255	SLC35C1	-0.31	0.007956109	0.016431959
10607	2618	TBL3	-0.31	0.001946967	0.004499117
19	10331	ABCA1	-0.31	0.001036733	0.002519582
8636	954	SSNA1	-0.31	0.007217539	0.015039432
4690	4382	NCK1	-0.31	0.002652766	0.005987594
10869	4325	USP19	-0.31	0.000216409	0.000584726
10598	1422	AHSA1	-0.31	0.00012605	0.000352369
4809	1575	SNU13	-0.31	0.000491176	0.001258976
8888	6324	MCM3AP	-0.31	4.66E-05	0.000138618
55870	7765	ASH1L	-0.31	0.000154667	0.000427457
5073	3062	PARN	-0.31	0.000628463	0.001583456
84946	1835	LTV1	-0.31	0.000913418	0.00224197
51073	1651	MRPL4	-0.31	0.001496705	0.003537304
200576	9568	PIKFYVE	-0.31	0.001813729	0.004217555
7187	7826	TRAF3	-0.31	6.73E-05	0.000195506
84501	3364	SPIRE2	-0.31	0.016472104	0.032007474
9320	9616	TRIP12	-0.31	1.07E-06	3.87E-06
81608	3035	FIP1L1	-0.31	0.000184277	0.000503884
2632	3118	GBE1	-0.31	1.98E-05	6.19E-05
160418	6627	TMTC3	-0.31	0.000955398	0.00233726
58190	2507	CTDSP1	-0.31	0.0003683	0.000963338
79754	2736	ASB13	-0.31	0.018775633	0.036013411
3454	5613	IFNAR1	-0.31	6.68E-05	0.000194412
55135	1918	WRAP53	-0.31	0.008874558	0.018172542
192111	2776	PGAM5	-0.31	0.00132898	0.003167871
1802	2519	DPH2	-0.31	0.003834609	0.008413599
55683	5190	KANSL3	-0.31	3.23E-05	9.80E-05
5830	3251	PEX5	-0.31	0.007624776	0.015815668
3344	5368	FOXN2	-0.31	0.025564134	0.047572636
9221	3947	NOLC1	-0.31	4.18E-06	1.42E-05
9836	2860	LCMT2	-0.31	0.00958237	0.019496113
51094	2119	ADIPOR1	-0.31	6.71E-05	0.000195233
829	2711	CAPZA1	-0.31	7.54E-06	2.49E-05
11011	5278	TLK2	-0.31	0.000244029	0.000655069
114049	1257	BUD23	-0.31	0.000771787	0.001918455

23531	2726	MMD	-0.31	0.0020028	0.004622127
55626	5062	AMBRA1	-0.31	0.000556441	0.001415438
56945	1167	MRPS22	-0.31	0.000770322	0.001915349
7014	2993	TERF2	-0.31	0.003688988	0.008125194
23587	1669	ELP5	-0.31	0.002237717	0.00511511
51564	4136	HDAC7	-0.31	2.19E-05	6.82E-05
56941	1657	HMCES	-0.31	0.000272708	0.000727004
11230	1316	PRAF2	-0.31	0.002662488	0.006007248
84273	2286	NOA1	-0.32	0.001449049	0.003433808
79902	2024	NUP85	-0.32	8.41E-05	0.000241551
131601	1872	TPRA1	-0.32	0.000444202	0.001148681
5607	2344	MAP2K5	-0.32	0.021883575	0.04129871
11159	2125	RABL2A	-0.32	0.022536549	0.042404893
201292	3325	TRIM65	-0.32	0.00432831	0.009415452
3020	1077	H3F3A	-0.32	3.97E-07	1.50E-06
142678	3410	MIB2	-0.32	0.000518219	0.001323707
57700	5828	FAM160B1	-0.32	0.004429855	0.009621031
51622	1834	CCZ1	-0.32	0.000139423	0.000388044
14	1842	AAMP	-0.32	0.00017516	0.000480359
3576	1718	CXCL8	-0.32	0.012191065	0.024256533
26073	2753	POLDIP2	-0.32	4.82E-05	0.00014316
780	3791	DDR1	-0.32	1.29E-05	4.13E-05
64151	3991	NCAPG	-0.32	1.30E-05	4.17E-05
84065	1629	TMEM222	-0.32	0.001135856	0.002742477
10295	2168	BCKDK	-0.32	0.001256028	0.003006481
29940	4367	DSE	-0.32	0.000119794	0.000336098
8744	1680	TNFSF9	-0.32	0.011101908	0.022286601
7247	3406	TSN	-0.32	1.36E-05	4.33E-05
79798	3938	ARMC5	-0.32	0.010537894	0.021255227
2931	2200	GSK3A	-0.32	0.004341063	0.009437418
7873	993	MANF	-0.32	0.000118445	0.00033268
360023	8263	ZBTB41	-0.32	0.021556009	0.040745443
3301	2405	DNAJA1	-0.32	2.90E-06	1.00E-05
10308	3293	ZNF267	-0.32	0.026423577	0.049038449
56654	1548	NPDC1	-0.32	0.002163781	0.004959503
56938	6750	ARNTL2	-0.32	0.001308282	0.003121886
79171	1703	RBM42	-0.32	0.000351957	0.000923037
4905	3995	NSF	-0.32	4.45E-05	0.000132863
57799	2599	RAB40C	-0.32	0.00579538	0.012317262
23012	4767	STK38L	-0.32	0.005914085	0.01254105
201725	3545	C4orf46	-0.32	0.002131858	0.004892643
27445	16317	PCLO	-0.32	0.020428455	0.038820619
7342	3873	UBP1	-0.32	3.68E-05	0.00011105
55432	6265	YOD1	-0.32	0.006818348	0.014286369
1981	5342	EIF4G1	-0.32	6.72E-08	2.74E-07
79018	3913	GID4	-0.32	0.015783665	0.030763948
79648	4793	MCPH1	-0.32	0.010450099	0.021092509
5298	3732	PI4KB	-0.32	8.26E-05	0.000237299

6117	4345	RPA1	-0.32	4.28E-05	0.000127919
27175	2154	TUBG2	-0.32	0.010922258	0.021963182
51132	10599	RLIM	-0.32	1.72E-05	5.42E-05
157769	5510	FAM91A1	-0.32	0.000124853	0.000349298
126299	1411	ZNF428	-0.32	0.001866245	0.004330039
55279	5267	ZNF654	-0.32	0.002142872	0.00491348
54529	2484	ASNSD1	-0.32	0.000341428	0.000897809
158	1784	ADSL	-0.32	0.000283246	0.000753738
22950	2982	SLC4A1AP	-0.32	0.00016665	0.000458862
54946	2214	SLC41A3	-0.32	0.002385942	0.00543018
56946	6729	EMSY	-0.32	0.001158772	0.002792873
129642	7308	MBOAT2	-0.32	0.000108271	0.000306136
8666	1127	EIF3G	-0.32	2.53E-05	7.80E-05
84993	1416	UBL7	-0.32	0.004299205	0.009353285
162427	3729	RETREG3	-0.32	0.00018605	0.000508105
55791	2224	LRIF1	-0.32	0.006435966	0.013557112
10484	4123	SEC23A	-0.32	1.11E-05	3.58E-05
1020	1206	CDK5	-0.32	0.012658952	0.025119975
1876	3256	E2F6	-0.32	0.003648586	0.008042188
10054	2671	UBA2	-0.32	3.83E-05	0.000115289
10078	1906	TSSC4	-0.32	0.005358725	0.01146602
808	2277	CALM3	-0.32	0.000111799	0.000315659
5427	1738	POLE2	-0.32	0.005703013	0.012131106
4719	3916	NDUFS1	-0.32	1.63E-05	5.14E-05
54977	2137	SLC25A38	-0.32	0.005439874	0.011617269
10980	1441	COPS6	-0.32	1.80E-06	6.32E-06
25853	3688	DCAF12	-0.32	6.88E-05	0.000199776
92305	2494	TMEM129	-0.32	0.000121178	0.000339497
7317	3843	UBA1	-0.33	2.19E-06	7.66E-06
201514	2371	ZNF584	-0.33	0.019546732	0.037328712
90639	4896	COX19	-0.33	0.012120592	0.024129823
10097	3929	ACTR2	-0.33	7.87E-08	3.18E-07
92140	6547	MTDH	-0.33	5.58E-07	2.08E-06
687	5208	KLF9	-0.33	2.00E-05	6.26E-05
2932	7098	GSK3B	-0.33	1.69E-05	5.34E-05
54386	2196	TERF2IP	-0.33	0.000433982	0.00112356
166968	5216	MIER3	-0.33	0.000679025	0.001702881
57534	9070	MIB1	-0.33	0.000114035	0.00032136
3632	3086	INPP5A	-0.33	0.011992756	0.023894067
8540	7706	AGPS	-0.33	3.77E-06	1.28E-05
64431	2503	ACTR6	-0.33	0.005695849	0.012117319
5018	1737	OXA1L	-0.33	0.000109442	0.0003092
220	3416	ALDH1A3	-0.33	0.001938669	0.004483378
10038	1998	PARP2	-0.33	0.000575015	0.001459344
81565	2422	NDEL1	-0.33	0.002552123	0.005780259
166647	4071	ADGRA3	-0.33	2.67E-05	8.22E-05
29098	1042	RANGRF	-0.33	0.011457032	0.022916643
4520	7888	MTF1	-0.33	0.002436598	0.005534833

10573	1148	MRPL28	-0.33	5.98E-05	0.000175133
6194	829	RPS6	-0.33	2.46E-09	1.14E-08
10678	2758	B3GNT2	-0.33	0.002045829	0.00471225
1841	1217	DTYMK	-0.33	0.002681251	0.00604728
9736	11326	USP34	-0.33	3.42E-06	1.17E-05
51184	1506	GPN3	-0.33	0.011278886	0.02260357
1973	1893	EIF4A1	-0.33	7.81E-08	3.16E-07
10776	5434	ARPP19	-0.33	9.72E-07	3.52E-06
11176	8799	BAZ2A	-0.33	3.58E-06	1.22E-05
10518	1566	CIB2	-0.33	0.009894709	0.02006584
54455	6145	FBXO42	-0.33	0.000829433	0.002047711
2961	1417	GTF2E2	-0.33	0.005452643	0.01164174
7057	5952	THBS1	-0.33	5.51E-06	1.85E-05
128077	3986	LIX1L	-0.33	0.000749338	0.001867878
80019	1711	UBTD1	-0.33	0.003799549	0.008342855
9101	4748	USP8	-0.33	4.23E-05	0.000126515
51667	3137	NUB1	-0.33	0.000270778	0.000722383
23233	5366	EXOC6B	-0.33	0.00154162	0.0036309
26502	1800	NARF	-0.33	0.006438887	0.013561659
1854	1581	DUT	-0.33	3.24E-05	9.83E-05
6464	3423	SHC1	-0.33	9.52E-08	3.82E-07
55588	3663	MED29	-0.33	2.60E-05	8.01E-05
79134	5938	TMEM185B	-0.33	2.12E-05	6.59E-05
26470	3347	SEZ6L2	-0.33	2.52E-05	7.77E-05
7283	1968	TUBG1	-0.33	0.00035454	0.000929535
9790	3730	BMS1	-0.33	1.10E-05	3.55E-05
79809	5585	TTC21B	-0.33	0.012483885	0.024794732
25807	1943	RHBDD3	-0.33	0.00320619	0.007132539
22818	1945	COPZ1	-0.33	1.66E-06	5.86E-06
168451	3303	THAP5	-0.33	0.001845337	0.004284886
26000	3254	TBC1D10B	-0.33	0.000751198	0.001871202
55422	4648	ZNF331	-0.33	0.004043905	0.008834637
129138	2164	ANKRD54	-0.33	0.003075358	0.006861247
3839	4210	KPNA3	-0.33	7.87E-05	0.000226889
116225	1419	ZMYND19	-0.33	7.58E-05	0.000219149
7702	2979	ZNF143	-0.33	0.00514324	0.01103418
22864	3877	R3HDM2	-0.33	0.001083513	0.002626447
160	3459	AP2A1	-0.33	5.29E-07	1.97E-06
55147	2554	RBM23	-0.33	0.000114674	0.000323008
85406	3330	DNAJC14	-0.33	8.45E-05	0.00024252
9776	4403	ATG13	-0.33	8.16E-06	2.68E-05
22976	3822	PAXIP1	-0.33	0.000253881	0.000680078
84135	5095	UTP15	-0.33	0.001568918	0.003691279
663	6353	BNIP2	-0.33	1.23E-05	3.94E-05
5289	3595	PIK3C3	-0.33	0.000230744	0.000621284
54780	1420	NSMCE4A	-0.33	0.000476402	0.001223931
2110	2142	ETFDH	-0.34	0.013969111	0.027504784
91368	1296	CDKN2AIPNL	-0.34	0.001224205	0.002937737

9831	3972	ZNF623	-0.34	0.013463416	0.026579776
182	5988	JAG1	-0.34	0.018583721	0.035677532
254394	4080	MCM9	-0.34	0.004677254	0.010121234
3068	2168	HDGF	-0.34	5.18E-07	1.93E-06
1639	4381	DCTN1	-0.34	2.87E-06	9.88E-06
84548	2588	TMEM185A	-0.34	0.005346586	0.011445559
5894	3070	RAF1	-0.34	2.23E-06	7.76E-06
6193	755	RPS5	-0.34	6.60E-06	2.19E-05
79039	4402	DDX54	-0.34	2.42E-05	7.48E-05
1052	1269	CEBPD	-0.34	8.52E-05	0.000244407
55127	8497	HEATR1	-0.34	8.32E-05	0.000238867
200081	4819	TXLNA	-0.34	1.84E-06	6.46E-06
79637	1846	ARMC7	-0.34	0.007996813	0.016506366
56926	3746	NCLN	-0.34	2.34E-05	7.25E-05
23139	5272	MAST2	-0.34	2.69E-05	8.26E-05
81688	2585	C6orf62	-0.34	2.82E-07	1.08E-06
79680	6693	RTL10	-0.34	0.003694827	0.008136038
3028	962	HSD17B10	-0.34	0.00012146	0.000340179
9545	4290	RAB3D	-0.34	0.001183061	0.00284678
6201	745	RPS7	-0.34	2.53E-06	8.77E-06
548645	2268	DNAJC25	-0.34	0.01431073	0.028124439
23107	2797	MRPS27	-0.34	7.58E-06	2.50E-05
1534	2983	CYB561	-0.34	0.00401484	0.008777614
55361	4204	PI4K2A	-0.34	8.90E-05	0.000254615
114884	3307	OSBPL10	-0.34	3.72E-05	0.000112193
2907	1896	GRINA	-0.34	2.70E-06	9.31E-06
23469	6874	PHF3	-0.34	2.15E-06	7.50E-06
79603	1758	CERS4	-0.34	0.005636247	0.011997703
1314	5639	COPA	-0.34	1.99E-08	8.52E-08
7701	6525	ZNF142	-0.34	0.002756235	0.00619595
84002	4178	B3GNT5	-0.34	0.000116354	0.000327427
9401	3740	RECQL4	-0.34	8.19E-05	0.000235609
339175	2474	METTL2A	-0.34	0.003075103	0.006861247
221184	2252	CPNE2	-0.34	0.000393633	0.001026277
56474	3934	CTPS2	-0.34	0.001909265	0.004420619
64855	3887	FAM129B	-0.34	1.54E-05	4.87E-05
60672	1617	MIIP	-0.34	0.011225123	0.02250852
2976	3961	GTF3C2	-0.34	2.30E-05	7.13E-05
9512	2831	PMPCB	-0.34	5.87E-06	1.96E-05
5351	3047	PLOD1	-0.34	1.48E-06	5.27E-06
781	7344	CACNA2D1	-0.34	0.022191842	0.041827162
9716	5106	AQR	-0.34	2.00E-05	6.24E-05
5994	2825	RFXAP	-0.34	0.018052889	0.034759832
23351	6164	KHNYN	-0.34	9.70E-05	0.000276401
1454	2746	CSNK1E	-0.34	0.00014406	0.000400447
133	1606	ADM	-0.34	7.12E-05	0.000206316
2936	3174	GSR	-0.34	3.63E-05	0.000109462
79834	10256	PEAK1	-0.34	0.000152241	0.000421737

11332	1557	ACOT7	-0.34	0.00010554	0.000298748
80895	1436	ILKAP	-0.34	0.006044878	0.012803123
5702	1618	PSMC3	-0.34	7.65E-06	2.52E-05
60678	2148	EEFSEC	-0.34	0.005933781	0.012573886
10920	2277	COPS8	-0.34	7.47E-05	0.000216218
79137	4283	RETREG2	-0.34	4.49E-06	1.52E-05
6196	5709	RPS6KA2	-0.34	0.000833969	0.002057765
26037	7187	SIPA1L1	-0.34	2.57E-05	7.92E-05
23219	5447	FBXO28	-0.34	0.000163889	0.000451679
131566	5925	DCBLD2	-0.34	3.37E-10	1.66E-09
11340	1415	EXOSC8	-0.34	7.01E-05	0.000203461
58509	3559	CACTIN	-0.34	0.001024866	0.002493472
85465	8143	SELENOI	-0.34	0.000527117	0.001344891
219771	3696	CCNY	-0.34	6.12E-06	2.04E-05
9862	3559	MED24	-0.34	1.93E-05	6.04E-05
51684	4817	SUFU	-0.34	0.002940221	0.00657875
10438	1233	C1D	-0.34	0.009473614	0.019294724
9150	3346	CTDP1	-0.34	0.000710421	0.001777104
78994	2076	PRR14	-0.34	0.002011527	0.004639857
55177	2281	RMDN3	-0.34	0.000484989	0.001244193
4668	3445	NAGA	-0.34	0.000137941	0.000384218
3476	1683	IGBP1	-0.34	0.000111587	0.00031516
6935	5866	ZEB1	-0.34	0.000120471	0.00033773
215	3697	ABCD1	-0.35	0.00793782	0.016399906
11021	2890	RAB35	-0.35	7.90E-05	0.000227772
25926	2638	NOL11	-0.35	7.00E-05	0.000203112
65977	3832	PLEKHA3	-0.35	0.006784244	0.014224976
2771	2256	GNAI2	-0.35	9.47E-05	0.000270009
81689	2012	ISCA1	-0.35	0.000304843	0.000806541
10495	3750	ENOX2	-0.35	0.015058027	0.029462728
27097	3408	TAF5L	-0.35	0.000508178	0.001300115
55798	2192	METTL2B	-0.35	0.013952118	0.02747437
5504	3468	PPP1R2	-0.35	6.60E-05	0.000192123
130074	5288	FAM168B	-0.35	2.29E-07	8.84E-07
55355	2661	HJURP	-0.35	2.17E-05	6.73E-05
9618	2844	TRAF4	-0.35	3.01E-05	9.19E-05
29117	2497	BRD7	-0.35	1.37E-06	4.88E-06
51011	1514	FAHD2A	-0.35	0.00112677	0.002721648
57707	4380	TLDC1	-0.35	0.001097791	0.002657608
7804	6619	LRP8	-0.35	0.002281115	0.005208951
54682	2327	MANSC1	-0.35	0.006741804	0.014140996
3717	4855	JAK2	-0.35	0.011689327	0.023326141
11261	3163	CHP1	-0.35	1.09E-05	3.53E-05
56052	2513	ALG1	-0.35	0.001555052	0.003661565
8427	3698	ZNF282	-0.35	0.000519998	0.001327679
79180	2424	EFHD2	-0.35	0.000210182	0.000568938
85476	3461	GFM1	-0.35	3.11E-05	9.48E-05
5576	5769	PRKAR2A	-0.35	3.82E-06	1.30E-05

153769	3867	SH3RF2	-0.35	0.0024185	0.005495832
7461	5458	CLIP2	-0.35	0.000565096	0.001436015
84678	4013	KDM2B	-0.35	5.35E-05	0.000158049
137392	1980	FAM92A	-0.35	0.002520337	0.005713364
9330	4232	GTF3C3	-0.35	0.000196803	0.000535578
83874	2020	TBC1D10A	-0.35	0.016130764	0.03138883
4436	3235	MSH2	-0.35	2.44E-07	9.40E-07
29970	1694	SCHIP1	-0.35	0.001534167	0.003614972
91056	6595	AP5B1	-0.35	0.000732153	0.001827091
2801	4262	GOLGA2	-0.35	1.36E-06	4.84E-06
10728	2046	PTGES3	-0.35	2.11E-07	8.17E-07
23381	4566	SMG5	-0.35	1.27E-06	4.54E-06
6509	4470	SLC1A4	-0.35	1.78E-05	5.60E-05
54700	3552	RRN3	-0.35	6.39E-05	0.000186494
64328	8074	XPO4	-0.35	0.000118443	0.00033268
257364	3250	SNX33	-0.35	0.003336068	0.007392773
6767	3597	ST13	-0.35	3.04E-08	1.28E-07
9525	3396	VPS4B	-0.35	1.66E-05	5.25E-05
55643	2211	BTBD2	-0.35	0.000133935	0.000373353
9564	3295	BCAR1	-0.35	0.000223621	0.000603387
6498	7187	SKIL	-0.35	1.59E-05	5.04E-05
56910	3136	STARD7	-0.35	5.01E-06	1.69E-05
7477	3809	WNT7B	-0.35	0.000941779	0.002307436
10527	6209	IPO7	-0.35	4.43E-08	1.84E-07
27295	2712	PDLIM3	-0.35	0.011454616	0.02291439
25904	2601	CNOT10	-0.35	0.000165908	0.000457031
6872	7869	TAF1	-0.35	0.000221123	0.0005971
22995	5322	CEP152	-0.35	0.004184225	0.009115413
4247	2728	MGAT2	-0.35	0.000115398	0.000324893
57649	3911	PHF12	-0.35	0.000997947	0.002432641
9325	2063	TRIP4	-0.35	0.001376398	0.003271685
196513	2154	DCP1B	-0.35	0.026454441	0.049085476
89796	11682	NAV1	-0.35	2.86E-05	8.75E-05
27069	2449	GHITM	-0.35	2.37E-06	8.23E-06
116843	2432	SLC18B1	-0.35	0.001865066	0.004327869
6873	4908	TAF2	-0.35	1.19E-05	3.82E-05
7704	7381	ZBTB16	-0.35	0.001525919	0.003598658
56848	2930	SPHK2	-0.35	0.004691918	0.010146786
649	3490	BMP1	-0.35	3.92E-07	1.48E-06
728577	6379	CNTNAP3B	-0.35	0.011360573	0.02274675
9733	4017	SART3	-0.35	5.30E-06	1.78E-05
51308	2207	REEP2	-0.35	0.001196436	0.002876627
3831	2528	KLC1	-0.35	1.18E-05	3.81E-05
91663	3126	MYADM	-0.35	1.03E-06	3.71E-06
51283	3039	BFAR	-0.35	9.26E-06	3.02E-05
607	6088	BCL9	-0.35	0.001421073	0.00337156
115752	3780	DIS3L	-0.35	0.000858924	0.00211552
121536	4059	AEBP2	-0.36	2.36E-05	7.29E-05

5978	7184	REST	-0.36	4.43E-05	0.000132319
3607	5262	FOXK2	-0.36	4.78E-06	1.61E-05
1725	1328	DHPS	-0.36	0.000105405	0.000298555
23264	5918	ZC3H7B	-0.36	1.51E-07	5.92E-07
79684	3436	MSANTD2	-0.36	0.00592806	0.012567685
6709	7850	SPTAN1	-0.36	4.57E-08	1.89E-07
23160	3542	WDR43	-0.36	2.56E-05	7.88E-05
5546	2134	PRCC	-0.36	2.35E-05	7.27E-05
57139	2554	RGL3	-0.36	0.00501551	0.010779676
64771	4387	C6orf106	-0.36	7.24E-07	2.65E-06
4850	3425	CNOT4	-0.36	0.000763041	0.001898308
4882	5267	NPR2	-0.36	7.29E-06	2.41E-05
56889	4349	TM9SF3	-0.36	9.05E-09	4.00E-08
51117	1581	COQ4	-0.36	0.000282483	0.000751822
139341	1170	FUNDC1	-0.36	0.001887286	0.004377143
6794	3075	STK11	-0.36	3.61E-05	0.000109025
54708	3279	MARCH5	-0.36	0.000128102	0.000357768
221710	4710	SMIM13	-0.36	0.001913432	0.004429689
57120	4616	GOPC	-0.36	1.57E-05	4.98E-05
64324	12330	NSD1	-0.36	3.68E-06	1.26E-05
142	4001	PARP1	-0.36	3.19E-06	1.10E-05
587	1562	BCAT2	-0.36	2.43E-05	7.52E-05
10970	3036	CKAP4	-0.36	1.88E-08	8.05E-08
57551	11959	TAOK1	-0.36	1.11E-05	3.58E-05
83607	4541	AMMECR1L	-0.36	0.000121279	0.000339726
6385	2606	SDC4	-0.36	1.30E-07	5.15E-07
91147	4144	TMEM67	-0.36	0.009875415	0.020034981
1820	4961	ARID3A	-0.36	0.000601287	0.001521022
8539	3918	API5	-0.36	2.74E-07	1.05E-06
55081	3223	IFT57	-0.36	0.003597396	0.007935261
84126	2579	ATRIP	-0.36	0.010396135	0.020990742
11128	6652	POLR3A	-0.36	0.000427056	0.001106757
115708	3257	TRMT61A	-0.36	0.002074144	0.004768196
6730	2782	SRP68	-0.36	1.39E-05	4.43E-05
6856	2300	SYPL1	-0.36	3.01E-06	1.03E-05
222229	2285	LRWD1	-0.36	0.002848584	0.006391418
80218	6172	NAA50	-0.36	5.00E-07	1.87E-06
4289	11190	MKLN1	-0.36	7.86E-05	0.000226626
25814	3340	ATXN10	-0.36	1.78E-07	6.98E-07
85403	4499	EAF1	-0.36	0.000414595	0.001076819
689	1028	BTF3	-0.36	8.87E-08	3.57E-07
10168	5399	ZNF197	-0.36	0.000545349	0.001388418
131544	10525	CRYBG3	-0.36	0.002690789	0.006067252
6867	6608	TACC1	-0.36	4.40E-06	1.49E-05
65056	3515	GPBP1	-0.36	2.86E-07	1.09E-06
9188	4714	DDX21	-0.36	4.22E-07	1.59E-06
29110	3098	TBK1	-0.36	0.000113246	0.000319389
10055	2067	SAE1	-0.36	2.94E-06	1.01E-05

6613	1065	SUMO2	-0.36	6.19E-07	2.29E-06
1893	2158	ECM1	-0.36	4.42E-05	0.000132138
10048	2669	RANBP9	-0.37	1.74E-06	6.15E-06
27107	5229	ZBTB11	-0.37	6.60E-05	0.000192229
3199	1791	HOXA2	-0.37	0.007667783	0.015901163
2584	1361	GALK1	-0.37	0.002004175	0.004624097
54976	1314	C20orf27	-0.37	0.001299207	0.00310072
8607	1789	RUVBL1	-0.37	0.000730027	0.001822042
7088	3534	TLE1	-0.37	1.97E-05	6.17E-05
1954	11156	MEGF8	-0.37	8.26E-07	3.01E-06
201595	3942	STT3B	-0.37	3.53E-10	1.74E-09
57504	2931	MTA3	-0.37	7.75E-06	2.55E-05
7289	3082	TULP3	-0.37	7.62E-05	0.000220114
124402	1428	UBALD1	-0.37	0.017041563	0.033005722
1385	7487	CREB1	-0.37	4.44E-05	0.000132471
10801	3505	SEPT9	-0.37	1.39E-06	4.95E-06
5740	5603	PTGIS	-0.37	0.003336926	0.007393752
79596	3624	RNF219	-0.37	0.000639202	0.001609144
7913	2826	DEK	-0.37	8.02E-08	3.24E-07
116113	4240	FOXP4	-0.37	0.001743901	0.004066355
26224	3554	FBXL3	-0.37	8.72E-06	2.85E-05
55069	4238	TMEM248	-0.37	5.11E-08	2.11E-07
51639	783	SF3B6	-0.37	4.08E-05	0.000122452
7023	2147	TFAP4	-0.37	0.011755387	0.023447431
1633	2618	DCK	-0.37	2.52E-05	7.77E-05
79867	2885	TCTN2	-0.37	0.001345268	0.00320283
388272	1410	C16orf87	-0.37	0.009291493	0.018952043
1979	7531	EIF4EBP2	-0.37	9.47E-07	3.43E-06
3845	5739	KRAS	-0.37	2.65E-05	8.14E-05
129685	4413	TAF8	-0.37	0.000225598	0.000608075
64844	3523	MARCH7	-0.37	7.32E-08	2.97E-07
5905	3100	RANGAP1	-0.37	4.64E-06	1.56E-05
84912	6816	SLC35B4	-0.37	0.000469074	0.001207024
79664	4349	ICE2	-0.37	6.59E-05	0.000192102
254263	1356	CNIH2	-0.37	0.008754297	0.017946966
55707	2037	NECAP2	-0.37	0.000177311	0.000486033
9200	1323	HACD1	-0.37	0.008074856	0.016652048
25	5652	ABL1	-0.37	6.52E-08	2.66E-07
3416	5382	IDE	-0.37	1.48E-05	4.71E-05
145173	4166	B3GLCT	-0.37	0.000624632	0.00157425
23102	6070	TBC1D2B	-0.37	0.000103399	0.000293434
29072	8457	SETD2	-0.37	1.31E-06	4.67E-06
5412	4355	UBL3	-0.37	0.000181898	0.000497837
2005	10685	ELK4	-0.37	7.08E-06	2.34E-05
7262	937	PHLDA2	-0.37	0.013517023	0.026676715
23288	6504	IQCE	-0.37	0.000605064	0.001529923
65986	9089	ZBTB10	-0.37	0.000476671	0.001224445
5819	2403	NECTIN2	-0.37	3.33E-08	1.40E-07

64779	2797	MTHFSD	-0.37	0.003277711	0.007276159
10600	2935	USP16	-0.37	1.95E-05	6.11E-05
10042	4137	HMGXB4	-0.37	2.59E-06	8.97E-06
8819	1126	SAP30	-0.37	0.006939163	0.01451042
11198	4684	SUPT16H	-0.37	1.11E-10	5.72E-10
144715	3886	RAD9B	-0.37	0.004217198	0.009183867
8408	5239	ULK1	-0.37	0.000159432	0.000439941
7862	4635	BRPF1	-0.37	5.58E-05	0.000164117
84284	926	NTPCR	-0.37	0.007797229	0.016139465
404093	3018	CUEDC1	-0.37	9.24E-06	3.01E-05
140612	5724	ZFP28	-0.38	0.020128288	0.038304635
284098	2350	PIGW	-0.38	0.001133193	0.002736419
3655	5359	ITGA6	-0.38	7.33E-05	0.000212264
84955	3965	NUDCD1	-0.38	0.000408464	0.001062138
80851	3202	SH3BP5L	-0.38	0.0004122	0.00107107
50808	4238	AK3	-0.38	2.78E-06	9.60E-06
54956	2664	PARP16	-0.38	0.008085735	0.016672547
83719	1016	YPEL3	-0.38	0.002063315	0.004748216
11266	1299	DUSP12	-0.38	0.002894885	0.00648466
375444	2939	C5orf34	-0.38	0.013134333	0.025976273
9351	2119	SLC9A3R2	-0.38	0.000855198	0.002107805
6189	953	RPS3A	-0.38	2.00E-05	6.25E-05
55832	6010	CAND1	-0.38	2.51E-08	1.07E-07
8884	3042	SLC5A6	-0.38	0.000502731	0.001287292
6415	893	SELENOW	-0.38	0.000721284	0.001801994
54867	2800	TMEM214	-0.38	8.51E-07	3.10E-06
84919	5256	PPP1R15B	-0.38	6.17E-06	2.06E-05
85364	2773	ZCCHC3	-0.38	0.000409477	0.00106446
26015	4704	RPAP1	-0.38	0.00173301	0.004045209
51439	4762	FAM8A1	-0.38	3.92E-05	0.000117844
10052	7559	GJC1	-0.38	2.32E-07	8.95E-07
5435	2099	POLR2F	-0.38	0.000399612	0.001040796
6210	492	RPS15A	-0.38	0.000105034	0.000297741
64785	2226	GINS3	-0.38	0.000167004	0.000459693
65263	2678	PYCR3	-0.38	0.005037586	0.010821887
64979	633	MRPL36	-0.38	0.000718845	0.001796658
59274	3068	TLNRD1	-0.38	0.000871522	0.002144766
3735	2032	KARS	-0.38	7.25E-08	2.94E-07
84897	5345	TBRG1	-0.38	0.000458453	0.001182264
90678	3257	LRSAM1	-0.38	0.000158531	0.000437591
10419	2412	PRMT5	-0.38	9.65E-06	3.14E-05
5970	2588	RELA	-0.38	4.26E-05	0.000127547
10329	1983	RXYLT1	-0.38	0.010003574	0.020267218
4849	2934	CNOT3	-0.38	0.000482255	0.001238074
54505	4491	DHX29	-0.38	0.000132142	0.000368529
132320	2702	SCLT1	-0.38	0.001488867	0.003520185
2475	8392	MTOR	-0.38	3.74E-08	1.56E-07
10921	2222	RNPS1	-0.38	8.92E-08	3.59E-07

26953	3847	RANBP6	-0.38	3.10E-05	9.46E-05
54929	1812	TMEM161A	-0.38	0.00059347	0.00150339
23132	9982	RAD54L2	-0.38	1.50E-05	4.77E-05
5144	8400	PDE4D	-0.38	0.001031158	0.002506806
23318	5699	ZCCHC11	-0.38	3.99E-06	1.36E-05
4976	6327	OPA1	-0.38	2.00E-07	7.78E-07
81624	4430	DIAPH3	-0.38	7.05E-07	2.59E-06
116224	3874	FAM122A	-0.38	0.006254787	0.013200504
56623	3409	INPP5E	-0.38	0.000533941	0.001361128
79066	5763	METTL16	-0.38	1.57E-05	4.98E-05
1839	2381	HBEGF	-0.38	0.020973456	0.039728824
642	2543	BLMH	-0.38	1.91E-05	5.99E-05
10946	2871	SF3A3	-0.38	2.73E-08	1.16E-07
1523	6555	CUX1	-0.38	3.35E-07	1.27E-06
144455	5742	E2F7	-0.38	3.37E-05	0.000101959
253635	3877	GPATCH11	-0.38	0.000519597	0.001326848
7184	2879	HSP90B1	-0.38	3.48E-11	1.86E-10
8664	1949	EIF3D	-0.38	2.70E-08	1.14E-07
81567	3231	TXNDC5	-0.38	7.80E-08	3.16E-07
3021	2909	H3F3B	-0.38	1.73E-10	8.75E-10
11102	3497	RPP14	-0.38	2.91E-05	8.88E-05
80304	3897	WDCP	-0.38	0.016670114	0.03236392
25988	2277	HINFP	-0.38	0.016323399	0.031739347
3551	3997	IKBKB	-0.38	0.000167105	0.000459901
23283	4127	CSTF2T	-0.38	3.40E-06	1.16E-05
27153	3074	ZNF777	-0.38	0.002956843	0.006612611
4681	2101	NBL1	-0.38	0.000166061	0.000457383
165055	2564	CCDC138	-0.38	0.01582319	0.030834223
1390	1850	CREM	-0.38	0.004076522	0.008900424
5611	5557	DNAJC3	-0.38	3.85E-07	1.46E-06
4363	6221	ABCC1	-0.38	3.29E-08	1.39E-07
10208	4164	USPL1	-0.39	0.000619737	0.001563464
9513	3016	FXR2	-0.39	3.25E-05	9.87E-05
54465	3298	ETAA1	-0.39	0.000663811	0.001667079
9927	4596	MFN2	-0.39	4.66E-08	1.93E-07
9374	1991	PPT2	-0.39	0.003988478	0.008723201
26986	2872	PABPC1	-0.39	6.67E-10	3.21E-09
51318	1807	MRPL35	-0.39	0.000128665	0.000359115
678	3702	ZFP36L2	-0.39	1.79E-05	5.63E-05
6202	705	RPS8	-0.39	2.70E-07	1.04E-06
144983	2249	HNRNPA1L2	-0.39	0.00091861	0.002253467
9125	3321	CNOT9	-0.39	4.83E-07	1.81E-06
8050	2521	PDHX	-0.39	7.95E-05	0.000229146
221120	1585	ALKBH3	-0.39	0.00418023	0.009108944
84641	3021	MFSD14B	-0.39	5.24E-07	1.95E-06
84230	7140	LRRC8C	-0.39	0.000118317	0.000332426
10459	1104	MAD2L2	-0.39	0.000118005	0.000331652
54897	6158	CASZ1	-0.39	0.012612233	0.025035655

55622	2926	TTC27	-0.39	0.001901151	0.004404701
5316	5191	PKNOX1	-0.39	2.29E-05	7.10E-05
7520	3448	XRCC5	-0.39	4.02E-13	2.43E-12
8554	5723	PIAS1	-0.39	1.31E-05	4.20E-05
80135	1975	RPF1	-0.39	0.000305481	0.000808066
54764	3417	ZRANB1	-0.39	1.39E-06	4.93E-06
6938	4712	TCF12	-0.39	2.42E-05	7.50E-05
7728	3842	ZNF175	-0.39	0.007172224	0.014951976
2806	2488	GOT2	-0.39	1.86E-07	7.26E-07
7268	2395	TTC4	-0.39	0.000568304	0.00144355
8727	2569	CTNNAL1	-0.39	1.17E-07	4.64E-07
146540	3235	ZNF785	-0.39	0.010312175	0.020849651
6483	4450	ST3GAL2	-0.39	2.22E-05	6.90E-05
5236	2480	PGM1	-0.39	1.02E-07	4.10E-07
55133	3700	SRBD1	-0.39	0.000781849	0.001939961
2000	3906	ELF4	-0.39	6.70E-05	0.000195017
23172	3008	ABRAXAS2	-0.39	0.000207692	0.000563056
55721	2053	IQCC	-0.39	0.022842296	0.042934721
84172	5047	POLR1B	-0.39	0.000733196	0.001829182
23450	9736	SF3B3	-0.39	5.69E-10	2.76E-09
87178	4567	PNPT1	-0.39	4.99E-06	1.68E-05
54947	5100	LPCAT2	-0.39	0.001635298	0.003832736
23598	3402	PATZ1	-0.39	1.25E-05	4.02E-05
23328	7232	SASH1	-0.39	1.29E-06	4.61E-06
51493	2081	RTCB	-0.39	5.10E-05	0.000150928
1026	2177	CDKN1A	-0.39	2.35E-05	7.26E-05
2235	3528	FECH	-0.39	0.000117662	0.000330794
3608	1934	ILF2	-0.39	2.12E-07	8.23E-07
57592	4630	ZNF687	-0.39	7.01E-05	0.000203413
29777	2264	ABT1	-0.39	0.000583879	0.001481206
8527	6104	DGKD	-0.39	0.000483553	0.001240689
55322	3396	C5orf22	-0.39	1.92E-05	6.02E-05
10466	4948	COG5	-0.39	0.000115243	0.000324505
56980	6176	PRDM10	-0.39	0.004703849	0.010171351
64863	3615	METTL4	-0.39	0.004813677	0.010396184
8667	1286	EIF3H	-0.39	4.08E-08	1.70E-07
9039	2126	UBA3	-0.39	2.24E-06	7.81E-06
91782	2791	CHMP7	-0.39	0.000948928	0.002323992
10452	1733	TOMM40	-0.39	1.08E-06	3.89E-06
9341	2212	VAMP3	-0.40	4.45E-07	1.67E-06
81691	2663	REXO5	-0.40	0.002406688	0.005471089
6741	1725	SSB	-0.40	2.27E-08	9.70E-08
6650	4958	CAPN15	-0.40	6.63E-06	2.20E-05
84243	3165	ZDHHC18	-0.40	8.27E-05	0.000237691
55090	2222	MED9	-0.40	0.000387545	0.001011295
23039	4880	XPO7	-0.40	3.04E-06	1.04E-05
220988	4930	HNRNPA3	-0.40	1.70E-12	9.90E-12
65059	9166	RAPH1	-0.40	1.19E-06	4.27E-06

57343	4420	ZNF304	-0.40	0.003297643	0.007314009
81831	3327	NETO2	-0.40	4.08E-08	1.70E-07
162681	5609	C18orf54	-0.40	0.000321016	0.000847268
64645	2701	MFSD14A	-0.40	8.84E-06	2.89E-05
5931	2020	RBBP7	-0.40	1.60E-08	6.90E-08
7064	2642	THOP1	-0.40	1.13E-05	3.65E-05
2958	1586	GTF2A2	-0.40	3.37E-05	0.000102114
8545	4428	CGGBP1	-0.40	6.81E-09	3.04E-08
285704	4255	RGMB	-0.40	0.000411912	0.001070476
57466	4161	SCAF4	-0.40	1.65E-05	5.23E-05
26043	10125	UBXN7	-0.40	1.28E-07	5.07E-07
5925	4593	RB1	-0.40	1.78E-09	8.30E-09
53615	2431	MBD3	-0.40	1.77E-07	6.92E-07
10585	2976	POMT1	-0.40	3.23E-05	9.81E-05
26048	3866	ZNF500	-0.40	0.008872122	0.018169647
11014	2873	KDELR2	-0.40	5.76E-11	3.04E-10
116138	2029	KLHDC3	-0.40	7.45E-07	2.73E-06
266727	6380	MDGA1	-0.40	0.018621545	0.035742423
124944	903	C17orf49	-0.40	3.99E-05	0.000119656
79871	2911	RPAP2	-0.40	0.015697488	0.030619493
79647	2726	AKIRIN1	-0.40	1.51E-07	5.92E-07
9563	8663	H6PD	-0.40	1.17E-07	4.66E-07
7756	2321	ZNF207	-0.40	1.31E-09	6.18E-09
162989	1975	DEDD2	-0.40	0.002252773	0.005148864
64943	1735	NT5DC2	-0.40	1.12E-06	4.03E-06
83448	4114	PUS7L	-0.40	0.011350963	0.022730071
6670	6061	SP3	-0.40	4.63E-09	2.09E-08
23646	2136	PLD3	-0.40	5.22E-06	1.75E-05
10199	2488	MPHOSPH10	-0.40	1.58E-05	5.00E-05
140901	6489	STK35	-0.40	3.16E-05	9.61E-05
708	1163	C1QBP	-0.40	4.59E-06	1.55E-05
6801	14201	STRN	-0.40	4.32E-07	1.63E-06
9861	1478	PSMD6	-0.40	8.40E-07	3.06E-06
162073	7722	ITPRIPL2	-0.40	2.49E-06	8.62E-06
84342	2522	COG8	-0.40	0.000360861	0.000945132
253430	6130	IPMK	-0.40	0.001172632	0.002824871
10914	4493	PAPOLA	-0.40	2.28E-10	1.14E-09
9126	4131	SMC3	-0.40	2.80E-10	1.39E-09
7982	2323	ST7	-0.40	0.00061248	0.001545816
439	1298	ASNA1	-0.40	5.96E-07	2.21E-06
9066	5021	SYT7	-0.40	0.007912721	0.016353755
6487	2215	ST3GAL3	-0.40	0.001293994	0.00308945
23521	1195	RPL13A	-0.40	1.11E-07	4.41E-07
55038	2435	CDCA4	-0.40	6.38E-06	2.12E-05
11331	1456	PHB2	-0.40	1.03E-07	4.14E-07
3646	1516	EIF3E	-0.40	4.31E-10	2.10E-09
4076	4195	CAPRIN1	-0.40	1.36E-09	6.40E-09
7514	4246	XPO1	-0.40	1.24E-09	5.85E-09

23592	4798	LEMD3	-0.40	0.000113124	0.000319097
10153	3341	CEBPZ	-0.40	6.09E-08	2.49E-07
5914	3163	RARA	-0.41	1.33E-05	4.23E-05
55425	2576	GPALPP1	-0.41	2.48E-05	7.65E-05
1387	10149	CREBBP	-0.41	5.08E-07	1.90E-06
56160	1748	NSMCE3	-0.41	0.000469359	0.001207409
7112	3947	TMPO	-0.41	2.31E-12	1.33E-11
221496	2563	LEMD2	-0.41	8.41E-06	2.75E-05
6159	737	RPL29	-0.41	2.86E-06	9.84E-06
64795	6201	RMND5A	-0.41	4.08E-06	1.39E-05
54870	3280	QRICH1	-0.41	2.18E-08	9.31E-08
8100	2706	IFT88	-0.41	0.001395861	0.003315286
11133	1742	KPTN	-0.41	0.001782736	0.004147114
51710	2777	ZNF44	-0.41	0.017221257	0.033324689
11100	3650	HNRNPUL1	-0.41	5.80E-08	2.38E-07
1968	3486	EIF2S3	-0.41	1.09E-11	6.03E-11
10174	2072	SORBS3	-0.41	6.17E-05	0.000180417
10248	949	POP7	-0.41	8.48E-05	0.000243396
84277	2534	DNAJC30	-0.41	0.002057034	0.004735603
55226	4039	NAT10	-0.41	2.14E-06	7.49E-06
7314	1223	UBB	-0.41	3.57E-08	1.50E-07
114790	3592	STK11IP	-0.41	0.00050522	0.001292734
199692	2759	ZNF627	-0.41	0.002331479	0.005313715
55789	2510	DEPDC1B	-0.41	8.67E-05	0.000248482
79943	4686	ZNF696	-0.41	0.01550398	0.030278602
56902	1994	PNO1	-0.41	0.003886391	0.008515649
56975	3392	FAM20C	-0.41	1.54E-07	6.04E-07
127002	2589	ATXN7L2	-0.41	0.02609464	0.048493837
55075	5464	UACA	-0.41	3.25E-07	1.24E-06
5534	3111	PPP3R1	-0.41	2.90E-07	1.11E-06
285282	3874	RABL3	-0.41	9.76E-05	0.000277848
9466	2685	IL27RA	-0.41	0.001938894	0.004483378
56897	2657	WRNIP1	-0.41	1.37E-06	4.88E-06
27440	1865	HDHD5	-0.41	0.000752752	0.00187481
55709	2439	KBTBD4	-0.41	0.000671785	0.001686153
23517	4222	MTREX	-0.41	6.37E-09	2.84E-08
113251	6419	LARP4	-0.41	6.89E-06	2.28E-05
9915	6576	ARNT2	-0.41	0.000787376	0.001952933
54344	432	DPM3	-0.41	0.001606743	0.003771284
8520	1705	HAT1	-0.41	1.36E-08	5.90E-08
27297	2767	CRCP	-0.41	3.71E-06	1.26E-05
155061	3869	ZNF746	-0.41	0.000362768	0.000949987
22808	4146	MRAS	-0.41	0.000100319	0.000285332
6925	6953	TCF4	-0.41	0.000325691	0.000858782
1723	2890	DHODH	-0.41	0.011037602	0.022170034
66036	6922	MTMR9	-0.41	0.001152338	0.002780006
2967	3424	GTF2H3	-0.41	5.60E-05	0.000164636
8239	10480	USP9X	-0.41	3.13E-11	1.68E-10

114904	3472	C1QTNF6	-0.41	0.000433338	0.001122057
196051	1716	PLPP4	-0.41	0.001755094	0.004088699
9857	13478	CEP350	-0.41	4.73E-06	1.59E-05
56675	3809	NRIP3	-0.41	0.000106867	0.000302358
55680	4135	RUFY2	-0.41	0.000151801	0.000420714
85315	4758	PAQR8	-0.41	0.000477212	0.001225658
51193	2623	ZNF639	-0.41	3.01E-05	9.19E-05
83932	3503	SPRTN	-0.41	0.000892366	0.002193023
5198	5325	PFAS	-0.41	5.10E-07	1.90E-06
1604	2348	CD55	-0.41	0.000100377	0.000285452
2820	5398	GPD2	-0.41	2.31E-06	8.05E-06
55841	6463	WWC3	-0.41	1.23E-05	3.94E-05
10322	2703	SMYD5	-0.41	0.000132035	0.000368288
55170	2665	PRMT6	-0.41	3.50E-05	0.000105863
6232	361	RPS27	-0.41	1.56E-05	4.95E-05
29775	3912	CARD10	-0.41	2.24E-06	7.79E-06
29088	1736	MRPL15	-0.42	0.000474769	0.001220442
5902	1172	RANBP1	-0.42	5.53E-08	2.27E-07
4291	1826	MLF1	-0.42	0.000895464	0.00219942
2734	9325	GLG1	-0.42	1.53E-08	6.61E-08
3619	6026	INCENP	-0.42	5.07E-08	2.09E-07
883	1955	KYAT1	-0.42	0.000885064	0.002176248
10314	4567	LANCL1	-0.42	9.16E-08	3.68E-07
79724	2302	ZNF768	-0.42	2.39E-06	8.29E-06
50515	5762	CHST11	-0.42	0.000151223	0.000419373
84650	976	EBPL	-0.42	0.000240289	0.000645517
9688	2775	NUP93	-0.42	1.09E-07	4.36E-07
55852	5192	TEX2	-0.42	1.85E-05	5.81E-05
23270	4144	TSPYL4	-0.42	2.46E-06	8.55E-06
10765	6396	KDM5B	-0.42	9.90E-09	4.36E-08
7525	4676	YES1	-0.42	5.10E-10	2.48E-09
8576	2416	STK16	-0.42	0.001859243	0.004315483
8501	2218	SLC43A1	-0.42	0.014669709	0.028775821
6187	962	RPS2	-0.42	1.40E-10	7.15E-10
4800	6181	NFYA	-0.42	2.06E-06	7.20E-06
8751	2838	ADAM15	-0.42	5.04E-08	2.08E-07
6397	5208	SEC14L1	-0.42	3.70E-08	1.55E-07
84166	5440	NLRC5	-0.42	4.37E-05	0.000130705
7411	1652	VBP1	-0.42	1.51E-06	5.36E-06
26173	6991	INTS1	-0.42	1.24E-09	5.85E-09
1762	3308	DMWD	-0.42	2.05E-05	6.38E-05
23279	4815	NUP160	-0.42	3.91E-07	1.48E-06
4331	1397	MNAT1	-0.42	4.49E-05	0.000133892
1431	2997	CS	-0.42	6.85E-10	3.30E-09
10478	2294	SLC25A17	-0.42	5.07E-05	0.000150205
202243	3041	CCDC125	-0.42	0.001718157	0.00401212
399665	3955	FAM102A	-0.42	1.49E-05	4.73E-05
26747	3487	NUFIP1	-0.42	0.000526162	0.001342646

95681	4609	CEP41	-0.42	0.000621368	0.001566912
11138	4453	TBC1D8	-0.42	2.62E-05	8.06E-05
84878	2318	ZBTB45	-0.42	0.005449425	0.011636267
4294	3042	MAP3K10	-0.42	0.001698479	0.003969298
4691	2732	NCL	-0.42	1.46E-14	9.86E-14
29960	1605	MRM2	-0.42	2.57E-05	7.92E-05
3843	5908	IPO5	-0.42	6.82E-12	3.80E-11
10298	2671	PAK4	-0.42	9.16E-06	2.98E-05
8720	4334	MBTPS1	-0.42	4.92E-11	2.61E-10
161882	3172	ZFPM1	-0.42	0.002511522	0.005695562
3145	1519	HMBS	-0.42	0.001939173	0.004483439
5001	1967	ORC5	-0.42	0.000462209	0.001191085
10253	2325	SPRY2	-0.42	0.009231461	0.018836082
9794	5725	MAML1	-0.42	5.32E-07	1.98E-06
7586	9121	ZKSCAN1	-0.42	1.40E-08	6.08E-08
23300	4852	ATMIN	-0.42	3.91E-07	1.48E-06
116988	2806	AGAP3	-0.42	2.42E-06	8.39E-06
84231	3681	TRAF7	-0.42	2.10E-08	9.00E-08
1917	2022	EEF1A2	-0.42	3.78E-05	0.00011386
50862	4084	RNF141	-0.43	3.47E-05	0.000104996
9818	4337	NUP58	-0.43	1.37E-08	5.95E-08
284129	2821	SLC26A11	-0.43	0.000259024	0.000692602
79774	1781	GRTP1	-0.43	0.00887941	0.018180381
79068	4413	FTO	-0.43	4.23E-08	1.76E-07
6632	1634	SNRPD1	-0.43	3.55E-08	1.49E-07
54617	5443	INO80	-0.43	6.05E-06	2.02E-05
9567	5016	GTPBP1	-0.43	1.41E-06	5.00E-06
116115	2938	ZNF526	-0.43	0.022071609	0.041631459
5784	13443	PTPN14	-0.43	4.27E-11	2.27E-10
64175	2334	P3H1	-0.43	3.59E-07	1.36E-06
10280	1716	SIGMAR1	-0.43	6.77E-08	2.76E-07
3134	1648	HLA-F	-0.43	0.000294483	0.000781165
84749	3745	USP30	-0.43	0.003846093	0.008435672
54861	4982	SNRK	-0.43	8.13E-06	2.67E-05
63925	4321	ZNF335	-0.43	1.20E-05	3.86E-05
79797	2380	ZNF408	-0.43	0.005325601	0.011406133
57182	8034	ANKRD50	-0.43	3.41E-05	0.000103362
80148	2475	PQLC1	-0.43	3.45E-05	0.000104298
5878	1703	RAB5C	-0.43	4.04E-07	1.53E-06
256356	9462	GK5	-0.43	0.000780385	0.001937386
5329	1563	PLAUR	-0.43	8.04E-05	0.000231331
51246	2069	SHISA5	-0.43	3.68E-09	1.67E-08
84058	1116	WDR54	-0.43	0.000172565	0.000473753
54805	3676	CNNM2	-0.43	0.003371033	0.007460951
64282	4361	PAPD5	-0.43	0.000486594	0.001247769
9690	5130	UBE3C	-0.43	6.07E-10	2.93E-09
84312	2604	BRMS1L	-0.43	0.001141096	0.002753257
80311	6433	KLHL15	-0.43	0.000471796	0.001213152

5550	3011	PREP	-0.43	2.52E-05	7.77E-05
51373	600	MRPS17	-0.43	0.000788718	0.001955376
57019	2173	CIAPIN1	-0.43	3.95E-05	0.000118716
7866	2129	IFRD2	-0.43	7.83E-06	2.58E-05
51728	834	POLR3K	-0.43	0.00298816	0.006679283
90956	2380	ADCK2	-0.43	0.00057237	0.001453045
23350	7072	U2SURP	-0.43	1.12E-10	5.78E-10
23210	2260	JMJD6	-0.43	0.000139634	0.000388508
197322	2872	ACSF3	-0.43	0.000291698	0.000774605
22794	4070	CASC3	-0.43	1.09E-07	4.36E-07
55501	1848	CHST12	-0.43	0.000567949	0.001442853
10574	1925	CCT7	-0.43	1.85E-12	1.07E-11
4215	4649	MAP3K3	-0.43	4.56E-06	1.54E-05
771	4118	CA12	-0.43	0.000240432	0.000645803
26024	5505	PTCD1	-0.43	0.000395163	0.001029963
10849	3286	CD3EAP	-0.43	0.000824497	0.002037506
54925	2614	ZSCAN32	-0.43	0.002384914	0.005428536
8467	7926	SMARCA5	-0.43	2.91E-12	1.66E-11
79607	3046	FAM118B	-0.43	0.002761548	0.006207108
26286	2763	ARFGAP3	-0.43	9.63E-09	4.24E-08
6668	3222	SP2	-0.43	0.000149396	0.000414696
91300	1829	R3HDM4	-0.43	2.29E-05	7.10E-05
4814	1298	NINJ1	-0.43	4.44E-05	0.000132471
51343	3617	FZR1	-0.43	7.81E-07	2.85E-06
56252	6143	YLPM1	-0.43	3.51E-09	1.60E-08
85440	6822	DOCK7	-0.43	1.30E-05	4.17E-05
1.08E+08	1532	LOC107987020	-0.43	0.009994892	0.020256557
1512	1516	CTSH	-0.43	3.11E-07	1.19E-06
2643	2431	GCH1	-0.43	0.001290394	0.003082511
9249	1503	DHRS3	-0.43	2.66E-10	1.32E-09
5423	1306	POLB	-0.43	0.013996283	0.027549125
59343	3151	SENP2	-0.43	1.21E-06	4.35E-06
7042	5884	TGFB2	-0.43	3.63E-10	1.79E-09
9880	6256	ZBTB39	-0.43	0.002552016	0.005780259
3221	1726	HOXC4	-0.43	0.00315709	0.007027709
158586	5651	ZXDB	-0.43	0.011241004	0.022537819
25966	5841	C2CD2	-0.44	0.003044886	0.006799238
374654	4575	KIF7	-0.44	0.000567675	0.001442363
388552	2619	BLOC1S3	-0.44	0.007789761	0.016127765
23383	4713	MAU2	-0.44	7.87E-07	2.87E-06
55295	3122	KLHL26	-0.44	0.011762525	0.023459035
8295	12608	TRRAP	-0.44	1.44E-07	5.68E-07
93973	3742	ACTR8	-0.44	2.19E-05	6.80E-05
414	4859	ARSD	-0.44	1.31E-05	4.20E-05
10229	2667	COQ7	-0.44	6.12E-05	0.000179019
11120	2981	BTN2A1	-0.44	8.37E-05	0.000240198
132720	2730	FAM241A	-0.44	0.002142028	0.004912178
9805	5243	SCRN1	-0.44	1.47E-11	8.01E-11

57496	8959	MKL2	-0.44	1.25E-05	4.00E-05
51514	4607	DTL	-0.44	1.47E-08	6.39E-08
79968	4031	WDR76	-0.44	6.15E-08	2.52E-07
9318	4109	COPS2	-0.44	2.78E-09	1.28E-08
79145	1932	CHCHD7	-0.44	0.001921287	0.004445557
7625	3729	ZNF74	-0.44	0.017626707	0.034027742
10163	5446	WASF2	-0.44	9.48E-12	5.25E-11
665	3505	BNIP3L	-0.44	4.93E-08	2.03E-07
55183	10479	RIF1	-0.44	1.21E-09	5.73E-09
9784	2160	SNX17	-0.44	6.29E-06	2.09E-05
51594	5909	NBAS	-0.44	1.72E-06	6.07E-06
5557	1471	PRIM1	-0.44	9.92E-06	3.22E-05
80305	2297	TRABD	-0.44	2.91E-07	1.11E-06
23239	6411	PHLPP1	-0.44	3.34E-05	0.000101131
9343	4169	EFTUD2	-0.44	3.55E-09	1.62E-08
80071	3827	CCDC15	-0.44	0.020600725	0.039097808
54453	4188	RIN2	-0.44	4.66E-06	1.57E-05
51645	1750	PPIL1	-0.44	3.83E-06	1.30E-05
613	6927	BCR	-0.44	2.21E-07	8.55E-07
84950	3062	PRPF38A	-0.44	1.21E-08	5.28E-08
284403	3417	WDR62	-0.44	0.000102662	0.00029153
84294	3693	UTP23	-0.44	0.0001126	0.00031777
200895	3952	DHFR2	-0.44	0.00424091	0.009234373
55892	5353	MYNN	-0.44	4.10E-05	0.000122995
9710	5783	KIAA0355	-0.44	6.27E-06	2.09E-05
3783	2272	KCNN4	-0.44	0.00389604	0.008534685
5163	3874	PDK1	-0.44	4.74E-08	1.96E-07
8824	3822	CES2	-0.44	4.00E-10	1.96E-09
84792	2229	FAM220A	-0.44	0.000811919	0.002008098
6787	4311	NEK4	-0.44	7.14E-06	2.36E-05
4736	726	RPL10A	-0.44	2.81E-06	9.67E-06
29101	1319	SSU72	-0.44	3.02E-08	1.28E-07
60370	1455	AVPI1	-0.44	0.007907723	0.016345326
1653	2755	DDX1	-0.44	4.49E-09	2.03E-08
26292	2583	MYCBP	-0.44	1.42E-05	4.51E-05
10730	4224	YME1L1	-0.44	3.88E-12	2.20E-11
64718	4036	UNKL	-0.44	0.000494211	0.001266208
3913	5731	LAMB2	-0.44	4.68E-09	2.11E-08
6606	1588	SMN1	-0.44	0.000125385	0.000350619
23038	4583	WDTC1	-0.44	2.69E-07	1.03E-06
3069	6383	HDLBP	-0.44	3.38E-14	2.23E-13
126308	3347	MOB3A	-0.44	1.40E-07	5.54E-07
64110	1700	MAGEF1	-0.44	1.07E-06	3.85E-06
23616	2592	SH3BP1	-0.44	0.000229168	0.000617229
27303	7866	RBMS3	-0.44	2.40E-07	9.24E-07
64746	3581	ACBD3	-0.44	6.44E-07	2.37E-06
9088	2126	PKMYT1	-0.44	0.000237149	0.000637563
23154	3519	NCDN	-0.44	2.15E-05	6.69E-05

81490	2488	PTDSS2	-0.44	1.01E-05	3.26E-05
5431	4059	POLR2B	-0.44	1.51E-11	8.23E-11
7074	7213	TIAM1	-0.44	0.000580355	0.001472686
58491	3222	ZNF71	-0.45	0.016060155	0.031261704
26155	2817	NOC2L	-0.45	2.36E-09	1.09E-08
55249	2702	YY1AP1	-0.45	2.18E-06	7.62E-06
9739	6046	SETD1A	-0.45	3.71E-07	1.41E-06
54868	3721	TMEM104	-0.45	5.19E-05	0.000153657
9791	2573	PTDSS1	-0.45	2.04E-08	8.72E-08
10664	3791	CTCF	-0.45	4.86E-09	2.19E-08
28964	3729	GIT1	-0.45	2.60E-06	9.01E-06
55326	5535	AGPAT5	-0.45	7.60E-06	2.51E-05
23269	8380	MGA	-0.45	1.25E-06	4.47E-06
84108	2217	PCGF6	-0.45	2.72E-05	8.34E-05
146050	5570	ZSCAN29	-0.45	0.000105506	0.000298745
116254	1138	GINM1	-0.45	3.71E-07	1.41E-06
94103	2147	ORMDL3	-0.45	1.81E-08	7.80E-08
9093	2700	DNAJA3	-0.45	1.06E-06	3.83E-06
6595	3314	SMARCA2	-0.45	1.60E-08	6.91E-08
3299	2571	HSF4	-0.45	0.014377259	0.028242691
23074	5026	UHRF1BP1L	-0.45	2.70E-05	8.28E-05
29997	1523	NOP53	-0.45	1.03E-12	6.06E-12
51719	3825	CAB39	-0.45	1.16E-09	5.50E-09
149951	1958	COMMD7	-0.45	4.73E-07	1.77E-06
200916	1977	RPL22L1	-0.45	0.001240875	0.002973815
10140	2243	TOB1	-0.45	3.96E-07	1.50E-06
1793	6793	DOCK1	-0.45	3.27E-10	1.62E-09
29959	2272	NRBP1	-0.45	9.85E-09	4.34E-08
22847	7730	ZNF507	-0.45	5.17E-05	0.000153067
5881	1077	RAC3	-0.45	0.000616985	0.001556966
9425	3281	CDYL	-0.45	6.05E-05	0.000176887
3098	3574	HK1	-0.45	4.01E-10	1.96E-09
9372	5813	ZFYVE9	-0.45	8.26E-05	0.000237268
27249	1466	MMADHC	-0.45	1.93E-08	8.27E-08
3799	5661	KIF5B	-0.45	1.17E-13	7.39E-13
6059	4193	ABCE1	-0.45	3.05E-09	1.40E-08
148479	3565	PHF13	-0.45	8.26E-08	3.33E-07
23326	5049	USP22	-0.45	1.19E-11	6.53E-11
6936	3099	GCFC2	-0.45	0.000173308	0.000475719
9167	1159	COX7A2L	-0.45	9.41E-08	3.77E-07
8675	4920	STX16	-0.45	5.38E-07	2.00E-06
27349	1515	MCAT	-0.45	0.001071305	0.002599337
6259	2828	RYK	-0.45	7.41E-10	3.56E-09
11237	6615	RNF24	-0.45	3.57E-06	1.22E-05
339318	2866	ZNF181	-0.45	0.005809794	0.012341992
130507	7918	UBR3	-0.45	4.54E-05	0.00013536
84549	3590	MAK16	-0.45	0.000192782	0.000525442
348180	1680	CTU2	-0.45	0.004258356	0.009270089

5442	3803	POLRMT	-0.45	5.21E-06	1.75E-05
64319	4403	FBRS	-0.45	1.21E-06	4.33E-06
79019	958	CENPM	-0.45	4.90E-05	0.000145689
11104	1839	KATNA1	-0.45	0.00011396	0.0003212
2992	1818	GYG1	-0.45	2.05E-05	6.39E-05
26168	2509	SENP3	-0.45	3.43E-08	1.44E-07
23019	8393	CNOT1	-0.45	1.21E-12	7.10E-12
4686	5023	NCBP1	-0.45	6.41E-09	2.86E-08
9846	6118	GAB2	-0.45	0.000191198	0.000521525
91369	4112	ANKRD40	-0.45	7.51E-08	3.05E-07
1.03E+08	989	U2AF1L5	-0.45	0.00107013	0.002596841
148206	8808	ZNF714	-0.45	9.29E-05	0.000265119
51663	4746	ZFR	-0.45	8.86E-11	4.59E-10
51104	3156	ABHD17B	-0.45	0.001484246	0.003510193
254428	4748	SLC41A1	-0.45	2.19E-07	8.47E-07
57597	10490	BAHCC1	-0.45	0.000168367	0.000462943
10127	3182	ZNF263	-0.45	3.78E-06	1.29E-05
5532	2964	PPP3CB	-0.45	6.83E-07	2.51E-06
8525	3623	DGKZ	-0.45	1.07E-06	3.85E-06
7277	1495	TUBA4A	-0.46	4.72E-06	1.59E-05
135112	4927	NCOA7	-0.46	1.21E-06	4.35E-06
23431	6643	AP4E1	-0.46	9.10E-06	2.97E-05
84905	3574	ZNF341	-0.46	0.016779865	0.032559205
6389	2887	SDHA	-0.46	1.84E-13	1.15E-12
63892	4779	THADA	-0.46	9.70E-06	3.15E-05
23170	3421	TTLL12	-0.46	1.44E-05	4.58E-05
1163	834	CKS1B	-0.46	0.000125338	0.000350545
23659	2691	PLA2G15	-0.46	8.98E-06	2.93E-05
1806	4063	DPYD	-0.46	9.23E-05	0.000263529
1650	2144	DDOST	-0.46	5.75E-15	3.97E-14
10634	2705	GAS2L1	-0.46	0.000259732	0.000694287
25913	3848	POT1	-0.46	1.50E-05	4.76E-05
9486	2688	CHST10	-0.46	0.000105916	0.000299765
7301	4001	TYRO3	-0.46	4.42E-06	1.50E-05
10885	3877	WDR3	-0.46	1.27E-05	4.08E-05
11224	475	RPL35	-0.46	2.02E-09	9.41E-09
5787	9466	PTPRB	-0.46	0.008578569	0.017613128
7695	3251	ZNF136	-0.46	0.007827339	0.016190472
55215	4762	FANCI	-0.46	3.54E-10	1.74E-09
1181	3077	CLCN2	-0.46	0.01186123	0.023642623
23648	3110	SSBP3	-0.46	4.50E-08	1.86E-07
55140	3232	ELP3	-0.46	0.000405989	0.00105632
729438	4223	CASTOR2	-0.46	5.87E-07	2.18E-06
6233	972	RPS27A	-0.46	2.72E-11	1.47E-10
83938	852	LRMDA	-0.46	0.01332968	0.026330378
9780	8106	PIEZO1	-0.46	3.47E-10	1.71E-09
51142	966	CHCHD2	-0.46	1.45E-08	6.28E-08
4217	4988	MAP3K5	-0.46	1.48E-06	5.25E-06

57198	5932	ATP8B2	-0.46	2.57E-07	9.87E-07
29855	5749	UBN1	-0.46	2.18E-09	1.01E-08
10152	6312	ABI2	-0.46	6.19E-09	2.77E-08
79174	1465	CRELD2	-0.46	5.60E-05	0.000164685
113115	1582	MTFR2	-0.46	0.006303558	0.013293961
558	4444	AXL	-0.46	1.39E-12	8.12E-12
84948	2412	TIGD5	-0.46	0.004526476	0.009821276
55718	3237	POLR3E	-0.46	3.90E-05	0.000117136
65082	2628	VPS33A	-0.46	3.01E-05	9.19E-05
84769	1360	MPV17L2	-0.46	0.000255156	0.000683184
375341	3590	C3orf62	-0.46	0.004102977	0.008952683
79172	4400	CENPO	-0.46	2.88E-07	1.10E-06
5827	977	PXMP2	-0.46	6.68E-05	0.000194412
7004	1787	TEAD4	-0.46	3.59E-05	0.000108338
54503	2340	ZDHHC13	-0.46	1.70E-06	6.01E-06
151230	4089	KLHL23	-0.46	1.80E-06	6.35E-06
56006	2341	SMG9	-0.46	2.11E-06	7.40E-06
6457	2035	SH3GL3	-0.46	0.019907799	0.037938962
57335	5513	ZNF286A	-0.47	2.87E-05	8.78E-05
221527	2164	ZBTB12	-0.47	0.003473784	0.007674981
55274	1690	PHF10	-0.47	3.39E-06	1.16E-05
8800	2740	PEX11A	-0.47	0.015308917	0.029920674
2193	1853	FARSA	-0.47	3.23E-06	1.11E-05
51363	4476	CHST15	-0.47	3.35E-07	1.27E-06
55103	7449	RALGPS2	-0.47	1.11E-06	4.01E-06
149345	1667	SHISA4	-0.47	0.000999913	0.0024371
7270	3220	TTF1	-0.47	3.19E-07	1.21E-06
10939	3223	AFG3L2	-0.47	4.65E-08	1.92E-07
89122	3380	TRIM4	-0.47	8.23E-06	2.70E-05
9672	4970	SDC3	-0.47	2.50E-11	1.35E-10
1545	5160	CYP1B1	-0.47	1.48E-08	6.40E-08
1459	1672	CSNK2A2	-0.47	2.02E-05	6.29E-05
9895	8218	TECPR2	-0.47	2.95E-05	9.02E-05
113	5753	ADCY7	-0.47	8.91E-06	2.91E-05
84311	1712	MRPL45	-0.47	1.80E-06	6.35E-06
1827	2363	RCAN1	-0.47	1.64E-06	5.79E-06
9212	1249	AURKB	-0.47	3.02E-08	1.27E-07
9772	4740	TMEM94	-0.47	3.58E-08	1.50E-07
113174	1545	SAAL1	-0.47	2.24E-05	6.97E-05
26060	6061	APPL1	-0.47	1.00E-10	5.19E-10
118460	1729	EXOSC6	-0.47	0.000191417	0.000521962
55145	2104	THAP1	-0.47	0.011632243	0.023222663
25917	3457	THUMPD3	-0.47	3.47E-08	1.46E-07
201562	3602	HACD2	-0.47	3.51E-10	1.73E-09
220134	2910	SKA1	-0.47	5.74E-06	1.92E-05
54778	4882	RNF111	-0.47	7.91E-06	2.60E-05
56986	2680	DTWD1	-0.47	0.001367582	0.003252904
26256	1485	CABYR	-0.47	0.003796847	0.008338048

25909	8684	AHCTF1	-0.47	1.53E-11	8.34E-11
23065	5707	EMC1	-0.47	6.21E-11	3.27E-10
6144	582	RPL21	-0.47	9.08E-08	3.65E-07
2257	6174	FGF12	-0.47	0.016230733	0.031572639
54434	8212	SSH1	-0.47	1.12E-08	4.88E-08
196528	7968	ARID2	-0.47	3.43E-08	1.44E-07
26020	3582	LRP10	-0.47	3.85E-13	2.33E-12
140775	8327	SMCR8	-0.47	1.68E-07	6.57E-07
170384	2023	FUT11	-0.47	2.85E-05	8.71E-05
55659	2583	ZNF416	-0.47	0.025872662	0.048116544
23588	1824	KLHDC2	-0.47	1.51E-05	4.79E-05
4999	3767	ORC2	-0.47	6.63E-07	2.44E-06
2799	5144	GNS	-0.47	6.07E-13	3.63E-12
692312	3240	PPAN-P2RY11	-0.47	0.001295236	0.003092
81875	2738	ISG20L2	-0.47	9.07E-08	3.64E-07
55810	4985	FOXJ2	-0.47	1.69E-06	5.97E-06
6596	5185	HLTF	-0.47	9.23E-10	4.40E-09
10512	5172	SEMA3C	-0.47	5.05E-07	1.89E-06
6548	4259	SLC9A1	-0.48	3.60E-06	1.23E-05
9487	3810	PIGL	-0.48	0.002304721	0.005256776
6229	662	RPS24	-0.48	4.16E-15	2.90E-14
6683	5192	SPAST	-0.48	3.04E-06	1.04E-05
339230	2052	CCDC137	-0.48	2.06E-06	7.20E-06
22954	3736	TRIM32	-0.48	5.71E-07	2.12E-06
5127	3209	CDK16	-0.48	5.53E-10	2.68E-09
65260	2114	COA7	-0.48	0.000832748	0.002055322
133957	1437	CCDC127	-0.48	0.000806825	0.001996333
10713	2239	USP39	-0.48	1.44E-09	6.74E-09
7812	3890	CSDE1	-0.48	8.67E-18	7.04E-17
6046	3685	BRD2	-0.48	1.40E-12	8.20E-12
9797	4909	TATDN2	-0.48	2.34E-08	9.95E-08
80349	1370	WDR61	-0.48	7.65E-05	0.000220872
283742	4392	FAM98B	-0.48	6.78E-09	3.02E-08
51272	2850	BET1L	-0.48	6.43E-07	2.37E-06
26512	4796	INTS6	-0.48	8.53E-09	3.77E-08
50512	2209	PODXL2	-0.48	0.021585613	0.040797061
79692	4817	ZNF322	-0.48	8.31E-06	2.72E-05
115704	3835	EVI5L	-0.48	2.21E-05	6.85E-05
28952	2333	CCDC22	-0.48	0.000535683	0.001365177
84310	1336	C7orf50	-0.48	2.86E-08	1.21E-07
113419	3381	TEX261	-0.48	3.18E-09	1.46E-08
51759	1817	C9orf78	-0.48	3.44E-08	1.44E-07
36	5941	ACADSB	-0.48	8.53E-05	0.000244725
56997	2872	COQ8A	-0.48	0.00014449	0.000401516
1965	4165	EIF2S1	-0.48	9.89E-12	5.47E-11
54556	1841	ING3	-0.48	6.31E-05	0.00018414
3915	7889	LAMC1	-0.48	3.28E-16	2.44E-15
30968	1426	STOML2	-0.48	9.10E-10	4.34E-09

23506	6541	BICRAL	-0.48	0.000116411	0.000327535
9100	3401	USP10	-0.48	5.38E-13	3.22E-12
388677	5324	NOTCH2NL	-0.48	0.003152303	0.007017932
55022	2609	PID1	-0.48	0.016794433	0.032583914
168544	2863	ZNF467	-0.48	0.015062611	0.029468451
10421	3439	CD2BP2	-0.48	2.42E-09	1.12E-08
1.08E+08	2306	LOC107985803	-0.48	0.001904211	0.00441064
55341	3359	LSG1	-0.48	1.18E-07	4.71E-07
2957	6106	GTF2A1	-0.48	1.81E-08	7.79E-08
57488	5957	ESYT2	-0.48	1.25E-09	5.89E-09
90417	1768	KNSTRN	-0.48	1.07E-06	3.86E-06
80315	7152	CPEB4	-0.48	2.97E-08	1.25E-07
127933	8277	UHMK1	-0.48	5.24E-11	2.77E-10
255403	4549	ZNF718	-0.48	0.002646524	0.005975024
84057	953	MND1	-0.49	0.006173278	0.013050185
286205	12168	SCAI	-0.49	2.20E-05	6.82E-05
29789	4353	OLA1	-0.49	4.60E-12	2.60E-11
29028	4696	ATAD2	-0.49	1.60E-13	1.00E-12
90459	4582	ERI1	-0.49	6.87E-06	2.28E-05
2649	7042	NR6A1	-0.49	0.002587383	0.005852657
63893	5309	UBE2O	-0.49	8.88E-07	3.23E-06
5595	1898	MAPK3	-0.49	4.63E-07	1.74E-06
9632	4527	SEC24C	-0.49	1.43E-11	7.83E-11
91442	1565	FAAP24	-0.49	0.005631687	0.011990871
23204	2417	ARL6IP1	-0.49	1.10E-16	8.39E-16
8895	4890	CPNE3	-0.49	1.34E-09	6.30E-09
65108	1562	MARCKSL1	-0.49	5.92E-07	2.19E-06
55920	4008	RCC2	-0.49	1.46E-12	8.56E-12
26521	823	TIMM8B	-0.49	0.001776476	0.004134175
171568	3995	POLR3H	-0.49	2.30E-06	8.00E-06
24149	7900	ZNF318	-0.49	3.29E-07	1.25E-06
7298	1603	TYMS	-0.49	5.20E-09	2.34E-08
155066	2741	ATP6V0E2	-0.49	5.19E-06	1.74E-05
54475	3081	NLE1	-0.49	0.000260397	0.000695855
1660	4543	DHX9	-0.49	1.27E-16	9.63E-16
254427	3460	PROSER2	-0.49	1.24E-10	6.35E-10
66005	1559	CHID1	-0.49	1.35E-08	5.88E-08
80028	7303	FBXL18	-0.49	1.38E-07	5.45E-07
8087	2667	FXR1	-0.49	3.36E-14	2.22E-13
57695	8032	USP37	-0.49	2.31E-06	8.05E-06
81603	2753	TRIM8	-0.49	2.72E-10	1.35E-09
26354	2047	GNL3	-0.49	1.38E-11	7.55E-11
55676	4625	SLC30A6	-0.49	4.27E-07	1.61E-06
9666	5310	DZIP3	-0.49	3.21E-05	9.74E-05
8573	8339	CASK	-0.49	2.34E-09	1.08E-08
2063	1804	NR2F6	-0.49	1.20E-10	6.16E-10
114034	2416	TOE1	-0.49	6.06E-06	2.02E-05
389072	7859	PLEKHM3	-0.49	0.000127286	0.000355599

1432	3485	MAPK14	-0.49	2.74E-09	1.26E-08
989	2416	SEPT7	-0.49	2.11E-13	1.31E-12
5430	6738	POLR2A	-0.49	2.55E-08	1.08E-07
26959	2767	HBP1	-0.49	5.87E-08	2.41E-07
51253	1489	MRPL37	-0.49	1.23E-08	5.36E-08
5520	2641	PPP2R2A	-0.49	4.54E-07	1.70E-06
4070	2080	TACSTD2	-0.49	1.47E-11	8.03E-11
11228	5326	RASSF8	-0.49	3.41E-08	1.43E-07
91661	4577	ZNF765	-0.49	0.004548004	0.009863168
55055	3207	ZWILCH	-0.49	1.08E-07	4.30E-07
25914	6743	RTTN	-0.49	1.01E-05	3.26E-05
29890	6650	RBM15B	-0.49	3.72E-08	1.55E-07
5903	10861	RANBP2	-0.49	2.24E-12	1.29E-11
55684	3073	RABL6	-0.49	5.29E-14	3.45E-13
55619	7802	DOCK10	-0.49	0.000155278	0.00042888
445329	1397	SULT1A4	-0.49	0.000915746	0.002247062
80325	1975	ABTB1	-0.49	0.00096946	0.002367422
27292	1573	DIMT1	-0.49	3.56E-05	0.000107635
1938	3163	EEF2	-0.49	3.35E-15	2.34E-14
440138	2595	ALG11	-0.49	0.003960566	0.008666422
27238	1746	GPKOW	-0.49	2.70E-05	8.28E-05
54707	1510	GPN2	-0.49	0.000422697	0.001096261
10113	2184	PREB	-0.49	7.03E-07	2.58E-06
1730	4539	DIAPH2	-0.49	0.000754817	0.001879163
56893	3617	UBQLN4	-0.49	8.87E-08	3.57E-07
7378	1492	UPP1	-0.50	0.000479124	0.001230392
7155	5453	TOP2B	-0.50	1.18E-13	7.44E-13
6154	609	RPL26	-0.50	1.60E-12	9.33E-12
9871	3973	SEC24D	-0.50	8.54E-10	4.08E-09
79109	3105	MAPKAP1	-0.50	1.70E-11	9.22E-11
10067	1583	SCAMP3	-0.50	2.80E-06	9.67E-06
7564	2550	ZNF16	-0.50	0.000459577	0.001184819
22938	2146	SNW1	-0.50	2.16E-12	1.25E-11
64794	4024	DDX31	-0.50	9.12E-06	2.97E-05
9368	2032	SLC9A3R1	-0.50	3.25E-07	1.24E-06
142891	6815	SAMD8	-0.50	4.00E-08	1.67E-07
29899	4121	GPSM2	-0.50	1.24E-07	4.91E-07
84449	5288	ZNF333	-0.50	0.003514382	0.007761782
347240	5468	KIF24	-0.50	0.000167866	0.000461637
5525	2811	PPP2R5A	-0.50	2.77E-07	1.06E-06
1964	4431	EIF1AX	-0.50	1.17E-11	6.42E-11
55234	7088	SMU1	-0.50	1.51E-11	8.22E-11
4644	12200	MYO5A	-0.50	1.15E-07	4.58E-07
7554	2210	ZNF8	-0.50	0.00548235	0.011698138
79673	3385	ZNF329	-0.50	0.001953507	0.004513642
10100	3211	TSPAN2	-0.50	0.000420295	0.00109019
83743	2456	GRWD1	-0.50	7.93E-06	2.61E-05
221079	7064	ARL5B	-0.50	3.75E-06	1.28E-05

1121	4807	CHM	-0.50	8.42E-08	3.40E-07
284439	3172	SLC25A42	-0.50	0.002654887	0.00599162
6138	2212	RPL15	-0.50	6.60E-16	4.82E-15
79571	4152	GCC1	-0.50	0.000224554	0.000605351
2177	5182	FANCD2	-0.50	2.65E-08	1.12E-07
10845	4788	CLPX	-0.50	7.65E-08	3.10E-07
83461	1921	CDCA3	-0.50	2.22E-06	7.73E-06
114794	8379	ELFN2	-0.50	4.38E-07	1.65E-06
375748	6733	ERCC6L2	-0.50	6.77E-08	2.76E-07
2230	3155	FDX1	-0.50	0.000100471	0.000285626
29071	1743	C1GALT1C1	-0.50	1.33E-06	4.76E-06
51714	3498	SELENOT	-0.50	2.51E-12	1.44E-11
9748	7705	SLK	-0.50	1.17E-10	6.02E-10
84722	1840	PSRC1	-0.50	0.000232425	0.000625345
6618	1561	SNAPC2	-0.50	0.000209113	0.00056639
4733	1467	DRG1	-0.50	1.68E-08	7.24E-08
222484	4593	LNX2	-0.50	2.39E-05	7.39E-05
339122	4497	RAB43	-0.50	0.002282173	0.005210619
163050	2963	ZNF564	-0.50	0.003926257	0.008596641
84662	3746	GLIS2	-0.50	2.16E-08	9.22E-08
9294	3589	S1PR2	-0.50	0.001155867	0.002787006
56681	3023	SAR1A	-0.50	3.04E-14	2.01E-13
6324	1668	SCN1B	-0.51	0.002184342	0.005001467
201627	4695	DENND6A	-0.51	4.73E-07	1.77E-06
25972	1169	UNC50	-0.51	2.58E-05	7.95E-05
1E+08	2940	ZBED6	-0.51	0.001358821	0.003233667
9897	3988	WASHC5	-0.51	1.14E-08	4.98E-08
7874	5249	USP7	-0.51	5.23E-13	3.14E-12
55320	4044	MIS18BP1	-0.51	4.25E-07	1.60E-06
51776	6121	MAP3K20	-0.51	1.75E-12	1.02E-11
23118	4198	TAB2	-0.51	1.08E-10	5.59E-10
9889	6897	ZBED4	-0.51	1.92E-06	6.75E-06
79879	5927	CCDC134	-0.51	2.24E-06	7.80E-06
9774	5881	BCLAF1	-0.51	3.15E-14	2.08E-13
22877	8418	MLXIP	-0.51	7.72E-14	4.95E-13
123811	2298	FOPNL	-0.51	7.42E-08	3.01E-07
5888	1756	RAD51	-0.51	0.000801586	0.0019842
55634	2452	KRBOX4	-0.51	0.001458515	0.003454397
2996	4424	GYPE	-0.51	0.00313057	0.006974792
124245	2944	ZC3H18	-0.51	2.35E-07	9.06E-07
3838	2126	KPNA2	-0.51	3.47E-16	2.57E-15
2021	1160	ENDOG	-0.51	0.003183071	0.007083616
8976	4447	WASL	-0.51	1.17E-10	6.01E-10
51304	3602	ZDHHC3	-0.51	1.91E-10	9.64E-10
5908	2165	RAP1B	-0.51	7.78E-14	4.99E-13
6839	2752	SUV39H1	-0.51	5.97E-05	0.000174879
93380	3704	MMGT1	-0.51	3.49E-08	1.46E-07
79035	1409	NABP2	-0.51	3.60E-05	0.000108701

3459	2144	IFNGR1	-0.51	3.20E-09	1.47E-08
54602	4671	NDFIP2	-0.51	1.14E-10	5.86E-10
153830	3592	RNF145	-0.51	9.01E-12	5.00E-11
3480	11776	IGF1R	-0.51	7.14E-10	3.43E-09
8318	1983	CDC45	-0.51	1.09E-06	3.93E-06
2539	2362	G6PD	-0.51	3.66E-08	1.53E-07
6857	4450	SYT1	-0.51	0.003838975	0.0084211
9451	4345	EIF2AK3	-0.51	1.48E-05	4.69E-05
79801	3471	SHCBP1	-0.51	7.35E-09	3.27E-08
79666	2977	PLEKHF2	-0.51	3.81E-05	0.000114796
51399	1273	TRAPPC4	-0.51	4.32E-05	0.000129259
55176	2432	SEC61A2	-0.51	0.000118221	0.000332209
50628	3882	GEMIN4	-0.51	4.96E-07	1.85E-06
148789	4672	B3GALNT2	-0.51	3.18E-08	1.34E-07
55809	7635	TRERF1	-0.51	0.001029523	0.00250343
49856	1678	WRAP73	-0.51	5.56E-05	0.000163495
2879	942	GPX4	-0.51	2.01E-08	8.62E-08
10236	2768	HNRNPR	-0.51	8.90E-19	7.58E-18
203245	3408	NAIF1	-0.51	0.000169984	0.000467173
4298	4459	MLLT1	-0.51	5.64E-10	2.73E-09
5433	2338	POLR2D	-0.51	8.06E-08	3.25E-07
79848	4276	CSPP1	-0.51	8.11E-07	2.96E-06
5528	3053	PPP2R5D	-0.52	1.14E-08	4.98E-08
284309	5292	ZNF776	-0.52	2.35E-05	7.27E-05
25996	1171	REXO2	-0.52	6.95E-07	2.55E-06
79892	3917	MCMBP	-0.52	5.32E-12	2.99E-11
3489	976	IGFBP6	-0.52	2.31E-09	1.07E-08
2118	1942	ETV4	-0.52	0.001753329	0.004085124
55006	1728	TRMT61B	-0.52	0.00070549	0.001765762
84236	5322	RHBDD1	-0.52	4.46E-06	1.51E-05
9887	5719	SMG7	-0.52	8.78E-08	3.53E-07
403	3891	ARL3	-0.52	7.83E-07	2.86E-06
51081	1432	MRPS7	-0.52	2.71E-08	1.15E-07
56983	3559	POGLUT1	-0.52	1.79E-05	5.64E-05
27079	2128	RPUSD2	-0.52	0.001735662	0.004050865
5434	2858	POLR2E	-0.52	2.90E-10	1.44E-09
11253	2844	MAN1B1	-0.52	2.15E-09	9.99E-09
7385	2226	UQCRC2	-0.52	1.32E-11	7.25E-11
79414	2440	LRFN3	-0.52	0.001365696	0.003248853
9724	5537	UTP14C	-0.52	1.25E-06	4.48E-06
441250	2618	TYW1B	-0.52	0.00110546	0.00267381
4928	6675	NUP98	-0.52	2.19E-14	1.47E-13
79446	1481	WDR25	-0.52	0.000445766	0.001151885
27005	1978	USP21	-0.52	1.36E-05	4.34E-05
60491	1610	NIF3L1	-0.52	7.18E-06	2.37E-05
7621	4018	ZNF70	-0.52	0.017469537	0.033757338
902	1646	CCNH	-0.52	7.44E-11	3.89E-10
80153	3480	EDC3	-0.52	8.06E-07	2.94E-06

56900	2775	TMEM167B	-0.52	1.19E-06	4.28E-06
84275	1346	SLC25A33	-0.52	0.000554913	0.001411754
89801	4840	PPP1R3F	-0.52	0.003584018	0.007908697
6598	1733	SMARCB1	-0.52	3.22E-10	1.59E-09
8455	6551	ATRN	-0.52	1.14E-11	6.26E-11
9610	2501	RIN1	-0.52	5.11E-06	1.72E-05
10855	4630	HPSE	-0.52	7.62E-08	3.09E-07
10592	5823	SMC2	-0.52	5.15E-15	3.57E-14
5859	2823	QARS	-0.52	9.48E-16	6.87E-15
11073	5139	TOPBP1	-0.52	1.04E-13	6.60E-13
92	5241	ACVR2A	-0.52	0.000174798	0.00047944
23518	4389	R3HDM1	-0.52	3.93E-07	1.49E-06
54919	3424	DNAAF5	-0.52	1.79E-07	6.98E-07
2068	3487	ERCC2	-0.52	1.47E-09	6.90E-09
6877	3297	TAF5	-0.52	0.004910892	0.010577855
139716	3897	GAB3	-0.52	0.018015405	0.034695178
23028	2921	KDM1A	-0.52	6.16E-14	3.99E-13
79598	7642	CEP97	-0.52	1.26E-06	4.49E-06
8665	1274	EIF3F	-0.52	1.29E-14	8.72E-14
23063	6258	WAPL	-0.52	1.00E-11	5.55E-11
8891	1693	EIF2B3	-0.53	2.07E-05	6.46E-05
7572	6293	ZNF24	-0.53	8.65E-13	5.13E-12
84933	1351	C8orf76	-0.53	0.001210362	0.002908141
770	1870	CA11	-0.53	3.59E-05	0.000108363
2790	1267	GNG10	-0.53	1.43E-10	7.29E-10
55857	2212	KIZ	-0.53	0.000966715	0.002361044
4702	859	NDUFA8	-0.53	7.70E-08	3.12E-07
79831	2481	KDM8	-0.53	0.017522191	0.033840684
221927	2986	BRAT1	-0.53	4.01E-08	1.67E-07
554282	2394	FAM72C	-0.53	0.001291444	0.003084605
401115	438	C4orf48	-0.53	0.000157657	0.000435246
7486	4178	WRN	-0.53	0.000154501	0.000427133
23510	3641	KCTD2	-0.53	1.25E-07	4.94E-07
5425	1655	POLD2	-0.53	5.79E-08	2.38E-07
90355	3064	C5orf30	-0.53	1.58E-05	5.01E-05
7980	2441	TFPI2	-0.53	2.37E-09	1.10E-08
51322	3619	WAC	-0.53	1.30E-14	8.74E-14
5708	2914	PSMD2	-0.53	2.33E-14	1.55E-13
55159	4918	RFWD3	-0.53	1.04E-13	6.62E-13
23186	5774	RCOR1	-0.53	1.22E-10	6.25E-10
84513	1453	PLPP5	-0.53	0.000402097	0.001046962
114112	2904	TXNRD3	-0.53	0.022682733	0.042657366
23411	3663	SIRT1	-0.53	2.21E-08	9.45E-08
3930	3821	LBR	-0.53	1.03E-15	7.44E-15
11060	3833	WWP2	-0.53	3.63E-09	1.65E-08
157506	3981	RDH10	-0.53	5.23E-05	0.000154744
201626	5496	PDE12	-0.53	2.97E-08	1.25E-07
127428	5739	TCEANC2	-0.53	6.98E-05	0.000202465

10128	5399	LRPPRC	-0.53	1.11E-16	8.47E-16
6305	7089	SBF1	-0.53	2.22E-13	1.37E-12
390	2745	RND3	-0.53	9.12E-13	5.41E-12
27248	2571	ERLEC1	-0.53	3.21E-09	1.47E-08
6782	4001	HSPA13	-0.53	2.43E-09	1.12E-08
2921	1166	CXCL3	-0.53	0.011621141	0.023205713
8975	7650	USP13	-0.53	1.92E-09	8.94E-09
6337	3323	SCNN1A	-0.53	5.61E-14	3.65E-13
25820	4963	ARIH1	-0.53	6.82E-11	3.58E-10
653820	2243	FAM72B	-0.53	0.000849307	0.002093865
5805	948	PTS	-0.53	2.84E-05	8.71E-05
5306	3696	PITPNA	-0.53	1.96E-12	1.14E-11
83746	3203	L3MBTL2	-0.54	2.81E-07	1.08E-06
745	5787	MYRF	-0.54	1.47E-08	6.35E-08
2886	2273	GRB7	-0.54	2.85E-07	1.09E-06
199731	2124	CADM4	-0.54	3.71E-08	1.55E-07
4292	2589	MLH1	-0.54	3.48E-09	1.59E-08
10681	3017	GNB5	-0.54	4.79E-07	1.79E-06
7086	2190	TKT	-0.54	2.99E-13	1.83E-12
55165	2607	CEP55	-0.54	3.65E-09	1.66E-08
2956	4120	MSH6	-0.54	7.78E-17	6.01E-16
84895	3608	MIGA2	-0.54	5.77E-08	2.37E-07
8658	9478	TNKS	-0.54	2.11E-09	9.79E-09
54557	5413	SGTB	-0.54	0.000100462	0.000285626
2356	2282	FPGS	-0.54	1.47E-09	6.91E-09
8567	5744	MADD	-0.54	6.43E-09	2.87E-08
868	6307	CBLB	-0.54	3.50E-07	1.33E-06
286826	3218	LIN9	-0.54	1.08E-05	3.50E-05
23031	5965	MAST3	-0.54	2.42E-06	8.42E-06
9663	6132	LPIN2	-0.54	7.67E-10	3.68E-09
5621	2675	PRNP	-0.54	1.48E-13	9.31E-13
2300	3207	FOXL1	-0.54	0.001772504	0.004125471
93517	4082	SDR42E1	-0.54	2.93E-05	8.96E-05
57215	1903	THAP11	-0.54	2.46E-05	7.61E-05
388558	3848	ZNF808	-0.54	0.024867651	0.046383416
91687	2340	CENPL	-0.54	5.49E-05	0.000161691
593	1790	BCKDHA	-0.54	8.72E-08	3.51E-07
114990	2864	VASN	-0.54	8.60E-11	4.46E-10
7398	3594	USP1	-0.54	7.60E-16	5.53E-15
3309	3973	HSPA5	-0.54	1.24E-17	1.00E-16
23456	3442	ABCB10	-0.54	1.94E-07	7.56E-07
8444	4291	DYRK3	-0.54	0.000103785	0.000294389
23198	6940	PSME4	-0.54	6.27E-12	3.51E-11
63931	2160	MRPS14	-0.54	1.11E-06	3.99E-06
27106	2529	ARRDC2	-0.54	3.05E-07	1.16E-06
91860	4598	CALML4	-0.54	0.000171093	0.000469785
54918	3384	CMTM6	-0.54	1.69E-14	1.14E-13
25886	2102	POC1A	-0.54	1.11E-05	3.57E-05

2034	5123	EPAS1	-0.54	1.18E-14	8.00E-14
51755	7558	CDK12	-0.54	4.95E-11	2.62E-10
90580	1414	TIMM29	-0.54	0.001138221	0.002747067
27085	3087	MTBP	-0.54	9.34E-05	0.000266388
25962	5425	VIRMA	-0.54	1.38E-11	7.54E-11
11113	7929	CIT	-0.54	4.43E-12	2.51E-11
261734	4361	NPHP4	-0.54	3.25E-05	9.87E-05
2972	3296	BRF1	-0.54	1.98E-09	9.22E-09
23293	4451	SMG6	-0.54	4.67E-10	2.28E-09
84224	3997	NBPF3	-0.54	0.000121018	0.000339103
286262	2641	TPRN	-0.54	5.70E-05	0.000167268
10733	3783	PLK4	-0.54	5.67E-07	2.11E-06
4946	1194	OAZ1	-0.54	4.13E-13	2.50E-12
51182	2690	HSPA14	-0.54	1.04E-08	4.56E-08
84937	4590	ZNRF1	-0.55	5.82E-06	1.95E-05
22809	2111	ATF5	-0.55	1.46E-08	6.31E-08
3204	2018	HOXA7	-0.55	8.02E-08	3.24E-07
22836	5531	RHOBTB3	-0.55	2.22E-16	1.67E-15
26149	4088	ZNF658	-0.55	0.002951373	0.00660143
92370	3303	PXYLP1	-0.55	5.98E-05	0.00017491
9726	6230	ZNF646	-0.55	7.42E-07	2.71E-06
197407	3145	ZNF48	-0.55	8.22E-05	0.000236317
51236	2549	HGH1	-0.55	2.13E-06	7.45E-06
7110	6903	TMF1	-0.55	7.83E-11	4.08E-10
55839	2357	CENPN	-0.55	7.23E-09	3.22E-08
11094	2968	CACFD1	-0.55	4.32E-07	1.63E-06
57693	4051	ZNF317	-0.55	2.22E-09	1.03E-08
2178	2425	FANCE	-0.55	0.000211486	0.000572121
203197	4470	TMEM268	-0.55	6.02E-05	0.000176015
53373	5152	TPCN1	-0.55	2.86E-15	2.02E-14
126731	5121	CCSAP	-0.55	4.35E-06	1.47E-05
163126	1409	EID2	-0.55	5.54E-06	1.86E-05
55762	5244	ZNF701	-0.55	0.000154161	0.000426391
90701	834	SEC11C	-0.55	1.04E-05	3.38E-05
7813	6971	EVI5	-0.55	1.97E-06	6.92E-06
112939	4526	NACC1	-0.55	5.68E-12	3.19E-11
3198	2561	HOXA1	-0.55	0.007875669	0.016282859
5573	3807	PRKAR1A	-0.55	3.29E-13	2.01E-12
3658	6110	IREB2	-0.55	3.25E-12	1.86E-11
64327	4536	LMBR1	-0.55	5.48E-09	2.46E-08
84890	3739	ADO	-0.55	3.80E-08	1.59E-07
27158	5048	NDOR1	-0.55	1.94E-10	9.76E-10
60485	3234	SAV1	-0.55	1.11E-11	6.14E-11
79188	3347	TMEM43	-0.55	3.23E-12	1.84E-11
23516	4706	SLC39A14	-0.55	3.94E-10	1.93E-09
728489	746	DNLZ	-0.55	0.001751584	0.004081594
23013	12227	SPEN	-0.55	1.31E-09	6.16E-09
2002	2732	ELK1	-0.55	1.63E-08	7.02E-08

83939	2996	EIF2A	-0.55	1.01E-12	5.95E-12
4204	7016	MECP2	-0.55	3.66E-10	1.80E-09
3419	2696	IDH3A	-0.56	1.76E-08	7.58E-08
283464	7409	GXYLT1	-0.56	1.91E-10	9.61E-10
54852	5027	PAQR5	-0.56	0.006734442	0.014127474
84444	7867	DOT1L	-0.56	1.93E-08	8.27E-08
54464	9798	XRN1	-0.56	1.05E-07	4.21E-07
54509	2451	RHOF	-0.56	7.48E-08	3.03E-07
23473	4133	CAPN7	-0.56	2.16E-09	1.00E-08
403341	6627	ZBTB34	-0.56	3.06E-06	1.05E-05
6575	3753	SLC20A2	-0.56	7.37E-08	2.99E-07
6124	1458	RPL4	-0.56	5.24E-22	5.20E-21
375	1904	ARF1	-0.56	1.08E-13	6.89E-13
414149	3383	ACBD7	-0.56	4.00E-06	1.36E-05
10579	3732	TACC2	-0.56	1.17E-05	3.76E-05
91664	5591	ZNF845	-0.56	0.000224098	0.000604398
139285	8443	AMER1	-0.56	0.020876989	0.039571404
26099	3693	SZRD1	-0.56	4.00E-13	2.42E-12
7026	4201	NR2F2	-0.56	7.66E-12	4.27E-11
80853	8503	KDM7A	-0.56	0.003447698	0.007621137
9094	1466	UNC119	-0.56	5.92E-07	2.20E-06
60401	3407	EDA2R	-0.56	1.31E-05	4.18E-05
79832	9289	QSER1	-0.56	1.27E-12	7.48E-12
55628	8085	ZNF407	-0.56	3.26E-06	1.12E-05
83941	1250	TM2D1	-0.56	4.03E-06	1.37E-05
79989	4288	TTC26	-0.56	2.79E-05	8.56E-05
29127	3192	RACGAP1	-0.56	1.74E-13	1.09E-12
8867	6942	SYNJ1	-0.56	6.28E-06	2.09E-05
6621	4588	SNAPC4	-0.56	1.46E-06	5.20E-06
26608	2553	TBL2	-0.56	5.92E-07	2.20E-06
4171	3504	MCM2	-0.56	1.32E-12	7.75E-12
3148	1516	HMGB2	-0.56	1.21E-21	1.18E-20
2937	1937	GSS	-0.56	3.81E-13	2.31E-12
7443	1715	VRK1	-0.56	6.30E-08	2.57E-07
151648	2837	SGO1	-0.56	1.32E-07	5.22E-07
23528	4878	ZNF281	-0.56	7.78E-11	4.06E-10
123606	6499	NIPA1	-0.56	9.65E-10	4.60E-09
5310	13768	PKD1	-0.56	3.82E-13	2.32E-12
51194	4420	IPO11	-0.56	2.09E-07	8.12E-07
57613	4651	FAM234B	-0.56	2.50E-06	8.65E-06
56981	7282	PRDM11	-0.56	0.000101537	0.000288472
55612	5167	FERMT1	-0.56	0.011803114	0.023534702
9839	5805	ZEB2	-0.56	4.37E-06	1.48E-05
7326	4208	UBE2G1	-0.56	2.84E-10	1.41E-09
22936	5688	ELL2	-0.56	5.67E-12	3.18E-11
79693	1871	YRDC	-0.56	0.000399895	0.001041381
84248	3713	FYTTD1	-0.56	2.07E-13	1.29E-12
54952	1902	TRNAU1AP	-0.56	3.41E-07	1.30E-06

54807	2158	ZNF586	-0.56	0.002398983	0.005455978
25799	3339	ZNF324	-0.56	0.000231017	0.000621926
165324	2061	UBXN2A	-0.57	8.33E-07	3.03E-06
25778	7418	DSTYK	-0.57	4.16E-09	1.89E-08
646962	971	HRCT1	-0.57	0.007270575	0.015137518
644096	1147	SDHAF1	-0.57	8.98E-05	0.000256782
7428	4560	VHL	-0.57	1.04E-15	7.54E-15
125950	3579	RAVER1	-0.57	9.03E-08	3.63E-07
9252	5433	RPS6KA5	-0.57	1.37E-05	4.38E-05
3149	3586	HMGB3	-0.57	2.66E-14	1.77E-13
285237	2752	C3orf38	-0.57	1.35E-07	5.32E-07
7549	3917	ZNF2	-0.57	0.002173007	0.004979364
10293	1990	TRAIP	-0.57	0.000224266	0.00060476
51087	1606	YBX2	-0.57	0.020071174	0.038211145
29888	2953	STRN4	-0.57	8.98E-12	4.98E-11
5536	2231	PPP5C	-0.57	2.92E-16	2.17E-15
89890	5228	KBTBD6	-0.57	3.65E-06	1.24E-05
59339	5418	PLEKHA2	-0.57	4.28E-12	2.42E-11
10347	5491	ABCA7	-0.57	3.66E-11	1.96E-10
54537	3477	FAM35A	-0.57	2.01E-10	1.01E-09
25973	2403	PARS2	-0.57	0.019576095	0.03738077
84516	7216	DCTN5	-0.57	1.50E-11	8.21E-11
129293	3042	TRABD2A	-0.57	7.93E-06	2.61E-05
56648	5537	EIF5A2	-0.57	3.16E-06	1.09E-05
488	8329	ATP2A2	-0.57	1.26E-18	1.07E-17
10454	3240	TAB1	-0.57	1.19E-08	5.19E-08
203069	1651	R3HCC1	-0.57	0.000116254	0.000327198
2009	4200	EML1	-0.57	0.00016243	0.000447936
8833	3239	GMPS	-0.57	2.28E-19	2.01E-18
29087	1019	THYN1	-0.57	5.00E-07	1.87E-06
23005	6067	MAPKBP1	-0.57	5.11E-07	1.91E-06
51616	2714	TAF9B	-0.58	4.85E-11	2.58E-10
143458	3824	LDLRAD3	-0.58	1.43E-05	4.57E-05
84271	3379	POLDIP3	-0.58	4.07E-13	2.47E-12
2117	5054	ETV3	-0.58	8.97E-08	3.60E-07
79813	3799	EHMT1	-0.58	4.68E-15	3.25E-14
6147	979	RPL23A	-0.58	0.002852858	0.006400199
3980	6164	LIG3	-0.58	1.30E-07	5.15E-07
3109	1412	HLA-DMB	-0.58	9.94E-05	0.000282635
5143	5126	PDE4C	-0.58	0.020373899	0.038729372
5862	3811	RAB2A	-0.58	3.21E-13	1.96E-12
79697	2502	RIOX1	-0.58	3.44E-06	1.18E-05
8452	3988	CUL3	-0.58	1.39E-16	1.06E-15
246243	1877	RNASEH1	-0.58	3.70E-05	0.000111558
9519	1353	TBPL1	-0.58	7.23E-06	2.39E-05
1522	1517	CTSZ	-0.58	5.93E-16	4.34E-15
11168	2834	PSIP1	-0.58	6.37E-16	4.66E-15
29800	2016	ZDHHC1	-0.58	0.012172353	0.024224727

3712	4507	IVD	-0.58	2.83E-09	1.30E-08
1796	2160	DOK1	-0.58	2.40E-07	9.24E-07
163859	4029	SDE2	-0.58	1.40E-05	4.45E-05
3720	5675	JARID2	-0.58	7.94E-07	2.90E-06
80267	6563	EDEM3	-0.58	1.42E-10	7.22E-10
4085	1453	MAD2L1	-0.58	2.28E-07	8.81E-07
389792	2724	IER5L	-0.58	3.27E-06	1.12E-05
5597	4204	MAPK6	-0.58	1.46E-12	8.54E-12
2200	11695	FBN1	-0.58	9.26E-17	7.11E-16
57325	3714	KAT14	-0.58	1.27E-05	4.08E-05
4216	5505	MAP3K4	-0.58	1.42E-09	6.69E-09
2909	8968	ARHGAP35	-0.58	5.53E-19	4.75E-18
154743	3969	BMT2	-0.58	9.87E-07	3.57E-06
55252	12502	ASXL2	-0.58	4.25E-09	1.92E-08
199953	3889	TMEM201	-0.58	3.40E-08	1.43E-07
64682	7768	ANAPC1	-0.58	2.80E-13	1.72E-12
24137	4504	KIF4A	-0.58	2.47E-13	1.52E-12
3306	2802	HSPA2	-0.58	1.87E-13	1.17E-12
126792	2793	B3GALT6	-0.58	4.54E-09	2.05E-08
11143	3202	KAT7	-0.58	4.34E-11	2.31E-10
10972	4163	TMED10	-0.58	4.55E-21	4.31E-20
55012	2021	PPP2R3C	-0.58	8.77E-06	2.87E-05
10797	2128	MTHFD2	-0.58	4.83E-12	2.72E-11
90780	3227	PYGO2	-0.58	8.43E-12	4.68E-11
57456	5157	KIAA1143	-0.58	1.58E-13	9.89E-13
2588	2804	GALNS	-0.58	9.70E-10	4.62E-09
55775	3534	TDP1	-0.59	2.31E-07	8.92E-07
2966	1827	GTF2H2	-0.59	2.62E-07	1.00E-06
1727	2923	CYB5R3	-0.59	2.81E-11	1.51E-10
6883	1134	TAF12	-0.59	2.42E-07	9.31E-07
65979	6140	PHACTR4	-0.59	2.12E-15	1.51E-14
26995	1439	TRUB2	-0.59	1.52E-10	7.71E-10
10959	2126	TMED2	-0.59	3.05E-18	2.53E-17
116841	2427	SNAP47	-0.59	9.49E-08	3.80E-07
8224	7443	SYN3	-0.59	0.014285491	0.028077942
26112	3416	CCDC69	-0.59	0.000208079	0.000563848
56852	5412	RAD18	-0.59	3.49E-08	1.46E-07
10049	1761	DNAJB6	-0.59	7.57E-16	5.51E-15
29882	2733	ANAPC2	-0.59	5.24E-09	2.35E-08
84455	2485	EFCAB7	-0.59	0.000154447	0.000427048
56919	5474	DHX33	-0.59	1.82E-11	9.87E-11
165215	3557	FAM171B	-0.59	0.003031284	0.006771417
51460	8002	SFMBT1	-0.59	4.08E-08	1.70E-07
79905	4064	TMC7	-0.59	0.006816854	0.014284924
10363	4060	HMG20A	-0.59	3.32E-10	1.64E-09
9306	5786	SOCS6	-0.59	6.95E-07	2.55E-06
113201	3896	CASC4	-0.59	1.57E-15	1.12E-14
90233	3737	ZNF551	-0.59	0.001977512	0.004564951

54585	3506	LZTFL1	-0.59	1.73E-07	6.78E-07
7693	4940	ZNF134	-0.59	3.73E-08	1.56E-07
84223	1950	IQCG	-0.59	0.001450101	0.003435845
57536	5922	KIAA1328	-0.59	0.001517545	0.003581794
55794	2610	DDX28	-0.59	6.75E-06	2.24E-05
23208	5298	SYT11	-0.59	2.31E-07	8.92E-07
55624	2746	POMGNT1	-0.59	7.67E-14	4.93E-13
3597	4017	IL13RA1	-0.59	1.43E-16	1.08E-15
10126	1504	DNAL4	-0.59	0.000642471	0.001616815
7559	5074	ZNF12	-0.59	1.62E-06	5.74E-06
51319	1757	RSRC1	-0.59	4.20E-07	1.58E-06
2975	7084	GTF3C1	-0.59	2.87E-19	2.51E-18
2039	2526	DMTN	-0.59	2.40E-07	9.26E-07
5253	5346	PHF2	-0.59	6.41E-13	3.83E-12
56985	1430	ADPRM	-0.59	0.00590147	0.012518783
51148	2411	CERCAM	-0.59	2.52E-13	1.55E-12
23514	2926	SPIDR	-0.59	9.58E-11	4.96E-10
79990	3045	PLEKHH3	-0.59	4.70E-06	1.59E-05
11031	4009	RAB31	-0.59	8.54E-12	4.74E-11
154807	5988	VKORC1L1	-0.59	1.03E-14	6.97E-14
28511	2462	NKIRAS2	-0.59	1.85E-13	1.15E-12
1.01E+08	5068	ISY1-RAB43	-0.59	3.20E-09	1.46E-08
55315	2277	SLC29A3	-0.60	0.021634147	0.040884442
10018	4603	BCL2L11	-0.60	4.68E-10	2.28E-09
83693	3668	HSDL1	-0.60	3.95E-08	1.65E-07
80742	3380	PRR3	-0.60	1.34E-07	5.29E-07
55033	5086	FKBP14	-0.60	1.20E-07	4.76E-07
57590	4682	WDFY1	-0.60	6.93E-14	4.47E-13
55756	2591	INTS9	-0.60	0.000370118	0.000967944
1407	3314	CRY1	-0.60	9.15E-08	3.67E-07
6769	2939	STAC	-0.60	7.68E-07	2.81E-06
26505	4930	CNNM3	-0.60	3.70E-09	1.68E-08
51138	1769	COPS4	-0.60	6.61E-08	2.70E-07
3065	2112	HDAC1	-0.60	1.95E-21	1.89E-20
7760	3294	ZNF213	-0.60	1.94E-05	6.06E-05
50807	6221	ASAP1	-0.60	1.63E-13	1.02E-12
92312	6124	MEX3A	-0.60	1.62E-06	5.74E-06
29883	2866	CNOT7	-0.60	1.45E-13	9.13E-13
22990	11078	PCNX1	-0.60	9.61E-14	6.13E-13
115557	2434	ARHGEF25	-0.60	4.23E-08	1.76E-07
1265	2492	CNN2	-0.60	8.55E-17	6.59E-16
4093	5517	SMAD9	-0.60	0.000120643	0.000338104
6904	3708	TBCD	-0.60	1.95E-14	1.30E-13
51277	4959	DNAJC27	-0.60	0.006424866	0.01353694
22894	7576	DIS3	-0.60	1.34E-13	8.45E-13
5912	8413	RAP2B	-0.60	1.99E-10	1.00E-09
23165	6286	NUP205	-0.60	6.71E-17	5.20E-16
9319	2392	TRIP13	-0.60	2.55E-11	1.37E-10

22907	4151	DHX30	-0.60	9.20E-14	5.88E-13
23020	7232	SNRNP200	-0.60	6.09E-21	5.75E-20
57104	2080	PNPLA2	-0.60	1.23E-09	5.84E-09
85457	4397	CIPC	-0.60	7.95E-05	0.000228982
23363	3928	OBSL1	-0.60	3.22E-16	2.40E-15
101	3172	ADAM8	-0.61	0.014047431	0.027640616
374819	4043	LRRC37A3	-0.61	4.61E-06	1.56E-05
4297	16649	KMT2A	-0.61	4.62E-14	3.03E-13
26122	4317	EPC2	-0.61	1.25E-09	5.89E-09
1051	2113	CEBPB	-0.61	7.88E-13	4.69E-12
3977	10041	LIFR	-0.61	1.32E-09	6.22E-09
9641	3460	IKBKE	-0.61	1.90E-05	5.95E-05
10767	6684	HBS1L	-0.61	9.87E-16	7.14E-15
2072	6765	ERCC4	-0.61	1.03E-05	3.35E-05
146223	4890	CMTM4	-0.61	5.88E-15	4.06E-14
23008	6346	KLHDC10	-0.61	8.69E-12	4.83E-11
4913	1103	NTHL1	-0.61	0.000163869	0.000451679
56967	7665	C14orf132	-0.61	5.29E-08	2.18E-07
162	4172	AP1B1	-0.61	1.41E-16	1.07E-15
8732	4656	RNGTT	-0.61	1.13E-08	4.95E-08
146059	4285	CDAN1	-0.61	2.76E-06	9.53E-06
728833	2518	FAM72D	-0.61	9.50E-05	0.000270888
1798	2014	DPAGT1	-0.61	1.34E-07	5.30E-07
57805	3873	CCAR2	-0.61	4.20E-15	2.92E-14
3202	1657	HOXA5	-0.61	6.44E-14	4.17E-13
4209	5960	MEF2D	-0.61	1.24E-08	5.38E-08
6125	1035	RPL5	-0.61	4.69E-23	4.88E-22
84881	2527	RPUSD4	-0.61	1.93E-05	6.03E-05
80709	5108	AKNA	-0.61	1.07E-08	4.68E-08
8535	2837	CBX4	-0.61	1.66E-07	6.52E-07
84187	5053	TMEM164	-0.61	2.76E-07	1.05E-06
55572	2104	FOXRED1	-0.61	9.22E-08	3.70E-07
3833	2260	KIFC1	-0.61	4.31E-13	2.60E-12
115	7660	ADCY9	-0.61	6.87E-10	3.31E-09
55254	4087	TMEM39A	-0.61	5.99E-08	2.46E-07
84321	1617	THOC3	-0.61	5.58E-14	3.63E-13
65124	4681	SOWAHC	-0.61	5.40E-08	2.22E-07
64900	4069	LPIN3	-0.61	2.23E-12	1.29E-11
23371	4762	TNS2	-0.61	1.45E-08	6.28E-08
80318	1628	GKAP1	-0.61	0.000162975	0.000449371
168374	3153	ZNF92	-0.61	7.21E-06	2.38E-05
9816	5652	URB2	-0.61	1.14E-05	3.67E-05
91754	5556	NEK9	-0.61	4.43E-14	2.90E-13
4867	2435	NPHP1	-0.61	0.000991147	0.002418057
92154	4766	MTSS1L	-0.61	6.03E-12	3.38E-11
55197	3732	RPRD1A	-0.61	2.84E-13	1.74E-12
373	3812	TRIM23	-0.61	0.000113685	0.00032053
284370	4027	ZNF615	-0.61	0.000504591	0.00129131

22937	3685	SCAP	-0.61	2.44E-16	1.83E-15
64236	1967	PDLIM2	-0.61	0.000117873	0.000331334
9609	4987	RAB36	-0.61	2.66E-05	8.18E-05
337867	2301	UBAC2	-0.61	7.74E-12	4.31E-11
11051	4407	NUDT21	-0.61	6.99E-23	7.22E-22
55313	6110	CPPED1	-0.61	5.80E-14	3.77E-13
79709	3428	COLGALT1	-0.62	1.18E-14	7.95E-14
26156	5137	RSL1D1	-0.62	1.10E-20	1.03E-19
22929	3295	SEPHS1	-0.62	5.70E-16	4.18E-15
152189	1192	CMTM8	-0.62	0.002064076	0.004748737
56255	6164	TMX4	-0.62	1.95E-14	1.31E-13
1138	2682	CHRNA5	-0.62	1.02E-06	3.69E-06
8541	4728	PPFIA3	-0.62	0.00012713	0.000355221
9653	6149	HS2ST1	-0.62	6.49E-17	5.04E-16
25885	6637	POLR1A	-0.62	1.26E-09	5.97E-09
8703	2195	B4GALT3	-0.62	7.91E-09	3.51E-08
26119	3014	LDLRAP1	-0.62	6.24E-08	2.55E-07
50809	3924	HP1BP3	-0.62	4.73E-22	4.70E-21
114987	4908	WDR31	-0.62	0.001060376	0.002574929
4303	3348	FOXO4	-0.62	1.90E-05	5.97E-05
93	11373	ACVR2B	-0.62	2.26E-06	7.89E-06
865	3105	CBFB	-0.62	7.81E-19	6.68E-18
57521	6585	RPTOR	-0.62	2.92E-11	1.57E-10
285367	1231	RPUSD3	-0.62	1.50E-05	4.76E-05
23505	6667	TMEM131	-0.62	8.33E-13	4.95E-12
27252	3503	KLHL20	-0.62	7.80E-07	2.85E-06
3692	1153	EIF6	-0.62	3.30E-15	2.31E-14
701	3749	BUB1B	-0.62	1.64E-17	1.31E-16
63906	2145	GPATCH3	-0.62	2.14E-05	6.65E-05
57185	4385	NIPAL3	-0.62	6.84E-14	4.42E-13
402778	3721	IFITM10	-0.62	1.27E-05	4.08E-05
5885	3773	RAD21	-0.63	1.64E-24	1.82E-23
79856	3566	SNX22	-0.63	0.004666149	0.010104587
152002	2608	XXYLT1	-0.63	1.33E-10	6.80E-10
79959	2813	CEP76	-0.63	3.53E-05	0.000106714
339745	5737	SPOPL	-0.63	1.27E-08	5.53E-08
114757	2166	CYGB	-0.63	0.00056971	0.001446912
120114	17975	FAT3	-0.63	0.006892045	0.01441868
57787	3604	MARK4	-0.63	4.79E-10	2.34E-09
1.01E+08	6256	C15orf38-AP3S2	-0.63	0.000365728	0.000956886
1E+08	5865	POM121C	-0.63	1.39E-11	7.64E-11
10782	2347	ZNF274	-0.63	1.13E-06	4.06E-06
5218	4872	CDK14	-0.63	2.72E-08	1.15E-07
54892	3434	NCAPG2	-0.63	1.11E-18	9.42E-18
84629	10569	TNRC18	-0.63	1.12E-16	8.59E-16
79753	4605	SNIP1	-0.63	2.74E-07	1.05E-06
54970	2411	TTC12	-0.63	0.00059837	0.001514723
55342	3703	STRBP	-0.63	9.77E-12	5.41E-11

85358	7113	SHANK3	-0.63	4.07E-06	1.38E-05
65094	2612	JMJD4	-0.63	3.81E-06	1.30E-05
29089	935	UBE2T	-0.63	1.52E-11	8.27E-11
9156	3078	EXO1	-0.63	1.80E-07	7.03E-07
55663	2227	ZNF446	-0.63	8.04E-06	2.64E-05
91775	8728	NXPE3	-0.63	2.87E-11	1.54E-10
408050	4318	NOMO3	-0.63	9.26E-09	4.09E-08
767558	3888	LUZP6	-0.63	1.65E-20	1.53E-19
83892	4179	KCTD10	-0.63	1.26E-16	9.57E-16
348	1234	APOE	-0.63	0.000316278	0.000835012
22874	7623	PLEKHA6	-0.63	2.68E-09	1.24E-08
60312	7527	AFAP1	-0.63	2.65E-12	1.52E-11
6776	3945	STAT5A	-0.63	1.35E-06	4.81E-06
162394	8555	SLFN5	-0.63	1.72E-15	1.23E-14
54407	4913	SLC38A2	-0.63	5.20E-18	4.27E-17
51360	4541	MBTPS2	-0.63	9.40E-09	4.15E-08
5469	8105	MED1	-0.63	1.76E-17	1.41E-16
26504	3801	CNNM4	-0.63	8.87E-08	3.57E-07
2800	4897	GOLGA1	-0.63	1.06E-08	4.67E-08
79812	6159	MMRN2	-0.63	0.005424575	0.011588696
10061	3172	ABCF2	-0.64	4.80E-15	3.33E-14
1.08E+08	1099	LOC107985082	-0.64	0.003923288	0.008591199
54665	6712	RSBN1	-0.64	4.37E-07	1.64E-06
10808	3570	HSPH1	-0.64	1.75E-17	1.40E-16
11339	1249	OIP5	-0.64	3.15E-05	9.59E-05
2671	2447	GFER	-0.64	3.63E-08	1.52E-07
2004	3951	ELK3	-0.64	2.60E-14	1.73E-13
10131	2304	TRAP1	-0.64	1.94E-15	1.38E-14
4035	14919	LRP1	-0.64	2.81E-19	2.46E-18
9990	7582	SLC12A6	-0.64	1.30E-10	6.62E-10
57670	12420	KIAA1549	-0.64	2.40E-06	8.34E-06
1112	7879	FOXN3	-0.64	1.36E-18	1.15E-17
6491	4622	STIL	-0.64	7.37E-14	4.74E-13
5796	5198	PTPRK	-0.64	8.02E-19	6.83E-18
752	4151	FMNL1	-0.64	9.82E-08	3.93E-07
56998	2965	CTNNBIP1	-0.64	6.26E-08	2.56E-07
127703	2934	C1orf216	-0.64	1.33E-09	6.28E-09
2033	8554	EP300	-0.64	8.43E-11	4.38E-10
55233	4920	MOB1A	-0.64	1.81E-24	2.01E-23
140460	4781	ASB7	-0.64	1.95E-08	8.37E-08
5082	3654	PDCL	-0.64	1.30E-12	7.60E-12
29763	1920	PACSIN3	-0.64	2.65E-09	1.22E-08
1889	5058	ECE1	-0.64	1.83E-24	2.02E-23
55739	2530	NAXD	-0.64	6.66E-12	3.72E-11
1.01E+08	1387	CENPS-CORT	-0.64	0.006716562	0.014098036
3188	2390	HNRNPH2	-0.64	2.14E-14	1.43E-13
65249	4437	ZSWIM4	-0.64	5.60E-09	2.51E-08
352954	3743	GATS	-0.64	8.05E-11	4.19E-10

90007	3825	MIDN	-0.64	1.12E-15	8.12E-15
64781	4339	CERK	-0.64	7.26E-11	3.80E-10
8458	6241	TTF2	-0.65	8.66E-11	4.49E-10
8125	2479	ANP32A	-0.65	1.16E-21	1.13E-20
51096	1894	UTP18	-0.65	1.35E-10	6.91E-10
8450	4727	CUL4B	-0.65	1.70E-17	1.36E-16
54904	4272	NSD3	-0.65	1.61E-12	9.40E-12
144132	8884	DNHD1	-0.65	0.001588517	0.003731954
55722	2291	CEP72	-0.65	0.00158377	0.003721785
4794	2581	NFKBIE	-0.65	7.05E-06	2.33E-05
55290	2007	BRF2	-0.65	0.002926669	0.006551727
54107	2281	POLE3	-0.65	9.31E-17	7.15E-16
352909	2231	DNAAF3	-0.65	0.014707141	0.028833331
11346	5413	SYNPO	-0.65	1.04E-14	7.05E-14
4783	2039	NFIL3	-0.65	2.69E-11	1.45E-10
9070	2719	ASH2L	-0.65	1.15E-10	5.92E-10
124583	3435	CANT1	-0.65	6.35E-16	4.64E-15
23580	3146	CDC42EP4	-0.65	1.10E-10	5.64E-10
4706	799	NDUFAB1	-0.65	1.47E-05	4.67E-05
57555	4378	NLGN2	-0.65	7.05E-11	3.69E-10
10189	1113	ALYREF	-0.65	0.001188686	0.002859542
145508	4566	CEP128	-0.65	4.70E-06	1.58E-05
53981	5075	CPSF2	-0.65	3.12E-18	2.59E-17
150864	5795	FAM117B	-0.65	7.02E-07	2.58E-06
388566	7149	ZNF470	-0.65	0.000456738	0.001178353
115992	2321	RNF166	-0.65	2.28E-08	9.72E-08
54996	2139	MARC2	-0.65	4.34E-06	1.47E-05
255057	3035	CBARP	-0.66	0.000443465	0.001147109
10244	1376	RABEPK	-0.66	4.55E-06	1.54E-05
134957	9285	STXBP5	-0.66	6.64E-11	3.48E-10
200316	2033	APOBEC3F	-0.66	1.14E-05	3.67E-05
10403	2209	NDC80	-0.66	8.12E-17	6.27E-16
58476	3932	TP53INP2	-0.66	2.05E-11	1.11E-10
7916	6926	PRRC2A	-0.66	2.91E-16	2.17E-15
10838	6320	ZNF275	-0.66	6.24E-07	2.31E-06
3151	1989	HMGN2	-0.66	9.14E-27	1.13E-25
90529	2771	STPG1	-0.66	5.19E-05	0.000153636
10531	3508	PITRM1	-0.66	1.52E-13	9.52E-13
5737	5260	PTGFR	-0.66	8.50E-06	2.78E-05
23097	6090	CDK19	-0.66	3.36E-13	2.05E-12
6018	6137	RLF	-0.66	3.48E-12	1.98E-11
55166	1759	CENPQ	-0.66	2.46E-05	7.61E-05
1912	2566	PHC2	-0.66	1.43E-22	1.45E-21
10062	1574	NR1H3	-0.66	0.000692616	0.001734762
11212	2576	PLPBP	-0.66	1.07E-08	4.70E-08
83538	2468	TTC25	-0.66	0.006501312	0.013683407
284069	2679	FAM171A2	-0.66	7.83E-08	3.17E-07
7976	6098	FZD3	-0.66	0.003780671	0.008308592

84668	6442	FAM126A	-0.66	3.08E-17	2.44E-16
1159	1563	CKMT1B	-0.66	0.006876944	0.014390477
53944	8163	CSNK1G1	-0.66	3.93E-11	2.10E-10
4351	2079	MPI	-0.66	1.40E-13	8.82E-13
93664	4935	CADPS2	-0.66	2.77E-05	8.49E-05
8623	2161	ASMTL	-0.66	5.16E-07	1.92E-06
10116	6805	FEM1B	-0.66	1.66E-15	1.18E-14
27340	9029	UTP20	-0.66	4.16E-12	2.36E-11
8795	4112	TNFRSF10B	-0.66	2.23E-16	1.68E-15
29914	3790	UBIAD1	-0.66	2.80E-10	1.39E-09
51545	1247	ZNF581	-0.66	2.39E-08	1.02E-07
1.01E+08	5155	ZNF891	-0.66	0.022510454	0.042360278
993	3767	CDC25A	-0.66	1.80E-08	7.72E-08
9262	5129	STK17B	-0.66	8.22E-11	4.27E-10
124454	3987	EARS2	-0.67	3.75E-09	1.70E-08
11187	2843	PKP3	-0.67	0.015887993	0.030950319
162972	4122	ZNF550	-0.67	3.70E-05	0.000111563
54964	2055	C1orf56	-0.67	0.001748302	0.004075013
4277	2450	MICB	-0.67	2.13E-10	1.07E-09
1810	3222	DR1	-0.67	3.91E-12	2.22E-11
89874	2653	SLC25A21	-0.67	0.020503652	0.03895102
22835	4411	ZFP30	-0.67	2.88E-05	8.79E-05
3161	3125	HMMR	-0.67	1.15E-17	9.26E-17
284004	1956	HEXDC	-0.67	1.20E-05	3.87E-05
9855	3865	FARP2	-0.67	8.67E-06	2.84E-05
80346	1666	REEP4	-0.67	2.78E-08	1.18E-07
162417	2005	NAGS	-0.67	0.02612647	0.048537758
10999	2990	SLC27A4	-0.67	1.15E-13	7.28E-13
51496	5002	CTDSPL2	-0.67	8.34E-18	6.77E-17
221960	1854	CCZ1B	-0.67	9.18E-13	5.44E-12
10447	2552	FAM3C	-0.67	3.30E-21	3.16E-20
1267	2866	CNP	-0.67	2.69E-22	2.71E-21
55746	4170	NUP133	-0.67	2.57E-14	1.72E-13
342371	7886	ATXN1L	-0.67	1.28E-13	8.08E-13
5511	2325	PPP1R8	-0.67	1.07E-15	7.74E-15
8454	3184	CUL1	-0.67	1.02E-16	7.79E-16
284273	7716	ZADH2	-0.67	1.48E-13	9.31E-13
80714	1560	PBX4	-0.67	0.014664113	0.02876802
2013	5220	EMP2	-0.68	1.66E-21	1.61E-20
11149	5506	BVES	-0.68	1.02E-06	3.68E-06
89839	1045	ARHGAP11B	-0.68	0.008335103	0.01715687
4953	2113	ODC1	-0.68	4.34E-19	3.75E-18
29979	4137	UBQLN1	-0.68	7.45E-24	8.04E-23
9572	2793	NR1D1	-0.68	2.43E-05	7.52E-05
6386	2223	SDCBP	-0.68	2.79E-16	2.08E-15
54784	2106	ALKBH4	-0.68	9.72E-05	0.000276801
221150	2826	SKA3	-0.68	2.75E-10	1.37E-09
55247	2402	NEIL3	-0.68	2.46E-07	9.46E-07

729533	2145	FAM72A	-0.68	7.22E-06	2.39E-05
2736	6646	GLI2	-0.68	5.57E-05	0.000163839
22846	5355	VASH1	-0.68	6.56E-11	3.44E-10
94107	1576	TMEM203	-0.68	1.12E-11	6.16E-11
81839	8691	VANGL1	-0.68	7.86E-17	6.07E-16
9925	4715	ZBTB5	-0.68	2.40E-09	1.11E-08
6449	2358	SGTA	-0.68	5.96E-16	4.37E-15
10276	3944	NET1	-0.68	2.38E-13	1.47E-12
94239	2182	H2AFV	-0.68	5.86E-26	6.96E-25
23641	1470	LDOC1	-0.68	1.34E-07	5.30E-07
10901	1048	DHRS4	-0.68	2.45E-06	8.50E-06
29946	1433	SERTAD3	-0.68	2.79E-07	1.07E-06
7092	7284	TLL1	-0.68	0.000418391	0.001085727
23180	2953	RFTN1	-0.68	2.02E-12	1.17E-11
1894	4205	ECT2	-0.68	1.10E-21	1.08E-20
5824	3722	PEX19	-0.68	9.20E-16	6.67E-15
55691	4809	FRMD4A	-0.68	3.71E-12	2.11E-11
147040	3081	KCTD11	-0.68	2.98E-10	1.47E-09
6320	1423	CLEC11A	-0.69	0.003982183	0.008710505
9584	3417	RBM39	-0.69	2.68E-16	2.00E-15
54662	3919	TBC1D13	-0.69	1.82E-13	1.14E-12
84918	3603	LRP11	-0.69	4.39E-17	3.44E-16
55010	2601	PARPBP	-0.69	1.14E-07	4.54E-07
339500	8565	ZNF678	-0.69	7.69E-06	2.54E-05
64207	4213	IRF2BPL	-0.69	5.79E-12	3.25E-11
51029	3955	DESI2	-0.69	8.64E-15	5.91E-14
140707	1272	BRI3BP	-0.69	4.02E-05	0.000120772
55527	3844	FEM1A	-0.69	2.48E-10	1.24E-09
1.03E+08	1599	LOC102724023	-0.69	3.17E-13	1.94E-12
23144	3331	ZC3H3	-0.69	1.04E-09	4.95E-09
8681	3379	JMJD7-PLA2G4B	-0.69	0.006734563	0.014127474
84553	5676	FAXC	-0.69	0.000467018	0.001202432
9530	4478	BAG4	-0.69	1.45E-07	5.73E-07
64789	2084	EXO5	-0.69	3.29E-05	9.97E-05
89876	4372	MAATS1	-0.69	0.003621272	0.007984954
1948	4439	EFNB2	-0.69	3.67E-16	2.72E-15
5501	2489	PPP1CC	-0.69	1.96E-25	2.27E-24
2547	2252	XRCC6	-0.69	7.20E-25	8.12E-24
5814	9098	PURB	-0.69	6.83E-20	6.17E-19
409	1795	ARRB2	-0.69	6.63E-11	3.48E-10
57539	6188	WDR35	-0.69	4.62E-10	2.25E-09
79008	1170	SLX1B	-0.69	1.59E-07	6.25E-07
548593	1170	SLX1A	-0.69	1.59E-07	6.25E-07
1969	3955	EPHA2	-0.69	2.09E-22	2.11E-21
283991	1604	UBALD2	-0.69	2.10E-10	1.05E-09
114898	1706	C1QTNF2	-0.70	0.004589813	0.00994898
4998	3309	ORC1	-0.70	1.08E-08	4.73E-08
84899	3551	TMTC4	-0.70	3.61E-09	1.64E-08

2289	3828	FKBP5	-0.70	2.31E-29	3.20E-28
55835	5089	CENPJ	-0.70	1.04E-10	5.37E-10
342357	7271	ZKSCAN2	-0.70	6.42E-07	2.36E-06
64428	2162	NARFL	-0.70	5.01E-08	2.07E-07
8531	1870	YBX3	-0.70	2.02E-21	1.95E-20
79796	4004	ALG9	-0.70	2.17E-10	1.09E-09
51606	2077	ATP6V1H	-0.70	1.81E-13	1.13E-12
84726	11062	PRRC2B	-0.70	1.49E-21	1.44E-20
54932	1386	EXD3	-0.70	7.27E-05	0.000210607
60509	2597	AGBL5	-0.70	3.21E-15	2.25E-14
152519	5285	NIPAL1	-0.70	0.000979772	0.002391705
2012	2804	EMP1	-0.70	1.52E-19	1.35E-18
285761	3602	DCBLD1	-0.70	1.44E-11	7.87E-11
253832	5213	ZDHHC20	-0.70	2.09E-22	2.11E-21
55367	3036	PIDD1	-0.70	4.04E-16	2.98E-15
259173	3355	ALS2CL	-0.70	4.52E-10	2.21E-09
113130	2599	CDCA5	-0.70	3.67E-16	2.71E-15
6777	4990	STAT5B	-0.70	1.01E-16	7.75E-16
5362	11457	PLXNA2	-0.70	5.97E-06	2.00E-05
25843	3782	MOB4	-0.70	4.58E-11	2.44E-10
1	1766	A1BG	-0.70	0.007806848	0.016155612
65988	3541	ZNF747	-0.70	9.70E-09	4.28E-08
51161	2096	C3orf18	-0.70	3.75E-05	0.000112917
5711	3394	PSMD5	-0.70	3.80E-18	3.14E-17
54454	7991	ATAD2B	-0.70	8.15E-08	3.29E-07
123263	2785	MTFMT	-0.70	1.56E-06	5.53E-06
1111	3205	CHEK1	-0.70	1.25E-12	7.36E-12
3106	1618	HLA-B	-0.70	3.24E-24	3.54E-23
27346	2585	TMEM97	-0.70	1.19E-15	8.60E-15
1659	4250	DHX8	-0.71	1.02E-14	6.91E-14
3796	4038	KIF2A	-0.71	1.78E-16	1.35E-15
201164	2591	PLD6	-0.71	0.005888183	0.012492087
10150	4632	MBNL2	-0.71	3.62E-15	2.53E-14
10228	4444	STX6	-0.71	2.09E-15	1.48E-14
83694	3026	RPS6KL1	-0.71	0.00099245	0.002420902
6156	571	RPL30	-0.71	3.97E-19	3.44E-18
92400	3073	RBM18	-0.71	3.32E-11	1.78E-10
27338	1206	UBE2S	-0.71	1.73E-18	1.45E-17
813	4666	CALU	-0.71	9.53E-30	1.33E-28
9775	1734	EIF4A3	-0.71	1.28E-17	1.03E-16
57523	7512	NYNRIN	-0.71	0.000146289	0.000406324
1062	8308	CENPE	-0.71	3.64E-23	3.80E-22
257160	2557	RNF214	-0.71	8.80E-07	3.20E-06
494470	5542	RNF165	-0.71	1.17E-06	4.20E-06
220359	1980	TIGD3	-0.71	0.01695048	0.032861552
286128	4518	ZFP41	-0.71	1.31E-07	5.19E-07
904	6706	CCNT1	-0.71	3.77E-13	2.29E-12
3614	2400	IMPDH1	-0.71	6.45E-11	3.39E-10

5983	2618	RFC3	-0.71	7.11E-15	4.88E-14
1.01E+08	942	PMF1-BGLAP	-0.71	0.008729463	0.017900184
56606	3167	SLC2A9	-0.71	0.000381282	0.00099581
6382	3223	SDC1	-0.71	1.02E-25	1.20E-24
79901	4211	CYBRD1	-0.71	3.14E-27	3.96E-26
54993	2074	ZSCAN2	-0.71	0.000109185	0.000308524
85460	7962	ZNF518B	-0.72	2.98E-12	1.70E-11
91179	3447	SCARF2	-0.72	1.93E-12	1.12E-11
143884	2633	CWF19L2	-0.72	2.53E-07	9.74E-07
9585	6342	KIF20B	-0.72	1.37E-25	1.60E-24
25946	2254	ZNF385A	-0.72	5.46E-08	2.25E-07
3835	2159	KIF22	-0.72	4.62E-19	3.99E-18
51386	1950	EIF3L	-0.72	2.35E-33	3.74E-32
84823	4644	LMNB2	-0.72	3.16E-29	4.33E-28
22925	6183	PLA2R1	-0.72	1.62E-12	9.44E-12
9638	1796	FEZ1	-0.72	4.81E-10	2.34E-09
55959	6562	SULF2	-0.72	6.19E-11	3.26E-10
643988	2104	FNDC10	-0.72	7.74E-08	3.13E-07
54821	4224	ERCC6L	-0.72	4.64E-08	1.92E-07
90411	4204	MCFD2	-0.72	2.80E-27	3.53E-26
4899	3553	NRF1	-0.72	6.28E-09	2.81E-08
23047	6064	PDS5B	-0.72	9.98E-22	9.77E-21
8407	1419	TAGLN2	-0.72	9.16E-27	1.13E-25
79872	4547	CBLL1	-0.72	1.05E-11	5.80E-11
140685	5110	ZBTB46	-0.72	0.00323023	0.007181523
84083	4375	ZRANB3	-0.72	6.07E-07	2.25E-06
57542	6592	KLHL42	-0.72	1.12E-14	7.59E-14
283337	3879	ZNF740	-0.72	8.48E-14	5.43E-13
6392	1395	SDHD	-0.72	1.92E-17	1.53E-16
27341	5455	RRP7A	-0.72	2.14E-19	1.89E-18
85463	9681	ZC3H12C	-0.72	1.22E-11	6.68E-11
9679	5740	FAM53B	-0.72	2.31E-12	1.33E-11
23401	2222	FRAT2	-0.72	2.08E-05	6.47E-05
1634	2272	DCN	-0.72	1.12E-18	9.47E-18
7008	4403	TEF	-0.73	6.88E-09	3.07E-08
55181	3283	SMG8	-0.73	2.47E-10	1.23E-09
81930	2748	KIF18A	-0.73	9.67E-16	7.00E-15
55146	1437	ZDHHC4	-0.73	2.98E-11	1.60E-10
25959	5104	KANK2	-0.73	1.73E-26	2.10E-25
23397	3196	NCAPH	-0.73	1.11E-14	7.53E-14
83549	2178	UCK1	-0.73	5.14E-19	4.42E-18
23471	2992	TRAM1	-0.73	1.82E-33	2.92E-32
92906	2596	HNRNPLL	-0.73	3.03E-08	1.28E-07
25829	3572	TMEM184B	-0.73	4.94E-22	4.90E-21
8660	7014	IRS2	-0.73	1.42E-20	1.32E-19
7272	3135	TTK	-0.73	1.22E-16	9.34E-16
10785	2049	WDR4	-0.73	7.71E-06	2.54E-05
374887	1487	YJEFN3	-0.73	0.002785902	0.006257098

283455	13533	KSR2	-0.73	0.019982481	0.038062684
8882	2181	ZPR1	-0.73	1.31E-08	5.71E-08
55007	2357	FAM118A	-0.73	4.14E-08	1.72E-07
3709	12599	ITPR2	-0.73	3.14E-18	2.61E-17
55823	2931	VPS11	-0.73	1.02E-14	6.94E-14
91801	3962	ALKBH8	-0.73	7.74E-06	2.55E-05
6604	1712	SMARCD3	-0.73	9.71E-11	5.02E-10
4673	3029	NAP1L1	-0.73	6.81E-40	1.35E-38
9429	4074	ABCG2	-0.73	5.63E-05	0.000165275
23171	3917	GPD1L	-0.73	4.14E-08	1.72E-07
57565	4259	KLHL14	-0.73	7.85E-07	2.87E-06
123169	2204	LEO1	-0.74	4.83E-13	2.91E-12
9917	5854	FAM20B	-0.74	1.02E-23	1.09E-22
2733	3361	GLE1	-0.74	2.55E-18	2.12E-17
57484	9267	RNF150	-0.74	2.99E-08	1.26E-07
54955	2630	C1orf109	-0.74	2.69E-08	1.14E-07
84876	1496	ORAI1	-0.74	1.92E-08	8.24E-08
22873	4841	DZIP1	-0.74	1.80E-20	1.66E-19
2817	3665	GPC1	-0.74	8.25E-27	1.02E-25
5537	4192	PPP6C	-0.74	3.20E-19	2.79E-18
9807	4366	IP6K1	-0.74	3.50E-20	3.19E-19
30837	8050	SOCS7	-0.74	1.22E-10	6.26E-10
55603	5617	FAM46A	-0.74	3.24E-11	1.74E-10
80119	2664	PIF1	-0.74	2.37E-07	9.15E-07
10795	5654	ZNF268	-0.74	5.81E-12	3.26E-11
147841	1892	SPC24	-0.74	1.53E-13	9.63E-13
9975	5076	NR1D2	-0.74	5.48E-21	5.19E-20
2043	6419	EPHA4	-0.74	3.06E-10	1.51E-09
643641	7655	ZNF862	-0.74	3.42E-07	1.30E-06
8372	1511	HYAL3	-0.74	0.000306084	0.000809422
22850	5206	ADNP2	-0.74	2.81E-17	2.23E-16
157922	7435	CAMSAP1	-0.74	6.57E-22	6.48E-21
283571	5116	PROX2	-0.74	0.011338581	0.022707835
8459	2385	TPST2	-0.75	1.42E-06	5.06E-06
79594	2492	MUL1	-0.75	2.38E-13	1.47E-12
2261	4168	FGFR3	-0.75	4.69E-11	2.49E-10
57508	5906	INTS2	-0.75	1.06E-07	4.23E-07
2778	1569	GNAS	-0.75	8.09E-29	1.09E-27
53339	3305	BTBD1	-0.75	2.84E-21	2.73E-20
4200	4635	ME2	-0.75	1.24E-16	9.46E-16
23169	6321	SLC35D1	-0.75	2.09E-08	8.94E-08
7561	2967	ZNF14	-0.75	0.001122123	0.00271153
5734	3181	PTGER4	-0.75	0.000349331	0.000916691
9695	6175	EDEM1	-0.75	3.61E-18	2.99E-17
10575	2352	CCT4	-0.75	1.48E-23	1.58E-22
10106	4852	CTDSP2	-0.75	9.80E-33	1.53E-31
79065	3890	ATG9A	-0.75	1.04E-18	8.85E-18
25777	3985	SUN2	-0.75	5.20E-27	6.50E-26

7037	4981	TFRC	-0.75	3.45E-22	3.46E-21
11264	5693	PXMP4	-0.75	8.00E-08	3.24E-07
9793	6727	CKAP5	-0.75	3.90E-31	5.77E-30
160518	9380	DENND5B	-0.75	4.30E-14	2.83E-13
11135	2181	CDC42EP1	-0.75	1.52E-14	1.02E-13
54471	5772	MIEF1	-0.75	1.06E-16	8.10E-16
221830	3892	TWISTNB	-0.75	4.59E-19	3.96E-18
29843	4573	SENP1	-0.75	1.18E-15	8.52E-15
283987	3369	HID1	-0.75	0.000348305	0.000914404
994	2996	CDC25B	-0.75	6.99E-25	7.90E-24
6256	5546	RXRA	-0.75	1.22E-26	1.49E-25
23361	6094	ZNF629	-0.75	7.53E-19	6.44E-18
64895	4656	PAPOLG	-0.75	5.65E-09	2.53E-08
56107	4605	PCDHGA9	-0.75	0.027011185	0.04999841
983	1912	CDK1	-0.75	1.41E-23	1.50E-22
54910	3487	SEMA4C	-0.75	2.31E-12	1.33E-11
3674	3449	ITGA2B	-0.75	0.001976708	0.004563689
64946	1405	CENPH	-0.75	3.22E-13	1.97E-12
5524	2634	PTPA	-0.75	9.33E-22	9.15E-21
53947	2084	A4GALT	-0.76	1.34E-24	1.50E-23
7019	3810	TFAM	-0.76	3.59E-17	2.83E-16
2553	2643	GABPB1	-0.76	4.25E-11	2.26E-10
3676	7281	ITGA4	-0.76	2.69E-13	1.66E-12
8019	5262	BRD3	-0.76	2.16E-29	3.00E-28
23420	4261	NOMO1	-0.76	3.14E-24	3.45E-23
157638	5451	FAM84B	-0.76	1.73E-20	1.60E-19
11182	2497	SLC2A6	-0.76	3.65E-11	1.95E-10
84296	3841	GINS4	-0.76	7.03E-10	3.39E-09
79723	2967	SUV39H2	-0.76	8.06E-11	4.19E-10
10444	4285	ZER1	-0.76	2.03E-20	1.87E-19
3996	4175	LLGL1	-0.76	6.85E-20	6.18E-19
10024	2455	TROAP	-0.76	5.29E-13	3.17E-12
375346	4746	STIMATE	-0.76	5.30E-10	2.58E-09
83999	5935	KREMEN1	-0.76	6.60E-13	3.93E-12
79870	2859	BAALC	-0.76	9.63E-07	3.49E-06
9331	4624	B4GALT6	-0.76	1.43E-09	6.73E-09
27330	8223	RPS6KA6	-0.76	7.17E-05	0.000207708
83886	1404	PRSS27	-0.76	0.015746285	0.030704565
147372	6063	CCBE1	-0.76	2.99E-17	2.37E-16
54494	2019	C11orf71	-0.76	5.25E-05	0.000155044
1975	4062	EIF4B	-0.76	2.21E-37	4.05E-36
25942	6544	SIN3A	-0.76	6.56E-27	8.15E-26
25920	2629	NELFB	-0.76	2.05E-20	1.89E-19
9096	5881	TBX18	-0.77	2.45E-16	1.83E-15
57109	2387	REXO4	-0.77	3.36E-14	2.22E-13
514	449	ATP5F1E	-0.77	5.59E-06	1.87E-05
59	1584	ACTA2	-0.77	1.27E-08	5.54E-08
57189	7296	KIAA1147	-0.77	1.20E-12	7.03E-12

6248	2344	RSC1A1	-0.77	5.07E-06	1.70E-05
60468	9118	BACH2	-0.77	7.74E-07	2.83E-06
10555	1569	AGPAT2	-0.77	4.80E-22	4.77E-21
283989	2053	TSEN54	-0.77	1.14E-09	5.42E-09
252969	2179	NEIL2	-0.77	0.00264541	0.005973267
8971	1524	H1FX	-0.77	3.02E-30	4.31E-29
133022	2037	TRAM1L1	-0.77	0.000363658	0.000951917
3635	5083	INPP5D	-0.77	0.003567382	0.007873942
4077	4597	NBR1	-0.77	8.25E-28	1.07E-26
79633	15175	FAT4	-0.77	5.16E-09	2.32E-08
80335	4143	WDR82	-0.77	4.89E-26	5.82E-25
11173	5472	ADAMTS7	-0.77	1.13E-12	6.65E-12
23543	6864	RBFOX2	-0.77	4.05E-26	4.85E-25
8864	6342	PER2	-0.77	2.21E-07	8.57E-07
54880	6397	BCOR	-0.77	7.21E-18	5.89E-17
9631	4617	NUP155	-0.77	4.91E-20	4.46E-19
406	2772	ARNTL	-0.77	4.88E-09	2.20E-08
5108	8610	PCM1	-0.77	1.64E-25	1.92E-24
6675	2331	UAP1	-0.77	3.19E-15	2.24E-14
64753	4404	CCDC136	-0.77	6.38E-06	2.12E-05
64397	8951	ZNF106	-0.77	5.88E-25	6.68E-24
23660	4444	ZKSCAN5	-0.77	3.10E-13	1.90E-12
6224	857	RPS20	-0.78	6.26E-20	5.67E-19
54442	2479	KCTD5	-0.78	8.13E-18	6.61E-17
23384	5657	SPECC1L	-0.78	2.22E-21	2.14E-20
55647	1543	RAB20	-0.78	1.87E-05	5.88E-05
6418	2836	SET	-0.78	3.17E-37	5.76E-36
55357	2936	TBC1D2	-0.78	2.60E-25	2.99E-24
196264	3986	MPZL3	-0.78	3.67E-05	0.000110676
80818	4347	ZNF436	-0.78	1.04E-08	4.56E-08
55194	1031	EVA1B	-0.78	2.97E-11	1.59E-10
7186	2472	TRAF2	-0.78	2.71E-16	2.02E-15
6804	2067	STX1A	-0.78	0.000429737	0.001113218
643853	3470	TMPPE	-0.78	0.026453423	0.049085476
2232	1881	FDXR	-0.78	1.09E-10	5.64E-10
10391	3386	CORO2B	-0.78	1.19E-06	4.28E-06
140809	2704	SRXN1	-0.78	2.90E-13	1.78E-12
9655	4494	SOCS5	-0.78	1.13E-11	6.24E-11
9697	6997	TRAM2	-0.78	6.07E-31	8.91E-30
7716	4656	VEZF1	-0.78	8.05E-25	9.04E-24
3146	4274	HMGB1	-0.79	4.64E-41	9.60E-40
7157	2591	TP53	-0.79	4.92E-26	5.85E-25
6603	2422	SMARCD2	-0.79	3.41E-19	2.97E-18
348487	1732	FAM131C	-0.79	0.00394905	0.00864335
140831	2831	ZSWIM3	-0.79	0.00075389	0.001877381
1063	10315	CENPF	-0.79	5.95E-37	1.07E-35
11123	2738	RCAN3	-0.79	2.11E-09	9.80E-09
7273	82029	TTN	-0.79	2.28E-05	7.08E-05

4603	4972	MYBL1	-0.79	7.41E-14	4.77E-13
50937	8213	CDON	-0.79	0.000511499	0.001307858
23347	8645	SMCHD1	-0.79	5.00E-31	7.37E-30
11156	2786	PTP4A3	-0.79	0.000170381	0.000468048
57541	5328	ZNF398	-0.79	2.80E-09	1.29E-08
5768	3199	QSOX1	-0.79	2.12E-37	3.89E-36
5770	3573	PTPN1	-0.79	6.82E-35	1.15E-33
27347	3278	STK39	-0.79	3.14E-23	3.29E-22
83591	4840	THAP2	-0.79	0.005362678	0.011471715
286046	3384	XKR6	-0.79	0.017780244	0.034305503
20	8166	ABCA2	-0.79	1.26E-18	1.06E-17
10291	5159	SF3A1	-0.80	1.47E-24	1.64E-23
6508	3571	SLC4A3	-0.80	1.21E-08	5.29E-08
1104	2624	RCC1	-0.80	1.55E-26	1.89E-25
22869	6556	ZNF510	-0.80	9.31E-10	4.43E-09
10587	2122	TXNRD2	-0.80	8.07E-12	4.49E-11
79029	2505	SPATA5L1	-0.80	9.97E-06	3.23E-05
146909	4098	KIF18B	-0.80	4.24E-13	2.56E-12
10565	7199	ARFGEF1	-0.80	1.19E-19	1.06E-18
4927	3521	NUP88	-0.80	3.62E-20	3.29E-19
137695	2518	TMEM68	-0.80	3.61E-10	1.78E-09
11190	7602	CEP250	-0.80	1.60E-25	1.87E-24
6834	1046	SURF1	-0.80	8.76E-11	4.54E-10
3718	5204	JAK3	-0.80	0.00094457	0.002313954
201299	971	RDM1	-0.80	0.013591734	0.026815226
162239	3403	ZFP1	-0.80	7.74E-10	3.71E-09
83658	723	DYNLRB1	-0.80	3.61E-21	3.44E-20
23177	5566	CEP68	-0.80	4.07E-11	2.17E-10
9538	2283	EI24	-0.80	3.82E-22	3.82E-21
150275	3784	CCDC117	-0.80	5.07E-15	3.51E-14
1.01E+08	1506	MICA	-0.80	3.55E-10	1.75E-09
79966	2618	SCD5	-0.80	7.48E-18	6.10E-17
1808	4513	DPYSL2	-0.80	2.90E-26	3.50E-25
6722	3739	SRF	-0.80	2.70E-18	2.25E-17
400720	7556	ZNF772	-0.80	1.77E-07	6.92E-07
8939	3164	FUBP3	-0.80	1.48E-31	2.22E-30
332	2650	BIRC5	-0.80	2.80E-24	3.09E-23
6744	4076	SSFA2	-0.80	2.91E-28	3.84E-27
4148	2600	MATN3	-0.80	2.26E-10	1.13E-09
3159	2029	HMGA1	-0.80	0.000119914	0.000336379
10370	1936	CITED2	-0.80	8.79E-28	1.14E-26
10051	5084	SMC4	-0.80	3.76E-42	8.09E-41
128346	6415	C1orf162	-0.80	0.004342752	0.009439934
27239	1846	GPR162	-0.80	5.50E-06	1.85E-05
81605	2710	URM1	-0.81	2.47E-20	2.26E-19
25928	1883	SOSTDC1	-0.81	0.002731668	0.006147724
84945	5316	ABHD13	-0.81	1.37E-12	8.00E-12
89891	1742	WDR34	-0.81	6.54E-28	8.51E-27

341	588	APOC1	-0.81	0.000594657	0.001505968
891	2177	CCNB1	-0.81	9.18E-39	1.76E-37
23203	2199	PMPCA	-0.81	3.06E-21	2.93E-20
56957	6360	OTUD7B	-0.81	2.30E-17	1.83E-16
84256	4141	FLYWCH1	-0.81	8.11E-20	7.29E-19
126282	3850	TNFAIP8L1	-0.81	7.27E-13	4.33E-12
81544	3370	GDPD5	-0.81	3.48E-11	1.87E-10
399687	7588	MYO18A	-0.81	2.92E-24	3.21E-23
6586	9565	SLIT3	-0.81	1.98E-05	6.19E-05
10893	4249	MMP24	-0.81	4.42E-24	4.80E-23
1.08E+08	3304	LOC107987428	-0.81	0.000152714	0.000422915
8722	1909	CTSF	-0.81	1.32E-23	1.41E-22
9410	1638	SNRNP40	-0.81	6.58E-18	5.38E-17
7291	1593	TWIST1	-0.81	2.00E-08	8.58E-08
5955	2213	RCN2	-0.81	8.32E-26	9.81E-25
23654	6361	PLXNB2	-0.81	3.44E-36	6.04E-35
79814	3154	AGMAT	-0.82	0.021780054	0.041120815
10671	1126	DCTN6	-0.82	3.38E-13	2.07E-12
9743	10048	ARHGAP32	-0.82	1.13E-08	4.95E-08
64784	5253	CRTC3	-0.82	1.32E-17	1.07E-16
80352	1986	RNF39	-0.82	0.019382151	0.037042278
51281	2977	ANKMY1	-0.82	8.54E-05	0.000244726
10570	2732	DPYSL4	-0.82	1.60E-06	5.65E-06
10245	988	TIMM17B	-0.82	1.67E-10	8.44E-10
6191	956	RPS4X	-0.82	4.97E-33	7.82E-32
404550	899	C16orf74	-0.82	0.000475126	0.001221184
23404	2229	EXOSC2	-0.82	8.54E-15	5.84E-14
80757	1538	TMEM121	-0.82	0.001160457	0.002796556
55672	6183	NBPF1	-0.82	3.99E-14	2.63E-13
51762	4859	RAB8B	-0.82	4.34E-17	3.40E-16
84695	2949	LOXL3	-0.82	4.80E-05	0.000142675
10103	1589	TSPAN1	-0.82	8.12E-10	3.89E-09
4775	6379	NFATC3	-0.82	6.73E-22	6.64E-21
3745	11856	KCNB1	-0.82	0.00117849	0.002836549
6130	890	RPL7A	-0.82	4.86E-23	5.06E-22
3203	802	HOXA6	-0.82	0.000461977	0.001190661
80055	10945	PGAP1	-0.82	2.85E-13	1.75E-12
54704	4257	PDP1	-0.83	1.68E-22	1.71E-21
9647	5170	PPM1F	-0.83	1.17E-16	8.92E-16
50810	2528	HDGFL3	-0.83	1.74E-23	1.85E-22
5923	4507	RASGRF1	-0.83	0.01102439	0.022148503
726	4398	CAPN5	-0.83	4.91E-16	3.61E-15
38	1533	ACAT1	-0.83	1.35E-15	9.72E-15
23221	5110	RHOBTB2	-0.83	1.30E-12	7.61E-12
64762	6574	GAREM1	-0.83	5.35E-09	2.40E-08
11278	10853	KLF12	-0.83	5.29E-13	3.17E-12
80727	4795	TTYH3	-0.83	9.93E-29	1.33E-27
64129	2095	TINAGL1	-0.83	9.95E-26	1.17E-24

440590	3776	ZYG11A	-0.83	1.63E-06	5.75E-06
134	2822	ADORA1	-0.83	5.30E-17	4.14E-16
6671	5885	SP4	-0.83	1.09E-07	4.34E-07
55699	3549	IARS2	-0.83	6.80E-23	7.03E-22
55196	6371	KIAA1551	-0.83	3.79E-12	2.15E-11
55224	2432	ETNK2	-0.83	8.64E-14	5.53E-13
41	3860	ASIC1	-0.83	1.39E-09	6.52E-09
85474	1463	LBX2	-0.83	0.003748671	0.008245405
4311	5692	MME	-0.83	5.02E-30	7.09E-29
23048	5703	FNBP1	-0.84	1.40E-24	1.56E-23
57418	1566	WDR18	-0.84	1.56E-19	1.38E-18
10615	3824	SPAG5	-0.84	7.32E-28	9.47E-27
23365	8971	ARHGEF12	-0.84	7.79E-38	1.45E-36
79980	2231	DSN1	-0.84	1.96E-25	2.27E-24
3832	5101	KIF11	-0.84	5.55E-33	8.70E-32
224	3640	ALDH3A2	-0.84	3.12E-24	3.43E-23
79906	1556	MORN1	-0.84	0.000151475	0.000420006
27289	1665	RND1	-0.84	0.005623208	0.011977123
162979	1604	ZNF296	-0.84	0.000442842	0.001145664
54149	5451	C21orf91	-0.84	2.29E-10	1.14E-09
2872	3090	MKNK2	-0.84	8.66E-33	1.35E-31
54935	740	DUSP23	-0.84	1.87E-16	1.41E-15
5966	7529	REL	-0.84	2.74E-08	1.16E-07
5962	4232	RDX	-0.84	8.23E-27	1.02E-25
81628	2369	TSC22D4	-0.84	3.02E-16	2.25E-15
9827	1717	RGP1	-0.84	1.55E-06	5.49E-06
50484	4883	RRM2B	-0.84	1.40E-17	1.13E-16
1464	8305	CSPG4	-0.84	8.31E-17	6.40E-16
55248	1946	TMEM206	-0.84	1.30E-07	5.14E-07
283248	2621	RCOR2	-0.84	0.005005786	0.010762682
114879	3898	OSBPL5	-0.84	1.14E-15	8.26E-15
1654	4717	DDX3X	-0.85	1.39E-42	3.03E-41
27034	2411	ACAD8	-0.85	1.50E-13	9.45E-13
56106	4617	PCDHGA10	-0.85	0.01840377	0.035354977
3092	7956	HIP1	-0.85	4.70E-22	4.68E-21
114984	1050	FLYWCH2	-0.85	4.97E-11	2.63E-10
160760	3748	PPTC7	-0.85	4.38E-14	2.88E-13
6495	2685	SIX1	-0.85	4.40E-11	2.34E-10
650	3191	BMP2	-0.85	0.00041935	0.001088057
2581	3897	GALC	-0.85	2.01E-12	1.17E-11
6241	3285	RRM2	-0.85	2.77E-33	4.40E-32
84159	7022	ARID5B	-0.85	1.11E-25	1.30E-24
124930	3138	ANKRD13B	-0.85	1.61E-07	6.32E-07
1.02E+08	1968	LOC101929895	-0.85	0.002256952	0.005157751
3383	3249	ICAM1	-0.85	6.04E-35	1.02E-33
627	4120	BDNF	-0.85	4.17E-19	3.60E-18
91461	2517	PKDCC	-0.85	0.019856886	0.037851857
84675	2527	TRIM55	-0.85	3.15E-10	1.56E-09

109	4031	ADCY3	-0.85	8.57E-24	9.24E-23
29902	1226	FAM216A	-0.85	2.18E-09	1.01E-08
51201	4923	ZDHHC2	-0.86	7.02E-15	4.82E-14
8862	3238	APLN	-0.86	0.000736766	0.00183757
7543	7291	ZFX	-0.86	1.33E-22	1.36E-21
79595	4055	SAP130	-0.86	2.03E-23	2.14E-22
10422	1877	UBAC1	-0.86	6.45E-20	5.84E-19
80271	3319	ITPKC	-0.86	1.21E-10	6.19E-10
1825	7676	DSC3	-0.86	2.83E-07	1.08E-06
26061	2233	HACL1	-0.86	1.49E-15	1.07E-14
648	3435	BMI1	-0.86	3.01E-08	1.27E-07
22934	1834	RPIA	-0.86	3.57E-11	1.91E-10
4493	520	MT1E	-0.86	6.11E-29	8.28E-28
79734	1747	KCTD17	-0.86	3.56E-15	2.49E-14
84971	1938	ATG4D	-0.86	2.43E-10	1.21E-09
6423	2005	SFRP2	-0.86	1.40E-10	7.15E-10
25792	2883	CIZ1	-0.86	1.11E-29	1.55E-28
440689	495	HIST2H2BF	-0.86	0.013370964	0.026403122
661	1946	POLR3D	-0.86	1.77E-05	5.57E-05
2288	3535	FKBP4	-0.86	8.70E-26	1.03E-24
9824	5802	ARHGAP11A	-0.86	6.57E-31	9.62E-30
5476	3065	CTSA	-0.86	1.76E-34	2.91E-33
200933	5743	FBXO45	-0.86	2.93E-16	2.18E-15
59283	8751	CACNG8	-0.86	1.31E-06	4.69E-06
23200	8182	ATP11B	-0.86	2.06E-24	2.28E-23
1955	6227	MEGF9	-0.86	7.11E-24	7.68E-23
83480	1520	PUS3	-0.86	3.46E-06	1.18E-05
10712	3093	FAM189B	-0.86	1.85E-12	1.08E-11
56892	1841	TCIM	-0.86	4.62E-08	1.91E-07
203	2263	AK1	-0.86	6.64E-23	6.87E-22
64748	2730	PLPPR2	-0.86	3.11E-19	2.72E-18
641	3229	BLM	-0.86	5.43E-14	3.54E-13
541468	1863	LURAP1	-0.86	0.000256582	0.000686691
25888	4773	ZNF473	-0.86	6.10E-14	3.96E-13
7410	4735	VAV2	-0.86	2.58E-30	3.70E-29
92595	2814	ZNF764	-0.86	3.74E-07	1.42E-06
22824	3905	HSPA4L	-0.86	3.15E-14	2.09E-13
8436	3265	CAVIN2	-0.87	1.06E-05	3.44E-05
9649	7512	RALGPS1	-0.87	6.75E-11	3.54E-10
8242	5547	KDM5C	-0.87	2.26E-34	3.73E-33
89927	2236	C16orf45	-0.87	1.91E-09	8.90E-09
6752	2996	SSTR2	-0.87	0.019416963	0.037100827
9055	3027	PRC1	-0.87	1.20E-40	2.44E-39
23322	5765	RPGRIP1L	-0.87	3.96E-12	2.24E-11
57758	4377	SCUBE2	-0.87	2.05E-08	8.79E-08
157	9068	GRK3	-0.87	0.001259002	0.003012382
56905	4443	C15orf39	-0.87	5.29E-12	2.98E-11
2036	5928	EPB41L1	-0.87	2.43E-25	2.80E-24

4784	5578	NFIX	-0.87	6.19E-17	4.81E-16
317781	4738	DDX51	-0.87	3.03E-15	2.13E-14
399979	5548	SNX19	-0.87	1.63E-21	1.58E-20
9650	2698	MTFR1	-0.87	1.19E-11	6.55E-11
2304	3473	FOXE1	-0.87	3.01E-09	1.38E-08
5681	3505	PSKH1	-0.87	2.04E-16	1.54E-15
10400	1007	PEMT	-0.87	1.66E-13	1.04E-12
81502	1636	HM13	-0.87	1.24E-40	2.52E-39
334	3620	APLP2	-0.88	6.05E-57	1.98E-55
5150	3242	PDE7A	-0.88	2.21E-13	1.37E-12
6617	2684	SNAPC1	-0.88	6.41E-21	6.06E-20
2919	1184	CXCL1	-0.88	1.12E-38	2.15E-37
7474	5708	WNT5A	-0.88	1.57E-24	1.74E-23
1861	2117	TOR1A	-0.88	8.69E-30	1.22E-28
285368	3804	PRRT3	-0.88	1.45E-10	7.39E-10
7832	2718	BTG2	-0.88	1.77E-09	8.28E-09
54762	3710	GRAMD1C	-0.88	3.78E-08	1.58E-07
23205	3428	ACSBG1	-0.88	0.002745692	0.00617381
7336	1810	UBE2V2	-0.88	1.70E-25	1.98E-24
116987	10475	AGAP1	-0.88	1.24E-24	1.39E-23
5743	4507	PTGS2	-0.88	2.55E-06	8.82E-06
5519	5172	PPP2R1B	-0.88	3.82E-17	3.00E-16
83850	5427	ESYT3	-0.88	0.019624915	0.037461913
23064	7602	SETX	-0.88	1.20E-35	2.07E-34
55167	4092	MSL2	-0.88	2.84E-17	2.25E-16
81873	2107	ARPC5L	-0.88	1.71E-20	1.58E-19
28960	1473	DCPS	-0.88	3.64E-10	1.79E-09
9919	8445	SEC16A	-0.88	4.29E-42	9.17E-41
64782	3136	AEN	-0.89	2.75E-14	1.83E-13
79746	1674	ECHDC3	-0.89	7.01E-05	0.000203259
50624	2279	CUZD1	-0.89	0.016822971	0.032635719
9700	6635	ESPL1	-0.89	1.33E-22	1.36E-21
8425	5084	LTBP4	-0.89	7.84E-31	1.15E-29
54443	4780	ANLN	-0.89	1.10E-41	2.33E-40
57710	4155	KIAA1614	-0.89	7.22E-05	0.000209131
3234	1954	HOXD8	-0.89	1.06E-11	5.85E-11
9674	4592	KIAA0040	-0.89	1.00E-11	5.54E-11
3732	1855	CD82	-0.89	7.03E-23	7.25E-22
57573	6162	ZNF471	-0.89	0.000167402	0.000460576
51116	1516	MRPS2	-0.89	3.38E-19	2.94E-18
5738	6113	PTGFRN	-0.89	4.08E-47	1.00E-45
65251	3234	ZNF649	-0.89	1.90E-07	7.41E-07
10594	7311	PRPF8	-0.90	3.98E-42	8.54E-41
728116	4019	ZBTB8B	-0.90	0.012279561	0.024421675
8829	5597	NRP1	-0.90	1.13E-44	2.63E-43
81618	2100	ITM2C	-0.90	5.58E-37	1.00E-35
11092	1965	SPACA9	-0.90	1.44E-05	4.59E-05
10609	2634	P3H4	-0.90	1.71E-26	2.07E-25

6526	11623	SLC5A3	-0.90	4.06E-27	5.08E-26
10987	1510	COPS5	-0.90	1.22E-15	8.76E-15
467	2106	ATF3	-0.90	5.80E-08	2.38E-07
78991	2142	PCYOX1L	-0.90	3.48E-12	1.99E-11
157313	5227	CDCA2	-0.90	6.47E-20	5.85E-19
90557	1458	CCDC74A	-0.90	7.90E-11	4.12E-10
10079	7622	ATP9A	-0.90	2.75E-33	4.36E-32
7840	12928	ALMS1	-0.90	1.17E-20	1.09E-19
9481	2156	SLC25A27	-0.90	0.011174921	0.022417974
80110	4676	ZNF614	-0.90	5.61E-11	2.96E-10
55200	2854	PLEKHG6	-0.90	0.011168667	0.022407958
10505	4162	SEMA4F	-0.90	7.50E-13	4.47E-12
127579	2381	DCST2	-0.90	0.022363891	0.042115699
51696	5621	HECA	-0.90	4.82E-15	3.35E-14
2524	3089	FUT2	-0.90	0.007825445	0.01618844
6431	3755	SRSF6	-0.91	1.09E-51	3.05E-50
9054	2385	NFS1	-0.91	1.20E-21	1.17E-20
6273	970	S100A2	-0.91	2.78E-05	8.51E-05
10926	3883	DBF4	-0.91	1.69E-23	1.80E-22
137886	4533	UBXN2B	-0.91	2.83E-18	2.36E-17
57178	7017	ZMIZ1	-0.91	8.73E-27	1.08E-25
3169	3465	FOXA1	-0.91	1.68E-05	5.30E-05
56652	2698	TWNK	-0.91	3.48E-11	1.87E-10
90381	6688	TICRR	-0.91	6.87E-15	4.72E-14
26039	4550	SS18L1	-0.91	3.22E-24	3.53E-23
55216	3250	NKAPD1	-0.91	3.19E-19	2.79E-18
10514	4131	MYBBP1A	-0.91	2.48E-25	2.86E-24
375057	7713	STUM	-0.91	0.014129254	0.027786231
3714	5845	JAG2	-0.91	9.02E-16	6.54E-15
3703	3081	STT3A	-0.91	3.49E-46	8.41E-45
1.08E+08	1904	LOC107985388	-0.91	0.002291858	0.005229451
170689	5309	ADAMTS15	-0.91	2.01E-09	9.36E-09
8601	1790	RGS20	-0.91	6.48E-09	2.89E-08
5580	2632	PRKCD	-0.91	3.39E-19	2.95E-18
114789	3399	SLC25A25	-0.91	3.72E-17	2.93E-16
138151	6899	NACC2	-0.91	3.80E-28	4.98E-27
57646	3965	USP28	-0.91	8.15E-18	6.63E-17
80024	3401	SLC8B1	-0.91	4.42E-16	3.25E-15
8243	9797	SMC1A	-0.91	2.78E-49	7.30E-48
10383	1591	TUBB4B	-0.91	1.01E-35	1.74E-34
10395	5946	DLC1	-0.91	5.79E-22	5.72E-21
55143	2468	CDCA8	-0.91	2.79E-28	3.67E-27
7067	2485	THRA	-0.92	1.55E-11	8.42E-11
3667	8743	IRS1	-0.92	5.53E-29	7.51E-28
6001	904	RGS10	-0.92	2.06E-15	1.46E-14
22856	3937	CHSY1	-0.92	3.22E-31	4.77E-30
9377	784	COX5A	-0.92	2.12E-33	3.38E-32
6608	3714	SMO	-0.92	4.55E-18	3.75E-17

219899	5221	TBCEL	-0.92	6.39E-13	3.82E-12
64764	6763	CREB3L2	-0.92	2.59E-29	3.58E-28
340156	5725	MYLK4	-0.92	0.006869177	0.014379304
730094	4445	C16orf52	-0.92	1.45E-13	9.10E-13
9442	1498	MED27	-0.92	1.14E-19	1.02E-18
26190	7340	FBXW2	-0.92	2.28E-28	3.01E-27
96764	3581	TGS1	-0.92	5.75E-19	4.94E-18
2788	4264	GNG7	-0.92	3.39E-06	1.16E-05
90120	2866	TMEM250	-0.92	2.10E-19	1.85E-18
27348	2710	TOR1B	-0.92	6.97E-28	9.05E-27
23212	1724	RRS1	-0.92	3.69E-14	2.43E-13
149420	4082	PDIK1L	-0.92	1.10E-08	4.82E-08
3054	7986	HCFC1	-0.92	6.05E-27	7.53E-26
259266	10008	ASPM	-0.92	6.14E-25	6.97E-24
93594	3336	TBC1D31	-0.92	1.94E-11	1.05E-10
577	5083	ADGRB3	-0.92	0.000406511	0.00105737
7013	2949	TERF1	-0.92	1.81E-22	1.84E-21
374946	2715	DRAXIN	-0.92	0.000811209	0.002006621
10500	4361	SEMA6C	-0.93	6.10E-07	2.26E-06
49854	7220	ZBTB21	-0.93	6.33E-17	4.92E-16
11245	3971	GPR176	-0.93	6.51E-24	7.04E-23
23313	6215	KIAA0930	-0.93	1.17E-28	1.57E-27
5695	1043	PSMB7	-0.93	3.21E-37	5.83E-36
857	2570	CAV1	-0.93	7.57E-37	1.35E-35
79187	1860	FSD1	-0.93	2.02E-13	1.25E-12
8821	8221	INPP4B	-0.93	5.04E-20	4.58E-19
23511	5710	NUP188	-0.93	2.25E-47	5.60E-46
9759	8367	HDAC4	-0.93	1.76E-13	1.10E-12
92126	9281	DSEL	-0.93	2.66E-14	1.77E-13
23060	8582	ZNF609	-0.94	7.64E-25	8.60E-24
1952	10562	CELSR2	-0.94	6.30E-25	7.14E-24
79586	2965	CHPF	-0.94	2.31E-26	2.79E-25
79805	4245	VASH2	-0.94	7.83E-06	2.58E-05
55635	4996	DEPDC1	-0.94	1.65E-31	2.47E-30
1033	906	CDKN3	-0.94	2.87E-17	2.28E-16
84247	5355	RTL6	-0.94	1.24E-23	1.33E-22
51006	2263	SLC35C2	-0.94	4.19E-33	6.61E-32
1849	3248	DUSP7	-0.94	1.66E-15	1.19E-14
339761	4805	CYP27C1	-0.95	3.91E-05	0.000117607
5744	1731	PTHLH	-0.95	0.007522476	0.01562264
9493	3020	KIF23	-0.95	1.10E-34	1.83E-33
284611	5359	FAM102B	-0.95	2.09E-10	1.05E-09
26271	2128	FBXO5	-0.95	2.18E-30	3.14E-29
5083	3122	PAX9	-0.95	0.024856181	0.046371758
81488	4302	POLR2M	-0.95	1.16E-27	1.49E-26
170961	3507	ANKRD24	-0.95	0.006574931	0.013823615
54491	4161	FAM105A	-0.95	2.63E-17	2.09E-16
115811	1822	IQCD	-0.95	3.07E-05	9.35E-05

79845	1864	RNF122	-0.95	0.002763892	0.006211591
1E+08	3757	LOC100289279	-0.95	0.001519928	0.003586337
1152	1476	CKB	-0.95	7.19E-22	7.08E-21
472	9657	ATM	-0.95	7.57E-26	8.94E-25
9787	2994	DLGAP5	-0.96	9.76E-38	1.81E-36
92196	551	DAPL1	-0.96	0.013580254	0.026795552
199800	2112	ADM5	-0.96	0.002733244	0.006149713
10807	2209	SDCCAG3	-0.96	3.87E-26	4.64E-25
79575	2114	ABHD8	-0.96	1.17E-13	7.41E-13
55901	3276	THSD1	-0.96	8.21E-16	5.97E-15
83875	2902	BCO2	-0.96	0.002022888	0.004664246
2342	2836	FNTB	-0.96	7.95E-12	4.43E-11
127544	2844	RNF19B	-0.96	9.31E-14	5.95E-13
4173	4808	MCM4	-0.96	3.07E-55	9.51E-54
51230	5783	PHF20	-0.96	1.27E-44	2.95E-43
115509	3565	ZNF689	-0.96	1.82E-12	1.06E-11
55174	2546	INTS10	-0.96	3.19E-15	2.24E-14
10868	4430	USP20	-0.96	8.90E-18	7.23E-17
54478	1565	PIMREG	-0.96	4.67E-25	5.34E-24
79000	2178	AUNIP	-0.96	9.92E-07	3.59E-06
53346	2360	TM6SF1	-0.96	1.21E-07	4.82E-07
84913	5873	ATOH8	-0.96	8.08E-11	4.20E-10
23395	4197	LARS2	-0.96	8.93E-21	8.40E-20
6917	2778	TCEA1	-0.96	3.47E-34	5.67E-33
79937	5229	CNTNAP3	-0.96	1.92E-09	8.93E-09
55706	4776	NDC1	-0.96	2.66E-30	3.81E-29
647310	2571	TEX22	-0.96	0.026559348	0.049249269
4254	5439	KITLG	-0.97	2.60E-36	4.60E-35
51333	1464	ZNF771	-0.97	2.22E-05	6.89E-05
9738	5448	CCP110	-0.97	1.35E-28	1.80E-27
1490	2358	CTGF	-0.97	3.08E-27	3.88E-26
899	4085	CCNF	-0.97	5.46E-32	8.30E-31
7464	5507	CORO2A	-0.97	4.14E-19	3.58E-18
602	2013	BCL3	-0.97	5.56E-23	5.78E-22
4957	3717	ODF2	-0.97	1.16E-31	1.74E-30
84181	9534	CHD6	-0.97	5.15E-38	9.67E-37
7185	4381	TRAF1	-0.97	6.41E-07	2.36E-06
4751	2144	NEK2	-0.97	1.48E-18	1.24E-17
64410	3683	KLHL25	-0.97	1.91E-06	6.71E-06
144699	1623	FBXL14	-0.97	0.000196559	0.000534998
30815	2447	ST6GALNAC6	-0.97	2.53E-31	3.75E-30
57405	1368	SPC25	-0.97	1.32E-20	1.23E-19
55502	1455	HES6	-0.97	4.59E-07	1.72E-06
51068	2864	NMD3	-0.97	8.43E-23	8.68E-22
79611	3943	ACSS3	-0.97	0.000244668	0.000656587
11174	6186	ADAMTS6	-0.97	5.32E-07	1.98E-06
10389	4143	SCML2	-0.98	4.46E-08	1.85E-07
147949	2257	ZNF583	-0.98	5.25E-05	0.0001552

83540	1923	NUF2	-0.98	7.94E-27	9.82E-26
64840	1843	PORCN	-0.98	5.01E-14	3.28E-13
5564	2403	PRKAB1	-0.98	1.77E-28	2.35E-27
1027	2535	CDKN1B	-0.98	2.42E-36	4.28E-35
64145	6230	RBSN	-0.98	9.34E-23	9.59E-22
51552	4379	RAB14	-0.98	1.80E-34	2.97E-33
8303	3275	SNN	-0.98	7.98E-19	6.80E-18
55969	1138	C20orf24	-0.98	8.84E-30	1.24E-28
4054	4688	LTBP3	-0.98	8.91E-45	2.08E-43
83604	4068	TMEM47	-0.98	3.22E-33	5.10E-32
1032	1184	CDKN2D	-0.98	9.66E-14	6.16E-13
4193	7077	MDM2	-0.98	3.04E-43	6.76E-42
9684	5247	LRRC14	-0.99	1.30E-26	1.59E-25
57727	3222	NCOA5	-0.99	2.51E-37	4.59E-36
57190	4261	SELENON	-0.99	1.34E-39	2.66E-38
142679	5226	DUSP19	-0.99	0.003698238	0.008142296
81575	4752	APOLD1	-0.99	1.95E-13	1.21E-12
64084	10818	CLSTN2	-0.99	1.88E-33	3.00E-32
79957	2670	PAQR6	-0.99	1.50E-13	9.45E-13
25806	1512	VAX2	-0.99	0.000836416	0.002063515
23108	6184	RAP1GAP2	-0.99	1.58E-16	1.19E-15
4774	9336	NFIA	-0.99	6.06E-17	4.72E-16
7994	9210	KAT6A	-0.99	1.98E-25	2.30E-24
55755	5896	CDK5RAP2	-0.99	7.24E-45	1.70E-43
151246	4245	SGO2	-0.99	2.77E-26	3.35E-25
65062	5488	TMEM237	-0.99	6.45E-29	8.75E-28
3233	4053	HOXD4	-1.00	1.98E-23	2.09E-22
1.08E+08	2842	LOC107987464	-1.00	0.002403115	0.005463667
22821	4245	RASA3	-1.00	1.10E-21	1.07E-20
4824	3169	NKX3-1	-1.00	5.99E-08	2.46E-07
8613	3324	PLPP3	-1.00	6.56E-14	4.24E-13
56912	1701	IFT46	-1.00	4.38E-14	2.88E-13
867	10844	CBL	-1.00	2.66E-27	3.37E-26
8894	2210	EIF2S2	-1.00	1.44E-37	2.67E-36
9918	4806	NCAPD2	-1.00	1.30E-51	3.63E-50
525	1956	ATP6V1B1	-1.00	0.01203431	0.023968795
57678	6406	GPAM	-1.00	3.22E-20	2.95E-19
219854	3630	TMEM218	-1.00	8.73E-10	4.17E-09
136288	2149	C7orf57	-1.00	0.008361152	0.017202519
3902	1770	LAG3	-1.00	0.023931942	0.044779143
9354	6125	UBE4A	-1.00	1.16E-22	1.19E-21
2305	3542	FOXM1	-1.00	2.99E-26	3.60E-25
6573	3222	SLC19A1	-1.00	5.91E-20	5.36E-19
1047	2744	CLGN	-1.00	7.79E-23	8.01E-22
6230	514	RPS25	-1.00	1.68E-09	7.84E-09
1.07E+08	3808	BUB1B-PAK6	-1.01	0.000339191	0.000892322
9885	3918	OSBPL2	-1.01	2.80E-34	4.59E-33
10388	5554	SYCP2	-1.01	1.28E-23	1.37E-22

7529	3076	YWHAB	-1.01	7.88E-59	2.68E-57
23762	3604	OSBP2	-1.01	7.68E-10	3.69E-09
11064	6581	CNTRL	-1.01	2.57E-22	2.59E-21
2199	4193	FBLN2	-1.01	9.87E-09	4.34E-08
57568	6695	SIPA1L2	-1.01	9.02E-34	1.46E-32
84085	4354	FBXO30	-1.01	1.25E-24	1.39E-23
8065	5920	CUL5	-1.01	2.48E-27	3.14E-26
2308	5772	FOXO1	-1.01	2.87E-29	3.94E-28
222950	3594	NYAP1	-1.01	0.00471874	0.010202309
144347	2160	RFLNA	-1.01	3.36E-24	3.67E-23
56112	4605	PCDHGA3	-1.01	0.017919797	0.034537254
29780	1713	PARVB	-1.01	1.96E-09	9.13E-09
730005	3393	SEC14L6	-1.01	2.20E-13	1.36E-12
4909	1074	NTF4	-1.01	0.004523026	0.00981499
6513	3687	SLC2A1	-1.02	5.67E-63	2.14E-61
160428	7098	ALDH1L2	-1.02	0.003279571	0.007279379
2637	1795	GBX2	-1.02	0.000397548	0.001035572
256691	3586	MAMDC2	-1.02	8.56E-23	8.80E-22
54768	4180	HYDIN	-1.02	0.000323423	0.000853242
80320	3763	SP6	-1.02	2.10E-05	6.55E-05
55787	4407	TXLNG	-1.02	2.19E-28	2.90E-27
1384	2791	CRAT	-1.02	1.74E-37	3.21E-36
1041	2583	CDSN	-1.02	0.001904814	0.004411461
6838	2326	SURF6	-1.02	1.66E-24	1.84E-23
10420	2941	TESK2	-1.02	3.43E-07	1.30E-06
84928	3537	TMEM209	-1.02	1.18E-28	1.58E-27
57153	3392	SLC44A2	-1.03	5.30E-40	1.06E-38
991	1697	CDC20	-1.03	8.69E-44	1.97E-42
11238	5261	CA5B	-1.03	2.17E-12	1.25E-11
9028	1452	RHBDL1	-1.03	0.000263784	0.000704587
5244	4037	ABCB4	-1.03	5.22E-05	0.000154316
63916	3550	ELMO2	-1.03	4.08E-36	7.14E-35
8324	3851	FZD7	-1.03	1.16E-13	7.35E-13
388610	1968	TRNP1	-1.03	3.65E-15	2.55E-14
353091	3729	RAET1G	-1.03	0.006963709	0.014560036
55070	2296	DET1	-1.03	6.35E-06	2.11E-05
22901	3434	ARSG	-1.03	5.85E-07	2.17E-06
51754	3079	TMEM8B	-1.03	8.85E-15	6.05E-14
8200	2380	GDF5	-1.03	3.12E-06	1.07E-05
11047	1499	ADRM1	-1.03	1.71E-33	2.73E-32
79365	3796	BHLHE41	-1.03	3.93E-40	7.89E-39
83449	3433	PMFBP1	-1.03	0.025121149	0.046841491
57684	2039	ZBTB26	-1.03	4.48E-08	1.86E-07
7296	3914	TXNRD1	-1.03	1.56E-55	4.87E-54
56413	1504	LTB4R2	-1.03	0.000219766	0.000593705
79866	2768	BORA	-1.03	6.96E-14	4.49E-13
9875	10808	URB1	-1.03	6.65E-25	7.52E-24
57636	5682	ARHGAP23	-1.04	8.30E-38	1.54E-36

11004	2877	KIF2C	-1.04	1.01E-42	2.21E-41
55872	2117	PBK	-1.04	3.40E-23	3.55E-22
57513	4804	CASKIN2	-1.04	1.56E-26	1.90E-25
8439	3571	NSMAF	-1.04	1.18E-21	1.15E-20
51512	4109	GTSE1	-1.04	3.39E-42	7.31E-41
124359	8131	CDYL2	-1.04	5.01E-11	2.65E-10
23042	2880	PDXDC1	-1.04	1.79E-17	1.43E-16
4902	1141	NRTN	-1.04	0.009555772	0.019450906
389289	1280	ANXA2R	-1.04	0.006465562	0.013611388
84798	1596	C19orf48	-1.04	9.08E-31	1.32E-29
5342	3016	PLGLB2	-1.04	0.011185436	0.022436536
1058	1419	CENPA	-1.04	1.40E-16	1.07E-15
55175	7410	KLHL11	-1.04	1.37E-17	1.11E-16
958	1601	CD40	-1.04	1.05E-28	1.41E-27
23508	5217	TTC9	-1.04	1.00E-13	6.37E-13
115948	2176	CCDC151	-1.04	0.020874868	0.039571404
57406	2392	ABHD6	-1.04	9.63E-08	3.86E-07
9328	2313	GTF3C5	-1.04	3.47E-45	8.19E-44
84168	4950	ANTXR1	-1.04	2.33E-36	4.13E-35
6734	3100	SRPRA	-1.04	1.12E-40	2.29E-39
970	1187	CD70	-1.04	7.60E-33	1.19E-31
8269	1682	TMEM187	-1.04	5.83E-05	0.000170765
1.07E+08	1450	BOLA2-SMG1P6	-1.05	3.21E-20	2.93E-19
688	3172	KLF5	-1.05	8.39E-33	1.31E-31
1296	4542	COL8A2	-1.05	9.22E-16	6.68E-15
26035	5689	GLCE	-1.05	3.37E-22	3.38E-21
51654	1599	CDK5RAP1	-1.05	4.37E-30	6.20E-29
388341	2634	LRRC75A	-1.05	1.25E-07	4.97E-07
7153	5740	TOP2A	-1.05	1.35E-85	8.24E-84
221935	9053	SDK1	-1.05	2.47E-19	2.17E-18
10137	6716	RBM12	-1.05	5.42E-57	1.77E-55
79034	2127	C7orf26	-1.05	1.90E-17	1.52E-16
4808	2462	NHLH2	-1.05	0.022851106	0.042946737
54436	4336	SH3TC1	-1.05	2.27E-12	1.31E-11
1737	4330	DLAT	-1.06	3.55E-28	4.66E-27
54753	3636	ZNF853	-1.06	2.87E-08	1.21E-07
10194	4911	TSHZ1	-1.06	1.37E-27	1.76E-26
1656	6127	DDX6	-1.06	5.80E-54	1.75E-52
7403	5504	KDM6A	-1.06	1.09E-25	1.28E-24
50506	6347	DUOX2	-1.06	0.023941412	0.044782715
7090	4785	TLE3	-1.06	6.24E-27	7.76E-26
253943	5062	YTHDF3	-1.06	1.52E-45	3.61E-44
23637	4857	RABGAP1	-1.06	9.25E-45	2.16E-43
26586	3645	CKAP2	-1.06	1.96E-46	4.76E-45
440804	5429	RIMBP3B	-1.06	0.014724902	0.028864967
84986	5482	ARHGAP19	-1.06	3.45E-16	2.55E-15
2770	3306	GNAI1	-1.07	6.38E-34	1.04E-32
26958	3154	COPG2	-1.07	5.93E-33	9.29E-32

84078	3008	KBTBD7	-1.07	1.97E-07	7.66E-07
9057	6368	SLC7A6	-1.07	3.13E-30	4.46E-29
2819	3083	GPD1	-1.07	0.017370958	0.033577804
7150	3734	TOP1	-1.07	1.49E-69	6.44E-68
51507	1674	RTF2	-1.07	1.02E-40	2.09E-39
84253	3699	GARNL3	-1.07	4.14E-08	1.72E-07
29068	8746	ZBTB44	-1.07	9.58E-36	1.66E-34
94032	1360	CAMK2N2	-1.07	1.07E-12	6.32E-12
51526	1998	OSER1	-1.07	5.40E-37	9.74E-36
4288	10957	MKI67	-1.07	3.30E-48	8.30E-47
10509	3649	SEMA4B	-1.07	1.94E-50	5.25E-49
57030	2993	SLC17A7	-1.07	0.020668681	0.039218403
201294	4092	UNC13D	-1.07	0.000170601	0.000468579
191	2279	AHCY	-1.08	1.17E-49	3.12E-48
28989	1539	NTMT1	-1.08	9.95E-17	7.64E-16
4293	11071	MAP3K9	-1.08	6.02E-33	9.42E-32
2306	4675	FOXD2	-1.08	0.000607373	0.001534453
23025	9847	UNC13A	-1.08	0.017136821	0.033168522
63935	2711	PCIF1	-1.08	3.25E-35	5.52E-34
26018	4353	LRIG1	-1.08	3.74E-17	2.94E-16
253558	4877	LCLAT1	-1.08	3.22E-26	3.87E-25
7059	3447	THBS3	-1.08	8.81E-22	8.66E-21
84733	3971	CBX2	-1.08	1.60E-17	1.28E-16
80124	8176	VCPIP1	-1.08	2.56E-17	2.04E-16
23613	4916	ZMYND8	-1.08	2.82E-60	9.96E-59
7222	3455	TRPC3	-1.08	7.24E-06	2.39E-05
333929	1713	SNAI3	-1.08	0.022430437	0.042227591
50859	2552	SPOCK3	-1.08	1.37E-06	4.88E-06
120071	1857	LARGE2	-1.08	0.015300042	0.029906617
7753	3976	ZNF202	-1.09	2.52E-10	1.26E-09
55686	3213	MREG	-1.09	9.95E-06	3.23E-05
8509	2969	NDST2	-1.09	4.13E-17	3.24E-16
3312	2382	HSPA8	-1.09	2.76E-75	1.37E-73
2775	5963	GNAO1	-1.09	0.000348932	0.000915915
54414	3033	SIAE	-1.09	1.12E-12	6.62E-12
54842	4712	MFSD6	-1.09	9.87E-28	1.27E-26
6372	1677	CXCL6	-1.09	1.49E-18	1.26E-17
22913	1734	RALY	-1.09	1.74E-51	4.84E-50
64077	1388	LHPP	-1.09	6.09E-15	4.20E-14
5724	4109	PTAFR	-1.09	0.002053529	0.004728147
6542	7584	SLC7A2	-1.10	2.60E-17	2.06E-16
400954	7641	EML6	-1.10	8.35E-08	3.37E-07
57468	6066	SLC12A5	-1.10	0.000208283	0.000564315
23387	5432	SIK3	-1.10	6.00E-19	5.15E-18
2152	2371	F3	-1.10	9.55E-14	6.10E-13
11091	3165	WDR5	-1.10	9.04E-44	2.05E-42
54820	3219	NDE1	-1.10	1.01E-33	1.63E-32
155054	3244	ZNF425	-1.10	0.000112567	0.000317727

10499	8468	NCOA2	-1.10	2.83E-26	3.41E-25
29116	2987	MYLIP	-1.11	4.55E-17	3.56E-16
23301	4573	EHBP1	-1.11	2.95E-52	8.45E-51
83723	2007	FAM57B	-1.11	0.006238082	0.013173074
11096	9583	ADAMTS5	-1.11	3.77E-24	4.11E-23
408	7483	ARRB1	-1.11	7.07E-17	5.48E-16
23235	5339	SIK2	-1.11	1.82E-23	1.93E-22
130574	4101	LYPD6	-1.11	1.32E-11	7.22E-11
7579	5215	ZSCAN20	-1.11	1.66E-11	9.03E-11
25802	3967	LMOD1	-1.11	7.39E-05	0.000213841
1869	2722	E2F1	-1.11	1.04E-45	2.48E-44
5376	1748	PMP22	-1.11	2.22E-17	1.77E-16
5976	5364	UPF1	-1.11	6.23E-61	2.25E-59
283820	4260	NOMO2	-1.11	7.65E-23	7.88E-22
4355	4318	MPP2	-1.11	2.72E-19	2.39E-18
126820	2992	WDR63	-1.12	0.000296062	0.000785138
1E+08	3232	EPOP	-1.12	4.12E-13	2.49E-12
1870	4547	E2F2	-1.12	5.83E-15	4.02E-14
9644	11366	SH3PXD2A	-1.12	6.97E-50	1.87E-48
10544	1506	PROCR	-1.12	1.02E-35	1.76E-34
8793	3569	TNFRSF10D	-1.12	6.73E-24	7.27E-23
8705	1704	B3GALT4	-1.12	0.001097864	0.002657608
116039	1701	OSR2	-1.12	0.00153222	0.003611547
340061	2065	TMEM173	-1.12	3.28E-28	4.30E-27
158135	3143	TTLL11	-1.12	1.44E-07	5.66E-07
55741	1918	EDEM2	-1.12	1.30E-29	1.82E-28
140823	470	ROMO1	-1.12	7.66E-08	3.10E-07
4091	3561	SMAD6	-1.12	2.55E-16	1.91E-15
441294	2587	CTAGE15	-1.13	0.008714566	0.017873762
5049	4192	PAFAH1B2	-1.13	1.20E-52	3.50E-51
55924	5989	FAM212B	-1.13	3.71E-16	2.74E-15
9912	4249	ARHGAP44	-1.13	0.000970777	0.002370313
80790	4102	CMIP	-1.13	7.26E-44	1.66E-42
140459	4650	ASB6	-1.13	2.71E-36	4.78E-35
4249	8229	MGAT5	-1.13	2.06E-39	4.05E-38
26086	2642	GPSM1	-1.13	8.47E-32	1.28E-30
63905	1342	MANBAL	-1.13	5.24E-38	9.82E-37
25939	3728	SAMHD1	-1.13	5.10E-54	1.54E-52
11142	1199	PKIG	-1.14	2.89E-21	2.77E-20
58489	2408	ABHD17C	-1.14	3.29E-15	2.31E-14
8818	1561	DPM2	-1.14	1.99E-32	3.07E-31
83983	1459	TSSK6	-1.14	0.00867071	0.017792028
22839	3442	DLGAP4	-1.14	1.13E-35	1.95E-34
5509	3481	PPP1R3D	-1.14	2.27E-14	1.52E-13
57608	9300	JCAD	-1.14	5.70E-41	1.18E-39
84811	2226	BUD13	-1.14	1.40E-21	1.36E-20
389813	2931	AJM1	-1.14	0.000200334	0.000544353
144717	3149	FAM109A	-1.14	2.71E-10	1.35E-09

150468	4770	CKAP2L	-1.15	1.43E-41	2.98E-40
222256	6415	CDHR3	-1.15	0.016232331	0.031572639
9821	6574	RB1CC1	-1.15	7.55E-46	1.80E-44
57148	7185	RALGAPB	-1.15	3.84E-59	1.32E-57
79183	6197	TTPAL	-1.15	6.34E-38	1.18E-36
57690	10595	TNRC6C	-1.15	8.33E-11	4.33E-10
57706	3423	DENND1A	-1.15	1.36E-25	1.59E-24
151313	1561	FAHD2B	-1.15	2.24E-12	1.30E-11
444	3608	ASPH	-1.15	8.62E-79	4.57E-77
1.05E+08	472	LOC105379554	-1.15	6.57E-05	0.000191381
29767	9186	TMOD2	-1.15	8.58E-09	3.79E-08
3034	2746	HAL	-1.16	0.019159646	0.03666436
4798	4861	NFRKB	-1.16	7.84E-25	8.81E-24
9217	7942	VAPB	-1.16	4.11E-70	1.80E-68
699	3630	BUB1	-1.16	5.35E-49	1.39E-47
84552	3847	PARD6G	-1.16	2.27E-10	1.14E-09
9718	1027	ECE2	-1.16	6.07E-07	2.25E-06
10691	2577	GMEB1	-1.16	5.35E-23	5.56E-22
51282	977	SCAND1	-1.16	3.44E-33	5.45E-32
890	2811	CCNA2	-1.16	1.05E-57	3.47E-56
51203	2326	NUSAP1	-1.16	2.67E-64	1.04E-62
84627	13464	ZNF469	-1.16	2.51E-07	9.63E-07
1E+08	8413	TMEM170B	-1.16	1.47E-10	7.47E-10
5880	1538	RAC2	-1.16	4.97E-28	6.51E-27
83468	2178	GLT8D2	-1.16	1.65E-16	1.25E-15
60526	3849	LDAH	-1.16	1.41E-20	1.31E-19
51092	3919	SIDT2	-1.16	3.44E-20	3.14E-19
6934	3966	TCF7L2	-1.16	3.99E-29	5.45E-28
9133	1566	CCNB2	-1.16	1.27E-55	3.99E-54
112616	1383	CMTM7	-1.17	2.66E-18	2.21E-17
83541	1881	FAM110A	-1.17	1.30E-10	6.65E-10
56992	4315	KIF15	-1.17	2.23E-36	3.96E-35
23051	9423	ZHX3	-1.17	1.46E-67	6.10E-66
317762	5020	CCDC85C	-1.17	7.44E-30	1.05E-28
54413	4471	NLGN3	-1.17	0.001971428	0.004552089
728715	576	LOC728715	-1.18	0.004939273	0.010636407
2122	4861	MECOM	-1.18	6.90E-41	1.42E-39
5335	5204	PLCG1	-1.18	1.60E-58	5.44E-57
57683	10239	ZDBF2	-1.18	2.64E-16	1.97E-15
6227	418	RPS21	-1.18	8.81E-05	0.000252092
1.01E+08	2712	GVQW2	-1.18	0.021451867	0.040552906
55652	3010	SLC48A1	-1.18	2.24E-38	4.27E-37
1909	3922	EDNRA	-1.18	2.28E-10	1.14E-09
116535	2308	MRGPRF	-1.18	0.026888143	0.049809293
9139	7339	CBFA2T2	-1.18	1.66E-44	3.82E-43
55156	3017	ARMC1	-1.18	6.69E-36	1.16E-34
8836	1444	GGH	-1.18	3.47E-33	5.48E-32
83849	3071	SYT15	-1.18	4.31E-05	0.000128835

1382	1156	CRABP2	-1.18	9.43E-15	6.43E-14
169714	4549	QSOX2	-1.18	4.28E-38	8.08E-37
122622	1780	ADSSL1	-1.18	2.14E-10	1.07E-09
26508	4131	HEYL	-1.18	0.000403356	0.001049776
79148	1246	MMP28	-1.18	7.56E-05	0.000218639
9398	3909	CD101	-1.19	0.004061826	0.008871608
25861	3394	WHRN	-1.19	1.16E-09	5.50E-09
6836	3042	SURF4	-1.19	1.93E-92	1.32E-90
4082	4322	MARCKS	-1.19	5.83E-67	2.41E-65
124056	2134	NOXO1	-1.19	0.000963694	0.002354313
1.01E+08	1941	RPL17-C18orf32	-1.19	4.83E-10	2.35E-09
122481	3222	AK7	-1.19	0.000287146	0.000763432
53820	2116	RIPPLY3	-1.20	3.67E-12	2.09E-11
55905	2478	RNF114	-1.20	1.92E-60	6.80E-59
5932	3081	RBBP8	-1.20	7.59E-75	3.73E-73
1949	3236	EFNB3	-1.20	1.31E-26	1.60E-25
344148	7145	NCKAP5	-1.20	4.90E-05	0.000145644
79973	2301	ZNF442	-1.20	0.006213682	0.013127788
155382	1609	VPS37D	-1.20	0.000565006	0.001435992
333	2514	APLP1	-1.20	2.72E-25	3.13E-24
6837	3910	MED22	-1.20	9.19E-42	1.94E-40
7633	3132	ZNF79	-1.20	1.51E-13	9.50E-13
84858	3353	ZNF503	-1.20	2.51E-31	3.73E-30
5157	1923	PDGFRL	-1.20	0.000112354	0.000317175
58490	3740	RPRD1B	-1.20	7.01E-56	2.22E-54
26130	6856	GAPVD1	-1.20	2.79E-39	5.47E-38
57644	6486	MYH7B	-1.21	0.005860135	0.012435552
26050	4698	SLITRK5	-1.21	3.35E-17	2.65E-16
22845	2267	DOLK	-1.21	1.07E-25	1.26E-24
81832	5092	NETO1	-1.21	6.73E-08	2.74E-07
116362	704	RBP7	-1.21	5.60E-11	2.96E-10
56262	4315	LRRC8A	-1.21	3.10E-44	7.11E-43
26012	2541	NSMF	-1.21	2.16E-55	6.74E-54
23266	5993	ADGRL2	-1.21	2.30E-14	1.54E-13
57082	9419	KNL1	-1.21	2.21E-37	4.06E-36
27439	3979	TMEM121B	-1.21	0.00011411	0.000321521
162968	3481	ZNF497	-1.22	4.01E-09	1.82E-08
4067	3280	LYN	-1.22	1.93E-25	2.24E-24
81794	3646	ADAMTS10	-1.22	0.003545128	0.007827741
5688	1050	PSMA7	-1.22	6.48E-13	3.87E-12
6615	1722	SNAI1	-1.22	4.68E-12	2.64E-11
9928	6785	KIF14	-1.22	2.78E-36	4.90E-35
8646	3468	CHRD	-1.22	6.24E-07	2.31E-06
1942	1585	EFNA1	-1.22	3.53E-15	2.47E-14
50861	2441	STMN3	-1.22	9.53E-21	8.96E-20
2149	3820	F2R	-1.22	1.52E-38	2.91E-37
54928	7242	IMPAD1	-1.22	5.66E-65	2.24E-63
26133	3040	TRPC4AP	-1.22	2.38E-77	1.21E-75

1951	11974	CELSR3	-1.22	2.24E-22	2.27E-21
10076	5378	PTPRU	-1.22	1.77E-48	4.51E-47
6484	1821	ST3GAL4	-1.23	3.75E-23	3.92E-22
27433	1597	TOR2A	-1.23	7.28E-19	6.22E-18
51490	4328	SPOUT1	-1.23	1.29E-43	2.90E-42
10769	2850	PLK2	-1.23	2.42E-80	1.32E-78
7764	5633	ZNF217	-1.23	6.33E-62	2.34E-60
85366	2793	MYLK2	-1.23	0.01499331	0.029351783
374899	5025	ZNF829	-1.23	3.74E-10	1.83E-09
3696	8271	ITGB8	-1.23	6.56E-41	1.35E-39
11069	4084	RAPGEF4	-1.23	0.00203442	0.004687793
54777	8293	CFAP46	-1.23	0.001128458	0.002725357
57446	3014	NDRG3	-1.23	7.57E-51	2.07E-49
128869	1649	PIGU	-1.23	1.31E-40	2.65E-39
1903	4404	S1PR3	-1.23	2.72E-13	1.67E-12
7483	4529	WNT9A	-1.23	4.51E-05	0.000134501
5979	4174	RET	-1.23	0.018109816	0.034846785
55138	2490	FAM90A1	-1.23	0.020088718	0.038240447
1.08E+08	3834	LOC107985115	-1.23	0.019719689	0.037622617
153090	6285	DAB2IP	-1.23	3.11E-47	7.72E-46
8490	5822	RGS5	-1.23	0.000402817	0.001048528
64793	3754	CEP85	-1.23	3.70E-27	4.65E-26
284434	6791	NWD1	-1.24	0.000332899	0.000876678
55534	7086	MAML3	-1.24	1.05E-05	3.39E-05
284353	4687	NKPD1	-1.24	0.022698519	0.042682535
92340	3046	PRR29	-1.24	2.57E-05	7.90E-05
6129	868	RPL7	-1.24	1.47E-52	4.27E-51
55661	2714	DDX27	-1.24	1.18E-56	3.85E-55
1.05E+08	2968	LOC105377135	-1.24	0.00099784	0.002432641
158401	4714	C9orf84	-1.24	0.001084051	0.002627392
372	3880	ARCN1	-1.24	6.32E-67	2.61E-65
53616	9207	ADAM22	-1.24	2.81E-16	2.10E-15
9183	2888	ZW10	-1.24	3.64E-33	5.75E-32
10564	8997	ARFGEF2	-1.24	2.72E-64	1.06E-62
53904	5229	MYO3A	-1.24	0.00380932	0.00836121
6546	6160	SLC8A1	-1.24	3.56E-13	2.17E-12
10434	2487	LYPLA1	-1.24	6.26E-35	1.05E-33
57169	6733	ZNFX1	-1.24	2.02E-74	9.73E-73
6874	4647	TAF4	-1.24	5.76E-40	1.15E-38
60436	3386	TGIF2	-1.25	9.82E-35	1.64E-33
286	8301	ANK1	-1.25	1.29E-05	4.12E-05
80235	3580	PIGZ	-1.25	8.78E-19	7.48E-18
114804	4995	RNF157	-1.25	3.09E-36	5.45E-35
54542	3858	RC3H2	-1.25	2.81E-29	3.87E-28
10815	2071	CPLX1	-1.25	0.024752129	0.046192189
57167	4505	SALL4	-1.25	1.49E-09	7.01E-09
9371	6135	KIF3B	-1.26	3.01E-68	1.28E-66
54898	3960	ELOVL2	-1.26	6.83E-25	7.72E-24

57403	8702	RAB22A	-1.26	1.92E-59	6.65E-58
43	2012	ACHE	-1.26	0.006363723	0.013416068
26147	2330	PHF19	-1.26	1.60E-61	5.88E-60
7433	2445	VIPR1	-1.26	0.025204107	0.046976457
147686	3717	ZNF418	-1.26	1.96E-08	8.42E-08
23336	7169	SYNM	-1.26	3.05E-26	3.67E-25
150383	1502	CDPF1	-1.26	1.37E-09	6.44E-09
9675	3829	TTI1	-1.26	1.46E-55	4.58E-54
8701	13896	DNAH11	-1.26	0.022190894	0.041827162
3399	1252	ID3	-1.26	4.38E-27	5.48E-26
72	1216	ACTG2	-1.26	0.025292131	0.047125691
1662	3289	DDX10	-1.26	4.60E-32	7.03E-31
23526	3452	ARHGAP45	-1.26	3.16E-13	1.93E-12
23621	5506	BACE1	-1.27	2.80E-49	7.34E-48
6507	3255	SLC1A3	-1.27	3.51E-24	3.84E-23
80139	3380	ZNF703	-1.27	2.40E-18	2.00E-17
9231	6657	DLG5	-1.27	5.90E-72	2.70E-70
4863	6471	NPAT	-1.27	2.12E-41	4.42E-40
57493	9303	HEG1	-1.27	8.42E-105	7.34E-103
374900	3970	ZNF568	-1.27	6.59E-07	2.43E-06
3643	9392	INSR	-1.27	9.02E-25	1.01E-23
11249	2560	NXPH2	-1.27	1.87E-05	5.89E-05
54733	3184	SLC35F2	-1.27	1.82E-37	3.35E-36
114609	2106	TIRAP	-1.27	9.70E-10	4.62E-09
8612	1320	PLPP2	-1.27	0.000161434	0.000445328
2619	2844	GAS1	-1.27	0.017507675	0.03382
53353	15171	LRP1B	-1.27	0.001324229	0.00315697
3107	1586	HLA-C	-1.28	1.47E-91	9.81E-90
26150	1276	RIBC2	-1.28	6.89E-05	0.000199942
55861	1095	DBNDD2	-1.28	1.96E-50	5.29E-49
487	3399	ATP2A1	-1.28	1.73E-05	5.44E-05
3770	2851	KCNJ14	-1.28	0.000385985	0.00100737
8140	4361	SLC7A5	-1.28	5.51E-60	1.93E-58
9580	4088	SOX13	-1.28	8.66E-22	8.52E-21
79927	1568	FAM110D	-1.29	0.024611179	0.045953282
1482	1924	NKX2-5	-1.29	0.025755682	0.04790902
171023	6749	ASXL1	-1.29	2.21E-79	1.19E-77
399473	4769	SPRED3	-1.29	7.28E-11	3.80E-10
2303	1683	FOXC2	-1.29	2.21E-07	8.58E-07
7007	6468	TECTA	-1.29	0.011685766	0.023324274
10023	2651	FRAT1	-1.29	1.64E-06	5.79E-06
84197	1527	POMK	-1.29	0.003337603	0.007394329
10219	1815	KLRG1	-1.29	0.026058363	0.048436551
56971	2264	CEACAM19	-1.29	1.17E-11	6.42E-11
5347	2293	PLK1	-1.30	2.45E-52	7.05E-51
26084	5168	ARHGEF26	-1.30	3.90E-16	2.88E-15
55089	3807	SLC38A4	-1.30	0.022925294	0.04305884
2627	3785	GATA6	-1.30	3.81E-13	2.31E-12

3487	2246	IGFBP4	-1.30	2.35E-61	8.62E-60
1594	2503	CYP27B1	-1.30	0.004351796	0.009458436
2827	2149	GPR3	-1.30	1.39E-10	7.08E-10
55713	4324	ZNF334	-1.30	4.52E-12	2.56E-11
5349	1001	FXYD3	-1.30	0.02436769	0.045512994
64757	1939	MARC1	-1.30	8.08E-09	3.58E-08
55388	4559	MCM10	-1.30	6.92E-30	9.73E-29
89857	5955	KLHL6	-1.30	0.025876952	0.048119486
2137	6176	EXTL3	-1.30	4.76E-39	9.25E-38
7480	2234	WNT10B	-1.30	0.00080902	0.002001486
51062	2687	ATL1	-1.31	1.40E-11	7.64E-11
367	8112	AR	-1.31	0.024027375	0.044930041
157310	901	PEBP4	-1.31	0.02400744	0.044901494
157570	3315	ESCO2	-1.31	4.47E-22	4.46E-21
85414	3261	SLC45A3	-1.31	1.10E-10	5.66E-10
64800	4128	EFCAB6	-1.31	0.001575209	0.003703627
22983	4615	MAST1	-1.31	5.00E-05	0.000148347
23539	1913	SLC16A8	-1.31	0.020892456	0.039596497
51804	6277	SIX4	-1.31	5.10E-28	6.67E-27
79827	2955	CLMP	-1.31	1.73E-23	1.84E-22
399474	2500	TMEM200B	-1.31	5.19E-19	4.46E-18
85236	857	HIST1H2BK	-1.32	1.91E-27	2.42E-26
492	6049	ATP2B3	-1.32	0.022989463	0.0431748
219623	5162	TMEM26	-1.32	0.006269189	0.013227757
7001	1052	PRDX2	-1.32	2.64E-82	1.49E-80
389812	690	LCN15	-1.32	0.026199212	0.048667809
84301	2680	DDI2	-1.32	4.89E-08	2.02E-07
10179	3748	RBM7	-1.32	7.54E-34	1.22E-32
348801	2233	LNP1	-1.32	6.71E-07	2.47E-06
53826	1919	FXYD6	-1.32	6.01E-16	4.40E-15
79041	1640	TMEM38A	-1.32	5.02E-14	3.28E-13
943	3616	TNFRSF8	-1.32	0.021178567	0.040081021
83742	3250	MARVELD1	-1.32	6.00E-46	1.43E-44
3766	5323	KCNJ10	-1.32	0.016968607	0.032889519
149986	2462	LSM14B	-1.32	2.78E-56	8.89E-55
721	5444	C4B	-1.33	5.21E-11	2.76E-10
112858	3384	TP53RK	-1.33	1.04E-31	1.56E-30
1374	5260	CPT1A	-1.33	5.14E-35	8.70E-34
83546	2095	RTBDN	-1.33	0.005884022	0.012484752
54873	2601	PALMD	-1.33	0.000768685	0.00191206
1435	3787	CSF1	-1.33	6.47E-65	2.55E-63
90204	2686	ZSWIM1	-1.33	1.58E-26	1.92E-25
8969	498	HIST1H2AG	-1.33	0.020947732	0.039688559
729288	5328	ZNF286B	-1.34	4.15E-07	1.57E-06
5465	9036	PPARA	-1.34	5.20E-32	7.93E-31
1477	2175	CSTF1	-1.34	3.14E-61	1.15E-59
9480	5538	ONECUT2	-1.34	2.25E-08	9.61E-08
80129	5123	CCDC170	-1.34	0.014536078	0.028538898

5866	2330	RAB3IL1	-1.34	1.43E-10	7.27E-10
29992	1323	PILRA	-1.34	4.71E-06	1.59E-05
56241	3201	SUSD2	-1.34	0.006027869	0.012770141
5203	1382	PFDN4	-1.34	6.58E-49	1.70E-47
1259	2790	CNGA1	-1.34	0.00767848	0.015919631
57716	4942	PRX	-1.34	3.04E-12	1.74E-11
347735	2066	SERINC2	-1.34	3.28E-64	1.27E-62
85450	6633	ITPRIP	-1.34	4.14E-40	8.31E-39
2016	2889	EMX1	-1.35	0.001470785	0.003480212
949	2736	SCARB1	-1.35	5.67E-25	6.45E-24
200931	1455	SLC51A	-1.35	0.024861497	0.046376806
57165	2411	GJC2	-1.35	0.00896309	0.018339036
386724	3008	AMIGO3	-1.35	7.45E-23	7.68E-22
84630	5298	TTBK1	-1.35	0.024302006	0.045399857
230	1665	ALDOC	-1.35	2.40E-37	4.39E-36
60437	2526	CDH26	-1.36	0.020515565	0.038969484
6261	15254	RYR1	-1.36	3.10E-07	1.18E-06
7481	1927	WNT11	-1.36	0.021825132	0.041197168
84671	7876	ZNF347	-1.36	3.58E-26	4.30E-25
388228	5010	SBK1	-1.36	8.79E-13	5.21E-12
283149	7389	BCL9L	-1.36	1.15E-34	1.92E-33
5990	3999	RFX2	-1.36	1.75E-18	1.47E-17
1E+08	1132	TMEM225B	-1.36	0.014733692	0.028875826
7802	2663	DNALI1	-1.36	0.00175579	0.004089784
974	988	CD79B	-1.36	0.023597248	0.044236749
5900	3693	RALGDS	-1.36	7.77E-53	2.27E-51
57643	2614	ZSWIM5	-1.36	0.019604204	0.037430421
57026	2072	PDXP	-1.36	2.43E-29	3.35E-28
9651	4765	PLCH2	-1.36	0.023236178	0.043610479
10005	1171	ACOT8	-1.37	5.56E-27	6.95E-26
57720	6853	GPR107	-1.37	1.44E-82	8.23E-81
80725	5653	SRCIN1	-1.37	3.83E-07	1.45E-06
1E+08	729	LOC100129083	-1.37	0.022080406	0.041643632
56155	4577	TEX14	-1.37	0.013945429	0.027467306
4851	9321	NOTCH1	-1.37	3.88E-67	1.61E-65
23394	6213	ADNP	-1.37	7.92E-103	6.67E-101
140730	5207	RIMS4	-1.37	0.022567923	0.042459431
27304	3050	MOCS3	-1.37	5.55E-31	8.17E-30
6616	2056	SNAP25	-1.37	4.41E-12	2.50E-11
7058	5898	THBS2	-1.37	0.004988111	0.010727277
30009	2589	TBX21	-1.37	0.018557699	0.035635276
815	4885	CAMK2A	-1.37	0.004056178	0.008860359
140290	2730	TCP10L	-1.37	0.018517624	0.035566009
55057	5194	CRYBG2	-1.37	0.002257995	0.005159471
5591	13504	PRKDC	-1.37	6.65E-111	6.49E-109
51364	1801	ZMYND10	-1.38	1.53E-06	5.42E-06
5159	5718	PDGFRB	-1.38	3.66E-13	2.23E-12
23109	3744	DDN	-1.38	0.01022311	0.020676632

50489	2728	CD207	-1.38	0.018090894	0.034814146
9379	3535	NRXN2	-1.38	0.018001533	0.034672222
140688	5750	NOL4L	-1.38	3.77E-51	1.04E-49
1E+08	1962	HSFX2	-1.38	0.017229367	0.033336752
1.01E+08	2645	INAFM2	-1.39	1.95E-25	2.27E-24
2113	5153	ETS1	-1.39	9.28E-66	3.74E-64
54954	4349	FAM120C	-1.39	2.43E-25	2.81E-24
875	2631	CBS	-1.39	2.18E-43	4.86E-42
128866	1664	CHMP4B	-1.39	1.94E-94	1.39E-92
155051	1895	CRYGN	-1.39	0.020017666	0.038121532
10773	4111	ZBTB6	-1.39	5.31E-31	7.82E-30
1.07E+08	3711	SETDB2-PHF11	-1.39	0.000109165	0.000308518
3363	3280	HTR7	-1.39	0.000307709	0.000813354
55859	862	BEX1	-1.40	0.017060026	0.03303788
6714	4118	SRC	-1.40	7.62E-82	4.26E-80
388536	3093	ZNF790	-1.40	4.48E-07	1.68E-06
124935	4849	SLC43A2	-1.40	8.00E-44	1.82E-42
3765	10852	KCNJ9	-1.40	0.016711793	0.03244129
8190	529	MIA	-1.40	0.020128904	0.038304635
23315	5325	SLC9A8	-1.40	3.49E-43	7.74E-42
54742	1635	LY6K	-1.40	4.91E-05	0.000145938
196500	2657	PIANP	-1.40	1.83E-29	2.55E-28
6780	3440	STAU1	-1.40	9.35E-110	8.89E-108
25981	13126	DNAH1	-1.40	7.72E-12	4.30E-11
158067	2133	AK8	-1.40	0.000202256	0.000549326
1.05E+08	732	LOC105370045	-1.41	0.001225436	0.002939591
221395	5805	ADGRF5	-1.41	7.24E-09	3.22E-08
1.07E+08	1320	CNPY3-GNMT	-1.41	0.01970728	0.037607019
5142	3546	PDE4B	-1.41	2.94E-05	8.97E-05
5295	6166	PIK3R1	-1.41	1.22E-84	7.20E-83
1.01E+08	1184	LOC100996574	-1.41	0.010990046	0.022086997
54510	5668	PCDH18	-1.41	4.77E-06	1.61E-05
9744	2523	ACAP1	-1.41	0.001180106	0.002840055
80863	1931	PRRT1	-1.41	0.006103055	0.012909414
53340	955	SPA17	-1.42	4.03E-13	2.44E-12
22844	4933	FRMPD1	-1.42	0.01885044	0.036129425
29785	2660	CYP2S1	-1.42	4.81E-09	2.17E-08
114907	6711	FBXO32	-1.42	9.85E-29	1.33E-27
575	5535	ADGRB1	-1.42	0.013710647	0.027037822
57575	5664	PCDH10	-1.42	2.19E-10	1.09E-09
4313	3343	MMP2	-1.42	3.15E-85	1.90E-83
7036	2582	TFR2	-1.43	0.000132881	0.000370532
79149	2211	ZSCAN5A	-1.43	2.03E-10	1.02E-09
9227	4735	LRAT	-1.43	1.05E-05	3.40E-05
576	4864	ADGRB2	-1.43	7.92E-61	2.85E-59
1293	8778	COL6A3	-1.43	0.014354782	0.028204774
283869	1033	NPW	-1.43	0.001905714	0.004412971
9394	3966	HS6ST1	-1.44	4.44E-43	9.83E-42

10882	1517	C1QL1	-1.44	5.96E-10	2.88E-09
79102	2836	RNF26	-1.44	4.51E-45	1.06E-43
4600	2721	MX2	-1.44	0.000300881	0.000796967
140706	2660	CCM2L	-1.44	0.001207084	0.002901444
1E+08	2881	TCF24	-1.44	0.016828175	0.032642251
7052	3974	TGM2	-1.44	1.40E-14	9.42E-14
90196	2811	SYS1	-1.44	3.18E-61	1.16E-59
145773	3468	FAM81A	-1.44	6.05E-08	2.48E-07
63876	6335	PKNOX2	-1.44	0.010843383	0.021815443
10411	3714	RAPGEF3	-1.44	2.24E-48	5.67E-47
257062	1523	CATSPERD	-1.44	0.016609317	0.032252934
54863	4069	TOR4A	-1.45	3.31E-56	1.05E-54
27090	1725	ST6GALNAC4	-1.45	1.28E-20	1.19E-19
56259	1862	CTNNBL1	-1.45	2.11E-72	9.70E-71
1.01E+08	2408	MYZAP	-1.45	1.05E-05	3.40E-05
8228	2940	PNPLA4	-1.45	4.67E-29	6.37E-28
51604	2223	PIGT	-1.45	6.04E-66	2.45E-64
201191	2858	SAMD14	-1.45	1.16E-12	6.84E-12
29118	1737	DDX25	-1.45	0.015987021	0.031132988
9329	8857	GTF3C4	-1.45	8.97E-84	5.21E-82
10004	3003	NAALADL1	-1.45	0.013484795	0.026618712
401236	1322	STMND1	-1.45	0.01338749	0.026432816
58512	3856	DLGAP3	-1.46	7.72E-05	0.000222885
25803	1866	SPDEF	-1.46	0.015651948	0.030534016
84466	7934	MEGF10	-1.46	0.007477121	0.015540237
1E+08	841	SAP25	-1.46	0.001340202	0.003191625
57171	2222	DOLPP1	-1.46	3.09E-46	7.46E-45
7335	2152	UBE2V1	-1.46	2.76E-91	1.84E-89
9854	3233	C2CD2L	-1.46	2.70E-16	2.02E-15
10371	6058	SEMA3A	-1.46	5.95E-32	9.05E-31
6640	2342	SNTA1	-1.46	3.83E-49	1.00E-47
50651	2527	SLC45A1	-1.46	3.49E-05	0.000105401
1.03E+08	3297	LOC102724159	-1.46	1.37E-23	1.47E-22
10297	10032	APC2	-1.46	0.000778241	0.001932875
84649	2331	DGAT2	-1.46	4.02E-11	2.15E-10
8492	4248	PRSS12	-1.46	2.58E-37	4.70E-36
3792	1718	KEL	-1.46	0.0106578	0.021468355
56956	3032	LHX9	-1.47	0.008160036	0.016821849
1.03E+08	2519	LOC102724229	-1.47	0.000153878	0.000425739
10525	4518	HYOU1	-1.47	1.11E-91	7.47E-90
8202	7991	NCOA3	-1.47	4.78E-66	1.94E-64
23732	1753	FRRS1L	-1.47	0.01259844	0.025011071
9498	7204	SLC4A8	-1.47	5.31E-19	4.57E-18
1745	2403	DLX1	-1.47	9.60E-15	6.54E-14
54206	3166	ERRFI1	-1.47	6.40E-100	5.12E-98
79864	3539	JHY	-1.47	5.59E-09	2.51E-08
83903	2797	HASPIN	-1.47	1.51E-18	1.27E-17
222865	2723	TMEM130	-1.48	0.00133224	0.003174365

1.05E+08	5232	LOC105372798	-1.48	0.000563263	0.001431769
53832	3095	IL20RA	-1.48	0.014153536	0.027827822
3087	1772	HHEX	-1.48	2.26E-12	1.30E-11
140825	1440	NEURL2	-1.48	0.000215034	0.000581276
85455	5852	DISP2	-1.48	1.23E-13	7.78E-13
92749	1852	DRC1	-1.48	0.013156304	0.026008144
8480	1790	RAE1	-1.48	1.42E-61	5.23E-60
389119	1058	FAM212A	-1.49	0.000799196	0.001978835
3352	2857	HTR1D	-1.49	0.001785339	0.004152626
9543	2282	IGDCC3	-1.49	0.013525636	0.026690749
80045	1934	GPR157	-1.49	0.000111208	0.000314141
83481	13861	EPPK1	-1.49	6.50E-14	4.21E-13
22919	2638	MAPRE1	-1.49	2.03E-107	1.86E-105
3911	11411	LAMA5	-1.49	4.47E-125	5.19E-123
28231	3581	SLCO4A1	-1.50	5.13E-38	9.64E-37
24146	1896	CLDN15	-1.50	5.32E-13	3.19E-12
5577	3640	PRKAR2B	-1.50	4.13E-19	3.57E-18
339456	987	TMEM52	-1.50	0.000334081	0.00087953
1960	3912	EGR3	-1.50	0.005497306	0.011727235
168667	2969	BMPER	-1.50	0.009419778	0.019198282
113622	1883	ADPRHL1	-1.51	1.77E-05	5.58E-05
339665	2763	SLC35E4	-1.51	6.49E-12	3.63E-11
1241	4331	LTB4R	-1.51	1.15E-22	1.18E-21
3664	4420	IRF6	-1.51	6.35E-25	7.20E-24
91893	3081	FDXACB1	-1.51	7.19E-06	2.38E-05
259236	1861	TMIE	-1.51	6.79E-05	0.000197259
90203	2724	SNX21	-1.51	1.99E-54	6.08E-53
347088	3644	ADGRD2	-1.51	0.010416064	0.021026202
84632	3798	AFAP1L2	-1.52	1.47E-63	5.65E-62
219855	4139	SLC37A2	-1.52	1.26E-33	2.02E-32
1.05E+08	1036	LOC105372731	-1.52	0.011655627	0.023266731
146760	3625	RTN4RL1	-1.52	0.011626016	0.023212839
1.03E+08	2517	LOC102724642	-1.52	0.005706518	0.01213711
80301	3738	PLEKHO2	-1.52	1.65E-30	2.38E-29
3167	1491	HMX2	-1.53	0.009932829	0.020139964
6781	3897	STC1	-1.53	3.53E-31	5.23E-30
11037	5534	STON1	-1.53	1.95E-15	1.39E-14
7275	6150	TUB	-1.53	8.03E-18	6.54E-17
64843	1918	ISL2	-1.53	0.002178853	0.004990829
83737	6260	ITCH	-1.53	1.18E-103	1.01E-101
1991	1020	ELANE	-1.53	0.011010703	0.022126009
23145	15580	SSPO	-1.53	6.40E-07	2.36E-06
252995	3046	FNDC5	-1.53	1.00E-05	3.24E-05
7253	2746	TSHR	-1.54	9.43E-13	5.59E-12
83756	3432	TAS1R3	-1.54	0.00152898	0.003604952
9265	4482	CYTH3	-1.54	3.95E-57	1.30E-55
55734	2689	ZFP64	-1.54	4.26E-42	9.14E-41
387522	2925	TMEM189-	-1.54	0.00847973	0.017430338

		UBE2V1			
55790	2683	CSGALNACT1	-1.54	4.96E-05	0.000147026
10481	3047	HOXB13	-1.54	1.21E-06	4.35E-06
401097	2606	C3orf80	-1.54	0.010658057	0.021468355
149478	2322	BTBD19	-1.54	5.78E-06	1.94E-05
116444	2683	GRIN3B	-1.54	0.000948868	0.002323992
9501	2470	RPH3AL	-1.54	7.21E-25	8.12E-24
407738	1758	FAM19A1	-1.54	0.009131678	0.018651759
3293	1607	HSD17B3	-1.54	0.009205157	0.018786726
79570	2930	NKAIN1	-1.55	0.00686826	0.014379079
79913	2479	ACTR5	-1.55	1.18E-23	1.26E-22
8785	2287	MATN4	-1.55	0.009569453	0.019474292
9334	4743	B4GALT5	-1.55	1.93E-120	2.08E-118
6789	6378	STK4	-1.55	1.34E-69	5.83E-68
57158	4160	JPH2	-1.55	1.39E-13	8.79E-13
10256	2475	CNKSR1	-1.55	0.002589913	0.005856888
729359	6353	PLIN4	-1.55	8.31E-06	2.72E-05
4826	1252	NNAT	-1.56	0.000511143	0.001307386
55244	3279	SLC47A1	-1.56	1.15E-15	8.28E-15
56917	1837	MEIS3	-1.56	1.49E-42	3.25E-41
388795	4133	EFCAB8	-1.56	0.009776409	0.019849987
155185	7136	AMZ1	-1.56	1.44E-39	2.84E-38
85452	4835	CFAP74	-1.56	0.008332261	0.017153333
8347	438	HIST1H2BC	-1.56	0.000848802	0.002092911
3887	1926	KRT81	-1.57	0.007129252	0.014872908
3240	1301	HP	-1.57	0.000186208	0.00050846
159371	2836	SLC35G1	-1.57	4.26E-13	2.57E-12
2281	1049	FKBP1B	-1.57	1.06E-13	6.76E-13
89958	3869	SAPCD2	-1.57	6.95E-71	3.10E-69
6812	3850	STXBP1	-1.57	7.18E-57	2.34E-55
1995	2165	ELAVL3	-1.57	0.008978582	0.018366503
2103	3451	ESRRB	-1.57	0.009011215	0.018429014
10953	2039	TOMM34	-1.57	2.15E-73	1.01E-71
2651	4037	GCNT2	-1.57	2.48E-32	3.82E-31
149175	2438	MANEAL	-1.57	2.23E-37	4.08E-36
9777	3792	TM9SF4	-1.58	1.06E-109	1.00E-107
8128	5464	ST8SIA2	-1.58	0.003861689	0.008464654
7079	1667	TIMP4	-1.58	0.000337103	0.000887091
442213	4178	PTCHD4	-1.58	1.51E-49	3.99E-48
131578	5883	LRRC15	-1.58	0.001658668	0.003883413
140606	739	SELENOM	-1.58	1.01E-36	1.80E-35
10602	5347	CDC42EP3	-1.58	9.65E-122	1.05E-119
6185	2532	RPN2	-1.58	1.86E-182	4.09E-180
348094	3122	ANKDD1A	-1.59	4.16E-12	2.36E-11
374907	3193	B3GNT8	-1.59	0.008339018	0.01716294
57611	4277	ISLR2	-1.59	0.007569627	0.015708612
566	936	AZU1	-1.59	0.007494092	0.015568225
23359	4718	FAM189A1	-1.59	1.86E-06	6.55E-06

59352	2554	LGR6	-1.59	0.001365323	0.003248402
339263	7912	C17orf51	-1.59	5.32E-25	6.06E-24
5522	4092	PPP2R2C	-1.60	0.001012493	0.002465392
3386	1277	ICAM4	-1.60	0.008165195	0.016824793
54734	1876	RAB39A	-1.60	0.00034939	0.00091671
51614	1368	ERGIC3	-1.60	2.04E-103	1.73E-101
147700	1793	KLC3	-1.60	1.98E-08	8.50E-08
23316	6695	CUX2	-1.60	0.007151657	0.014916146
51497	2320	NELFCD	-1.60	3.70E-116	3.84E-114
80329	3048	ULBP1	-1.60	2.29E-05	7.10E-05
4846	3983	NOS3	-1.60	0.00266164	0.006006098
23329	7378	TBC1D30	-1.60	0.000476198	0.001223584
4212	3116	MEIS2	-1.60	8.00E-42	1.69E-40
5818	4130	NECTIN1	-1.60	4.85E-24	5.26E-23
84985	1566	FAM83A	-1.61	0.006578257	0.013828971
55800	5682	SCN3B	-1.61	0.007541317	0.015657177
128497	752	SPATA25	-1.61	0.001945812	0.004497033
3768	5006	KCNJ12	-1.61	3.94E-15	2.75E-14
284422	908	SMIM24	-1.61	2.25E-05	6.97E-05
146395	4538	GSG1L	-1.61	0.007503327	0.015585588
7454	1844	WAS	-1.61	0.001936354	0.00447867
53345	1483	TM6SF2	-1.61	0.000345856	0.000908646
11151	1741	CORO1A	-1.61	3.95E-13	2.39E-12
440073	6474	IQSEC3	-1.62	0.006693981	0.01405562
131540	1111	ZDHHC19	-1.62	0.007417749	0.015427668
6543	3264	SLC8A2	-1.62	0.007379853	0.015350647
728392	1503	LOC728392	-1.62	5.61E-18	4.59E-17
5141	4019	PDE4A	-1.62	6.79E-07	2.50E-06
2494	4782	NR5A2	-1.62	0.006404176	0.013494947
80108	2442	ZFP2	-1.62	0.003406606	0.007533116
392862	4107	GRID2IP	-1.62	0.001898199	0.004398435
6590	625	SLPI	-1.63	9.95E-87	6.22E-85
10053	1756	AP1M2	-1.63	1.00E-05	3.24E-05
162466	1967	PHOSPHO1	-1.63	0.000609277	0.001538387
8193	2354	DPF1	-1.63	2.04E-17	1.63E-16
116092	1317	DNTTIP1	-1.63	2.22E-73	1.04E-71
440854	3691	CAPN14	-1.63	0.000333922	0.000879241
115650	934	TNFRSF13C	-1.63	0.001154931	0.00278513
56953	1614	NT5M	-1.63	1.25E-16	9.54E-16
652	1825	BMP4	-1.63	1.21E-27	1.55E-26
4884	5437	NPTX1	-1.63	0.005804024	0.012331209
65055	3868	REEP1	-1.63	1.90E-08	8.16E-08
5798	3327	PTPRN	-1.63	0.005802163	0.012330203
728113	2100	ANXA8L1	-1.63	1.70E-27	2.17E-26
348738	1902	C2orf48	-1.63	8.90E-06	2.91E-05
400746	3991	NCMAP	-1.63	0.000150312	0.000416978
84623	2536	KIRREL3	-1.64	0.001736501	0.004052041
10904	2115	BLCAP	-1.64	6.70E-78	3.46E-76

6000	2441	RGS7	-1.64	9.73E-07	3.52E-06
84107	3660	ZIC4	-1.64	0.005913155	0.01254105
51278	2350	IER5	-1.64	2.25E-52	6.50E-51
3822	1223	KLRC2	-1.64	0.000716774	0.001791734
1143	2845	CHRNB4	-1.64	0.001816145	0.00422262
64399	4713	HHIP	-1.64	1.68E-11	9.15E-11
94031	2316	HTRA3	-1.65	1.33E-10	6.80E-10
22899	3241	ARHGEF15	-1.65	0.005605033	0.011942707
219654	4933	ZCCHC24	-1.65	2.84E-48	7.19E-47
90952	1629	ESAM	-1.65	0.003147637	0.007008423
3232	2556	HOXD3	-1.65	2.79E-18	2.32E-17
55245	2354	UQCC1	-1.65	2.55E-99	1.99E-97
9118	3245	INA	-1.65	3.15E-15	2.21E-14
3196	2170	TLX2	-1.66	0.005556087	0.01184552
51268	2020	PIPOX	-1.66	0.005012085	0.010774922
3624	3586	INHBA	-1.66	3.69E-37	6.70E-36
2298	2204	FOXD4	-1.66	0.000706908	0.001769061
79784	6807	MYH14	-1.66	0.005584704	0.011902244
2262	3078	GPC5	-1.66	0.001612264	0.003782742
27092	3430	CACNG4	-1.66	0.002316844	0.005281715
64220	2176	STRA6	-1.67	5.12E-05	0.000151535
6855	2449	SYP	-1.67	3.71E-07	1.41E-06
9825	4032	SPATA2	-1.68	3.33E-42	7.19E-41
166979	1912	CDC20B	-1.68	0.003745543	0.008239545
9034	1596	CCRL2	-1.68	0.005474083	0.011683303
1.02E+08	499	LOC101927345	-1.68	0.004826646	0.010417862
3758	2457	KCNJ1	-1.68	0.004897949	0.010551478
154790	1239	CLEC2L	-1.68	0.004889067	0.010535955
84915	2728	FAM222A	-1.68	5.92E-06	1.98E-05
202500	2184	TCTE1	-1.68	0.003645001	0.008035283
54831	1924	BEST2	-1.69	0.00486199	0.010483961
56129	3756	PCDHB7	-1.69	0.004192521	0.009132367
3397	1068	ID1	-1.69	7.73E-64	2.98E-62
83593	3532	RASSF5	-1.69	2.77E-26	3.34E-25
6405	3379	SEMA3F	-1.69	3.74E-67	1.57E-65
2040	3198	STOM	-1.69	1.12E-123	1.27E-121
374860	4544	ANKRD30B	-1.69	0.00277015	0.006224867
60625	2548	DHX35	-1.70	4.52E-52	1.28E-50
3575	4491	IL7R	-1.70	3.30E-09	1.51E-08
23604	3494	DAPK2	-1.70	5.62E-14	3.66E-13
1748	1765	DLX4	-1.70	9.46E-09	4.17E-08
126393	1480	HSPB6	-1.70	0.000707765	0.001770958
390928	2788	ACP7	-1.70	0.004776622	0.010319919
51098	2045	IFT52	-1.70	3.91E-93	2.75E-91
1543	2701	CYP1A1	-1.70	0.00269966	0.006085709
948	4172	CD36	-1.71	0.001139421	0.002749589
90113	3552	VWA5B2	-1.71	0.000216171	0.00058417
8970	481	HIST1H2BJ	-1.71	8.75E-05	0.000250658

23037	11093	PDZD2	-1.71	2.68E-65	1.07E-63
9052	2856	GPRC5A	-1.71	8.91E-71	3.96E-69
8600	1852	TNFSF11	-1.71	0.001062431	0.002579568
8817	1999	FGF18	-1.71	6.77E-12	3.78E-11
53342	2307	IL17D	-1.71	1.18E-06	4.24E-06
4772	2882	NFATC1	-1.72	9.71E-26	1.14E-24
947	2621	CD34	-1.72	3.56E-05	0.000107496
23166	7884	STAB1	-1.72	0.004442708	0.009647767
1311	2471	COMP	-1.72	0.001897081	0.004396576
952	5694	CD38	-1.72	0.000894849	0.002198225
656	4615	BMP8B	-1.72	3.00E-08	1.27E-07
63939	5080	FAM217B	-1.72	1.11E-65	4.47E-64
390937	2497	LOC390937	-1.72	0.002848117	0.006391178
146713	2832	RBFOX3	-1.72	0.004034267	0.008815749
200958	2611	MUC20	-1.72	2.60E-07	9.98E-07
128646	868	SIRPD	-1.72	0.004337619	0.009432237
56961	2598	SHD	-1.72	0.003979281	0.008705228
140686	1000	WFDC3	-1.72	0.000204968	0.000556181
7161	5091	TP73	-1.72	1.36E-73	6.46E-72
136	1907	ADORA2B	-1.73	8.27E-31	1.21E-29
2529	2428	FUT7	-1.73	0.00424236	0.0092364
9625	4970	AATK	-1.73	0.000120193	0.000337057
3671	2128	ISLR	-1.73	0.003851863	0.008447285
117248	4313	GALNT15	-1.73	4.00E-08	1.67E-07
645191	2130	LINGO3	-1.74	0.003754838	0.008257947
112936	3690	VPS26B	-1.74	1.21E-44	2.82E-43
94015	3375	TTYH2	-1.74	2.69E-07	1.03E-06
5321	3008	PLA2G4A	-1.74	1.13E-20	1.06E-19
84570	7087	COL25A1	-1.74	0.000138744	0.000386344
10955	4379	SERINC3	-1.74	2.49E-181	5.34E-179
1381	782	CRABP1	-1.74	0.003615065	0.007972258
375033	4518	PEAR1	-1.75	3.34E-14	2.21E-13
140597	1123	TCEAL2	-1.75	0.000498593	0.00127688
23054	6751	NCOA6	-1.75	2.88E-86	1.78E-84
54739	2562	XAF1	-1.75	1.00E-09	4.76E-09
1592	2119	CYP26A1	-1.75	0.002875045	0.006444278
8022	2197	LHX3	-1.75	0.003517193	0.007767025
161497	4638	STRC	-1.75	0.000459435	0.001184622
10406	570	WFDC2	-1.75	9.34E-110	8.89E-108
8813	1115	DPM1	-1.76	4.69E-96	3.43E-94
1280	4880	COL2A1	-1.76	1.89E-14	1.27E-13
84467	9086	FBN3	-1.76	0.003232459	0.007185578
4858	7673	NOVA2	-1.76	0.003183401	0.007083616
5064	2845	PALM	-1.77	4.94E-33	7.78E-32
51083	830	GAL	-1.77	0.003040514	0.006790329
63941	1945	NECAB3	-1.77	1.69E-90	1.11E-88
84628	2763	NTNG2	-1.77	0.001560585	0.003672645
6660	5138	SOX5	-1.78	0.000416804	0.001082241

644943	2698	RASSF10	-1.78	1.90E-20	1.75E-19
8365	374	HIST1H4H	-1.78	0.001629645	0.003820495
2307	1353	FOXS1	-1.78	0.003128799	0.00697198
25980	2525	AAR2	-1.78	4.19E-96	3.09E-94
5414	1592	SEPT4	-1.78	3.74E-25	4.28E-24
3061	1926	HCRTR1	-1.78	0.003102691	0.006917883
1.05E+08	3724	LOC105373132	-1.79	0.002401566	0.005460844
222663	7476	SCUBE3	-1.79	1.36E-13	8.56E-13
1.08E+08	7801	LOC107984282	-1.79	0.003000298	0.006703885
50619	2320	DEF6	-1.79	2.51E-12	1.44E-11
387521	2295	TMEM189	-1.80	2.04E-102	1.70E-100
389692	2373	MAFA	-1.80	0.002643304	0.005969271
10324	2472	KLHL41	-1.80	0.000425033	0.001101835
1.08E+08	756	LOC107986035	-1.80	2.02E-05	6.31E-05
285313	3304	IGSF10	-1.81	2.73E-05	8.39E-05
54626	4273	HES2	-1.81	0.000650062	0.001633937
7134	705	TNNC1	-1.81	0.00176372	0.004107179
729956	6035	SHISA7	-1.81	0.000682634	0.001710967
140710	14387	SOGA1	-1.81	2.57E-79	1.37E-77
90668	4296	CARMIL3	-1.82	0.00186298	0.004323594
81621	2050	KAZALD1	-1.82	4.79E-17	3.74E-16
7138	1077	TNNT1	-1.82	1.94E-51	5.36E-50
199964	863	TMEM61	-1.82	0.001092641	0.002646409
161357	5250	MDGA2	-1.82	0.002512863	0.005697875
10044	2557	SH2D3C	-1.82	0.001105171	0.002673475
283576	3408	ZDHHC22	-1.82	0.002495841	0.005661445
8370	396	HIST2H4A	-1.82	0.000347984	0.000913697
554313	396	HIST2H4B	-1.82	0.000347984	0.000913697
729747	1982	ZNF878	-1.83	0.000470675	0.001210445
56884	4769	FSTL5	-1.83	0.001777938	0.004137035
127294	5800	MYOM3	-1.83	0.002421162	0.005500474
3589	2381	IL11	-1.83	2.11E-13	1.31E-12
116969	1196	ART5	-1.83	1.20E-06	4.31E-06
6926	4775	TBX3	-1.83	1.87E-23	1.98E-22
9715	4591	FAM131B	-1.84	6.34E-08	2.59E-07
9353	6424	SLIT2	-1.84	6.20E-19	5.32E-18
7349	813	UCN	-1.84	7.66E-05	0.000221272
375593	1358	TRIM73	-1.84	0.000469788	0.001208337
23237	2948	ARC	-1.84	0.001322909	0.003154246
389136	10257	VGLL3	-1.84	5.82E-40	1.16E-38
1434	3628	CSE1L	-1.84	3.80E-226	1.27E-223
440829	1674	SHISA8	-1.85	0.000608791	0.001537589
91975	3406	ZNF300	-1.85	1.23E-40	2.50E-39
79983	3854	POF1B	-1.85	0.002110582	0.0048482
125965	1679	COX6B2	-1.85	2.22E-07	8.59E-07
9901	6457	SRGAP3	-1.85	9.28E-18	7.52E-17
57333	1953	RCN3	-1.85	1.12E-41	2.36E-40
51085	3445	MLXIPL	-1.85	8.67E-08	3.49E-07

29906	2097	ST8SIA5	-1.85	0.001827268	0.004245704
57524	5695	CASKIN1	-1.85	1.30E-07	5.14E-07
11247	1821	NXPH4	-1.86	9.41E-20	8.44E-19
375791	1314	CYSRT1	-1.86	6.08E-09	2.72E-08
3017	824	HIST1H2BD	-1.86	1.13E-12	6.65E-12
1303	8643	COL12A1	-1.86	6.39E-132	8.80E-130
27296	1240	TP53TG5	-1.86	0.001959965	0.004527386
54854	2491	FAM83E	-1.87	0.001933159	0.004471862
51702	2938	PADI3	-1.87	0.000257386	0.000688686
113235	6422	SLC46A1	-1.87	1.21E-96	9.00E-95
2769	2311	GNA15	-1.87	0.000883817	0.002173518
256472	2546	TMEM151A	-1.87	0.00032974	0.000868874
255239	2543	ANKK1	-1.87	0.000417654	0.001084132
4621	7404	MYH3	-1.88	5.99E-16	4.39E-15
51012	2575	PRELID3B	-1.88	1.35E-104	1.16E-102
8514	3813	KCNAB2	-1.88	4.07E-33	6.42E-32
8938	4499	BAIAP3	-1.88	1.50E-18	1.26E-17
56963	3058	RGMA	-1.88	0.001749186	0.00407654
3005	2336	H1F0	-1.89	6.23E-124	7.14E-122
1910	2544	EDNRB	-1.89	0.00169376	0.003959312
59341	3095	TRPV4	-1.89	1.12E-31	1.68E-30
9071	2490	CLDN10	-1.89	7.04E-06	2.33E-05
3012	564	HIST1H2AE	-1.89	1.58E-05	5.01E-05
387496	1543	RASL11A	-1.90	1.60E-26	1.94E-25
5326	5656	PLAGL2	-1.90	2.20E-77	1.13E-75
8334	546	HIST1H2AC	-1.90	4.94E-09	2.22E-08
23057	5467	NMNAT2	-1.90	2.73E-07	1.05E-06
340745	3157	LRIT2	-1.90	0.000599741	0.00151776
140699	2043	MROH8	-1.90	5.34E-08	2.20E-07
4586	17474	MUC5AC	-1.91	0.001558665	0.003668614
4900	1237	NRGN	-1.91	1.02E-30	1.48E-29
22974	3361	TPX2	-1.91	2.07E-185	4.65E-183
283726	3146	SAXO2	-1.91	0.001020056	0.002482788
146754	14793	DNAH2	-1.91	0.000432444	0.001119904
57452	3971	GALNT16	-1.91	4.94E-32	7.54E-31
2906	5267	GRIN2D	-1.91	1.34E-27	1.72E-26
6098	6685	ROS1	-1.91	0.000103849	0.000294522
57540	4872	DISP3	-1.91	4.37E-06	1.48E-05
11248	5553	NXPH3	-1.91	3.23E-13	1.97E-12
10669	1783	CGREF1	-1.91	8.67E-09	3.83E-08
11309	4057	SLCO2B1	-1.91	9.87E-13	5.84E-12
5727	7894	PTCH1	-1.92	1.37E-21	1.33E-20
9796	1923	PHYHIP	-1.92	0.00014297	0.000397543
116151	3066	FAM210B	-1.92	2.22E-170	4.38E-168
91120	3164	ZNF682	-1.93	3.54E-09	1.61E-08
132243	4637	H1FOO	-1.93	0.001345893	0.003203888
1E+08	1016	HGC6	-1.93	0.001376054	0.003271663
2812	958	GP1BB	-1.93	5.05E-05	0.000149688

379	1602	ARL4D	-1.93	6.48E-123	7.34E-121
65989	2046	DLK2	-1.94	2.97E-24	3.26E-23
3553	1498	IL1B	-1.94	3.74E-06	1.27E-05
1136	3318	CHRNA3	-1.94	0.000240054	0.000644983
55653	1269	BCAS4	-1.94	1.29E-34	2.14E-33
5933	5763	RBL1	-1.94	3.39E-122	3.77E-120
81607	3520	NECTIN4	-1.94	2.68E-14	1.78E-13
57463	5123	AMIGO1	-1.94	1.93E-27	2.45E-26
5794	3924	PTPRH	-1.94	6.09E-10	2.94E-09
9355	2416	LHX2	-1.94	1.89E-05	5.92E-05
57121	2695	LPAR5	-1.94	7.63E-06	2.51E-05
9148	3940	NEURL1	-1.94	1.25E-06	4.47E-06
1.08E+08	5338	LOC107985410	-1.95	0.000787416	0.001952933
8349	2223	HIST2H2BE	-1.95	2.48E-35	4.23E-34
3747	4666	KCNC2	-1.95	0.000101195	0.000287548
3485	1119	IGFBP2	-1.95	1.63E-18	1.37E-17
256949	2801	KANK3	-1.95	0.000120217	0.000337071
80217	3670	CFAP43	-1.95	2.00E-05	6.25E-05
54922	3311	RASIP1	-1.95	4.22E-05	0.0001263
1.03E+08	2997	LOC400927-CSNK1E	-1.95	5.22E-07	1.94E-06
51334	1737	PRR16	-1.95	1.07E-22	1.09E-21
81572	1384	PDRG1	-1.96	3.46E-69	1.49E-67
30811	7385	HUNK	-1.96	0.00027837	0.00074143
3014	1651	H2AFX	-1.96	2.08E-75	1.03E-73
1.05E+08	2110	LOC105373944	-1.96	0.001098281	0.002658257
23017	4487	FAIM2	-1.96	0.00110278	0.002668054
9452	1851	ITM2A	-1.96	0.001137131	0.002744809
9537	3270	TP53I11	-1.97	2.21E-53	6.55E-52
9469	6998	CHST3	-1.97	1.26E-121	1.37E-119
11065	860	UBE2C	-1.97	2.45E-167	4.74E-165
23046	9339	KIF21B	-1.97	3.56E-29	4.88E-28
7048	4413	TGFBR2	-1.99	1.27E-133	1.77E-131
254528	1731	MEIOB	-1.99	0.000591542	0.001498934
1672	484	DEFB1	-1.99	2.85E-13	1.75E-12
23129	4892	PLXND1	-1.99	3.34E-110	3.22E-108
610	3459	HCN2	-2.00	9.29E-10	4.43E-09
169611	6111	OLFML2A	-2.00	1.10E-129	1.44E-127
5915	2680	RARB	-2.00	3.29E-05	9.98E-05
164633	3001	CABP7	-2.00	4.46E-05	0.000133035
26164	3684	MTG2	-2.00	1.71E-129	2.22E-127
4059	2559	BCAM	-2.00	2.01E-196	5.10E-194
4684	5342	NCAM1	-2.00	2.32E-05	7.18E-05
23310	4982	NCAPD3	-2.00	4.22E-129	5.36E-127
284391	6350	ZNF844	-2.00	2.39E-09	1.11E-08
1602	4099	DACH1	-2.01	5.98E-06	2.00E-05
7056	4048	THBD	-2.01	1.15E-08	5.01E-08
283948	2102	NHLRC4	-2.01	0.000856658	0.002110816

5745	2007	PTH1R	-2.01	5.21E-05	0.000153945
6853	3210	SYN1	-2.02	1.96E-06	6.87E-06
23509	5232	POFUT1	-2.02	7.80E-204	2.24E-201
1.01E+08	3195	LOC100996713	-2.02	0.00076245	0.001897102
7732	2993	RNF112	-2.02	0.000601028	0.001520584
9143	2076	SYNGR3	-2.03	7.67E-18	6.25E-17
142680	2099	SLC34A3	-2.03	0.000439528	0.001137255
8786	1078	RGS11	-2.03	8.03E-05	0.000231065
4605	2783	MYBL2	-2.03	2.02E-137	2.91E-135
64098	4201	PARVG	-2.04	9.90E-09	4.36E-08
116835	2222	HSPA12B	-2.04	0.000207564	0.000562795
128486	958	FITM2	-2.05	9.60E-13	5.68E-12
117581	1186	TWIST2	-2.05	0.000629363	0.001585499
91752	4700	ZNF804A	-2.05	0.000183069	0.000500889
64411	5028	ARAP3	-2.05	3.19E-91	2.12E-89
1.08E+08	4421	LOC107987188	-2.05	0.000621647	0.001567393
79839	2132	CCDC102B	-2.06	0.0001189	0.000333852
23467	5831	NPTXR	-2.06	8.19E-102	6.71E-100
4133	6043	MAP2	-2.07	1.10E-53	3.28E-52
441631	3975	TSPAN11	-2.07	2.76E-10	1.37E-09
64137	3843	ABCG4	-2.07	5.65E-10	2.74E-09
1012	3720	CDH13	-2.08	5.56E-102	4.57E-100
1747	2613	DLX3	-2.08	0.000406061	0.001056352
3991	2762	LIPE	-2.08	2.93E-17	2.32E-16
2203	1647	FBP1	-2.08	0.00056174	0.001428101
1050	2631	CEBPA	-2.08	9.04E-07	3.28E-06
5138	3820	PDE2A	-2.08	8.97E-05	0.00025654
8447	1956	DOC2B	-2.08	0.000554735	0.001411504
116931	10440	MED12L	-2.09	3.89E-19	3.37E-18
6790	2237	AURKA	-2.09	3.76E-197	9.69E-195
79625	2911	NDNF	-2.10	5.77E-09	2.58E-08
4487	1940	MSX1	-2.10	4.62E-10	2.25E-09
168455	4426	CCDC71L	-2.10	2.96E-128	3.62E-126
59285	1748	CACNG6	-2.10	0.000188927	0.000515567
26232	1724	FBXO2	-2.10	4.19E-19	3.62E-18
7442	4133	TRPV1	-2.10	5.34E-33	8.39E-32
4585	3958	MUC4	-2.10	0.000174795	0.00047944
284339	2145	TMEM145	-2.11	3.40E-12	1.94E-11
978	985	CDA	-2.11	3.97E-15	2.77E-14
126567	3124	C2CD4C	-2.11	2.74E-12	1.57E-11
6004	2432	RGS16	-2.12	4.73E-05	0.000140776
8532	2321	CPZ	-2.12	1.03E-19	9.23E-19
55544	2401	RBM38	-2.12	1.51E-99	1.20E-97
57692	3714	MAGEE1	-2.12	4.08E-16	3.01E-15
5176	1493	SERPINF1	-2.13	2.25E-20	2.07E-19
100	1558	ADA	-2.13	1.49E-43	3.34E-42
10501	3925	SEMA6B	-2.14	9.92E-115	1.01E-112
4185	4836	ADAM11	-2.15	4.65E-18	3.82E-17

22943	1815	DKK1	-2.15	2.31E-34	3.80E-33
112885	3500	PHF21B	-2.15	0.000255927	0.000685042
222183	3642	SRRM3	-2.15	2.02E-06	7.09E-06
6943	1513	TCF21	-2.15	7.14E-18	5.84E-17
1364	1859	CLDN4	-2.15	3.87E-64	1.50E-62
51440	4454	HPCAL4	-2.15	4.01E-06	1.36E-05
3932	2102	LCK	-2.16	7.14E-05	0.000207
66004	4593	LYNX1	-2.16	6.37E-63	2.40E-61
56479	6265	KCNQ5	-2.16	7.94E-06	2.61E-05
27115	5387	PDE7B	-2.16	2.20E-18	1.84E-17
283316	5953	CD163L1	-2.17	4.15E-05	0.000124444
4051	2643	CYP4F3	-2.17	0.000152022	0.000421262
64856	4630	VWA1	-2.17	7.98E-91	5.25E-89
2202	3034	EFEMP1	-2.17	6.52E-210	2.00E-207
84894	3004	LINGO1	-2.18	0.000268206	0.000715967
653689	1104	GSTT2B	-2.18	2.73E-36	4.82E-35
5197	744	PF4V1	-2.18	0.000253386	0.000678854
723790	534	HIST2H2AA4	-2.18	3.79E-22	3.79E-21
8337	534	HIST2H2AA3	-2.18	3.79E-22	3.79E-21
147495	2849	APCDD1	-2.18	0.000296775	0.000786795
55786	2106	ZNF415	-2.19	3.14E-05	9.55E-05
22859	7564	ADGRL1	-2.19	2.06E-128	2.56E-126
129804	3765	FBLN7	-2.19	5.68E-85	3.41E-83
11322	2490	TMC6	-2.20	1.53E-37	2.82E-36
81928	3785	CABLES2	-2.20	1.42E-115	1.46E-113
51673	1068	TPPP3	-2.20	0.000166515	0.000458562
338596	2195	ST8SIA6	-2.20	2.33E-07	8.99E-07
5266	579	PI3	-2.21	1.53E-08	6.62E-08
1571	1667	CYP2E1	-2.21	3.65E-10	1.80E-09
3855	1723	KRT7	-2.21	2.02E-99	1.59E-97
5420	5911	PODXL	-2.21	9.31E-28	1.20E-26
7539	2906	ZFP37	-2.21	3.70E-13	2.25E-12
7133	3641	TNFRSF1B	-2.22	4.95E-34	8.07E-33
388335	2532	TMEM220	-2.22	6.73E-42	1.43E-40
11240	3537	PADI2	-2.23	1.65E-13	1.03E-12
139735	6365	ZFP92	-2.23	1.50E-05	4.76E-05
388394	1110	RPRML	-2.23	0.000184522	0.000504477
27127	3537	SMC1B	-2.23	0.000184975	0.000505531
6657	2520	SOX2	-2.23	8.49E-05	0.000243502
9172	4916	MYOM2	-2.23	7.66E-07	2.80E-06
653145	2048	ANXA8	-2.24	4.67E-97	3.49E-95
5327	3221	PLAT	-2.24	1.38E-99	1.10E-97
2882	1246	GPX7	-2.25	4.44E-05	0.00013262
5152	1658	PDE9A	-2.25	0.000155068	0.000428432
94027	1228	CGB7	-2.26	5.48E-15	3.78E-14
7546	2991	ZIC2	-2.26	0.000106631	0.000301738
10235	3285	RASGRP2	-2.26	0.000105533	0.000298748
89853	3677	MVB12B	-2.27	6.19E-49	1.60E-47

11045	1284	UPK1A	-2.27	0.00017007	0.000467337
29942	1972	PURG	-2.27	9.70E-05	0.000276346
27128	3096	CYTH4	-2.28	3.44E-06	1.18E-05
54492	5824	NEURL1B	-2.28	1.30E-108	1.20E-106
314	2681	AOC2	-2.28	1.56E-10	7.93E-10
2676	1980	GFRA3	-2.28	2.08E-07	8.06E-07
8326	2342	FZD9	-2.28	7.11E-09	3.17E-08
1056	2386	CEL	-2.29	1.92E-28	2.55E-27
26251	2989	KCNG2	-2.29	2.50E-09	1.16E-08
5272	4287	SERPINB9	-2.29	2.17E-82	1.23E-80
10331	2720	B3GNT3	-2.30	2.10E-14	1.41E-13
10468	2431	FST	-2.30	4.46E-38	8.41E-37
6693	6911	SPN	-2.30	9.24E-06	3.01E-05
22997	3333	IGSF9B	-2.31	9.91E-05	0.000281985
63940	1501	GPSM3	-2.31	2.52E-24	2.77E-23
201161	1181	CENPV	-2.31	7.83E-27	9.70E-26
254439	1185	C11orf86	-2.32	0.000120561	0.000337929
3209	2514	HOXA13	-2.32	4.10E-05	0.000122865
55130	2811	ARMC4	-2.32	5.64E-20	5.12E-19
1001	3591	CDH3	-2.32	1.17E-19	1.04E-18
138429	2193	PIP5KL1	-2.33	5.90E-14	3.83E-13
79987	12356	SVEP1	-2.33	3.19E-14	2.11E-13
6653	9388	SORL1	-2.33	1.29E-08	5.63E-08
5457	3817	POU4F1	-2.33	1.49E-07	5.87E-07
147837	1303	ZNF563	-2.33	6.63E-08	2.71E-07
114795	9143	TMEM132B	-2.33	4.43E-06	1.50E-05
54456	2236	MOV10L1	-2.34	8.13E-05	0.000234049
57580	5985	PREX1	-2.34	2.23E-65	8.91E-64
5413	2115	SEPT5	-2.34	1.49E-26	1.82E-25
2895	8137	GRID2	-2.34	1.92E-05	6.01E-05
10232	2127	MSLN	-2.34	4.10E-13	2.48E-12
25927	1687	CNRIP1	-2.35	4.18E-45	9.85E-44
7025	2115	NR2F1	-2.35	3.59E-26	4.32E-25
2042	3219	EPHA3	-2.36	1.16E-05	3.72E-05
116	3187	ADCYAP1	-2.36	9.07E-05	0.000259138
84958	1989	SYTL1	-2.36	1.03E-51	2.90E-50
7122	2332	CLDN5	-2.36	9.02E-05	0.000257704
1236	2157	CCR7	-2.36	1.68E-06	5.95E-06
26153	5902	KIF26A	-2.36	4.16E-05	0.000124708
2781	6896	GNAZ	-2.37	4.19E-24	4.55E-23
84152	1315	PPP1R1B	-2.37	6.17E-05	0.000180422
2902	3686	GRIN1	-2.38	1.36E-05	4.35E-05
29842	7041	TFCP2L1	-2.38	3.85E-25	4.41E-24
10326	1518	SIRPB1	-2.38	1.46E-87	9.27E-86
25992	7015	SNED1	-2.39	6.05E-42	1.28E-40
2523	4072	FUT1	-2.39	1.02E-16	7.83E-16
23349	5839	PHF24	-2.40	4.91E-05	0.000145773
441478	2639	NRARP	-2.40	7.37E-22	7.26E-21

1295	3042	COL8A1	-2.40	0.00E+00	0.00E+00
9284	1349	NPIPA1	-2.40	2.42E-76	1.23E-74
55107	4410	ANO1	-2.40	6.44E-05	0.000187863
130497	1911	OSR1	-2.41	3.84E-13	2.33E-12
163051	4916	ZNF709	-2.41	2.61E-12	1.50E-11
9001	3880	HAP1	-2.42	3.86E-12	2.19E-11
10633	1071	RASL10A	-2.42	7.85E-06	2.58E-05
126147	2019	NTN5	-2.43	4.61E-07	1.73E-06
388799	692	FAM209B	-2.43	1.40E-05	4.45E-05
2645	2442	GCK	-2.43	6.34E-07	2.34E-06
7137	866	TNNI3	-2.43	1.27E-05	4.08E-05
23149	3061	FCHO1	-2.44	1.40E-14	9.46E-14
81610	2445	FAM83D	-2.46	3.54E-143	5.51E-141
25789	1712	TMEM59L	-2.46	3.01E-14	2.00E-13
25850	2945	ZNF345	-2.47	7.59E-13	4.52E-12
1278	5411	COL1A2	-2.47	2.73E-05	8.38E-05
147906	2834	DACT3	-2.47	6.06E-09	2.71E-08
57595	3782	PDZD4	-2.47	4.70E-24	5.11E-23
4486	4216	MST1R	-2.48	4.44E-08	1.84E-07
57509	4152	MTUS1	-2.49	3.87E-58	1.30E-56
9536	1787	PTGES	-2.49	1.79E-51	4.96E-50
3581	3771	IL9R	-2.49	2.11E-09	9.82E-09
3761	1913	KCNJ4	-2.49	8.05E-11	4.19E-10
7805	2223	LAPTM5	-2.50	3.61E-46	8.69E-45
25907	1876	TMEM158	-2.50	1.91E-23	2.02E-22
5803	6475	PTPRZ1	-2.50	3.02E-05	9.21E-05
127343	2881	DMBX1	-2.52	1.45E-06	5.16E-06
2944	1255	GSTM1	-2.52	2.10E-31	3.14E-30
5026	2266	P2RX5	-2.52	1.63E-28	2.16E-27
55190	2413	NUDT11	-2.52	1.38E-14	9.34E-14
9023	1378	CH25H	-2.52	4.09E-07	1.54E-06
26301	1962	GBGT1	-2.52	3.10E-39	6.06E-38
7851	2405	MALL	-2.52	1.37E-155	2.32E-153
3782	11956	KCNN3	-2.52	1.82E-07	7.10E-07
4583	15720	MUC2	-2.53	9.97E-06	3.24E-05
57574	4466	MARCH4	-2.53	6.22E-10	3.00E-09
147138	3163	TMC8	-2.53	1.72E-05	5.43E-05
3006	732	HIST1H1C	-2.53	9.98E-89	6.35E-87
1116	1867	CHI3L1	-2.54	2.34E-05	7.25E-05
347	1148	APOD	-2.54	1.53E-05	4.86E-05
2155	2892	F7	-2.54	2.26E-05	7.00E-05
149297	3638	FAM78B	-2.54	2.24E-05	6.96E-05
10659	7647	CELF2	-2.54	1.22E-07	4.84E-07
1746	2308	DLX2	-2.55	1.80E-10	9.08E-10
3560	4050	IL2RB	-2.55	1.32E-05	4.22E-05
482	3018	ATP1B2	-2.56	1.35E-18	1.14E-17
3872	1574	KRT17	-2.56	6.82E-35	1.15E-33
8912	7395	CACNA1H	-2.56	4.95E-18	4.07E-17

1757	3234	SARDH	-2.56	2.25E-22	2.27E-21
6563	3519	SLC14A1	-2.57	1.18E-05	3.80E-05
136853	2806	SSC4D	-2.57	4.72E-60	1.66E-58
8351	921	HIST1H3D	-2.58	2.05E-07	7.96E-07
55068	2706	ENOX1	-2.58	1.89E-09	8.82E-09
1E+08	6199	ZNF605	-2.58	1.25E-45	2.97E-44
28951	4408	TRIB2	-2.58	7.97E-92	5.41E-90
768239	4700	PSAPL1	-2.59	1.57E-05	4.98E-05
5646	803	PRSS3	-2.59	2.69E-06	9.29E-06
25830	2068	SULT4A1	-2.60	6.51E-12	3.64E-11
1281	5490	COL3A1	-2.60	1.19E-06	4.28E-06
283358	3438	B4GALNT3	-2.61	1.27E-09	6.00E-09
140862	2610	ISM1	-2.61	1.49E-38	2.85E-37
3670	2729	ISL1	-2.61	8.53E-10	4.08E-09
345462	2605	ZNF879	-2.62	2.00E-06	7.02E-06
220441	8803	RNF152	-2.62	2.30E-19	2.02E-18
1.05E+08	1166	LOC105371933	-2.62	1.15E-05	3.70E-05
85352	6741	SHISAL1	-2.63	1.10E-05	3.56E-05
5139	7319	PDE3A	-2.63	1.10E-05	3.54E-05
5175	6831	PECAM1	-2.63	1.10E-05	3.54E-05
7148	3143	TNXB	-2.64	1.73E-06	6.09E-06
80022	7312	MYO15B	-2.64	9.95E-27	1.22E-25
489	4739	ATP2A3	-2.64	5.52E-37	9.93E-36
164284	3439	APCDD1L	-2.65	5.19E-18	4.26E-17
148113	4199	CILP2	-2.65	1.87E-08	8.05E-08
4046	1578	LSP1	-2.66	8.62E-06	2.82E-05
1129	7372	CHRM2	-2.66	5.87E-07	2.18E-06
954	2087	ENTPD2	-2.66	3.09E-09	1.42E-08
1E+08	2590	FOXO6	-2.66	2.13E-22	2.16E-21
2977	16140	GUCY1A2	-2.67	4.35E-52	1.24E-50
6398	1797	SECTM1	-2.67	5.19E-25	5.92E-24
60680	2481	CELF5	-2.68	1.01E-09	4.82E-09
57451	9090	TENM2	-2.68	6.16E-26	7.30E-25
9568	5802	GABBR2	-2.69	3.93E-11	2.10E-10
147948	2928	ZNF582	-2.69	6.54E-06	2.18E-05
643596	1395	RNF224	-2.70	1.14E-08	4.99E-08
2318	9149	FLNC	-2.70	2.53E-197	6.62E-195
219699	6440	UNC5B	-2.70	5.26E-13	3.16E-12
162461	1497	TMEM92	-2.70	1.06E-38	2.03E-37
8639	2412	AOC3	-2.72	1.60E-10	8.12E-10
2548	3679	GAA	-2.72	6.74E-138	9.97E-136
338761	1987	C1QL4	-2.73	1.78E-32	2.75E-31
6096	3604	RORB	-2.73	2.65E-06	9.16E-06
51162	1288	EGFL7	-2.73	1.13E-78	5.95E-77
151473	4252	SLC16A14	-2.73	3.17E-45	7.49E-44
91409	1470	CCDC74B	-2.74	9.79E-31	1.42E-29
7643	3767	ZNF90	-2.74	5.53E-10	2.68E-09
10266	808	RAMP2	-2.74	4.91E-20	4.46E-19

9708	5503	PCDHGA8	-2.75	1.22E-21	1.19E-20
5307	2383	PITX1	-2.75	5.57E-33	8.73E-32
2044	7289	EPHA5	-2.76	2.40E-31	3.58E-30
126353	2891	MISP	-2.77	1.39E-07	5.48E-07
339983	5894	NAT8L	-2.77	1.40E-13	8.86E-13
136227	2751	COL26A1	-2.77	1.13E-10	5.82E-10
8904	2008	CPNE1	-2.79	0.00E+00	0.00E+00
1375	2798	CPT1B	-2.79	8.02E-76	4.02E-74
23251	7844	KIAA1024	-2.79	3.67E-14	2.42E-13
10630	2826	PDPN	-2.80	4.44E-201	1.25E-198
8348	467	HIST1H2BO	-2.80	2.42E-07	9.33E-07
286336	3479	FAM78A	-2.80	1.08E-12	6.35E-12
26032	4881	SUSD5	-2.82	1.34E-86	8.34E-85
151742	9903	PPM1L	-2.82	9.81E-81	5.38E-79
6545	2323	SLC7A4	-2.82	6.85E-08	2.79E-07
154091	5367	SLC2A12	-2.84	5.48E-99	4.23E-97
1002	6530	CDH4	-2.84	8.32E-144	1.31E-141
7111	3275	TMOD1	-2.85	3.13E-36	5.51E-35
3479	5422	IGF1	-2.85	1.06E-10	5.49E-10
22979	7433	EFR3B	-2.87	6.74E-11	3.53E-10
53358	11357	SHC3	-2.87	5.07E-26	6.03E-25
268	2065	AMH	-2.87	4.04E-52	1.15E-50
10736	2178	SIX2	-2.89	1.27E-09	6.00E-09
64093	3716	SMOC1	-2.90	1.42E-30	2.05E-29
1.03E+08	3887	HOTS	-2.91	2.06E-11	1.12E-10
729262	3292	NUTM2B	-2.91	2.43E-16	1.82E-15
2583	3687	B4GALNT1	-2.91	8.27E-101	6.71E-99
54097	1452	FAM3B	-2.92	7.81E-07	2.85E-06
1132	1817	CHRM4	-2.92	8.04E-10	3.85E-09
1009	3905	CDH11	-2.93	9.14E-44	2.07E-42
2869	2448	GRK5	-2.93	2.14E-23	2.26E-22
84709	1339	MGARP	-2.93	3.27E-17	2.58E-16
22843	6465	PPM1E	-2.94	4.62E-37	8.34E-36
57491	5622	AHRR	-2.94	9.95E-32	1.50E-30
79883	2477	PODNL1	-2.95	4.83E-95	3.49E-93
11033	2264	ADAP1	-2.95	6.97E-26	8.25E-25
3751	5333	KCND2	-2.96	6.31E-20	5.71E-19
7424	2103	VEGFC	-2.96	3.10E-68	1.31E-66
3142	2308	HLX	-2.98	3.24E-23	3.40E-22
9311	2314	ASIC3	-2.98	3.49E-12	1.99E-11
8642	10765	DCHS1	-2.98	7.39E-68	3.11E-66
57537	6078	SORCS2	-2.98	9.84E-93	6.85E-91
10343	7660	PKDREJ	-2.99	6.12E-08	2.51E-07
79844	4523	ZDHHC11	-2.99	8.82E-21	8.30E-20
6331	8338	SCN5A	-3.00	1.05E-22	1.07E-21
768	1561	CA9	-3.01	1.66E-08	7.14E-08
10234	2250	LRRC17	-3.01	5.55E-10	2.69E-09
54102	4048	CLIC6	-3.02	1.54E-08	6.65E-08

80303	1877	EFHD1	-3.02	5.39E-67	2.23E-65
2687	2283	GGT5	-3.04	8.17E-12	4.54E-11
54757	4202	FAM20A	-3.04	8.73E-12	4.84E-11
51450	1033	PRRX2	-3.04	1.79E-43	4.00E-42
4037	3714	LRP3	-3.05	1.16E-72	5.37E-71
4440	1239	MSI1	-3.06	8.88E-10	4.24E-09
80332	3435	ADAM33	-3.06	1.33E-16	1.01E-15
55964	4656	SEPT3	-3.06	3.28E-27	4.13E-26
90649	3897	ZNF486	-3.07	9.54E-23	9.78E-22
360205	518	PRAC2	-3.07	5.01E-11	2.65E-10
633	2223	BGN	-3.08	1.67E-13	1.04E-12
23428	4257	SLC7A8	-3.09	4.11E-09	1.86E-08
118738	3513	ZNF488	-3.09	1.20E-15	8.66E-15
51393	2564	TRPV2	-3.09	1.78E-25	2.07E-24
440193	5861	CCDC88C	-3.11	1.16E-47	2.91E-46
51316	1416	PLAC8	-3.12	1.89E-56	6.10E-55
7060	3109	THBS4	-3.12	3.55E-14	2.34E-13
6492	8436	SIM1	-3.13	2.27E-128	2.80E-126
2049	4234	EPHB3	-3.13	2.86E-09	1.32E-08
7448	1678	VTN	-3.13	7.70E-10	3.69E-09
8900	1892	CCNA1	-3.14	3.68E-29	5.03E-28
54331	3823	GNG2	-3.15	4.75E-61	1.73E-59
92815	496	HIST3H2A	-3.16	2.26E-10	1.13E-09
94137	7978	RP1L1	-3.16	6.23E-09	2.78E-08
57134	3480	MAN1C1	-3.17	3.93E-26	4.70E-25
128853	1112	DUSP15	-3.17	1.37E-13	8.65E-13
2124	2009	EVI2B	-3.17	8.16E-12	4.53E-11
9935	3395	MAFB	-3.20	1.29E-08	5.62E-08
9423	5954	NTN1	-3.21	1.27E-49	3.38E-48
1356	4517	CP	-3.21	6.35E-149	1.03E-146
57622	3332	LRFN1	-3.21	5.12E-14	3.35E-13
23098	7305	SARM1	-3.22	7.57E-43	1.67E-41
1.08E+08	3646	LOC107985728	-3.22	2.50E-14	1.66E-13
9254	5350	CACNA2D2	-3.23	6.21E-16	4.54E-15
641700	913	ECSCR	-3.23	8.53E-09	3.77E-08
126129	2807	CPT1C	-3.24	4.04E-18	3.33E-17
56776	4922	FMN2	-3.24	1.96E-29	2.72E-28
26468	3383	LHX6	-3.26	1.08E-30	1.57E-29
3606	1143	IL18	-3.26	9.34E-11	4.83E-10
3800	6958	KIF5C	-3.26	6.70E-39	1.29E-37
3400	3891	ID4	-3.27	1.77E-08	7.59E-08
5010	2661	CLDN11	-3.28	1.51E-164	2.79E-162
6909	3396	TBX2	-3.28	2.10E-11	1.13E-10
404037	3461	HAPLN4	-3.28	6.41E-11	3.37E-10
8745	6231	ADAM23	-3.28	1.14E-09	5.39E-09
54567	3420	DLL4	-3.29	1.06E-09	5.04E-09
2066	11600	ERBB4	-3.29	3.91E-10	1.92E-09
3481	4539	IGF2	-3.29	5.20E-23	5.41E-22

5228	1848	PGF	-3.29	2.17E-27	2.75E-26
25791	2183	NGEF	-3.30	2.59E-32	3.98E-31
390205	2230	LRRC10B	-3.31	7.99E-10	3.83E-09
10991	2489	SLC38A3	-3.32	1.56E-77	8.02E-76
134548	3474	SOWAHA	-3.32	1.05E-19	9.37E-19
23542	4090	MAPK8IP2	-3.34	5.01E-25	5.71E-24
148398	2575	SAMD11	-3.35	1.07E-51	2.99E-50
59284	2361	CACNG7	-3.37	4.63E-09	2.09E-08
56242	2351	ZNF253	-3.38	4.24E-15	2.95E-14
64388	4275	GREM2	-3.42	2.66E-09	1.23E-08
3852	2320	KRT5	-3.44	3.27E-19	2.85E-18
83648	3989	FAM167A	-3.44	4.46E-31	6.59E-30
8633	9879	UNC5C	-3.45	3.62E-31	5.36E-30
1800	2326	DPEP1	-3.45	1.02E-16	7.79E-16
22801	6107	ITGA11	-3.45	1.17E-73	5.60E-72
8843	2152	HCAR3	-3.46	3.45E-10	1.70E-09
151	3371	ADRA2B	-3.47	6.51E-11	3.42E-10
2192	2370	FBLN1	-3.48	0.00E+00	0.00E+00
349136	2408	WDR86	-3.49	2.89E-24	3.18E-23
6755	2657	SSTR5	-3.54	4.25E-32	6.50E-31
6330	4177	SCN4B	-3.55	8.73E-23	8.97E-22
83857	8719	TMTC1	-3.55	6.93E-26	8.21E-25
864	4042	RUNX3	-3.55	7.28E-14	4.69E-13
9945	3035	GFPT2	-3.55	5.64E-112	5.57E-110
138311	2334	FAM69B	-3.55	1.16E-61	4.28E-60
25960	6050	ADGRA2	-3.56	6.19E-73	2.88E-71
163933	2640	FAM43B	-3.58	3.31E-10	1.64E-09
56666	2984	PANX2	-3.59	2.80E-20	2.57E-19
10319	3996	LAMC3	-3.59	1.98E-13	1.23E-12
84969	2227	TOX2	-3.59	2.16E-35	3.68E-34
84674	3650	CARD6	-3.59	1.67E-32	2.60E-31
2151	3295	F2RL2	-3.61	6.74E-36	1.17E-34
728927	9049	ZNF736	-3.61	3.34E-41	6.95E-40
26051	5841	PPP1R16B	-3.62	1.82E-10	9.19E-10
79948	2321	PLPPR3	-3.62	2.32E-21	2.23E-20
619279	14403	ZNF704	-3.63	1.31E-19	1.17E-18
4004	693	LMO1	-3.63	8.90E-14	5.69E-13
64073	485	C19orf33	-3.64	4.26E-52	1.21E-50
1299	2485	COL9A3	-3.64	9.20E-84	5.32E-82
4052	5870	LTBP1	-3.66	4.64E-65	1.84E-63
5552	1314	SRGN	-3.67	2.16E-298	1.24E-295
145781	4662	GCOM1	-3.67	1.21E-10	6.18E-10
7781	1816	SLC30A3	-3.68	1.09E-17	8.81E-17
25758	8103	KIAA1549L	-3.68	1.04E-34	1.74E-33
4326	2432	MMP17	-3.69	6.96E-11	3.65E-10
23671	1796	TMEFF2	-3.69	2.75E-13	1.69E-12
9619	3805	ABCG1	-3.71	6.84E-68	2.89E-66
4811	5867	NID1	-3.71	0.00E+00	0.00E+00

3235	1902	HOXD9	-3.72	1.05E-130	1.40E-128
1833	1553	EPYC	-3.73	3.78E-11	2.02E-10
4330	7569	MN1	-3.73	2.50E-78	1.30E-76
57057	3002	TBX20	-3.74	4.44E-21	4.22E-20
1.01E+08	2820	TPBGL	-3.77	5.97E-19	5.12E-18
83700	3515	JAM3	-3.78	3.75E-56	1.20E-54
83439	2932	TCF7L1	-3.78	1.66E-42	3.61E-41
2624	3263	GATA2	-3.78	1.81E-32	2.80E-31
257407	3595	C2orf72	-3.79	2.89E-23	3.04E-22
348093	2019	RBPMS2	-3.79	3.80E-78	1.97E-76
162494	4512	RHBDL3	-3.81	7.97E-64	3.07E-62
7087	2068	ICAM5	-3.81	1.63E-25	1.89E-24
79047	3916	KCTD15	-3.82	1.55E-25	1.81E-24
389816	1212	LRRC26	-3.84	5.29E-21	5.01E-20
401399	2411	PRRT4	-3.86	8.63E-15	5.91E-14
1E+08	7424	ARMCX4	-3.87	5.34E-17	4.16E-16
136259	1480	KLF14	-3.88	3.62E-12	2.06E-11
23460	4940	ABCA6	-3.88	5.61E-16	4.11E-15
23416	3487	KCNH3	-3.89	1.45E-40	2.92E-39
57476	7883	GRAMD1B	-3.89	8.34E-27	1.03E-25
1366	1532	CLDN7	-3.90	5.96E-84	3.48E-82
9464	2368	HAND2	-3.90	2.64E-12	1.52E-11
60529	5466	ALX4	-3.91	2.75E-15	1.94E-14
6817	1396	SULT1A1	-3.92	4.06E-76	2.06E-74
2786	5123	GNG4	-3.93	4.62E-134	6.52E-132
84951	4073	TNS4	-3.95	1.87E-31	2.79E-30
92241	3723	RCSD1	-3.96	9.60E-13	5.68E-12
441027	1888	TMEM150C	-3.97	6.19E-22	6.12E-21
170850	3821	KCNG3	-3.98	6.54E-15	4.50E-14
55806	5528	HR	-3.98	1.65E-35	2.84E-34
1846	5625	DUSP4	-3.99	2.70E-62	1.01E-60
8913	7646	CACNA1G	-4.00	7.21E-23	7.44E-22
6474	3497	SHOX2	-4.01	9.23E-89	5.92E-87
4773	6732	NFATC2	-4.02	9.56E-44	2.16E-42
9244	1856	CRLF1	-4.03	2.80E-27	3.53E-26
338442	2082	HCAR2	-4.04	9.00E-18	7.30E-17
4804	3420	NGFR	-4.05	3.29E-47	8.16E-46
3055	2165	HCK	-4.05	2.37E-30	3.40E-29
153572	2760	IRX2	-4.08	1.10E-108	1.02E-106
126326	4317	GIPC3	-4.12	5.28E-61	1.91E-59
1404	4469	HAPLN1	-4.14	3.08E-15	2.17E-14
148252	3412	DIRAS1	-4.18	1.66E-20	1.54E-19
59271	2080	EVA1C	-4.19	1.01E-33	1.63E-32
5136	2592	PDE1A	-4.19	1.78E-223	5.75E-221
338707	3463	B4GALNT4	-4.20	5.26E-41	1.09E-39
6886	4171	TAL1	-4.29	3.69E-30	5.24E-29
5104	2352	SERPINA5	-4.30	1.92E-27	2.45E-26
1852	2293	DUSP9	-4.30	2.45E-19	2.16E-18

343578	2838	ARHGAP40	-4.32	2.35E-41	4.90E-40
283078	1649	MKX	-4.35	1.31E-22	1.34E-21
57593	2706	EBF4	-4.37	2.06E-44	4.74E-43
401207	5680	C5orf63	-4.38	7.25E-26	8.57E-25
83595	3278	SOX7	-4.38	1.31E-39	2.60E-38
5097	3698	PCDH1	-4.39	1.63E-101	1.33E-99
2018	2908	EMX2	-4.45	1.11E-16	8.47E-16
50649	4442	ARHGEF4	-4.46	2.65E-55	8.24E-54
2828	3014	GPR4	-4.47	7.42E-17	5.74E-16
56105	3308	PCDHGA11	-4.49	1.38E-78	7.23E-77
131583	3182	FAM43A	-4.53	5.57E-28	7.27E-27
1240	5110	CMKLR1	-4.55	1.31E-20	1.22E-19
53637	2351	S1PR5	-4.58	2.51E-32	3.86E-31
4854	8089	NOTCH3	-4.61	1.68E-109	1.58E-107
7020	3279	TFAP2A	-4.62	4.53E-87	2.84E-85
54039	1922	PCBP3	-4.62	9.75E-26	1.15E-24
3755	2237	KCNG1	-4.65	6.06E-30	8.55E-29
153478	9010	PLEKHG4B	-4.65	5.06E-59	1.73E-57
728118	5334	NUTM2A	-4.66	1.00E-53	2.99E-52
2022	3070	ENG	-4.67	5.94E-178	1.26E-175
5950	879	RBP4	-4.68	1.27E-59	4.44E-58
221662	2586	RBM24	-4.69	2.80E-22	2.82E-21
1301	5035	COL11A1	-4.73	5.57E-35	9.41E-34
2878	1779	GPX3	-4.80	0.00E+00	0.00E+00
23225	5462	NUP210	-4.81	0.00E+00	0.00E+00
4188	1451	MDFI	-4.86	7.73E-54	2.32E-52
8641	4578	PCDHGB4	-4.87	1.62E-60	5.75E-59
64321	2350	SOX17	-4.90	4.42E-38	8.34E-37
146664	4068	MGAT5B	-4.93	6.49E-31	9.52E-30
5087	5041	PBX1	-5.05	9.68E-54	2.90E-52
1271	1886	CNTFR	-5.26	2.14E-31	3.20E-30
65982	2716	ZSCAN18	-5.30	1.48E-77	7.63E-76
2564	2804	GABRE	-5.34	8.88E-75	4.35E-73
63934	6432	ZNF667	-5.35	1.50E-28	1.99E-27
25878	9800	MXRA5	-5.35	1.07E-136	1.53E-134
84366	403	PRAC1	-5.43	7.85E-27	9.72E-26
10170	1993	DHRS9	-5.44	2.34E-52	6.74E-51
2290	3216	FOXG1	-5.45	6.52E-35	1.10E-33
56603	4117	CYP26B1	-5.47	2.11E-40	4.25E-39
3910	6396	LAMA4	-5.47	3.80E-53	1.12E-51
1289	8402	COL5A1	-5.48	0.00E+00	0.00E+00
290	3658	ANPEP	-5.50	1.35E-186	3.16E-184
90187	3827	EMILIN3	-5.55	6.32E-107	5.70E-105
3237	1463	HOXD11	-5.58	7.63E-57	2.48E-55
4248	8485	MGAT3	-5.58	2.31E-42	4.99E-41
5739	2256	PTGIR	-5.70	1.26E-30	1.83E-29
84929	3119	FIBCD1	-5.91	1.18E-82	6.79E-81
1.08E+08	735	LOC107987373	-6.03	6.89E-51	1.89E-49

1497	4163	CTNS	-6.20	2.04E-62	7.65E-61
55287	1845	TMEM40	-6.21	1.08E-55	3.41E-54
55966	4214	AJAP1	-6.22	1.64E-38	3.12E-37
2995	1242	GYPC	-6.36	1.12E-40	2.28E-39
6422	4482	SFRP1	-6.59	1.49E-64	5.84E-63
23729	3838	SHPK	-6.76	5.12E-52	1.45E-50
4232	2326	MEST	-6.83	0.00E+00	0.00E+00
2313	3150	FLI1	-6.87	2.56E-98	1.95E-96
3236	1814	HOXD10	-7.02	2.10E-57	6.94E-56
51200	2817	CPA4	-7.60	0.00E+00	0.00E+00
91607	5015	SLFN11	-7.87	2.22E-113	2.22E-111
