# Supplementary figures and images for: Drug Repurposing in Rare Diseases: An Integrative Study of Drug Screening and Transcriptomic Analysis in Nephropathic Cystinosis
Source: Int J Mol Sci. 2021 Nov 27;22(23):12829. doi: 10.3390/ijms222312829 (PMC8657658; doi:10.3390/ijms222312829)

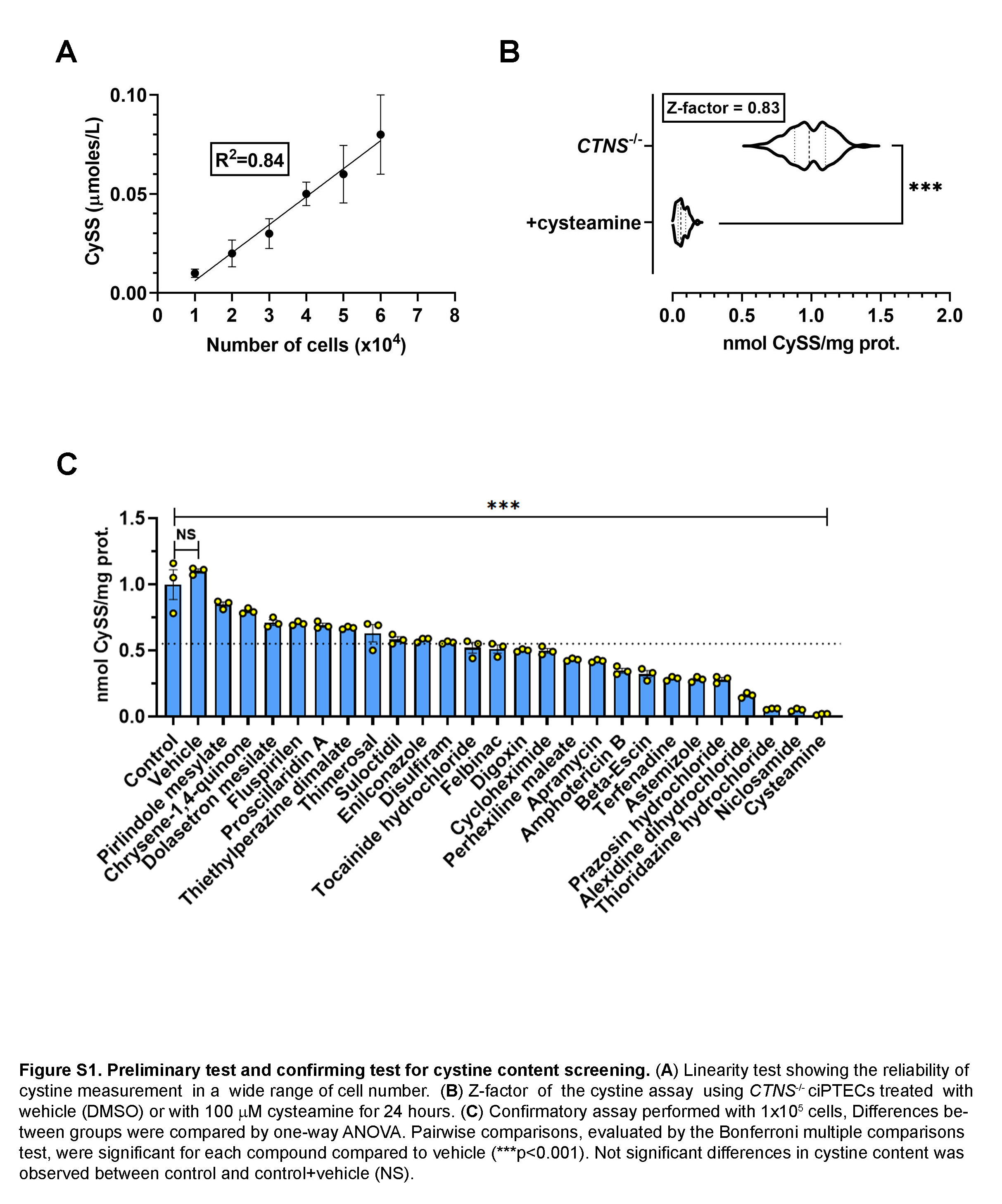

Supplement: Supplementary file 1 [file ijms-22-12829-s001.zip › Supplementary Figure S1.jpg]

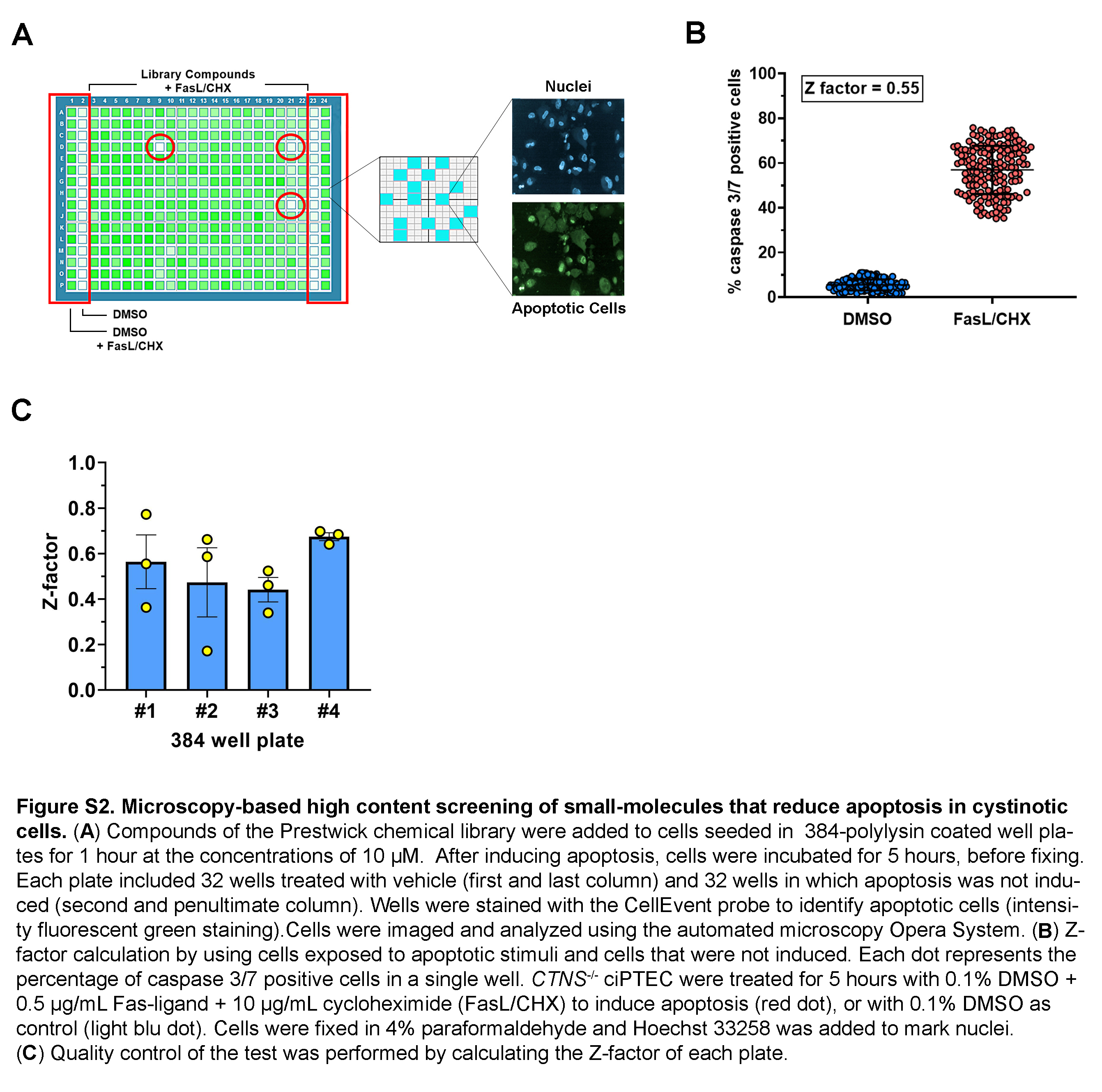

Supplement: Supplementary file 1 [file ijms-22-12829-s001.zip › Supplementary Figure S2.jpg]

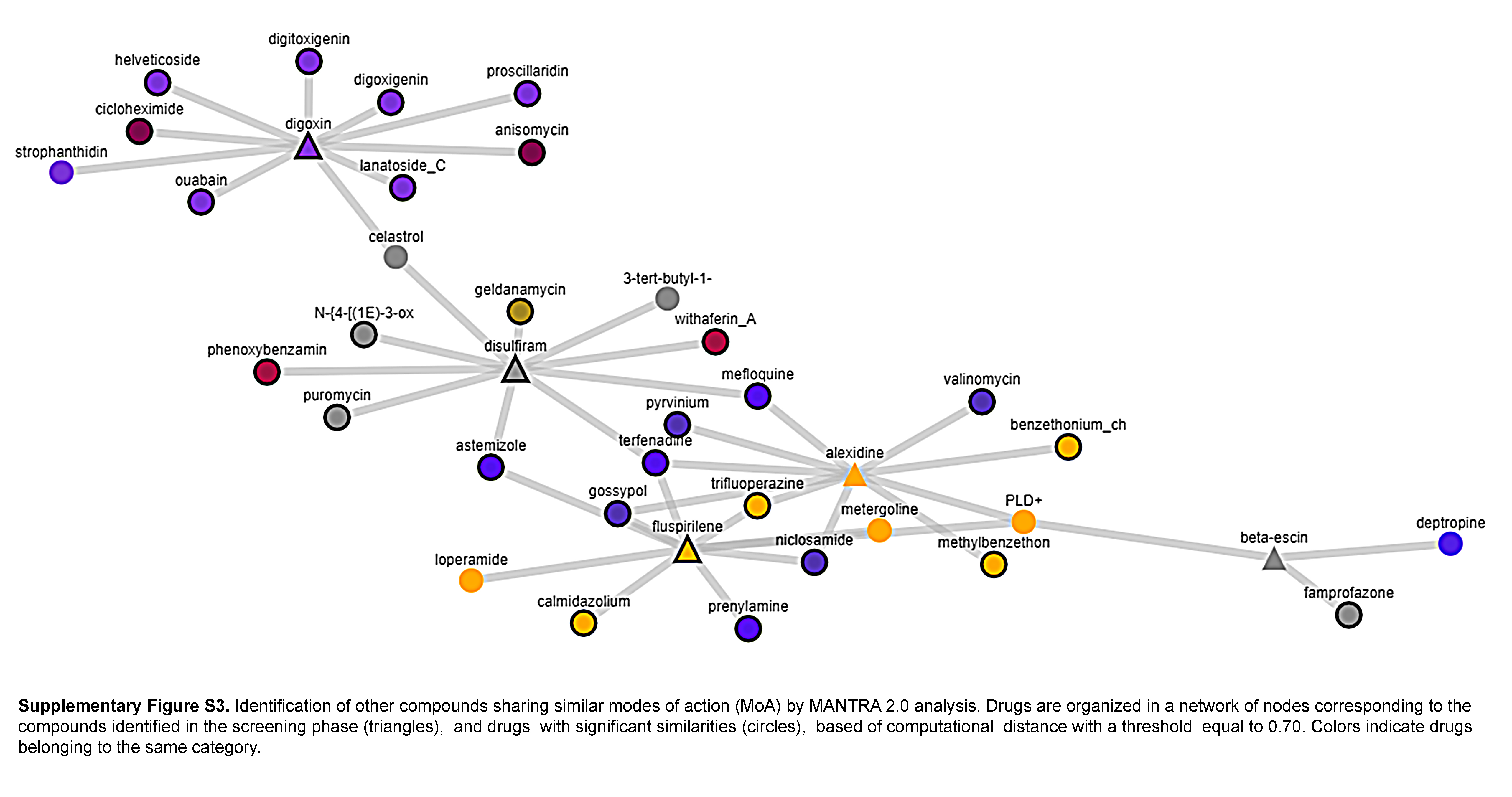

Supplement: Supplementary file 1 [file ijms-22-12829-s001.zip › Supplementary Figure S3.jpg]
